# Prediction of Carbohydrate Binding Sites on Protein Surfaces with 3-Dimensional Probability Density Distributions of Interacting Atoms

**DOI:** 10.1371/journal.pone.0040846

**Published:** 2012-07-25

**Authors:** Keng-Chang Tsai, Jhih-Wei Jian, Ei-Wen Yang, Po-Chiang Hsu, Hung-Pin Peng, Ching-Tai Chen, Jun-Bo Chen, Jeng-Yih Chang, Wen-Lian Hsu, An-Suei Yang

**Affiliations:** 1 Genomics Research Center, Academia Sinica, Taipei, Taiwan; 2 Institute of Information Sciences, Academia Sinica, Taipei, Taiwan; 3 Bioinformatics Program, Taiwan International Graduate Program, Institute of Information Science, Academia Sinica, Taipei, Taiwan; 4 Institute of Biomedical Informatics, National Yang-Ming University, Taipei, Taiwan; 5 Institute of Bioinformatics and Systems Biology, National Chiao-Tung University, Hsinchu, Taiwan; 6 Department of Computer Science, National Tsing-Hua University, Hsinchu, Taiwan; University of Cincinnati College of Medicine, United States of America

## Abstract

Non-covalent protein-carbohydrate interactions mediate molecular targeting in many biological processes. Prediction of non-covalent carbohydrate binding sites on protein surfaces not only provides insights into the functions of the query proteins; information on key carbohydrate-binding residues could suggest site-directed mutagenesis experiments, design therapeutics targeting carbohydrate-binding proteins, and provide guidance in engineering protein-carbohydrate interactions. In this work, we show that non-covalent carbohydrate binding sites on protein surfaces can be predicted with relatively high accuracy when the query protein structures are known. The prediction capabilities were based on a novel encoding scheme of the three-dimensional probability density maps describing the distributions of 36 non-covalent interacting atom types around protein surfaces. One machine learning model was trained for each of the 30 protein atom types. The machine learning algorithms predicted tentative carbohydrate binding sites on query proteins by recognizing the characteristic interacting atom distribution patterns specific for carbohydrate binding sites from known protein structures. The prediction results for all protein atom types were integrated into surface patches as tentative carbohydrate binding sites based on normalized prediction confidence level. The prediction capabilities of the predictors were benchmarked by a 10-fold cross validation on 497 non-redundant proteins with known carbohydrate binding sites. The predictors were further tested on an independent test set with 108 proteins. The residue-based Matthews correlation coefficient (MCC) for the independent test was 0.45, with prediction precision and sensitivity (or recall) of 0.45 and 0.49 respectively. In addition, 111 unbound carbohydrate-binding protein structures for which the structures were determined in the absence of the carbohydrate ligands were predicted with the trained predictors. The overall prediction MCC was 0.49. Independent tests on anti-carbohydrate antibodies showed that the carbohydrate antigen binding sites were predicted with comparable accuracy. These results demonstrate that the predictors are among the best in carbohydrate binding site predictions to date.

## Introduction

Many biological processes are driven by protein-carbohydrate interactions. Carbohydrates are the most prominent molecules displayed on mammalian cell surfaces. It has been estimated that more than 70% of human proteins are glycosylated [1] and in certain cell types, more than 80% of glycoconjugates are glycolipids [2] – it can be envisaged that all human cells are covered with high density of carbohydrates that are covalently linked to membrane glycoproteins, glycolipids, and other glycoconjugates on cell surfaces. In consistent with the abundance of the carbohydrates on cells, the cell-surface carbohydrates mediate molecular targeting in cell adhesion, signaling and migration of tumor cells, interactions between immune cells and microorganisms and recognition between pathogens and hosts [2]. All these processes are enabled mostly by non-covalent protein-carbohydrate interactions.

The determinants of non-covalent protein-carbohydrate interactions are hydrogen bonding and nonpolar interactions [3]–[7]. The axial and equatorial hydroxyl (OH) groups on the sugar ligands are both hydrogen bond donors and acceptors, and the sugar ring oxygen is a hydrogen bond acceptor. Moreover, the acetamido and carboxylate groups on the sugar moieties frequently participate extensive hydrogen bonding networks responsible for the specificity of the protein-carbohydrate interactions. These groups form direct hydrogen bonding networks with protein carboxyl groups as hydrogen bond acceptors and with protein main chain and side chain amide groups as hydrogen bond donors. Divalent cation- and water-bridged hydrogen bonds also involve as integral determinants of the protein-carbohydrate interactions. In addition to the highly hydrophilic groups, the sugar rings, formed by the aliphatic carbons and the protons covalently linked to them, frequently interact with protein aromatic side chains in the binding sites, and to a lesser extent, with alkyl side chains of hydrophobic residues in proteins. The concerted three-dimensional arrangement of the amino acid side chains and main chains dictates the specificity and affinity of the protein-carbohydrate interactions.

Although the polar and non-polar interactions as the main driving forces are shared by all non-covalent protein-carbohydrate recognitions, the three-dimensional arrangements of amino acids in carbohydrate binding sites are diverse. The main reason for the complexity is that the geometrical shape of a carbohydrate binding site determines the 3-D arrangement of the polar and nonpolar groups for carbohydrate-recognition: In shallow sugar binding sites on some lectins or carbohydrate binding modules, the bottoms of the binding sites are frequently lined with aromatic or aliphatic groups for the sugar ring lying flat on the binding surfaces; in deep sugar binding crevices on some carbohydrate processing enzymes or carbohydrate transporting proteins, the bottoms of the crevices are frequently lined with polar groups to form hydrogen bonding networks with the OH groups or other polar groups on the sugar rings while the aromatic side chains interacting with the sugar rings are frequently distributed on the side walls of the crevices. The complex geometry of the carbohydrate-recognition surfaces makes computational prediction of the non-covalent carbohydrate binding sites on protein surfaces a difficult challenge.

Computational prediction methods for carbohydrate binding site on proteins have been described: Taroni et al. [8] analyzed characteristic propensities of amino acids in sugar binding sites of proteins with known structure. These propensities were optimized to calculate the probability for a protein surface patch to be a carbohydrate binding site. Shionyu-Mitsuyama et al [9] constructed empirical rules for carbohydrate-protein interactions by building three-dimensional probability density maps of protein atoms around monosaccharide moieties. Tentative carbohydrate binding sites on a protein of known structure were then marked with high score based on the match of the protein surface atom distribution with the probability density maps. Malik and Ahmad [10] used neural network trained with evolutionary sequence information to predict carbohydrate binding site on proteins. Kulharia et al [11] established a two step-method to identify tentative sugar binding site on proteins and then to evaluate the sites with amino acid propensity scores. Nassif et al [12] predicted protein-glucose binding sites by representing a tentative glucose binding site as a vector of geometric and chemical features and the key features were selected for support vector machines predictions. These methods are successful to various extents in predicting carbohydrate binding sites on proteins, and yet prediction accuracy remains to be improved.

The goal of this work is two-fold: to develop an accurate predictor to recognize non-covalent carbohydrate binding sites on protein surfaces and to identify the key residues in the tentative binding sites. The binding site locations and the key residues involving in the carbohydrate binding are essential information for further experimental investigations, which might include site-directed mutagenesis experiments, therapeutics design targeting against proteins interacting with carbohydrate ligands, and engineering protein-carbohydrate interactions. The predictions were carried out with machine learning algorithms, designed to recognize interacting atom distribution patterns associated with carbohydrate binding atoms on protein surfaces. In order to carry out the machine learning procedure, protein surface atoms were first categorized into 30 atom types; one machine learning model was trained for each of the atom types. Each of the input attributes for the machine learning models was the normalized distance-weighted sum of the three-dimensional probability density of one of the 36 interacting atom types on the protein surface – 5 atom types are from carbohydrate ligands, 30 from proteins, and 1 from water. The probability density maps around a protein atom were constructed based on the three-dimensional distribution of the non-covalent interacting atoms around the same protein atom type in the protein structure database. The machine learning models learned the patterns of the attributes to distinguish binding site atoms from non-binding site atoms, and the likelihood of a protein surface atom to involve in a putative carbohydrate binding site was normalized into a prediction confidence level between 0 and 1. Protein surface atoms with high confidence level from the predictors were clustered to yield tentative carbohydrate-binding patches. The prediction capabilities of the predictors were first benchmarked by 10-fold cross validations on 497 non-redundant proteins with known carbohydrate-binding structures. The predictors were then tested on an independent test set with 108 proteins that had not been included in the training-validation set of 497 proteins. Moreover, 111 unbound carbohydrate-binding proteins were tested with the trained predictors to benchmark the prediction capabilities for proteins structures of unknown carbohydrate binding sites. The computational methodology was further tested to predict the carbohydrate binding surfaces and the key residues in the binding sites in 15 non-redundant anti-carbohydrate antibodies; none of these antibody-carbohydrate complexes were used for training/validating the predictors. The prediction results showed that the predictors developed in this work were not only able to identify putative carbohydrate binding sites on proteins with relatively superior accuracy, they could also provide information on the residues key to the carbohydrate recognitions.

## Results

### Protein surface attributes characterizing carbohydrate interaction sites on proteins

**Figure 1:**
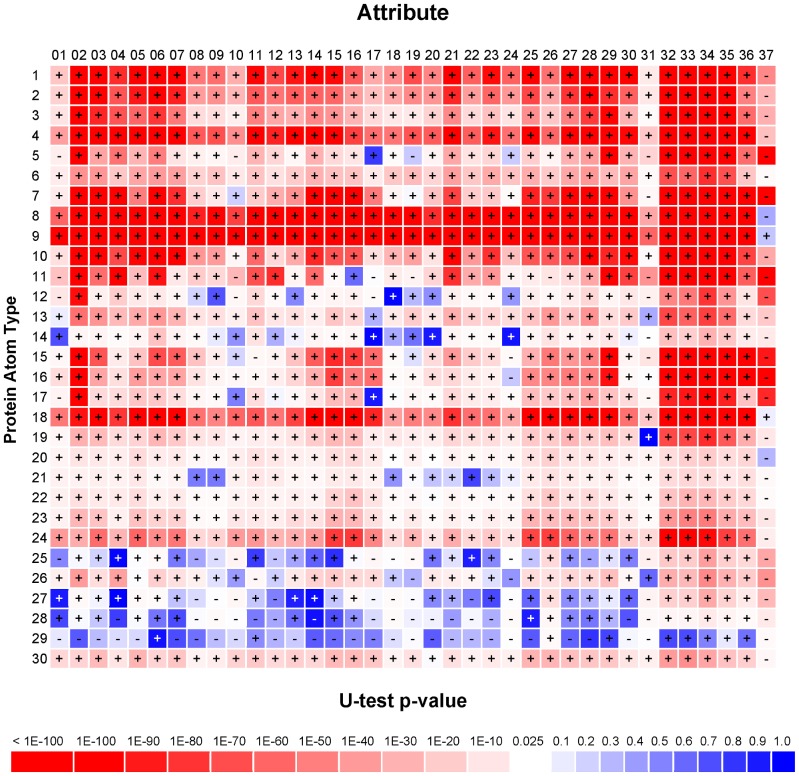
The p-values of Mann-Whitney U-tests on the 37 attributes for each of the 30 atom types in proteins. The p-values were calculated with the Mann-Whitney U-test implemented as the function ranksum in MATLAB. Two sets of data were input to the function and the output p-value is the probability for the two distributions of data to be statistically indistinguishable. More details on the U-tests shown in this figure are described in the associated text.

The protein surface attributes characterizing protein-carbohydrate interaction sites are highlighted in Figure 1. The y-axis in Figure 1 is the atom type index *i* = 1∼30 (atom types 1∼30 in Table 1), and the x-axis is the index *j* = 1∼36 for the 36 interacting atom types (atom types 1∼36 in Table 1) and the 37^th^ feature reflecting the local geometry of the protein surface (see Methods). The matrix elements in Figure 1 are the Mann-Whitney U-test p-value results (see Methods). Here, the values used in each of the U-tests for feature *j* of atom type *i* are *A_i,j_* as shown in Equation (2) for *i* = 1∼30 and *j* = 1∼36 or *a_i,37_* for *i* = 1∼30 as shown in Equation (3). One of the two groups of values for each of the U-tests consisted of *A_i,j_* (or *a_i,37_*) calculated for the protein surface atoms of type *i* in carbohydrate-binding sites in the S497 dataset and the other group consisted of *A_i,j_* (or *a_i,37_*) calculated for non-carbohydrate binding atoms of type *i* in the same dataset. The p-value of the U-test is color-coded as shown in the Figure; the plus(+) sign in the matrix element indicates that the averaged feature value for the carbohydrate-binding site atoms is larger than the averaged feature value for the non-carbohydrate-binding site atoms and the negative(−) sign indicates the opposite.

**Table 1:** Protein and carbohydrate atom types.

ID #	Atom Type	Radius(Å)	Description
1	NH1	1.65	Backbone NH
2	C	1.76	Backbone C
3	CH1E	1.87	Backbone CA (exc. Gly)
4	O	1.40	Backbone O
5	CH0	1.76	Arg CZ, Asn CG, Asp CG, Gln CD, Glu CD
6	CH1S	1.87	Sidechain CH1: Ile CB, Leu CG, Thr CB, Val CB
7	CH2E	1.87	Tetrahedral CH2 (except CH2P,CH2G) All CB
8	CH3E	1.87	Tetrahedral CH3
9	CR1E	1.76	Aromatic CH (except CR1W, CRHH, CR1H)
10	OH1	1.40	Alcohol OH (Ser OG, Thr OG1, Tyr OH)
11	OC	1.40	Carboxyl O (Asp OD1, OD2, Glu OE1, OE2)
12	OS	1.40	Sidechain O: Asn OD1, Gln OE1
13	CH2G	1.87	Gly CA
14	CH2P	1.87	Pro CB, CG, CD
15	NH1S	1.65	Sidechain NH: Arg NE, His ND1, NE1, Trp NE1
16	NC2	1.65	Arg NH1, NH2
17	NH2	1.65	Asn ND2, Gln NE2
18	CR1W	1.76	Trp CZ2, CH2
19	CY2	1.76	Tyr CZ
20	SC	1.85	Cys S
21	CF	1.76	Phe CG
22	SM	1.85	Met S
23	CY	1.76	Tyr CG
24	CW	1.76	Trp CD2, CE2
25	CRHH	1.76	His CE1
26	NH3	1.50	Lys NZ
27	CR1H	1.76	His CD2
28	C5	1.76	His CG
29	N	1.65	Pro N
30	C5W	1.76	Trp CG
31	HOH	1.40	Water
32	O.RX	1.40	Ring oxygen
33	O.LX	1.40	Oxygen of hydroxyl group
34	C.RX	1.87	Ring carbon
35	C.LX	1.87	Non-sugar carbon
36	N.LX	1.65	Nitrogen of N-acetyl group

The protein atom types 1∼31 have been previously defined by Laskowski et al [23] with minor modifications. The atom types from 32 to 36 were defined in this work for carbohydrate molecules.

The results of the U-tests shown in Figure 1 indicate that the surface attributes (*A_i,j_* and *a_i,j_* in Equation (2) and Equation (3) respectively) are encoded with substantial information in distinguishing the carbohydrate-binding site atoms from non-carbohydrate-binding surface atoms. With the conventional threshold of p-value = 0.025, the matrix elements colored in red (p-value<0.025) correspond to the atom types (y-axis) and the PDM/geometric features (x-axis) that characterize carbohydrate binding sites on protein surfaces. As expected, the U-test results for the geometric features *a_i,37_* in Figure 1 (column 37) reflect the fact that carbohydrate binding sites are concaved cavities on protein surfaces. The five *A_i,j_, j* = 32∼36, are consistently enriched around carbohydrate-binding site atoms (columns 32∼36 in Figure 1). The enrichment is due to the additive effect of the corresponding PDMs associated with the clustered carbohydrate-binding atoms. In addition, as highlighted by the matrix elements colored in red in Figure 1, these carbohydrate-binding atoms include several protein atom types *i*, especially for *i* = 1∼4 (backbone atoms), 6∼9 (non-polar carbon atoms), 10∼11 (polar oxygen atoms), 15∼16 (polar nitrogen atoms), and 18, 24, 30 (tryptophan atoms), and to a lesser extent *i* = 19,23 (tyrosine atoms). These results of the U-test p-values in Figure 1 are in good agreement with previous statistically surveys, where protein-carbohydrate interactions are governed by direct or water/cation-mediated hydrogen bonding involving main chain and side chain polar oxygen and nitrogen atoms and by non-polar interactions with aromatic and aliphatic side chains, in particular tryptophan and tyrosine [3]–[6], [8]. The U-test results in Figure 1 shows that the PDM/geometric features as described in Equations (2)∼(3) are effective attributes in distinguishing the carbohydrate-binding site atoms on protein surfaces.

### Atom-based protein surface carbohydrate binding site predictions with machine learning models

**Figure 2:**
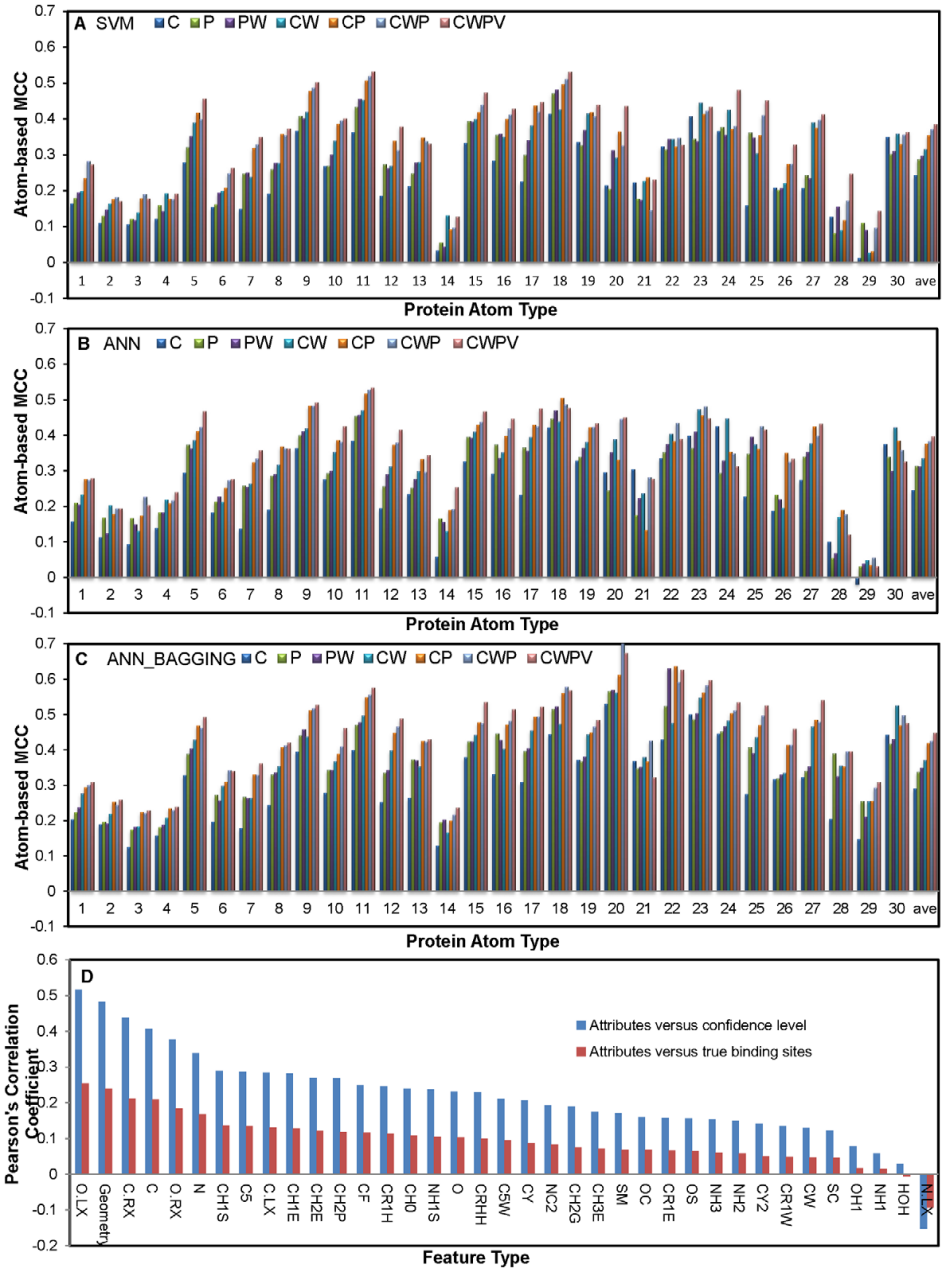
Prediction accuracy on carbohydrate binding for each of the 30 atom types on proteins. Panels A, B, and C show prediction results from the 10-fold cross validation on the S497 set with SVM, ANN, and ANN_BAGGING machine learning algorithm respectively. The numbers below the x-axis are the indexes of the protein atom types shown in Table 1. The accuracy averaged over the 30 atom types is shown towards the end of the x-axis. The y-axis shows the 10-fold cross validation MCC (Equation (9)) of the machine learning results. For each of the predictions, 7 different combinations of the 37 attributes (Equation (3)) were used as input sets for the machine learning algorithm: C – attributes 32∼36; P – attributes 1∼30; PW – attributes 1∼31; CW – attributes 31∼36; CP – attributes 1∼30 and 32∼36; CWP – attributes 1∼36; CWPV – attributes 1∼37. In panel D, the blue histogram shows the correlations between prediction confidence level derived from the predictions shown in panel C and attributes derived from concentrations of PDMs. The prediction confidence level for each surface atom to be in a carbohydrate binding site is correlated to various extents with the 37 attributes (*a_i,j_* (*j* = 1∼37) as shown in Equation (3)), which were used as inputs for the machine learning predictors in making the predictions. The Pearson's correlation coefficients, which are the measurements for the linear correlations between the prediction confidence level and the attributes, are shown in the y-axis. The x-axis shown the feature types, each of which corresponds to one of the *a_i,j_* (*j* = 1∼37, see Table 1 and Equation (3)). In panel D, the red histogram shows the Pearson's correlations between positive or negative assignment in carbohydrate binding and the same attributes derived from concentrations of PDMs. The positive assignments were given a value of 1 and negative assignments were given a value of 0.

The Mann-Whitney U-tests shown in Figure 1 suggest that the attributes (*a_i,j,_,i = 1∼30, j = 1∼37*) as described in Equation (3) for each of the 30 protein atom types are statistically significant in distinguishing carbohydrate binding atoms on protein surface from non-carbohydrate-binding surfaces. Based on the premise, machine learning models were trained and cross-validated (10 folds on S497 set, see Methods) for each of the 30 protein atom types; each of the machine learning models used the 37 attributes as inputs. The contributions of the attributes to the prediction accuracies were tested and the results are shown in Figure 2.

Figure 2A shows the cross-validated prediction accuracies (MCC) of the SVM predictors for each of the 30 protein atom types. The subset of attributes *a_i,j,_, j* = 32∼36 were derived from the corresponding PDMs of the interacting atoms from carbohydrate ligands and were expected to contribute to a large extent to the prediction capability. With this set of attributes as inputs, the SVM predictors were able to reach the overall average accuracy of MCC = 0.25. With more attributes added as inputs, the accuracy of the SVM predictors increased. The maximal overall average accuracy of MCC = 0.38 was reached when all the 37 attributes were used as inputs for predictor training.

Figure 2A also shows that the predictors for some of the atom types were more accurate in distinguishing carbohydrate-binding site atoms from non-binding atoms. The trends are in good agreement with the p-values of the U-tests: For atom types 14, 28, and 29 for which the U-test p-values were relatively large across the 37 attributes (Figure 1), the prediction accuracies were expectedly inferior. In contrast, predictors for the atom types with the most significant U-test p-values were among the most accurate predictors. Nevertheless, the predictors for the main chain atoms (atom types 1∼4,) were among the worst in prediction accuracy (Figure 2), although the p-values shown in Figure 1 were relative small for these atom types. These results suggest that small U-test p-value is necessary but not sufficient condition for accurate predictors.

The ANN and ANN_BAGGING predictors showed the same general trend in prediction accuracy as the SVM predictors. Figure 2B shows the results from the ANN predictors. The results are highly comparable with the results shown in Figure 2A, indicating that the machine learning predictors from both methodologies converged to similar optimized prediction capabilities. Figure 2C shows that the ANN_BAGGING models further improved the prediction accuracy for all the 30 atom types and the average overall accuracy were MCC = 0.45. The improvements were particularly significant for atom types 14, 28, and 29. These improvements in ANN_BAGGING predictors are attributed to the superior methodology in handling imbalanced distribution of the positive and negative cases in the training dataset [13].

The blue histogram in Figure 2D shows the Pearson's correlation coefficients between the prediction confidence level and the attributes of various types (*j* = 1∼37, see Table 1) calculated in Equation (3) for protein surface atoms. As shown in the histogram, increasing prediction confidence level is correlated with increasing value of the attributes derived from carbohydrate ligands (O.LX, C.RX, O.RX, C.LX, see Table 1). This indicates that, as expected from Figure 1 and Figures 2A∼2C, these attributes contribute substantially to the prediction capability. In addition, the geometry attribute also provides informative characteristics in supporting prediction confidence. The attributes derived from PDMs of protein hydrophilic atoms (NH3, NH1, NC2, OH1, NH1S, OC, NH2, OS, see Table 1) are less correlated with prediction confidence level (Figure 2D), suggesting that the patterns of the PDMs for the hydrophilic atoms are less distinguishable between the carbohydrate binding sites and the protein surfaces. The attribute derived from the PDM of N.LX on carbohydrate ligands is negatively correlated with prediction confidence level, suggesting that protein surface patches with denser PDM of N.LX are less likely to be carbohydrate binding sites.

The red histogram in Figure 2 D shows the Pearson's correlation coefficients between the positive (1) or negative (0) assignments for carbohydrate binding and the attribute values for atoms on the protein surfaces. In theory, attributes (x-axis) correlated to the positive or negative assignments with higher correlation coefficients (y-axis) should contribute statistically more weight in prediction accuracy. This expectation has been validated by the almost identical trends in comparing the red histogram with the blue histogram shown in Figure 2D, indicating that indeed the contributions of the features to the prediction accuracy as indicated in the previous paragraph are in good agreement with the statistical expectations.

### Residue-based protein surface carbohydrate binding site predictions with machine learning models

The atom-based predictions as described in the previous section were further processed into residue-based predictions by converting the outputs of the atom-based machine learning models into confidence levels (see Methods section). The confidence level measurement allows predictions for various protein atom types to be integrated on a normalized ground so that tentative carbohydrate binding sites can form a surface patch composed of various atom types with high confidence level in atom-based predictions. This methodology, combining the atom-based predictions into residue-based predictions of carbohydrate binding patch, increased the prediction accuracy to MCC = 0.5, as shown in the summary of Table 2.

**Table 2:** Carbohydrate binding site prediction accuracy benchmarks for 10-fold cross validations and independent tests.

Dataset/method	Acc	Pre	Sen	Spe	MCC	Fsc
S497/ANN	0.95(0.953±0.003)	0.50(0.503±0.049)	0.52(0.522±0.044)	0.97(0.974±0.003)	0.49(0.487±0.032)	0.51(0.511±0.032)
S497/SVM	0.96(0.955±0.003)	0.53(0.525±0.045)	0.51(0.504±0.046)	0.98(0.977±0.002)	0.49(0.49±0.037)	0.52(0.513±0.036)
S497/ANN_BAGGING	0.95(0.954±0.003)	0.51(0.512±0.037)	0.54(0.54±0.036)	0.97(0.974±0.001)	0.50(0.501±0.031)	0.53(0.525±0.031)
S108/ANN_BAGGING	0.96	0.45	0.49	0.97	0.45	0.47

The carbohydrate binding site predictions were carried out with the ANN, SVM and ANN_BAGGING algorithm on the proteins from the S497 dataset and the prediction accuracy of the ANN_BAGGING on the independent test set S108. Matthews correlation coefficient (MCC), F-score(Fsc), Accuracy(Acc), Precision(Pre), Sensitivity(Sen) and Specificity(Spe) are shown in Equations (4)∼(9). The benchmarks for the ten-fold cross validation results (i.e., S497/ANN, S497/SVM, and S497/ANN_BAGGING) are shown in two ways: The values enclosed in parenthesis are the averaged benchmark value and standard deviation calculated from the ten-fold results; the values above the average±standard-deviation pairs are the overall benchmark values calculated with the combined TP, TN, FP, FN cases from the test sets of the ten-fold cross-validation results.

The machine learning predictors were converged to optimized prediction capabilities in the ten-fold cross validations for all the machine learning algorithms tested. As shown in Table 2, the overall benchmarks are essentially indistinguishable from the averaged benchmarks (in parenthesis) derived from averaging the ten-fold cross validation results. In addition, the standard deviations of the ten-fold cross validation benchmarks are consistently small for the three machine learning models (Table 2). These results indicated that the machine learning models were optimized with stably generalizable prediction capabilities for new protein structures which had not been used in training sets.

**Figure 3:**
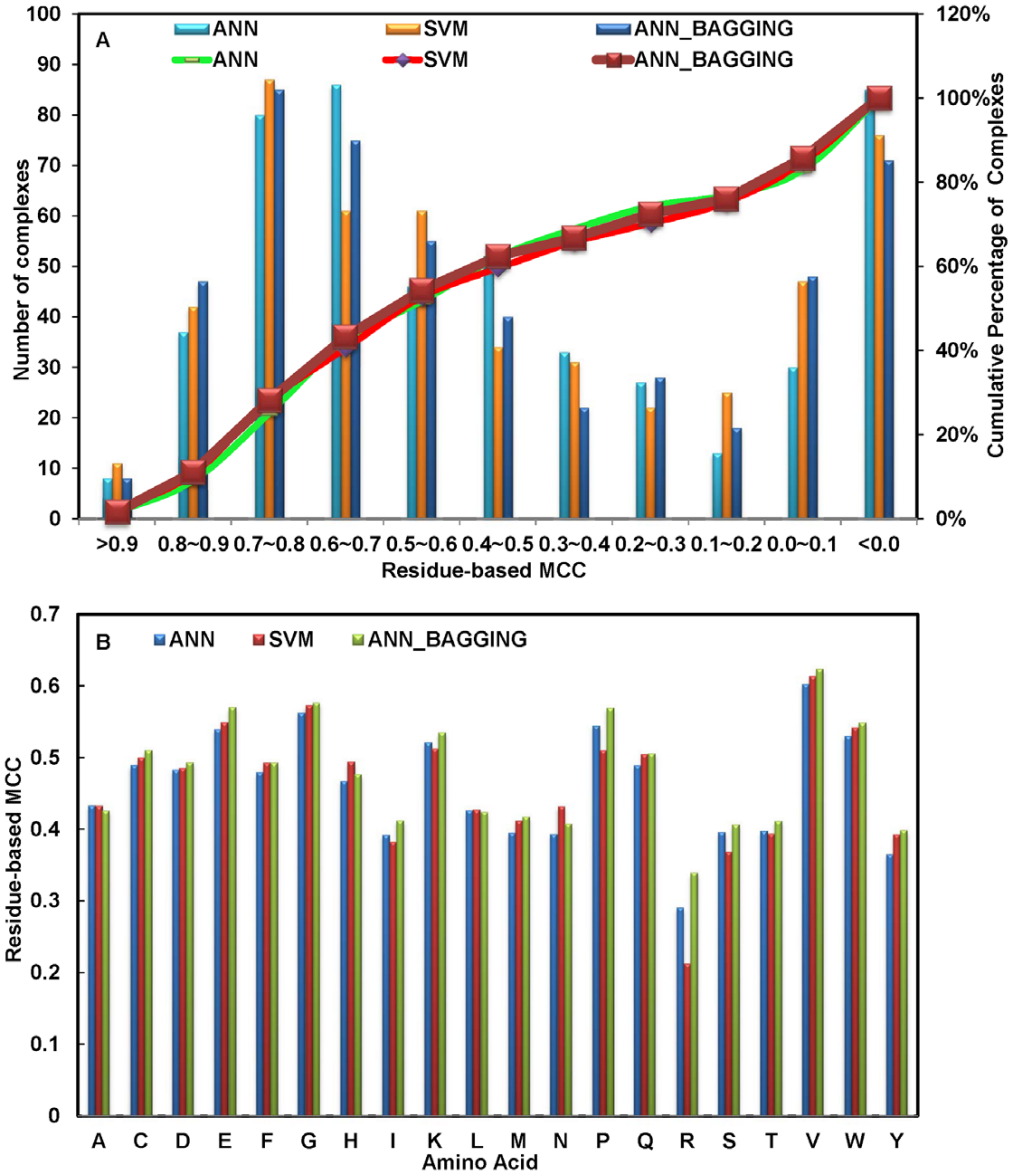
Carbohydrate binding site prediction accuracy based on the 10-fold cross validation on the S497 dataset. (A) Proteins in S497 set were sorted according to the prediction MCC for each of the proteins. Detailed prediction results for each of the cases in the S497 set are shown in Tables S2, S3, and S4. For each of the MCC ranges shown in the x-axis, the histograms show the number (as shown by the y-axis on the left-hand side of the panel) of the S497 proteins predicted with the MCC within the designated MCC range. The three sets of the predictions shown in the panel were carried out with the ANN, SVM, or the ANN_BAGGING algorithm respectively, and all the 37 attributes were used as inputs for the machine learning algorithms. The superimposed curves are the cumulative percentage of proteins in the S497 set that were predicted with MCC greater than the threshold indicated in the x-axis. The percentage is shown by the y-axis on right-hand side. (B) Residue-based MCC (y-axis) for each of the 20 natural amino acids (x-axis) were calculated from the results of the 10-fold cross validation on the S497 set. Again, the three sets of the predictions shown in the panel were carried out with the ANN, SVM, or the ANN_BAGGING algorithm respectively.

The three types of machine learning algorithm showed little difference in the residue-based prediction accuracy (Table 2). The distribution of the prediction accuracy for carbohydrate binding sites on proteins is shown in Figure 3A. As shown in the Figure, the prediction capabilities of the three types of machine learning algorithm were about the same in prediction accuracy. Empirically, the carbohydrate binding site on a protein can be correctly identified with MCC>0.2. By this criterion, 72.6% of the proteins in the S497 set can be predicted with reasonable confidence for the carbohydrate binding sites with the ANN_BAGGING prediction method (Figure 3A). Figure 3B shows the distribution of the prediction accuracy versus the 20 natural amino acid types. The results indicate that predictions for the residue types of VAL, GLY, GLU, PRO, TRP, LYS, CYS and GLN were relatively accurate.

**Figure 4:**
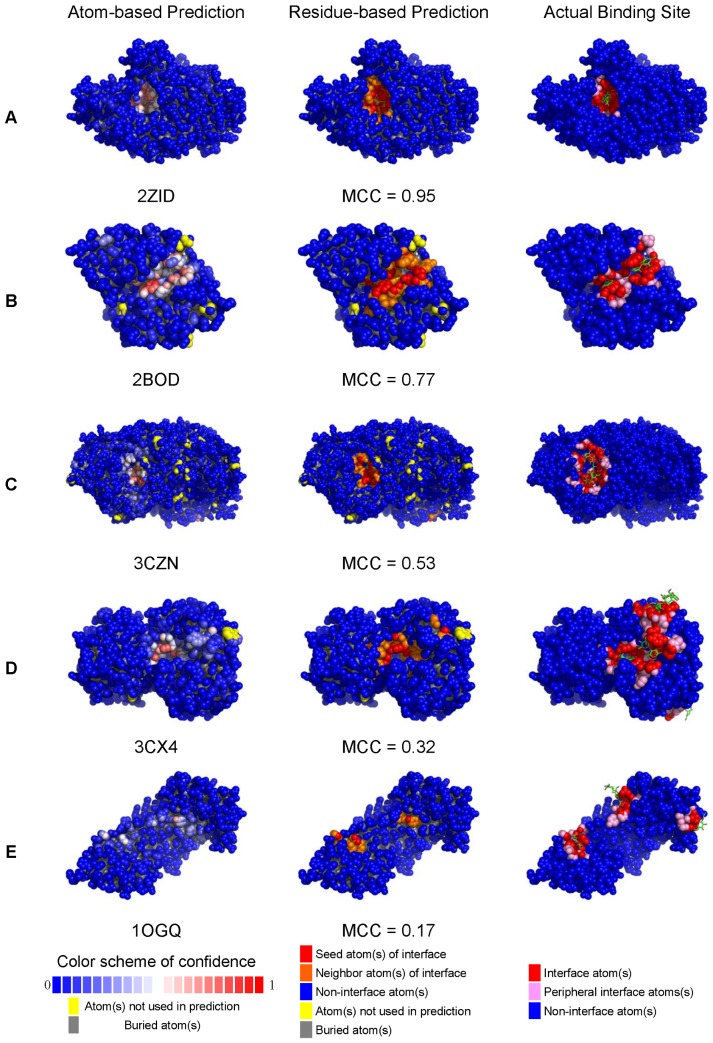
Examples of carbohydrate binding site predictions on proteins in the S497 set. Each of the atoms in the protein models of the atom-based predictions (left-hand column) is color-coded according to the prediction confidence level for the atom to involve in carbohydrate binding. The color code for the confidence level is shown as a colored bar at the bottom of this column. Atoms colored in red of various level of depth are the seeds for the carbohydrate binding site patch predictions. The protein models of the middle column show the residue-based carbohydrate binding site predictions. The residues predicted to be in the carbohydrate binding sites are colored in red or orange. The red atoms were predicted with confidence level greater than 0.5; atoms in the orange portion are the atoms belonging to the residues in the residue-based carbohydrate binding site prediction but the prediction confidence levels are less than 0.5. The protein models of the right-hand side column are color-coded for the residues in close contact with the carbohydrate ligands. The atoms colored in red are within 5 Å distance to any atom of the corresponding carbohydrate ligand. The atoms colored in pink are the atoms in the contact residues but are not within 5 Å to the corresponding carbohydrate ligand. The PDB code name and the MCC for each of the examples are also shown. The protein models showing the carbohydrate binding site predictions for each of the proteins in the S497 dataset are available for interactive examination from the web webserver http://ismblab.genomics.sinica.edu.tw/> benchmark > protein-carbohydrate.

A few examples of protein-carbohydrate binding site predictions in S497 with various MCC for the binding site prediction are shown in Figure 4. Table S2 (for ANN predictions), Table S3 (for SVM predictions), and Table S4 (for ANN_BAGGING predictions) list the details of the prediction accuracy benchmarks for each of the proteins in the S497 set. Interactive examination of the prediction results for each of the proteins in the S497 dataset can be accessed from the web server: http://ismblab.genomics.sinica.edu.tw/> benchmark > protein-carbohydrate.

Benchmarks for the independent test of the ANN_BAGGING predictions on the S108 test set are also shown and compared with those of the 10-fold cross validation in Table 2. The independent test MCC of 0.45 is slightly inferior to the MCC of 0.5 from the 10-fold cross validation, but nevertheless, the results indicate that the prediction methods can be generalized with reasonable accuracy to query protein structures not included in the training and test sets. Detailed benchmarks for each of the proteins in the S108 set are shown in Table S5. The maximum sequence identity for each of the proteins in S108 to any proteins in S497 is also shown in Table S5. Evidently, prediction accuracy is not correlated with sequence similarity of the two datasets. Interactive examination of the prediction results for each of the S108 proteins can be accessed from the web server: http://ismblab.genomics.sinica.edu.tw/> benchmark > protein-carbohydrate.

**Figure 5:**
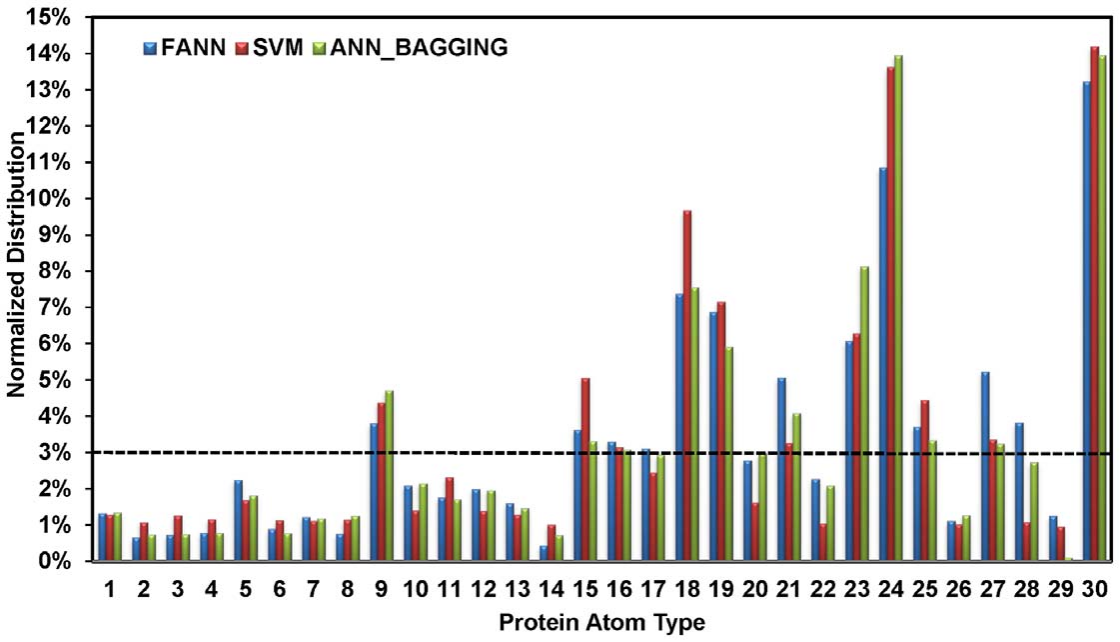
Distributions of protein atom types in the predicted atom-based carbohydrate binding sites in proteins. The percentage *P_i_* (shown in the y-axis of the panel) for the atom type *i* is calculated by the equation below: 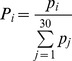
, where 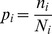 *N_i_* is the total number of atom type *i* in the dataset, while *n_i_* is the number of atom type *i* with prediction confidence level greater than 0.1 based on the ANN, SVM, or ANN_BAGGING predictions on the S497 set. In completely random predictions, *P_i_∼*1/30 for *i = *1∼30. This baseline is shown as the dashed line in the figure. When *P_i_>*1/30, the atom type *i* is more frequent to appear in the predicted atom-based carbohydrate binding sites.

Only a few side chain atom types are the key determinants in predicting a carbohydrate binding site on proteins. Figure 5 shows the distributions of the atom type composition in predicted carbohydrate-binding sites. The atom type distributions are mostly dominated by side chain atoms from Trp (atom types 9, 18, 24, 30 in Figure 5), Tyr (atom types 9, 19, 23 in Figure 5), His (atom types 25, 27, 28 in Figure 5), Phe (atom types 9, 21 in Figure 5), and side chain hydrogen bond donors from Arg, Asn, Gln (atom types 15∼17 in Figure 5).

Table 2 shows the optimized prediction results for carbohydrate binding sites in proteins with carbohydrate ligands in the complex structures, and hence the prediction accuracy could only reflect a post-facto observation. In order to demonstrate the prediction capabilities on protein structures of unknown carbohydrate binding sites, it is essential to benchmark the prediction accuracy on a set of unbound protein structures, for which the structures were determined in the absence of the carbohydrate ligands while the carbohydrate bind sites are known from the complex structures of the same protein co-crystallized with the carbohydrate ligand. Subsets of unbound proteins from S497 and S108 were established as S88 and S23 respectively and were used to benchmark the accuracies of the predictors. Detailed benchmarks for each of the proteins in S88 and S23 are shown in Table S6 and Table S7 respectively. The prediction accuracies are compared for the bound and the unbound structures in Table 3. In general, the carbohydrate binding site predictions for the unbound structures are comparable to the bound structure, although the predictions for the unbound structures are slightly less accurate than the predictions for the bound structures. Results in Table 4 demonstrate that the proteins in S23 with very low sequence identities (<30%) to the proteins in the training set (S497) were able to be predicted with comparable accuracy to those of the cross-validations shown in Table 2, indicating that the optimized predictors shown in Table 2 are generalizable to unbound proteins previously unseen. Interactive examination of the prediction results for each of the S88 and S23 proteins can be accessed from the web server: http://ismblab.genomics.sinica.edu.tw/> benchmark > protein-carbohydrate.

**Table 3:** Prediction accuracies for the unbound and bound proteins.

Dataset/method	UnboundPre	UnboundSen	Unbound MCC	Bound Pre	Bound Sen	Bound MCC
S88/ANN	0.49	0.54	0.48	0.53	0.57	0.52
S88/SVM	0.51	0.51	0.48	0.57	0.55	0.53
S88/ANN_BAGGING	0.55	0.49	0.49	0.54	0.58	0.53
S23/ANN_BAGGING	0.50	0.53	0.49	0.53	0.65	0.56

The prediction accuracies are compared side-by-side for the unbound and bound proteins in S88 and in S23. The accuracy benchmarks and prediction algorithms are described above.

**Table 4:** Prediction results for proteins with sequence identity less than 30%.

PDB-code	Acc	Pre	Sen	Spe	MCC	Fsc	TP	TN	FP	FN	ID%
2UVE	0.94	0.34	0.93	0.94	0.55	0.5	14	452	27	1	16.3
2WSU	0.94	0.33	0.78	0.95	0.49	0.47	7	261	14	2	17.2
2XHH	0.94	0.50	0.29	0.98	0.35	0.36	2	100	2	5	17.9
1NOF	0.97	0.63	0.71	0.98	0.65	0.68	10	317	6	4	19.3
3ACF	0.94	0.40	0.44	0.96	0.39	0.42	4	161	6	5	22.7
3M9W	0.94	0.33	0.88	0.94	0.52	0.48	7	231	14	1	22.7
2X2S	0.93	0.40	0.86	0.93	0.56	0.55	6	122	9	1	22.9
1R13	0.96	0.63	0.71	0.98	0.65	0.68	5	126	3	2	23.7
3NSM	0.97	0.29	0.50	0.98	0.37	0.37	5	491	12	5	25.2
1M71	0.95	0.00	0.00	0.99	−0.02	0.00	0	196	2	8	25.6
2XHN	0.96	0.83	0.38	1.00	0.54	0.51	9	439	2	15	25.8
3K01	0.96	0.93	0.50	1.00	0.67	0.65	13	327	1	13	26.9
1MSB	0.95	1.00	0.38	1.00	0.60	0.55	3	98	0	5	29.6
**overall**	0.95	0.46	0.56	0.97	0.49	0.51	85	3321	98	67	

The prediction results for each of the proteins in S23 with maximum sequence identity less than 30% to any proteins in S497 are listed in this Table. The columns are defined as in Table 5. The ID% column shows the maximum sequence identity to any proteins in S497.

**Figure 6:**
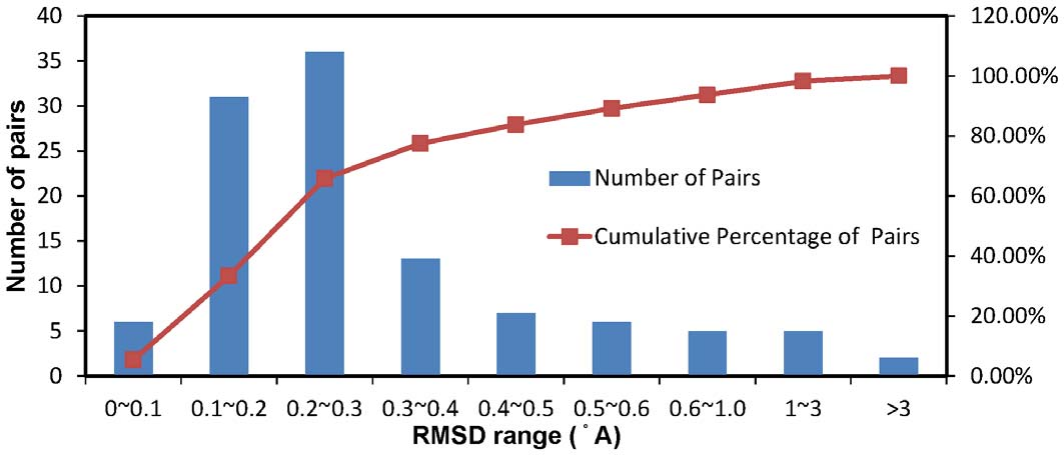
Distribution of the RMSDs of the bound-unbound protein pairs for proteins in S88 and S23. The details for the RMSDs are shown in Table S8.

The comparable accuracies for the predictions of bound and unbound protein structures as shown in Table 3 are resulted from the fact that protein-carbohydrate interactions do not change (due to, for example, induced fit) the unbound structures in most of the cases. As shown in Figure 6, 77.5% of the RMSDs for the unbound-bound structure pairs are less than 0.4 Å, although large conformational change after the ligand binding also occurs in a few cases (Table S8). But there is little correlation between the RMSD and the MCC for carbohydrate binding site prediction (correlation coefficient = −0.16).

### Carbohydrate binding sites on anti-carbohydrate antibodies

**Figure 7:**
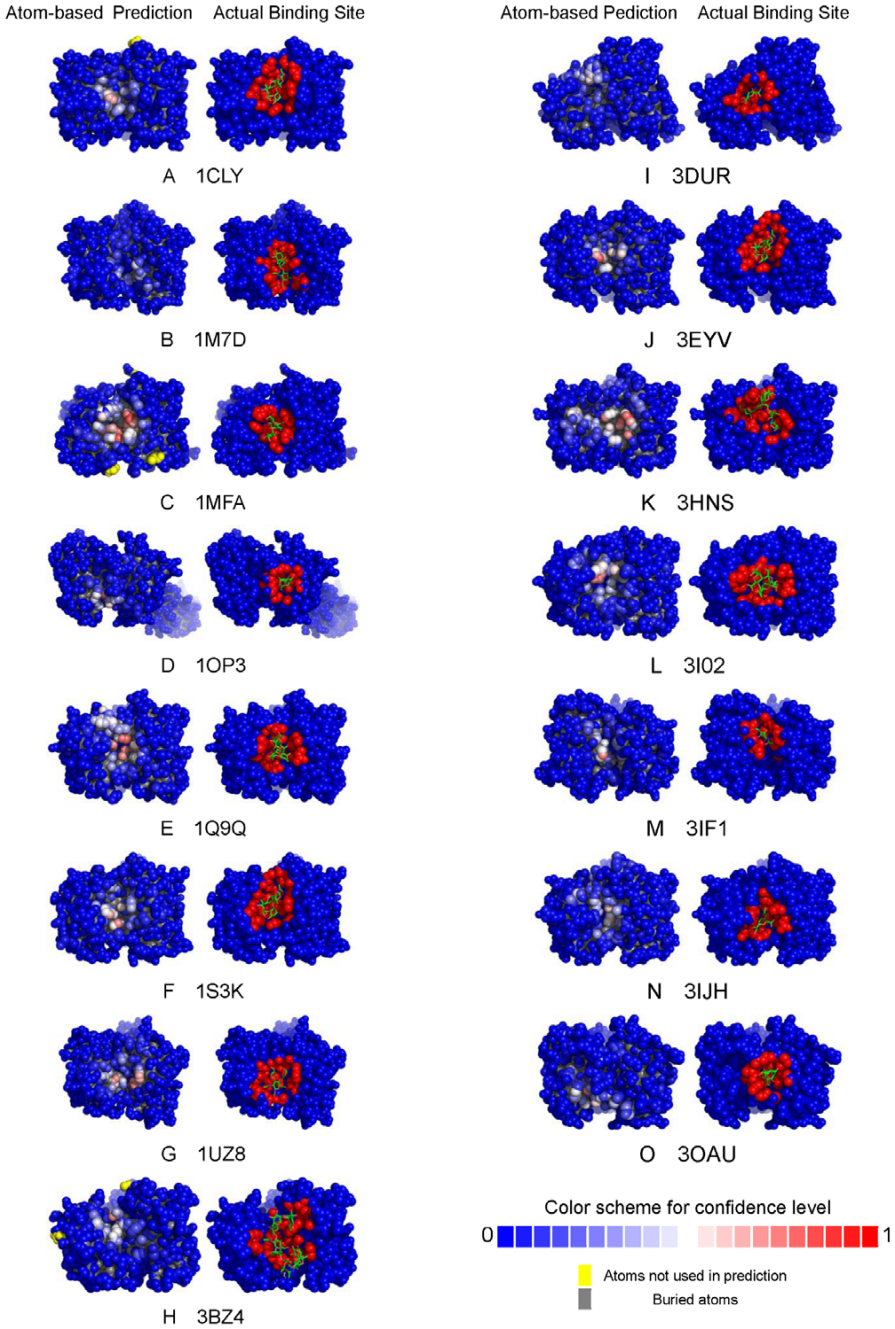
Carbohydrate binding site predictions for the anti-carbohydrate antibodies in PDB. Non-redundant anti-carbohydrate antibodies from PDB before June of 2011 were predicted with the SVM predictors trained with the S497 dataset, where none of these antibodies were included. The predicted atom-based carbohydrate binding patches and the actual binding sites with carbohydrate antigens (stick models) are compared side-by-side in each of the independent test cases shown in the panels. The prediction confidence level is color-coded as described in Figure 4; the actual binding sites are highlighted by the atoms colored in red to indicate that these atoms are within 5 Å to any atoms of the carbohydrate antigen. Table 5 shows the prediction accuracy benchmarks. The antibody models showing the carbohydrate binding site predictions are available for interactive examination from the web webserver http://ismblab.genomics.sinica.edu.tw/> benchmark > protein-carbohydrate.

Carbohydrate binding sites on the antibody variable domain structures from 15 non-redundant antibody-carbohydrate complexes from PDB (protein data bank) were predicted with the SVM predictors. These antibody-carbohydrate complexes had not been used for the training of the predictors, and thus are additional independent cases in testing the predictors. Figure 7 shows the results of the predicted carbohydrate binding patches on the antibodies. Table 5 summarizes the accuracy benchmarks for the binding site predictions. The antigen binding sites for the antibodies were predicted with reasonable accuracy with the average MCC of 0.49, which is close to the average performance for the predictions on proteins in the datasets of S497 and S108 (Table 2).

**Table 5:** Prediction results for the carbohydrate binding sites on anti-carbohydrate antibodies in PDB.

PDB-code	Acc	Pre	Sen	Spe	MCC	Fsc	TP	TN	FP	FN
1CLY	0.98	1.00	0.33	1.00	0.57	0.50	5	386	0	10
1M7D	0.96	0.83	0.25	1.00	0.44	0.39	5	384	1	15
1MFA	0.97	0.61	1.00	0.97	0.77	0.76	11	191	7	0
1OP3	0.96	0.33	0.15	0.99	0.21	0.21	2	392	4	11
1Q9Q	0.98	0.64	0.69	0.99	0.66	0.67	9	383	5	4
1S3K	0.98	0.78	0.47	1.00	0.59	0.58	7	376	2	8
1UZ8	0.97	0.73	0.50	0.99	0.59	0.59	8	379	3	8
3BZ4	0.95	0.43	0.18	0.99	0.26	0.25	3	370	4	14
3DUR	0.93	0.43	0.23	0.98	0.28	0.30	3	193	4	10
3EYV	0.97	0.78	0.44	1.00	0.57	0.56	7	389	2	9
3HNS	0.97	0.67	0.59	0.99	0.61	0.63	10	382	5	7
3I02	0.97	0.63	0.36	0.99	0.46	0.46	5	384	3	9
3IF1	0.99	1.00	0.58	1.00	0.76	0.74	7	383	0	5
3IJH	0.97	0.50	0.43	0.98	0.45	0.46	6	381	6	8
3OAU	0.96	0.22	0.15	0.98	0.16	0.18	2	379	7	11
**overall**	0.97	0.63	0.41	0.99	0.49	0.50	90	5352	53	129

Matthews correlation coefficient (MCC), F-score(Fsc), Accuracy(Acc), Precision(Pre), Sensitivity(Sen) and Specificity(Spe) are shown in Equations (4)∼(9). TP, FP, TN, and FN are true positive, false positive, true negative, and false negative respectively.

## Discussion

The optimized predictors, trained and cross-validated with S497 set, were evaluated with four independent test sets to benchmark the generalizability of the predictors. (1) Test on new proteins: Results from the test set S108, which contains new protein-carbohydrate complexes released after S497 set and shares at the most 50% sequence identity with any of the proteins in S497, are summarized in Table 2 and Table S5, demonstrating the generalizability of the predictors to new proteins not used in optimizing the predictors. (2) Test on truly unbound proteins: Unlike S497 and S108 proteins, proteins in S88 are carbohydrate-binding proteins without the co-crystalized ligands in the structures. The test results shown in Table 3 and in Table S6 suggest that the optimized predictors are generalizable to unbound structures. (3) Test on both new and truly unbound proteins: Proteins in S23 are unbound carbohydrate-binding proteins and share at the most 50% sequence identity to any of the S497 proteins. The benchmarks for S23 shown in Table 3 and Table S7 indicate that the predictors are generalizable to new and unbound structures. (4) Test on a totally different class of carbohydrate-binding proteins: Antibodies binding to carbohydrate antigens were tested for carbohydrate binding site predictions (Table 5 and Figure 7). Taken together, these test results, with MCC ranging from 0.45 to 0.49, demonstrate reasonable generalizability for the predictors to predict carbohydrate binding sites on new unbound protein structures unseen previously.

The prediction accuracy of the predictors herein depends on two main factors: the binding site geometry and the amino acid composition. Protein-carbohydrate binding sites are geometrically diverse. Carbohydrate processing enzymes and carbohydrate transporting proteins frequently bind to carbohydrates with deep crevices, while on the other extreme some sugar binding proteins interact with sugar moieties without substantial indentation on the protein surface. The carbohydrate binding sites that were predicted with high accuracy typically have deeply concave binding surfaces with aromatic side chains packing against the hydrophobic patches of the sugar rings and with hydrogen bonding acceptors and donors surrounding the hydrophilic groups on the ligand. These proteins are frequently enzymes related to carbohydrate processing, mostly carbohydrate hydrolase and transferase. Some lectins and other sugar binding proteins with deeply concave binding sites were also frequently predicted with high accuracy. Figure 4 shows a few typical examples with decreasing prediction accuracy – as the binding site geometry becomes less concave, the prediction accuracy decreases in general. Figure 4 also shows a general trend of increasing difficulty in predicting multiple carbohydrate binding patches on one protein.

The contribution of the geometry attribute to the prediction capability is also evident in Figure 2D, where the prediction confidence levels are highly correlated with the geometry attribute with Pearson's correlation coefficient of 0.48. In addition, the attributes derived from carbohydrate ligands are main contributors to the prediction capability, as demonstrated in Figures 2A∼2D. Figure 2 also shows that patterns of PDMs derived from non-covalent interacting atoms from proteins also provide useful information in distinguishing carbohydrate binding surfaces from other protein surfaces. Among these protein atom types, PDMs derived from hydrophobic and aromatic carbons are more informative than those corresponding to hydrophilic atom types. This is likely due to the fact that the hydrophilic nature of the carbohydrate binding sites is similar to that of protein surfaces, while the hydrophobic/aromatic residues in the carbohydrate binding sites are distinguishable to an extent from protein surfaces not involving in carbohydrate binding. Taken together, the carbohydrate binding sites with concave geometry and with amino acid composition able to interact with hydrophobic carbons were able to be predicted with relatively high confidence.

The predictions failed in about 15% of the proteins in S497. These carbohydrate binding sites were predicted with MCC less or equal to 0 (Figure 3A and Tables S2, S3, S4). These failed predictions can be categorized into four types: First, the carbohydrate binding sites are deeply buried such that the bind sites are no longer accessible as surface patches. These binding sites were not predicted with the methodologies in this work. Second, the carbohydrate binding sites are flat or even convex surfaces, for which accurate prediction of a cluster of atoms marking the binding site is intrinsically difficult for the methodologies. Third, rare combinations of amino acid types and side chain conformations that had not been observed frequently in known carbohydrate binding sites were frequently predicted as false negatives. Fourth, it is difficult to distinguish between non-covalent binding and covalent linking of the sugar moieties to a few experimental structures where the key covalent linkage information is missing in the structural data. These ‘binding sites’ were consistently failed to be predicted accurately.

The cluster of atoms with high confidence level in the predicted carbohydrate binding sites are mostly composed of side chain atoms from Trp (atom types 9, 18, 24, 30), Tyr (atom types 9, 19, 23), His (atom types 25, 27, 28), Phe (atom types 9, 21), side chain hydrogen bond donors from Arg, Asn, and Gln (atom types 15∼17), and to a lesser extent, from side chain hydrogen bond acceptors (atom types 10∼12) (Figure 5). These atom types are the key determinants in successfully predicting a carbohydrate binding site. These machine learning results are in good agreement with the atom types frequently observed to interact with carbohydrates in proteins [3]–[6]. The backbone hydrogen bond donors and acceptors are also frequently involved in protein-carbohydrate hydrogen bonding networks, but these atoms were less likely to be predicted with high confidence level (atom types, 2 and 4) (Figure 5). This is likely due to the fact that the large number of negative backbone atoms made the training data for the machine learning models extremely biased to the negative predictions.

Antibody-carbohydrate interactions are governed by the same set of energetic contributions as in protein-carbohydrate interactions. As demonstrated in the prediction results in Figure 7 and Table 5, the predictors trained with the S497 protein-carbohydrate interaction dataset were equally accurate in predicting carbohydrate binding sites on anti-carbohydrate antibodies. Except for the 2G12 antibodies (1OP3 and 3OAU), all the antigen binding sites on the antibodies in Table 5 are situated at the cavity surrounded by both of the variable domains. The geometrical shapes of the antigen binding sites lined with carbohydrate-binding residues (see previous paragraph) were clearly recognizable as carbohydrate-binding sites on the antibodies. The inaccuracy for the carbohydrate binding site predictions on 2G12 antibodies (MCC = 0.16 for 3OAU in Table 5) are partially due to the unconventional binding geometry of the carbohydrate binding sites and partially due to the lack of aromatic residues lining the carbohydrate binding surfaces. Nevertheless, the prediction results shown in Figure 7 and Table 5 demonstrate that the predictors developed in this work can be used to identify antibody structures targeting carbohydrate antigens. The residues highlighted with high prediction confidence level shown in Figure 7 are mostly aromatic residues (Trp, Tyr, and Phe) and to a lesser extent residues with hydrogen bonding donors and acceptors in the side chains. These residues were predicted to be key residues involving in the antibody-carbohydrate recognitions.

Result shown in Figure 5 is largely in good agreement with the U-test analysis shown in Figure 1. Except for the backbone atoms, the atom types with small p-values (colored in red in Figure 1) from the U-tests (i.e., distinguishable distribution patterns of the attributes between the binding and non-binding surfaces) were mostly predicted with higher accuracy in carbohydrate binding. This indicates that the U-test analysis is a useful tool in predicting the machine learning outcomes before the training of the machine learning models. These results strongly support the usage of U-tests as shown in Figure 1 in designing the attribute encoding for the machine learning algorithms. Nevertheless, the predictors for the main chain atoms (atom types 1∼4, p-values shown in Figure 1 were relative small for these atom types) were among the worst in prediction accuracy (Figure 2 and Figure 5), suggesting that small U-test p-value is necessary but not sufficient condition for accurate predictors. Other factors such as the side chain type and the secondary structure type that have not been included as input attributes could affect the prediction results for the main chain atoms.

The overall MCC for the prediction models were 0.45 for the proteins in the S108 test set (Table 2). The sensitivity and specificity are 0.49 and 0.97 respectively with the ANN_BAGGING algorithm (Table 2). In comparison, the prediction sensitivity and specificity from Malik and Ahmad [10] on 40 proteins with evolutionary information are 0.87 and 0.23 respectively, and the predictions with single sequences on the same set of proteins yield 0.68 and 0.55 respectively for sensitivity and specificity. Predictions of 29 glucose binding sites are more successful [12], with sensitivity of 0.8966 and specificity of 0.9333. But the prediction of monosaccharide binding sites on proteins is difficult to be compared with the prediction of carbohydrate binding sites involving multiple sugar moieties and diverse binding site geometries. MCC has been regarded as the most balanced benchmark for the two-class predictions as in this work, but none of the previous methodologies had been evaluated by the MCC benchmark. Hence it is difficult to compare the accuracies of the current predictors with the previous ones. Nevertheless, the accuracy of the current predictors has been vigorously validated with the largest dataset so far, and the results suggest that these predictors are among the best predictors for carbohydrate binding sites on proteins to date.

## Methods

### Three-dimensional probability density maps (PDMs) of non-covalent interacting atoms on protein surfaces

Construction probability density maps (PDMs) with protein non-covalent interacting atom pair database for 31 types (Table 1, atom types 1∼31) of non-covalent interacting atoms from amino acids and crystal water molecules of known protein structures has been described previously [14]. Details of the computational methodology are described in Text S1. The PDMs were constructed with interacting atoms within 5 Å of the surface atoms. Carbohydrate atoms were categorized into 5 atom types (Table 1, atom types 32∼36). The PDMs of the 5 types of carbohydrate atoms on protein surface were constructed with protein-carbohydrate interacting atom pair database derived from 3463 protein-carbohydrate complexes (from PDB version 2010, Jan 15). In order to keep the PDMs high in information content and low in noise from irrelevant interactions, non-interacting atomic pairs were eliminated with a filter system based on the work by McConkey et al. [15] (Table S1). An example of a set of 36 PDMs on a protein are shown in Figure S1. Interactive 3-D graphic presentation of the PDMs can be viewed from the web-server http://ismblab.genomics.sinica.edu.tw/ > gallery.

As shown in Table 1, there are a total of 30 ‘protein atom types’ (see Table 1 for protein atom types 1–30), derived from the 20 natural amino acids in proteins. For ‘non-covalent interacting atom types’ or simply ‘interacting atom types’, these atom types are interacting non-covalently with ‘protein atom types’. These types can be either originated from proteins (types 1–30) or from carbohydrate ligands (types 32–36) and water (type 31).

### Data sets

The machine learning models were first trained and tested with a selected set of protein-carbohydrate complexes. These complexes were selected with the criterion that at least 25 protein atoms are within 5 Å to the carbohydrate ligand. Base on the criterion, a total of 497 non-redundant protein-carbohydrate complexes with pair-wise sequence identity less than 90% were selected from 3463 protein-carbohydrate complexes (from PDB before Jan/2010). The selected set of protein-carbohydrate complexes is referred as the S497 set (Tables S2, S3, and S4). An independent test set was selected from more recent PDB entries. 2261 carbohydrate complex structures released between Jan/2010 and Jun/2011 were collected from PDB with the same criterion as above. 108 carbohydrate binding proteins with pairwise sequence identity less than 50% to any of the proteins in the S497 set were selected to form the S108 set (Table S5) for independent test of the optimized predictors. Predictors trained and validated with the S497 and S108 sets were further tested with unbounded proteins, for which the structures were determined in the absence of the carbohydrate ligands, so as to validate the prediction capabilities in realistic situations where the protein-carbohydrate binding sites are unknown. 88 unbound structures with 100% sequence identity to one of the proteins in S497 were selected from PDB to form the S88 set. These protein structures, unlike the corresponding protein structures in the S497 set, were determined in the absence of the carbohydrate ligands. Similarly, 23 unbound structures with 100% sequence identity to one of the proteins in the S108 set were selected to form the S23 set.

### PDM-based attributes as inputs for machine learning algorithms

Protein atoms were categorized into 30 atom types, and machine learning models were trained for each of the atom types. The input attributes for the machine learning models were calculated from the PDMs on the protein surface. In order to prevent self-information used in the predictions, all the interacting atom information involving the query protein were eliminated from the corresponding PDMs, mimicking a blind test for the protein structure that is not in the known protein database.

For each atom *i* on the surface of the query protein (solvent accessible surface area of atom *i*>0), the PDM values associated with the grids within 5 Å radius centered at the atom were summed in Equation (1).
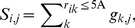
(1)where *S_i,j_* is the PDM sum for interacting atom type *j* at atom *i*; *r_ik_* is the distance between atom *i* to a grid point *k*; *g_k,j_* is the PDM value of interacting atom type *j* at grid point *k*.

*A_i,j_* (*j* = 1∼36) associated with each atom *i* was calculated with Equation (2).
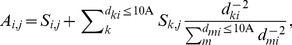
(2)where *S_i,j_* is defined in Equation (1); *d_ki_* is the distance between atom *i* and atom *k*.

The attribute set (*a_i,j_* (*j* = 1∼36)) for the machine learning models on atom *i* were derived from *A_i,j_* (*j* = 1∼36) with the following scaling scheme:

if *A_i,j_*>*M_max,j_* then *a_i,j_* = 1; otherwise

if *A_i,j_*<*M_min,j_* then *a_i,j_* = 0; otherwise
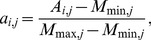
(3)where *M_max,j_* is the median of the distribution of the maximal *A_i,j_* from each of the proteins in S497 and *M_min,j_* is the median of the distribution of the minimal *A_i,j_* of the proteins in S497. Figure S2 shows the distributions of the maximal and minimal *A_i,j_* from the proteins in S497. The values of *M_min,j_* and *M_max,j_* are also listed in Figure S2. *a_i,j_* (*j* = 1∼36) are the first 36 attributes for machine learning; the 37^th^ attribute for the atom *i* was the fraction of the space not occupied by the van der Waals volume of the protein in the 10 Å sphere centered at the atom *i*. This attribute was also scaled between 0 and 1 with Equation (3) (see also Figure S2 for the distribution of *M_max,37_* and *M_min,37_*).

### Prediction of carbohydrate binding sites with three types of machine learning algorithm

Only the atoms on protein surfaces with SASA (solvent accessible surface area) greater than zero were used as training or testing cases. For each of the 30 atom types on proteins, machine learning models were trained and validated with the negative and positive cases found in the S497 and S108 sets. A positive case was a protein atom within 5 Å to any atoms of the carbohydrate ligand in the protein-carbohydrate complex. Protein atoms contacting aglycone components, such as peptides and lipids attaching to the sugar moieties, were not considered as positive cases. For unbound proteins in S88 and in S23, the positive cases were determined according the assignments in the corresponding protein-carbohydrate complexes in the S497 and S108 sets.

For each of the 30 atom types, the following three machine learning predictors were trained and cross-validated: Artificial neural network (ANN) – The details of the standard feed-forward back-propagation ANN [16] methodology has been described previously [13]. The input layer consisted of 37 nodes, for which the input attributes are described in Equations (1)∼(3). The hidden layers had 76 nodes, twice the sum of the input and the output. The output layer had a single node with the activity value between 0 and 1, matching the negative (0) and positive case (1) respectively. The learning rate for both the hidden layer and the output layer were 0.01; the momentum was 0.1. The training iteration was stopped as the mean absolute error between the ANN output values and the target values converged. The parameter set and the architecture of ANN were determined empirically for optimal performance.

Support vector machines (SVM) – The details of the standard SVM methodology has been described previously [13]. In brief, the SVM is a two-class classification approach with a maximized-margin hyperplane, where margin is the distance from the separating hyperplane to the closest data point [17] [18]. The cost (c) and gamma (γ) parameters of the SVM were optimized with grid searching for the optimal Matthews correlation coefficient (MCC) using only the training dataset.

For both the ANN and SVM training, all the positive cases and twice as many randomly selected negative cases from the training set were used. The selection of the ratio between the positive and the negative cases was optimized empirically.

Artificial neural network with bootstrap aggregation (ANN_BAGGING) – Bootstrap aggregation in machine learning was used to partially eliminate learning biases resulting from imbalanced training dataset, which is due to the fact that the negative atoms for carbohydrate binding in the training set greatly outnumber the positive atoms. The methodology [13] included multiple predictors to produce an ensemble of prediction results [19], and the final prediction was calculated by averaging with equal weight the output values from the predictors [20]. Each predictor of the ensemble was trained with a different sampling (bag) of the training set. In each bag, all of the positive cases in S497 were included, along with 1.5 times randomly sampled negative cases. The bag number was set to twenty, which balanced computational efficiency and prediction accuracy. Each of the bags was used to train an artificial neural network model. Here, a high speed resilient back-propagation (RPROP) training technique was used [21], [22]. Resilient propagation is capable of automatic adjustment for learning rate and momentum, with the advantage of faster convergence while requiring less manual determination of network parameters. Each of the ANN models was trained for 1000 iterations. During the training, the model was tested on validation set (see below) after every ten training iterations. The number of training iteration which yielded the best MCC (see below for MCC definition) on the validation set was used to determine the parameters of the predictors.

### 10-fold cross validation

All the machine learning algorithms above were trained and tested with 10-fold cross validations – 10 cross validations, each with 80% of the data from the S497 set as the training set; 10% of the data as the validation set; the remaining 10% of the data as the testing set.

For each of the predictors, a threshold for the output activity value was determined with the validation set; positive predictions have the output activity values greater than or equal to the threshold, while the negative predictions have the output activity values smaller than the threshold. All the thresholds were determined with the validation set to optimize the MCC for the predictions.

### Prediction accuracy benchmarks

The machining learning performance of the trained ANN, SVM and ANN_BAGGING models were benchmarked by accuracy (Acc), precision (Pre), sensitivity (Sen), specificity (Spe), F-score (Fsc) and Matthews correlation coefficient (MCC).
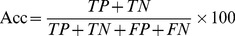
(4)

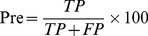
(5)

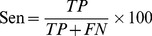
(6)

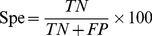
(7)

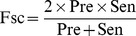
(8)

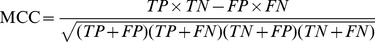
(9)where TP is the number of true positives; TN the number of true negatives; FP the number of false positives; and FN the number of false negatives. Sensitivity (also known as recall) can be viewed as a measurement of completeness, whereas precision is a measurement of exactness or fidelity. MCC, as a measurement of the quality of two class classifications (positive and negative), is generally regarded as a balanced measurement which can be used even if the classes are of very different sizes as in the carbohydrate-protein interactions. Its value ranges between −1 and 1; random correlation gives MCC of 0 while perfect correlation yields 1 in MCC.

### Confidence level for predictions

Prediction activity (output from ANN and ANN_BAGGING) or probability (output from SVM) with value ranging from 0 to 1 was normalized to prediction confidence level so that the normalized prediction results based on confidence level from various machine learning models for different protein atom types can be compared and integrated on level ground to form tentative carbohydrate binding patches, which invariably consist of various types of protein atoms. For each of the 30 protein atom types, the machine learning outputs from the validation sets were sorted into bins of interval 0.1. The confidence level of each of the bins was calculated as the fraction of true positive over the total number of predictions in the bin. In the end, lookup-tables for the output-confidence relationships were constructed; the machine learning outputs can be converted to prediction confidence levels with these lookup-tables. The look-up tables are charted in Figure S3.

### Prediction of patches of atoms as carbohydrate binding sites on proteins

Protein surface carbohydrate binding site is defined by a cluster of surface atoms (SASA>0) with high positive prediction confidence level. Protein surface atoms with confidence level for positive prediction greater than 50% were used as cluster centers to include neighboring surface atoms within radius of 5 Å. Within each of the surface patches, all the surface atoms with the confidence level for positive prediction greater than 10% were included in the tentative patch of atoms as carbohydrate binding site. If the pairwise distances of a subset of seeds were all within 10 Å, the subset patches were merged as one patch. The parameters were optimized for residue-based prediction accuracy on the validation set.

### Residue-based predictions for the carbohydrate binding sites

To facilitate comparison of this work with previous methods predicting carbohydrate binding sites at the residue level, a heuristic procedure was used to transform the atom-based binding site predictions as described in the previous paragraph into binding site predictions at the residue level: only the residues with more than 30% of the surface atoms (SASA>0) included in the atom-based binding patch were considered as positive residues of the residue-based patch. Similarly, actual carbohydrate binding sites at the residue level for proteins with known carbohydrate ligands were defined by patches of positive residues, each of which has more than 30% of the surface atoms (SASA>0 in the absence of carbohydrate ligand) on the residue within 5 Å to any atom of the carbohydrate ligand. This definition enabled the comparison of prediction results with actual binding sites at the residue level. The percentage parameter was optimized for residue-based prediction accuracy with the validation set.

### Mann-Whitney U-test

Mann-Whitney U-test is a non-parametric statistical method to test whether two groups of numerical values come from identical continuous distributions of equal medians – increasing p-value indicates decreasing difference of the two distributions and p-value of 1 indicates that the two distributions are statistically indistinguishable. The Mann-Whitney U-tests were carried out with the statistic tool *ranksum* in MATLAB (http://www.mathworks.com/help/toolbox/stats/ranksum.html).

### Web Site

Predictions can be submitted to the webserver http://ismblab.genomics.sinica.edu.tw/. All the benchmark results can also be accessed in interactive graphic presentations from the same web address above.

## Supporting Information

Figure S1 **Examples of PDMs around urtica dioica agglutinin (PDB code: 1EN2).** The contours are colored in blue, black, yellow and red to represent the probability density distributions of non-covalent interacting nitrogen, carbon, sulfur, and oxygen respectively. The protein molecule is shown as the solvent asscessible surface model; surface protein atoms are colored in red, blue, yellow, and white for oxygen, nitrogen, sulfur, and carbon respectively. The carbohydrate ligand is shown in stick model. Interactive 3-D graphic presentation of the PDMs can be viewed from the web server http://ismblab.genomics.sinica.edu.tw/ > gallery.
(DOC)

Figure S2 **Distributions of maximal and minial** ***A_i,j_*** **(** **Equation (2)** **in the main text) calculated from the proteins in S497.** *M_max,j_* shown in each of the panels is the median of the distribution of the maximal *A_i,j_* (distributions colored in blue) and *M_min,j_* is the median of the distribution of the minimal *A_i,j_* (distributions colored in red).
(DOC)

Figure S3 **Lookup charts converting output activity (probability) from the corresponding machine learning predictor to prediction confidence level.** For each of the 30 protein atom types, the machine learning outputs from the validation sets were sorted into bins of interval 0.1. The confidence level of each of the bins was calculated as the fraction of true positive over the total number of predictions in the bin. The panels (a), (b), and (c) are derived from ANN, SVM, and ANN_BAGGING predictions respectively. In each of the panel, two sets of curves are shown; one set for the prediction confidence level described as above (i.e., the positive prediction confidence); the other set for the negative prediction confidence. The sum of the positive prediction confidence level and the negative prediction confidence level equals to one.
(DOC)

Table S1 **A filter system used to eliminate non-interacting atomic pairs based on the work by McConkey et al with modifications.** The carbohydrate-protein interactions were added to the Table by following the principle that aliphatic carbons do not interact with polar oxygen and nitrogen atoms. During the construction of the PDMs, only the atom pairs with the matrix value less than −0.1 were included in the probability density maps. The detail descriptions of the protein/carbohydrate atom types are shown in Table 1 in the main text.
(DOC)

Table S2 **Ten-fold cross validation ANN prediction accuracy benchmarks on the S497 dataset.** The dataset, the ten-fold cross validation, and the benchmark measurements have been described in the main text. Matthews correlation coefficient (MCC), F-score(Fsc), Accuracy(Acc), Precision(Pre), Sensitivity(Sen) and Specificity(Spe) are shown in Equations (4)∼(9). TP, FP, TN, and FN are true positive, false positive, true negative, and false negative respectively. C1∼C7 represent carbohydrate binding sites in each of the test proteins; different protein has different number of binding sites. In these columns, the number of the predicted true positive atoms is shown over the actual number of atoms involving in the binding site. Interactive examination of the prediction results for each of the proteins in the S497 dataset can be accessed from the web server: http://ismblab.genomics.sinica.edu.tw/> benchmark > protein-carbohydrate.
(DOC)

Table S3 **Ten-fold cross validation SVM prediction accuracy benchmarks on the S497 dataset.** The dataset, the ten-fold cross validation, and the benchmark measurements have been described in the main text. Matthews correlation coefficient (MCC), F-score(Fsc), Accuracy(Acc), Precision(Pre), Sensitivity(Sen) and Specificity(Spe) are shown in Equations (4)∼(9). TP, FP, TN, and FN are true positive, false positive, true negative, and false negative respectively. C1∼C7 represent carbohydrate binding sites in each of the test proteins; different protein has different number of binding sites. In these columns, the number of the predicted true positive atoms is shown over the actual number of atoms involving in the binding site. Interactive examination of the prediction results for each of the proteins in the S497 dataset can be accessed from the web server: http://ismblab.genomics.sinica.edu.tw/> benchmark > protein-carbohydrate.
(DOC)

Table S4 **Ten-fold cross validation ANN_BAGGING prediction accuracy benchmarks on the S497 dataset.** The dataset, the ten-fold cross validation, and the benchmark measurements have been described in the main text. Matthews correlation coefficient (MCC), F-score(Fsc), Accuracy(Acc), Precision(Pre), Sensitivity(Sen) and Specificity(Spe) are shown in Equations (4)∼(9). TP, FP, TN, and FN are true positive, false positive, true negative, and false negative respectively. C1∼C7 represent carbohydrate binding sites in each of the test proteins; different protein has different number of binding sites. In these columns, the number of the predicted true positive atoms is shown over the actual number of atoms involving in the binding site. Interactive examination of the prediction results for each of the proteins in the S497 dataset can be accessed from the web server: http://ismblab.genomics.sinica.edu.tw/> benchmark > protein-carbohydrate.
(DOC)

Table S5 **ANN_BAGGING prediction accuracy benchmarks on the independent test set S108.** The dataset and the benchmark measurements have been described in the main text. Matthews correlation coefficient (MCC), F-score(Fsc), Accuracy(Acc), Precision(Pre), Sensitivity(Sen) and Specificity(Spe) are shown in Equations (4)∼(9). TP, FP, TN, and FN are true positive, false positive, true negative, and false negative respectively. C1∼C6 represent carbohydrate binding sites in each of the test proteins; different protein has different number of binding sites. In these columns, the number of the predicted true positive atoms is shown over the actual number of atoms involving in the binding site. The upper-limit of the pairwise sequence identity for each of the proteins in S108 to the homologues in S497 is shown in ID% column. Interactive examination of the prediction results for each of the proteins in the 108 independent test set can be accessed from the web server: http://ismblab.genomics.sinica.edu.tw/> benchmark > protein-carbohydrate.
(DOC)

Table S6 **ANN_BAGGING prediction accuracy benchmarks on the unbound set S88.** The dataset and the benchmark measurements have been described in the main text. Matthews correlation coefficient (MCC), F-score(Fsc), Accuracy(Acc), Precision(Pre), Sensitivity(Sen) and Specificity(Spe) are shown in Equations (4)∼(9). TP, FP, TN, and FN are true positive, false positive, true negative, and false negative respectively. Interactive examination of the prediction results for each of the proteins in the 88 unbound test set can be accessed from the web server: http://ismblab.genomics.sinica.edu.tw/> benchmark > protein-carbohydrate.
(DOC)

Table S7 **ANN_BAGGING prediction accuracy benchmarks on the unbound set S23.** The dataset and the benchmark measurements have been described in the main text. Matthews correlation coefficient (MCC), F-score(Fsc), Accuracy(Acc), Precision(Pre), Sensitivity(Sen) and Specificity(Spe) are shown in Equations (4)∼(9). TP, FP, TN, and FN are true positive, false positive, true negative, and false negative respectively. Interactive examination of the prediction results for each of the proteins in the 23 unbound test set can be accessed from the web server: http://ismblab.genomics.sinica.edu.tw/> benchmark > protein-carbohydrate.
(DOC)

Table S8 **Root mean square deviation (RMSD) between carbohydrate-bound and unbound structure.** The structural alignments were performed by PyMOL package. Interactive examination of the superimposed results for each pair of bound and unbound proteins can be accessed from the web server: http://ismblab.genomics.sinica.edu.tw/> benchmark > protein-carbohydrate.
(DOC)

Text S1 **Supplemental methods.**
(DOC)
