# Supplementary material for: Prediction of Carbohydrate Binding Sites on Protein Surfaces with 3-Dimensional Probability Density Distributions of Interacting Atoms
Source: PLoS One. 2012 Jul 25;7(7):e40846. doi: 10.1371/journal.pone.0040846 (PMC3405063; doi:10.1371/journal.pone.0040846)
Supplement: Figure S1 — Examples of PDMs around urtica dioica agglutinin (PDB code: 1EN2). The contours are colored in blue, black, yellow and red to represent the probability density distributions of non-covalent interacting nitrogen, carbon, sulfur, and oxygen respectively. The protein molecule is shown as the solvent asscessible surface model; surface protein atoms are colored in red, blue, yellow, and white for oxygen, nitrogen, sulfur, and carbon respectively. The carbohydrate ligand is shown in stick model. Interactive 3-D graphic presentation of the PDMs can be viewed from the web server http://ismblab.genomics.sinica.edu.tw/ > gallery. (DOC) [file pone.0040846.s001.doc]

**Figure S1**

| **Interacting atom type 1** | **Interacting atom type 2** |
| --- | --- |
| **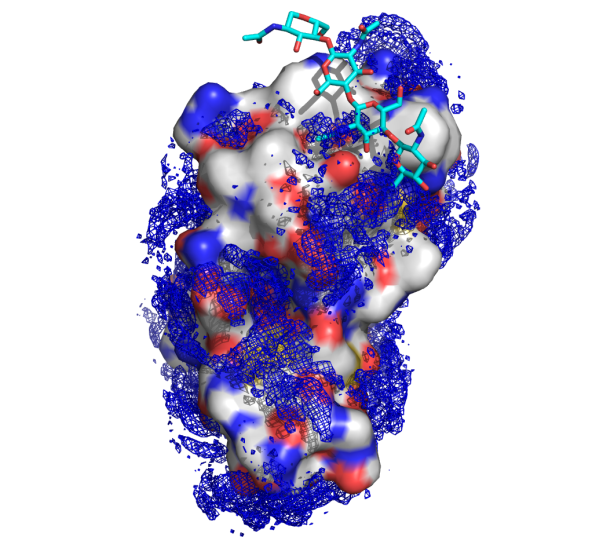**  **Contour threshold**  **= 0.0006** | **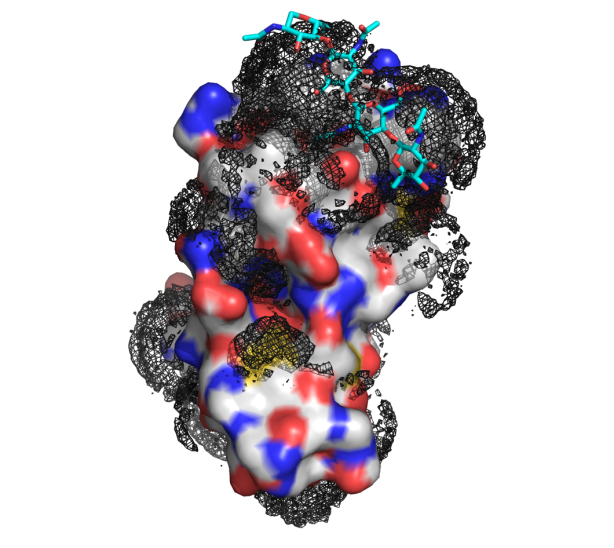**  **Contour threshold**  **= 0.0006** |
| **Interacting atom type 3** | **Interacting atom type 4** |
| **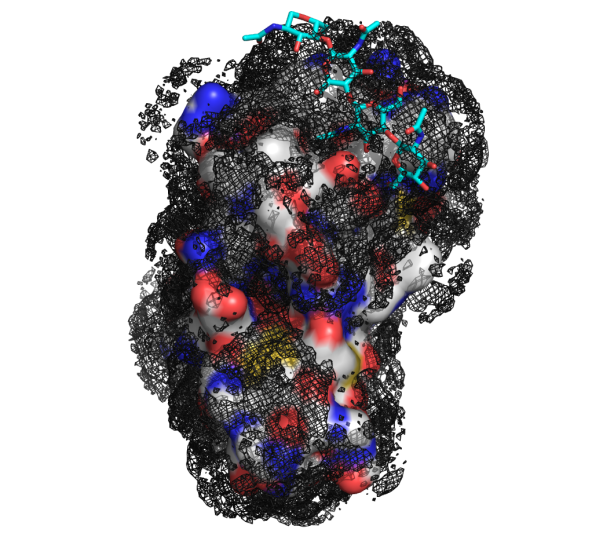**  **Contour threshold**  **= 0.0006** | **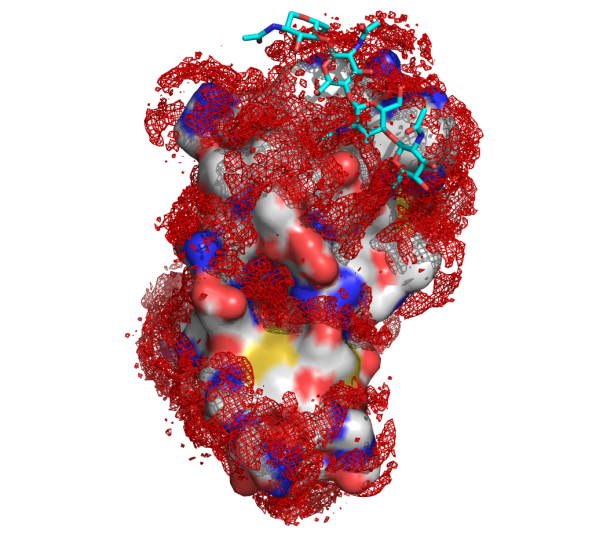**  **Contour threshold**  **= 0.0006** |
| **Interacting atom type 5** | **Interacting atom type 6** |
| **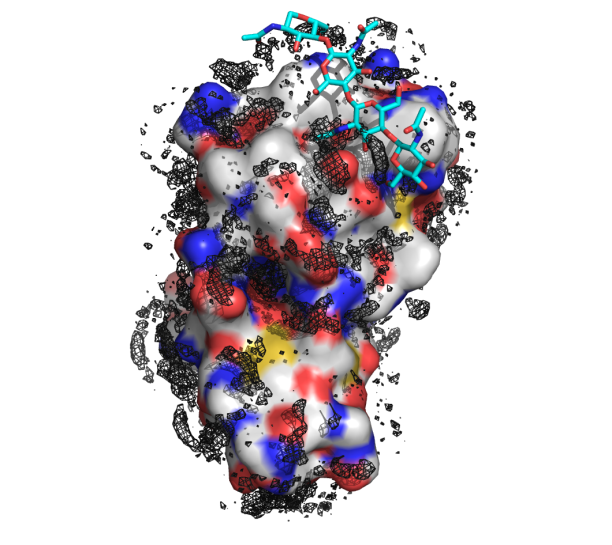**  **Contour threshold**  **= 0.0006** | **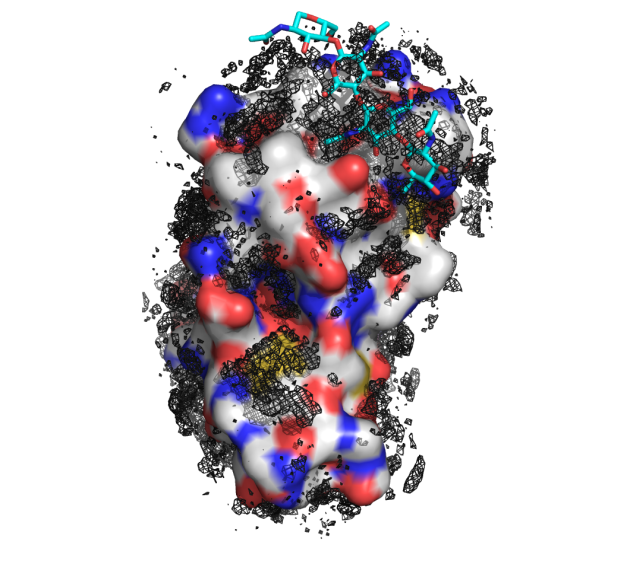**  **Contour threshold**  **= 0.0006** |

| **Interacting atom type 7** | **Interacting atom type 8** |
| --- | --- |
| **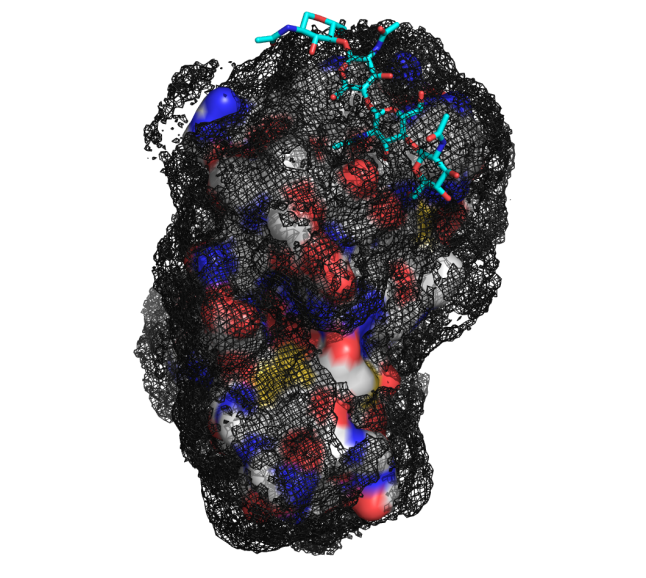**  **Contour threshold**  **= 0.0006** | **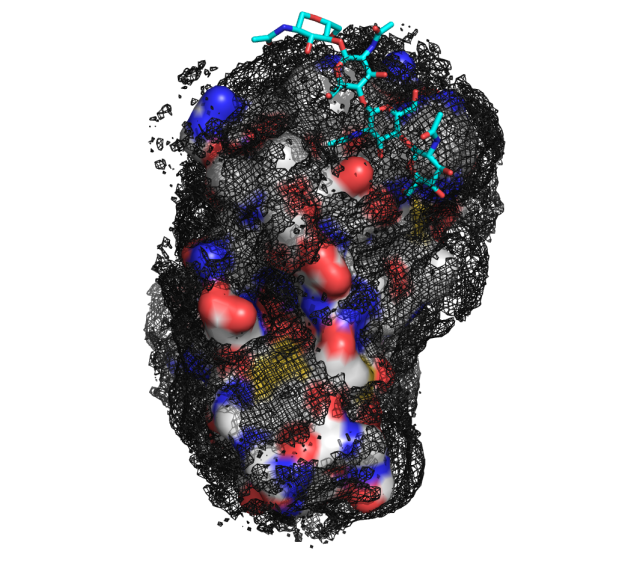**  **Contour threshold**  **= 0.0006** |
| **Interacting atom type 9** | **Interacting atom type 10** |
| **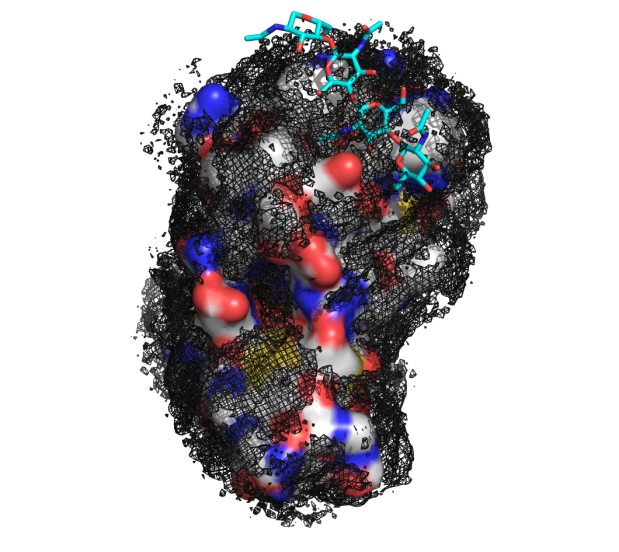**  **Contour threshold**  **= 0.0006** | **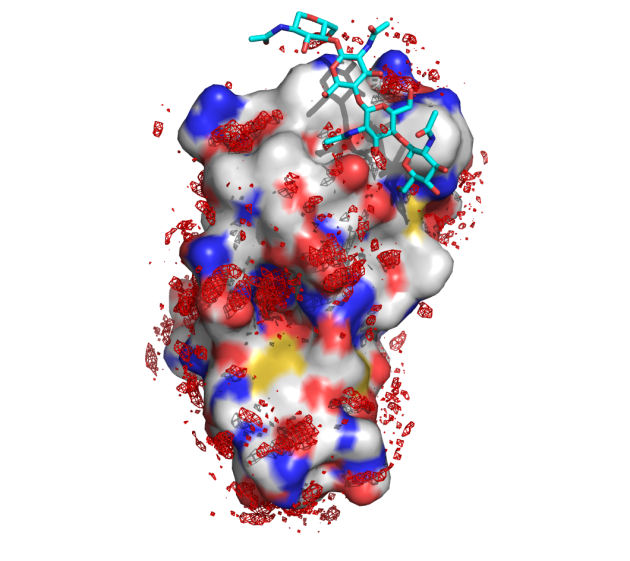**  **Contour threshold**  **= 0.0006** |
| **Interacting atom type 11** | **Interacting atom type 12** |
| **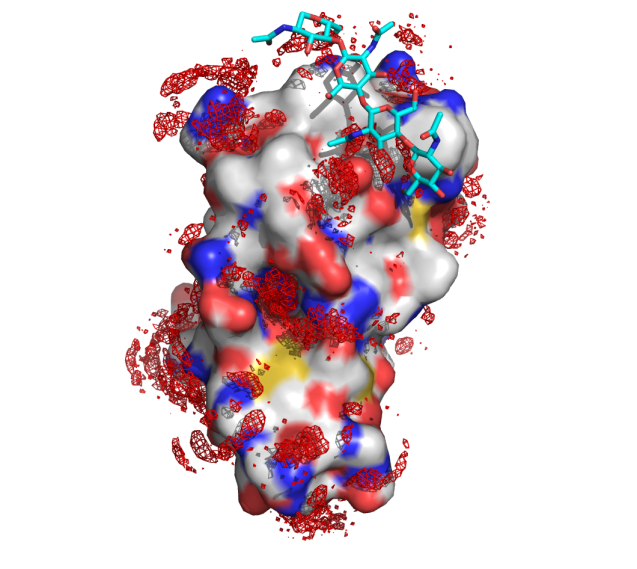**  **Contour threshold**  **= 0.0006** | **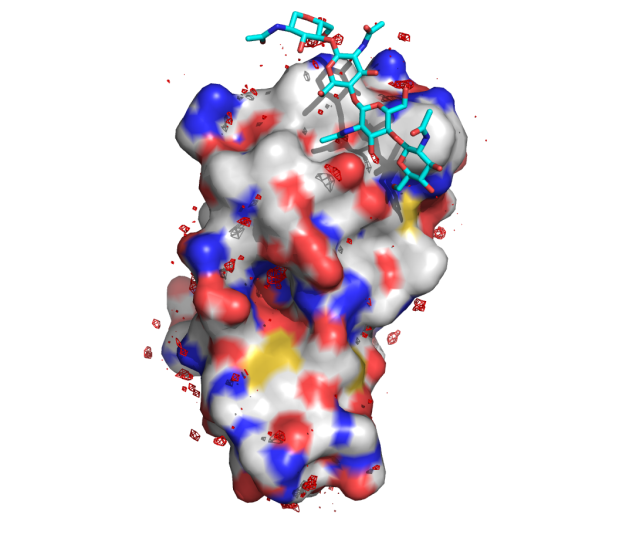**  **Contour threshold**  **= 0.0006** |

| **Interacting atom type 13** | **Interacting atom type 14** |
| --- | --- |
| **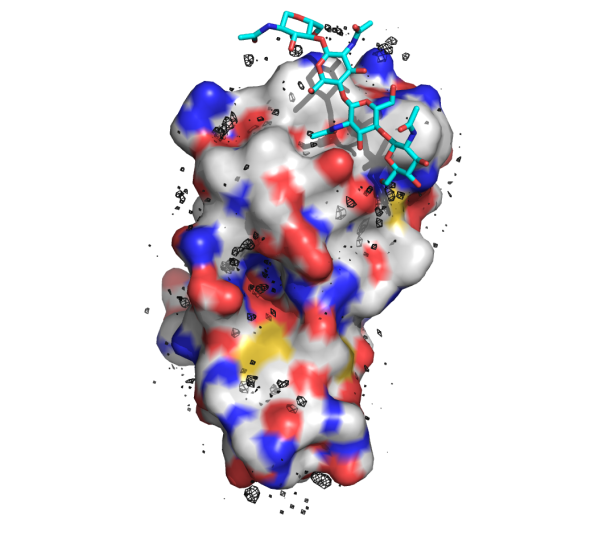**  **Contour threshold**  **= 0.0006** | **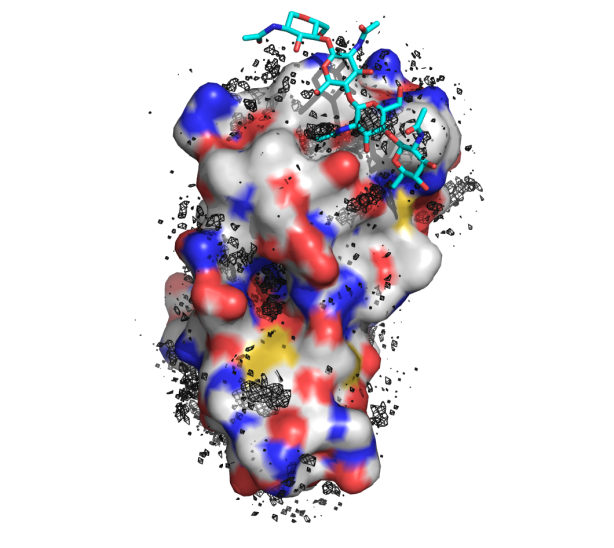**  **Contour threshold**  **= 0.0006** |
| **Interacting atom type 15** | **Interacting atom type 16** |
| **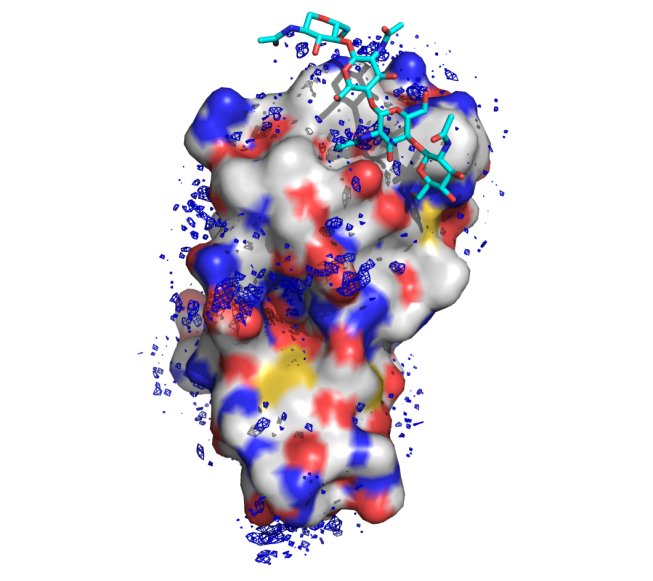**  **Contour threshold**  **= 0.0006** | **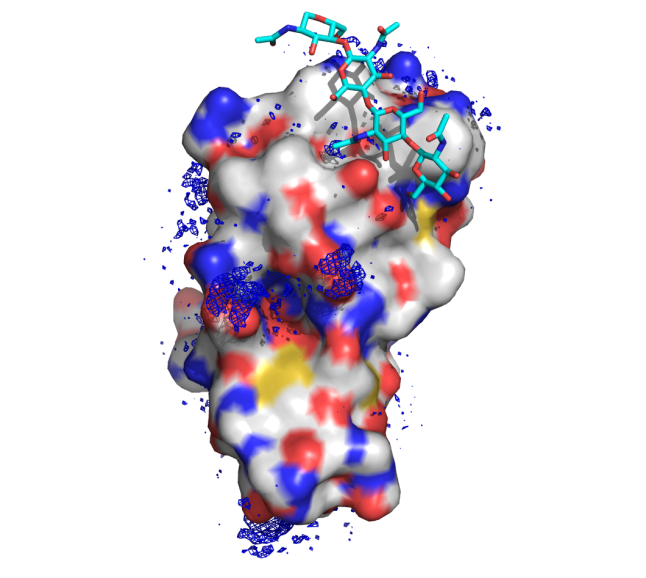**  **Contour threshold**  **= 0.0006** |
| **Interacting atom type 17** | **Interacting atom type 18** |
| **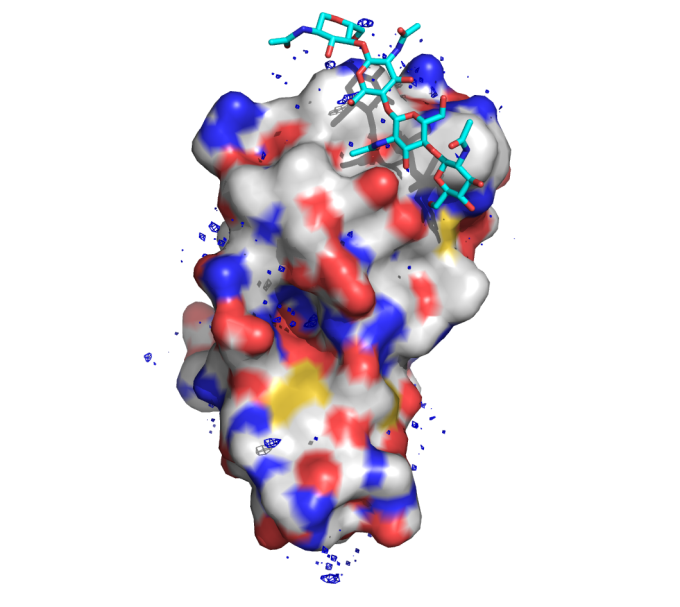**  **Contour threshold**  **= 0.0006** | **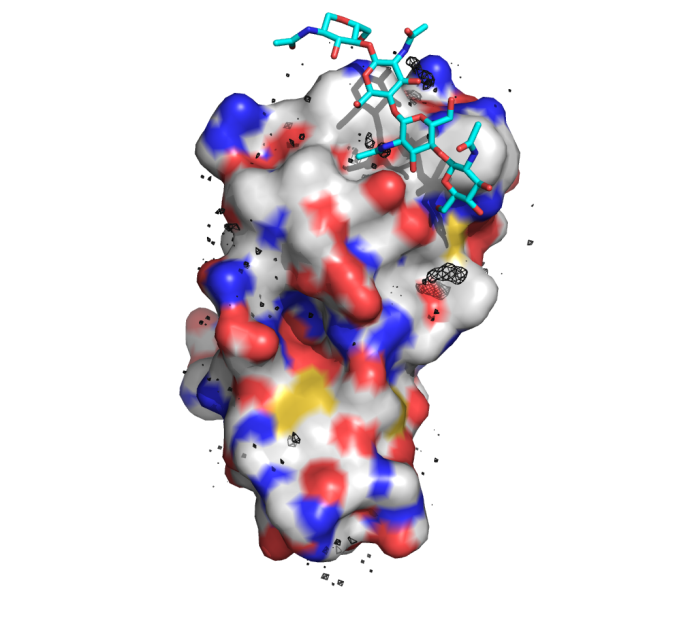**  **Contour threshold**  **= 0.0006** |

| **Interacting atom type 19** | **Interacting atom type 20** |
| --- | --- |
| **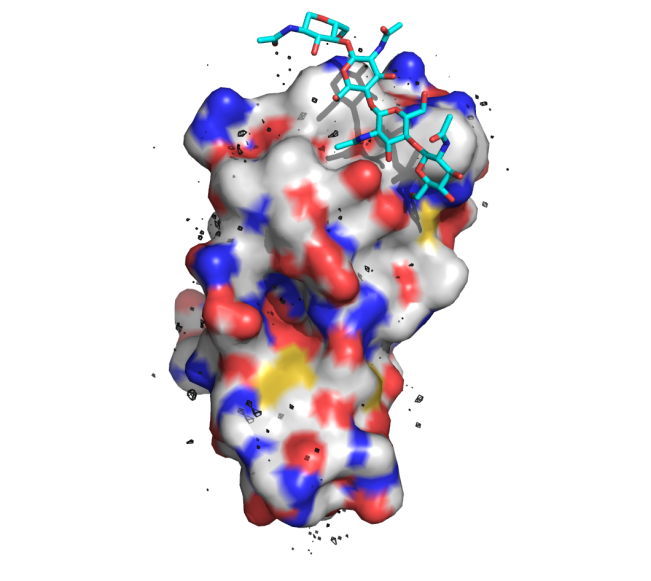**  **Contour threshold**  **= 0.0006** | **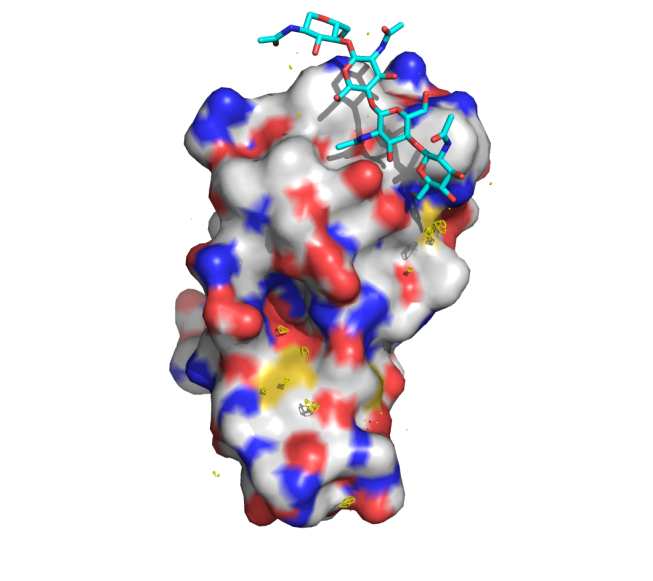**  **Contour threshold**  **= 0.0006** |
| **Interacting atom type 21** | **Interacting atom type 22** |
| **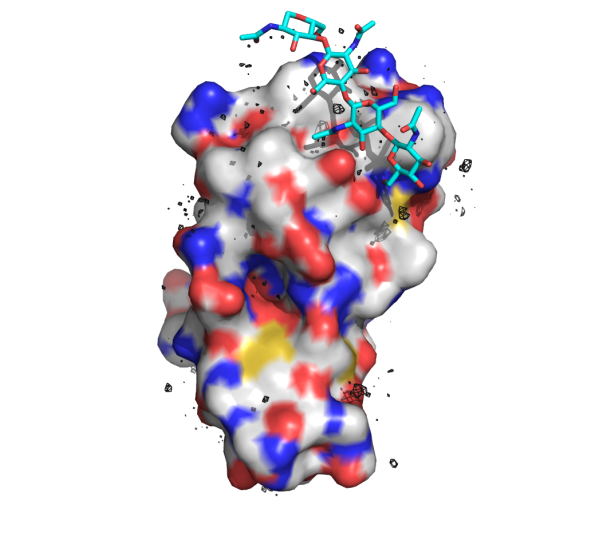**  **Contour threshold**  **= 0.0006** | **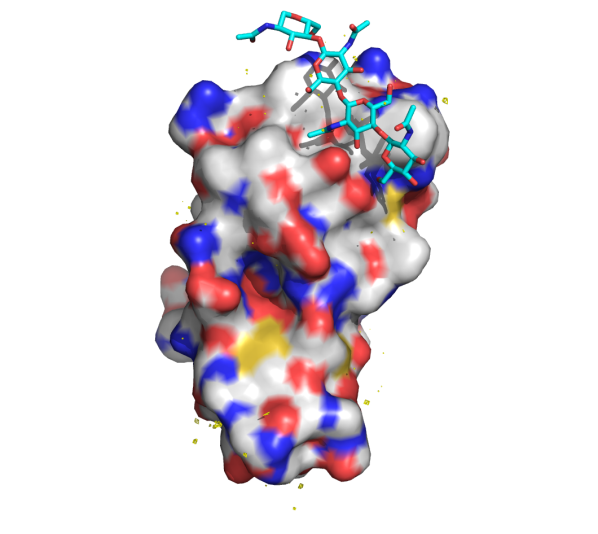**  **Contour threshold**  **= 0.0006** |
| **Interacting atom type 23** | **Interacting atom type 24** |
| **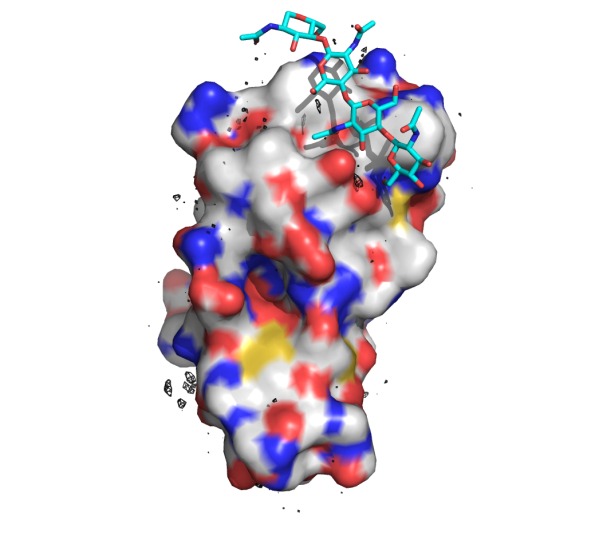**  **Contour threshold**  **= 0.0006** | **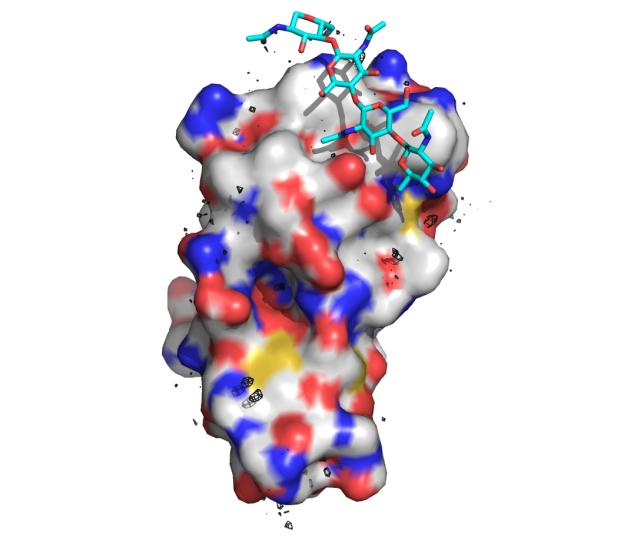**  **Contour threshold**  **= 0.0006** |

| **Interacting atom type 25** | **Interacting atom type 26** |
| --- | --- |
| **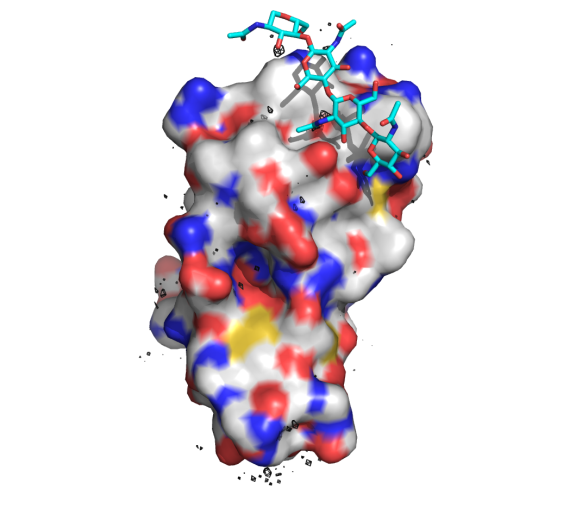**  **Contour threshold**  **= 0.0006** | **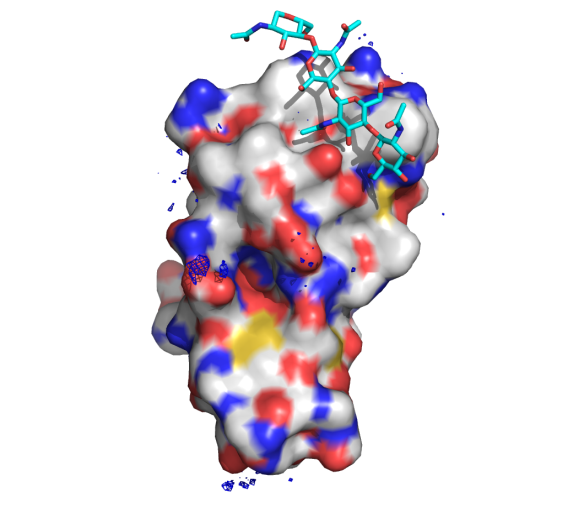**  **Contour threshold**  **= 0.0006** |
| **Interacting atom type 27** | **Interacting atom type 28** |
| **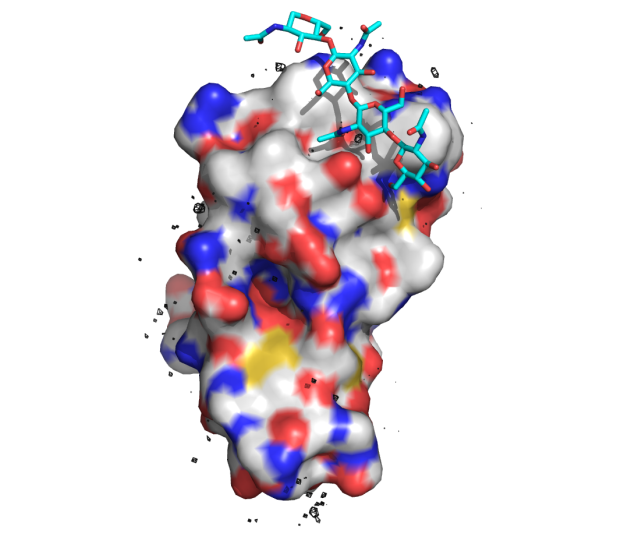**  **Contour threshold**  **= 0.0006** | **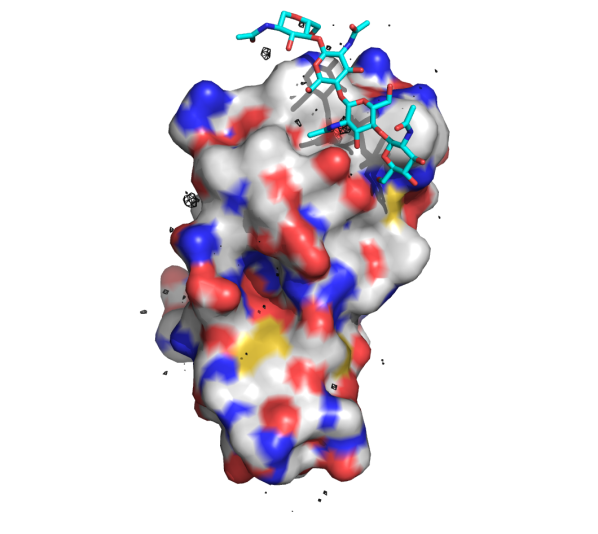**  **Contour threshold**  **= 0.0006** |
| **Interacting atom type 29** | **Interacting atom type 30** |
| **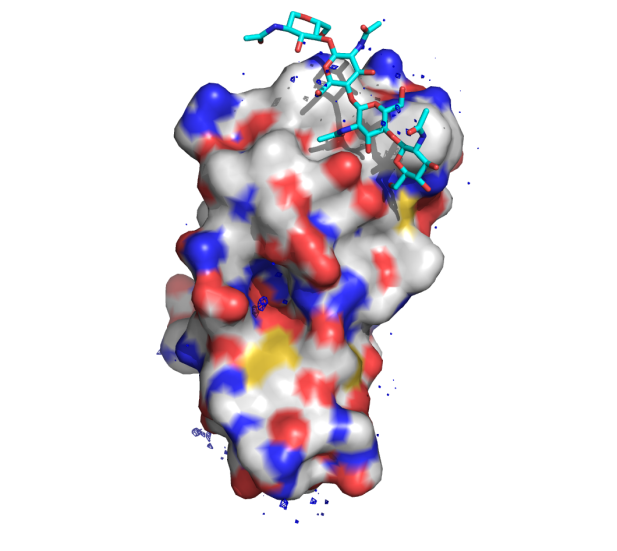**  **Contour threshold**  **= 0.0006** | **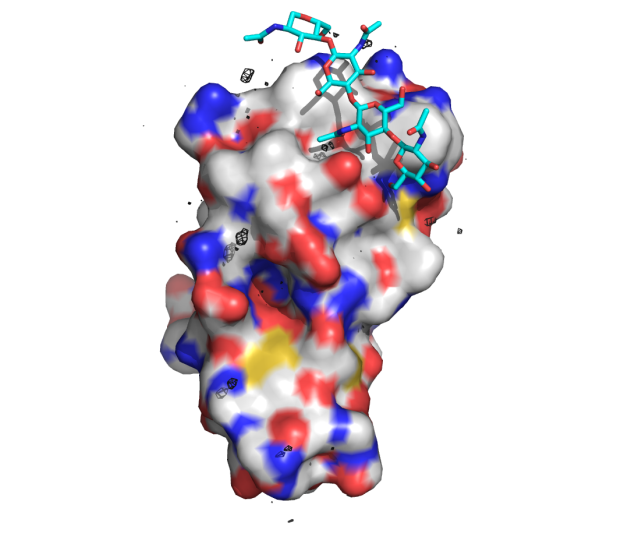**  **Contour threshold**  **= 0.0006** |

| **Interacting atom type 31** | **Interacting atom type 32** |
| --- | --- |
| **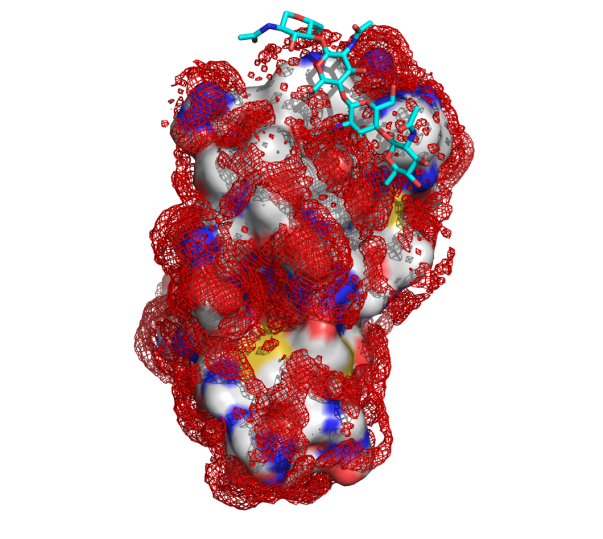**  **Contour threshold**  **= 0.0006** | **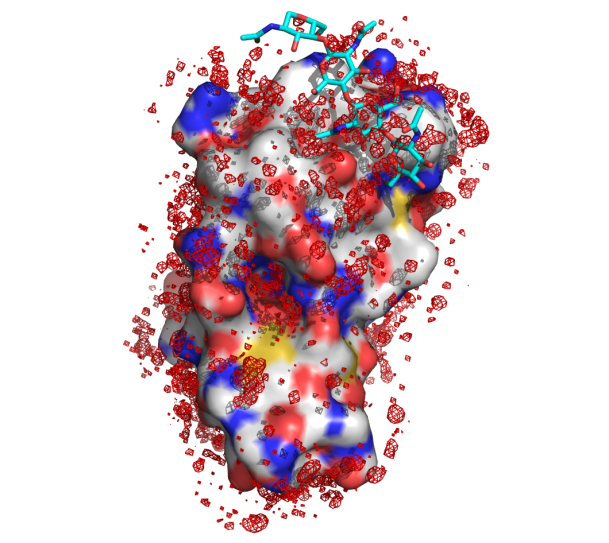**  **Contour threshold**  **= 0.0006** |
| **Interacting atom type 33** | **Interacting atom type 34** |
| **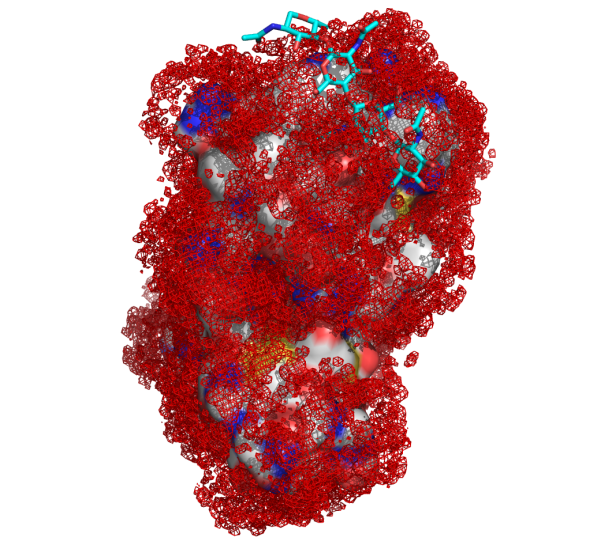**  **Contour threshold**  **= 0.0006** | **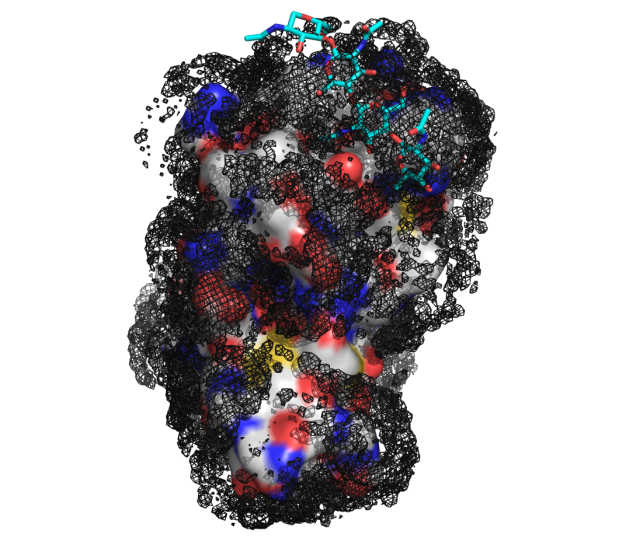**  **Contour threshold**  **= 0.0006** |
| **Interacting atom type 35** | **Interacting atom type 36** |
| **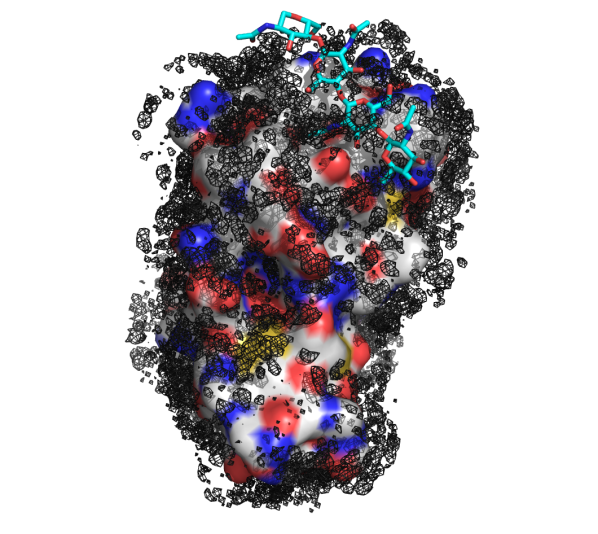**  **Contour threshold**  **= 0.0006** | **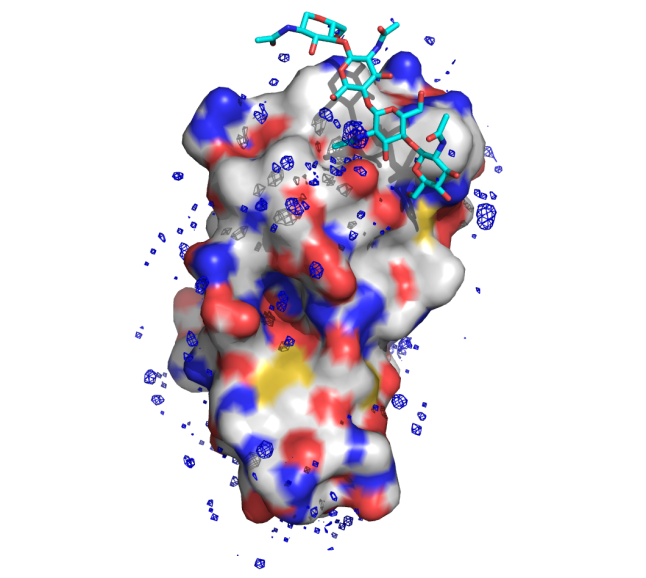**  **Contour threshold**  **= 0.0006** |

**Figure S1:** Examples of PDMs around urtica dioica agglutinin (PDB code: 1EN2). The contours are colored in blue, black, yellow and red to represent the probability density distributions of non-covalent interacting nitrogen, carbon, sulfur, and oxygen respectively. The protein molecule is shown as the solvent asscessible surface model; surface protein atoms are colored in red, blue, yellow, and white for oxygen, nitrogen, sulfur, and carbon respectively. The carbohydrate ligand is shown in stick model. Interactive 3-D graphic presentation of the PDMs can be viewed from the web server <http://ismblab.genomics.sinica.edu.tw/> > gallery.
