# Supplementary material for: Prediction of Carbohydrate Binding Sites on Protein Surfaces with 3-Dimensional Probability Density Distributions of Interacting Atoms
Source: PLoS One. 2012 Jul 25;7(7):e40846. doi: 10.1371/journal.pone.0040846 (PMC3405063; doi:10.1371/journal.pone.0040846)
Supplement: Figure S2 — Distributions of maximal and minial Ai,j ( Equation (2) in the main text) calculated from the proteins in S497. Mmax,j shown in each of the panels is the median of the distribution of the maximal Ai,j (distributions colored in blue) and Mmin,j is the median of the distribution of the minimal Ai,j (distributions colored in red). (DOC) [file pone.0040846.s002.doc]

**Figure S2**

**Interacting atom type** 1


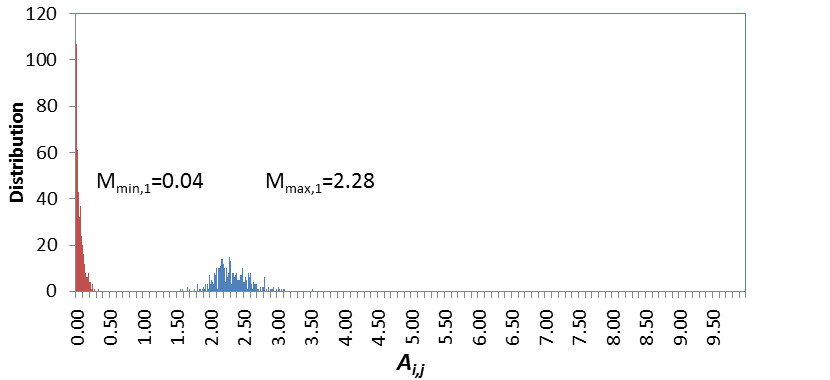


**Interacting atom type** 2


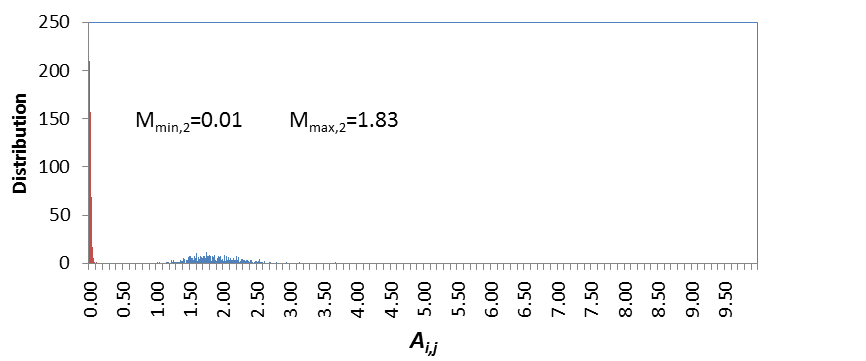


**Interacting atom type** 3


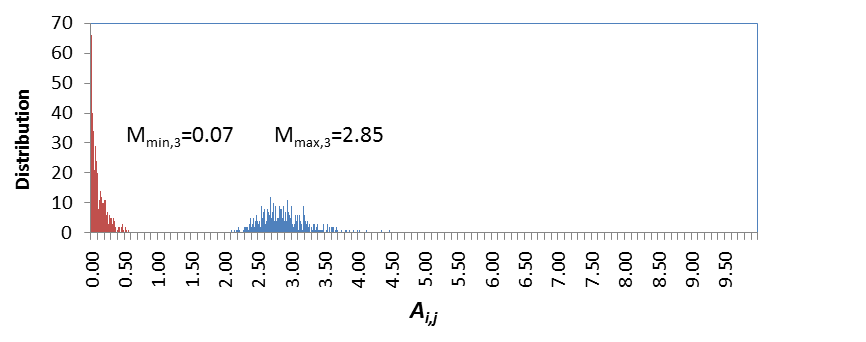


**Interacting atom type** 4


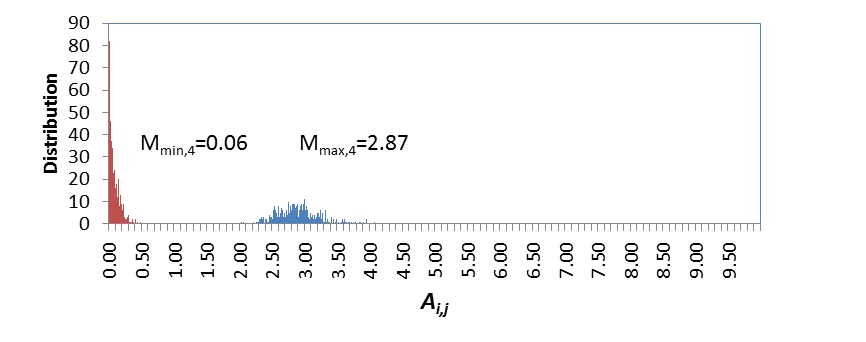


**Interacting atom type** 5


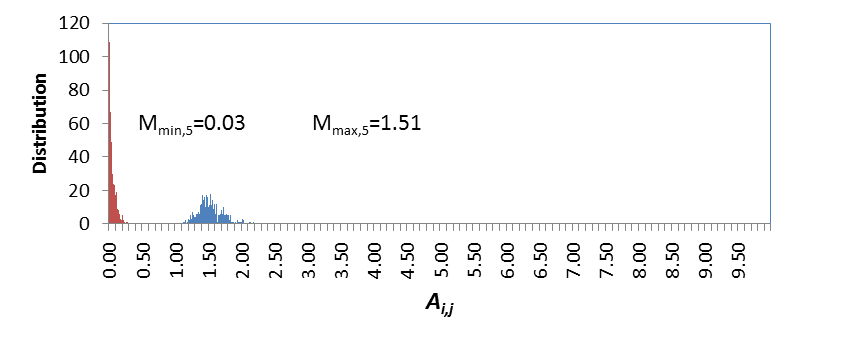


**Interacting atom type** 6


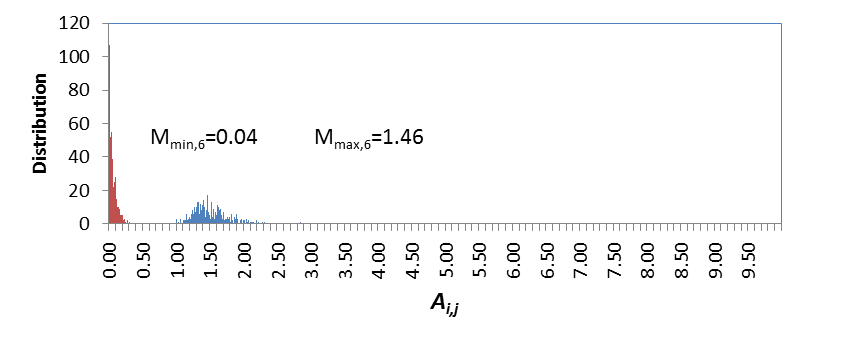


**Interacting atom type** 7


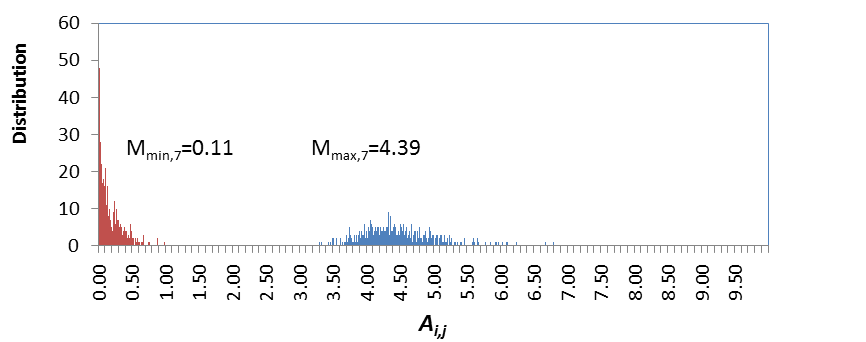


**Interacting atom type** 8


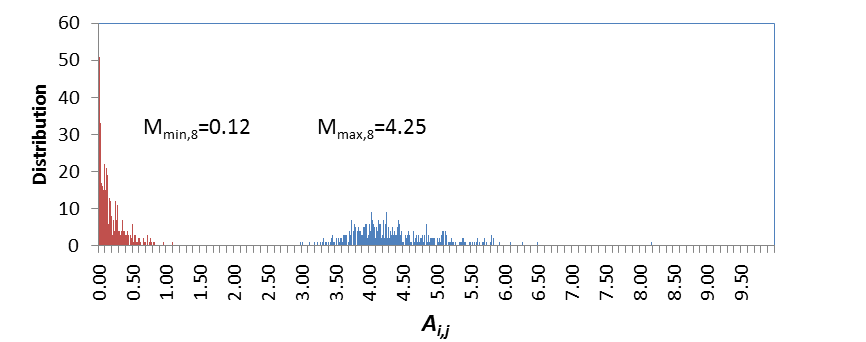


**Interacting atom type** 9


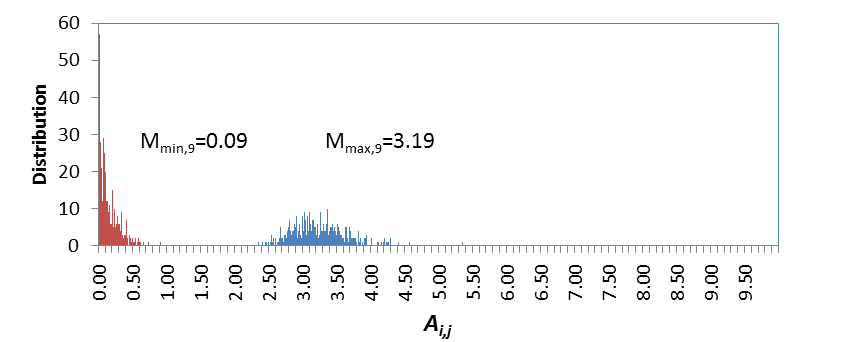


**Interacting atom type** 10


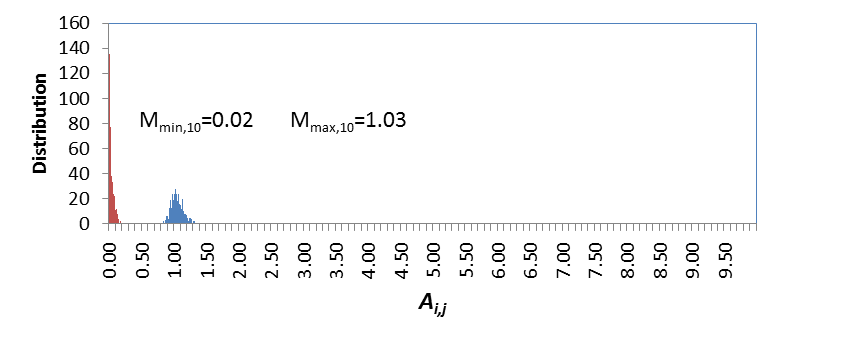


**Interacting atom type** 11


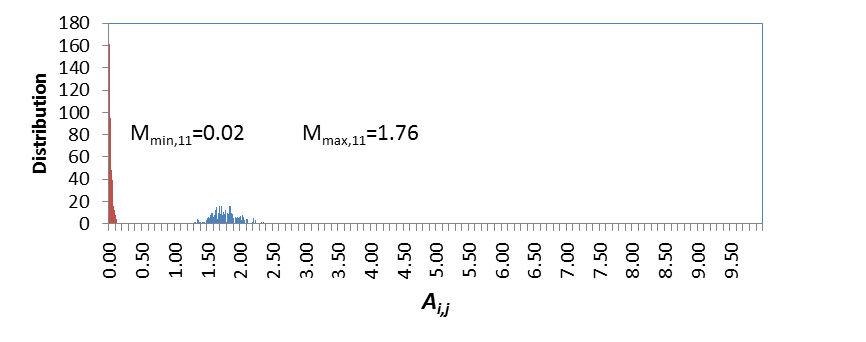


**Interacting atom type** 12


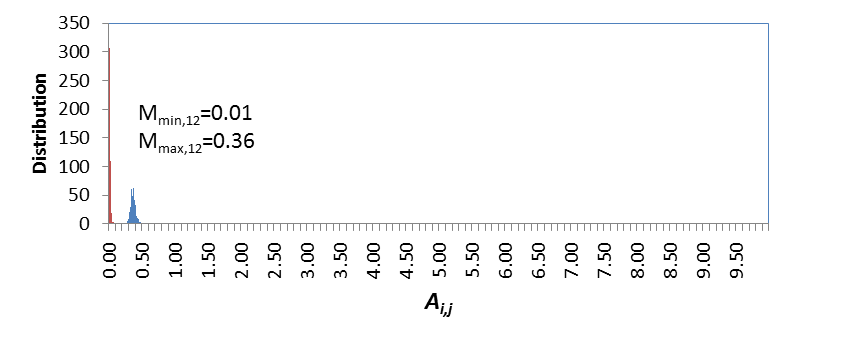


**Interacting atom type** 13


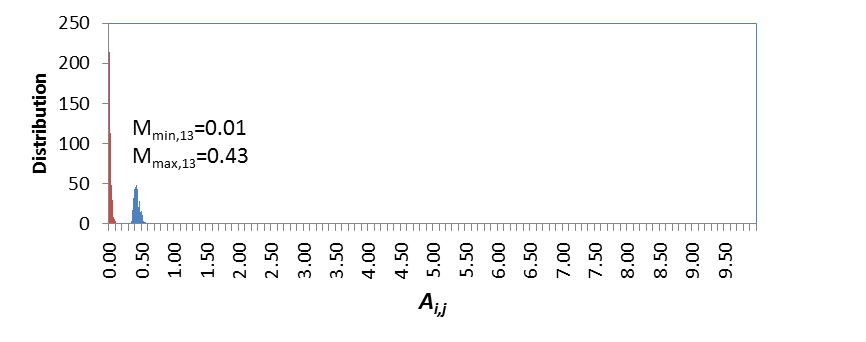


**Interacting atom type** 14


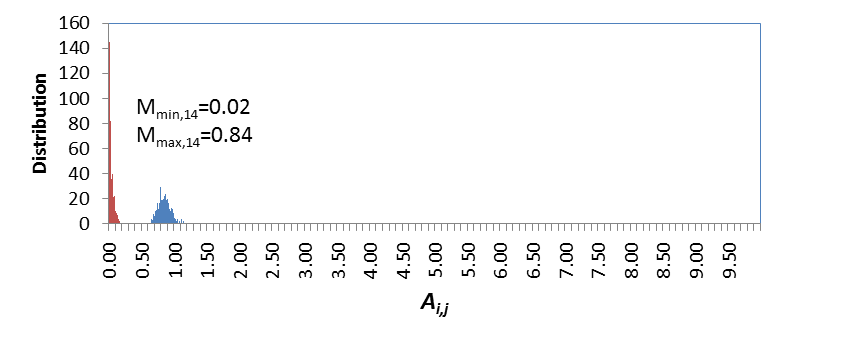


**Interacting atom type** 15


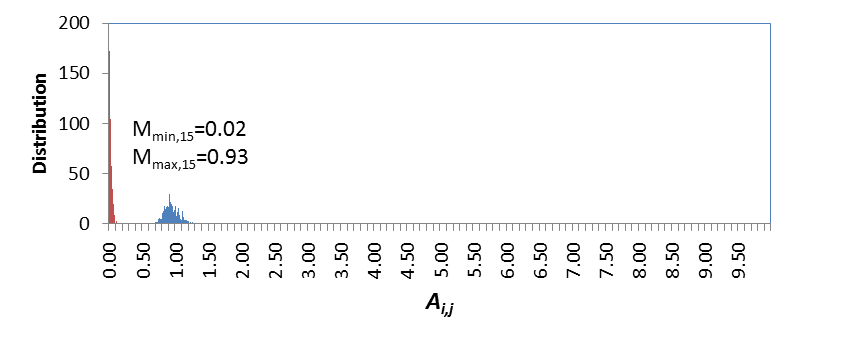


**Interacting atom type** 16


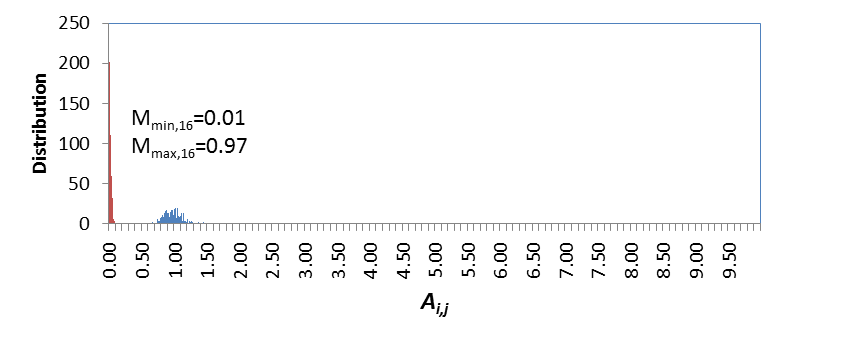


**Interacting atom type** 17


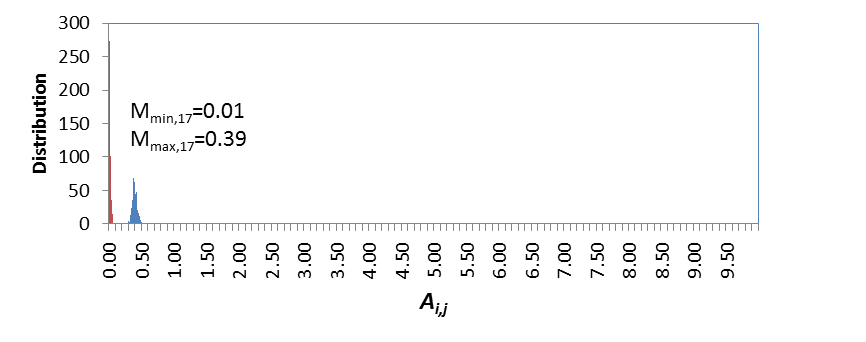


**Interacting atom type** 18


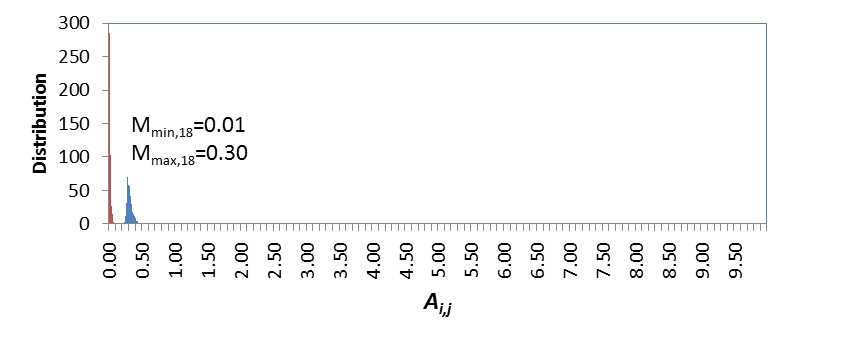


**Interacting atom type** 19


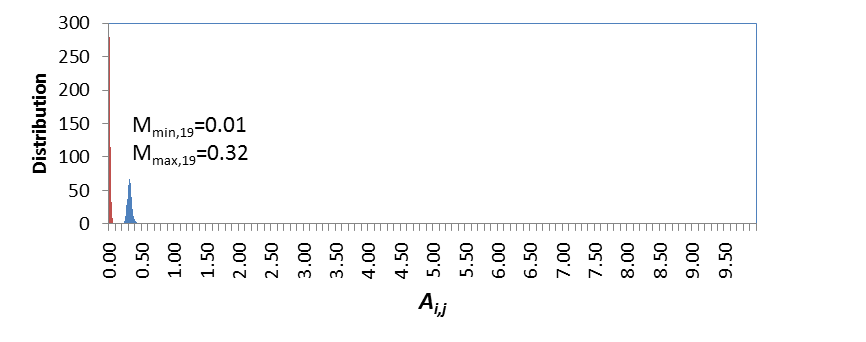


**Interacting atom type** 20


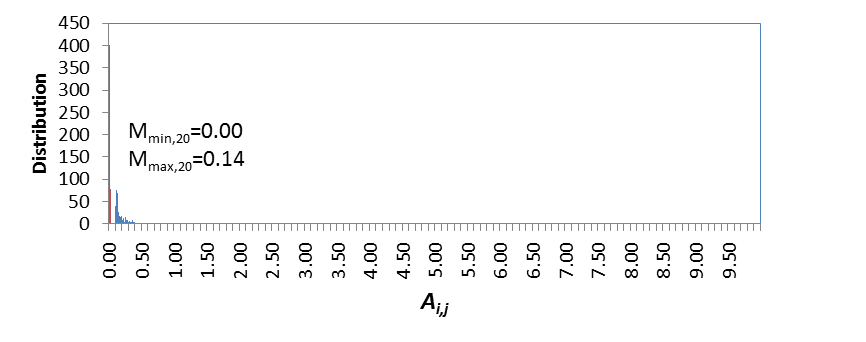


**Interacting atom type** 21


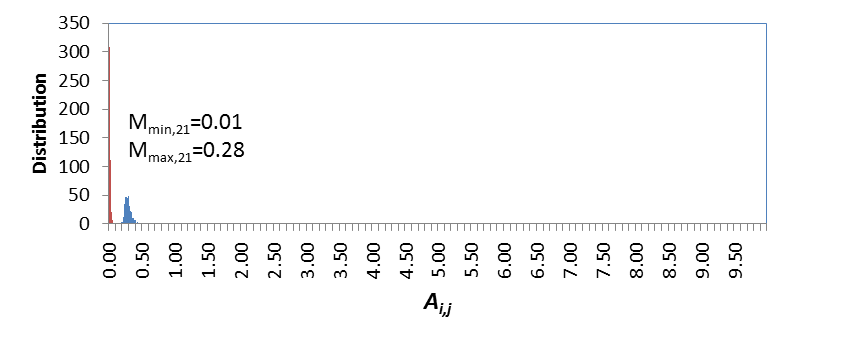


**Interacting atom type** 22


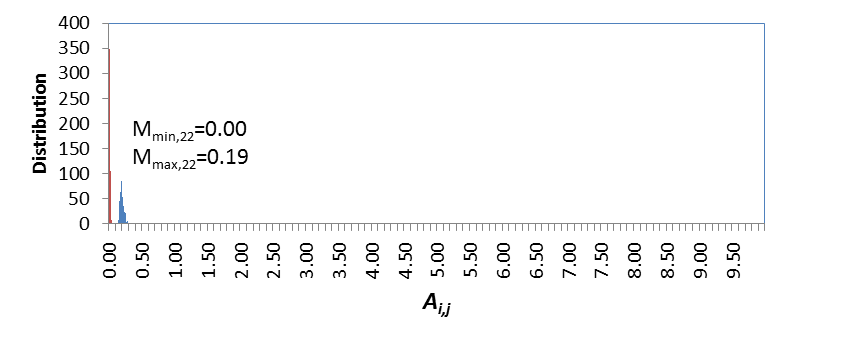


**Interacting atom type** 23


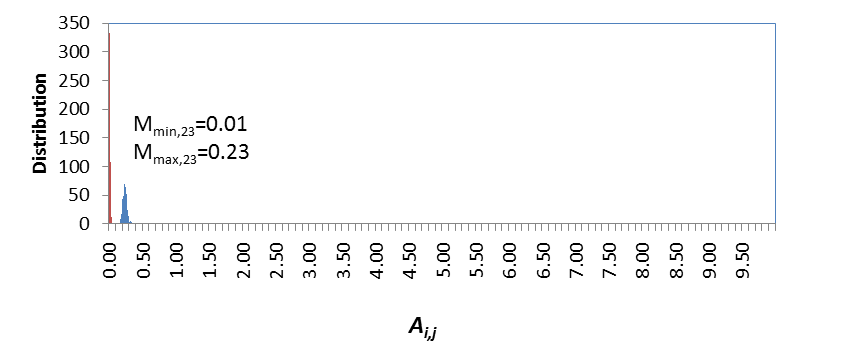


**Interacting atom type** 24


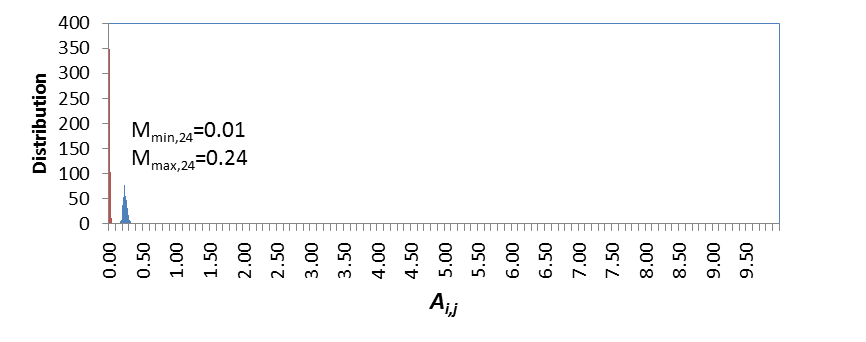


**Interacting atom type** 25


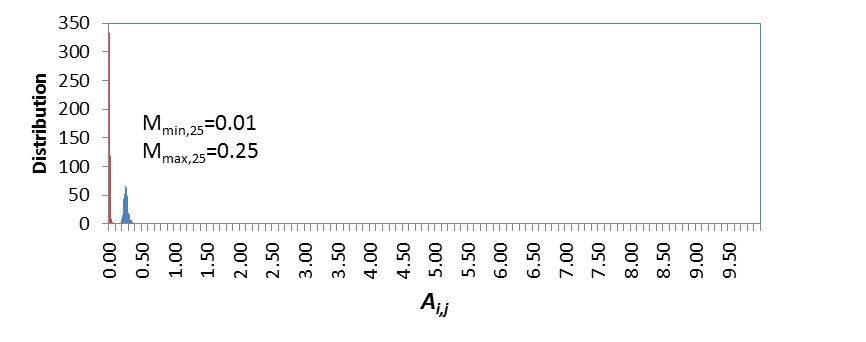


**Interacting atom type** 26


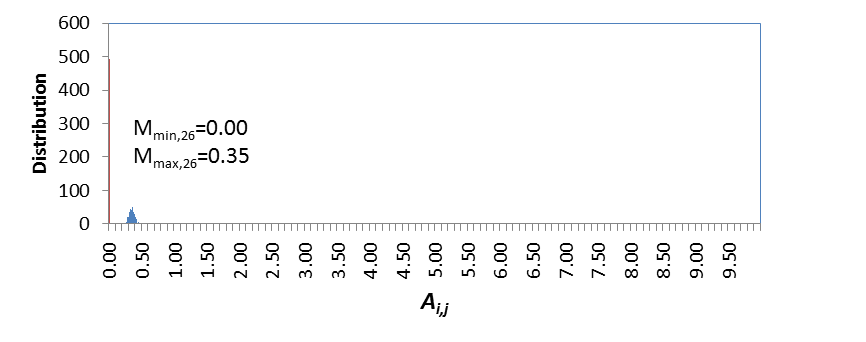


**Interacting atom type** 27


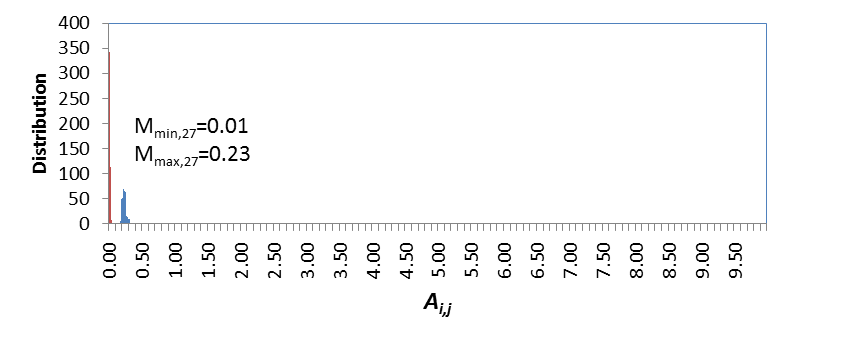


**Interacting atom type** 28


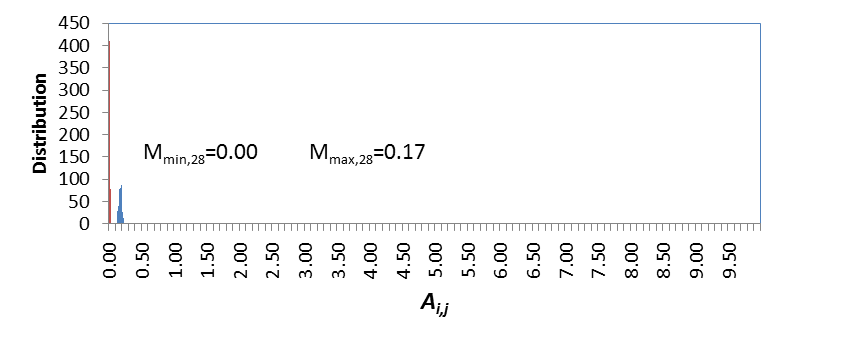


**Interacting atom type** 29


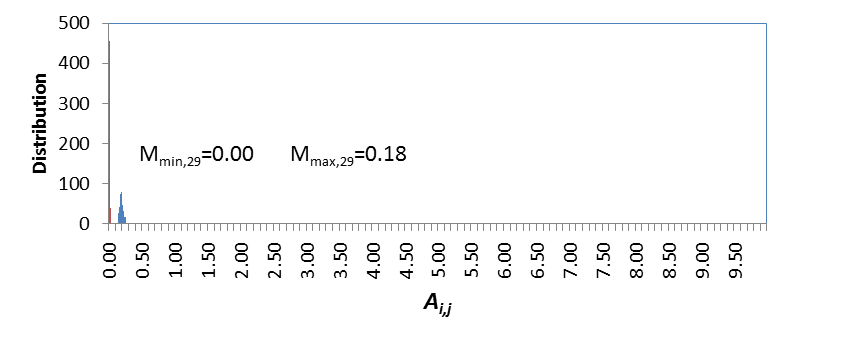


**Interacting atom type** 30


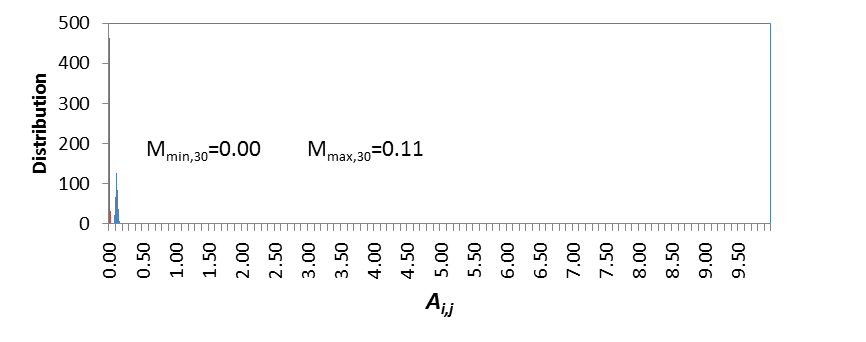


**Interacting atom type** 31


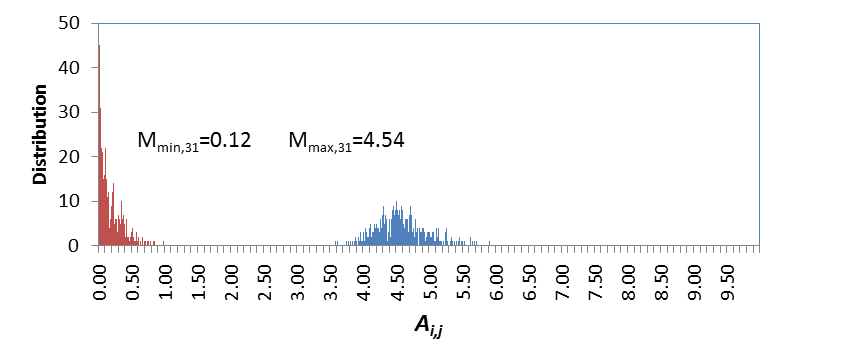


**Interacting atom type** 32


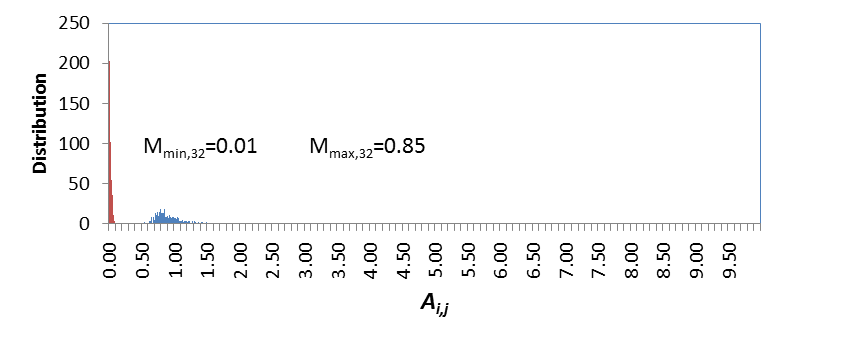


**Interacting atom type** 33


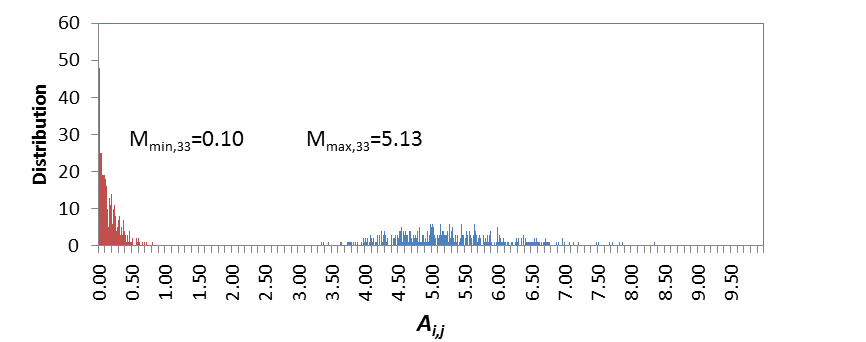


**Interacting atom type** 34


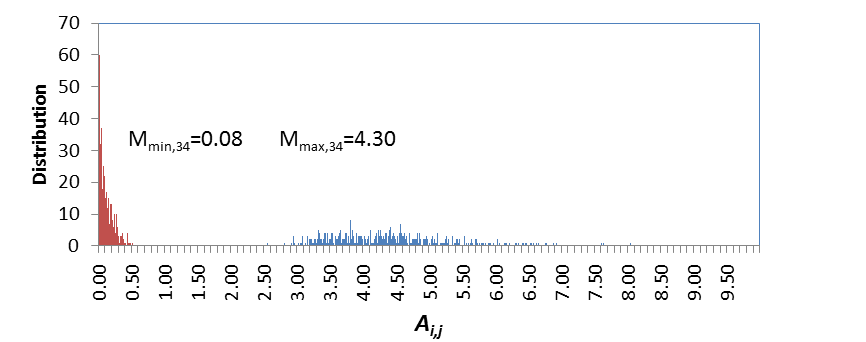


**Interacting atom type** 35


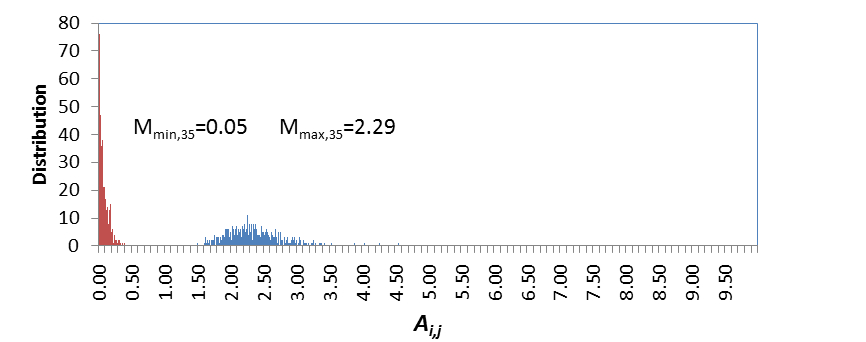


**Interacting atom type** 36

**
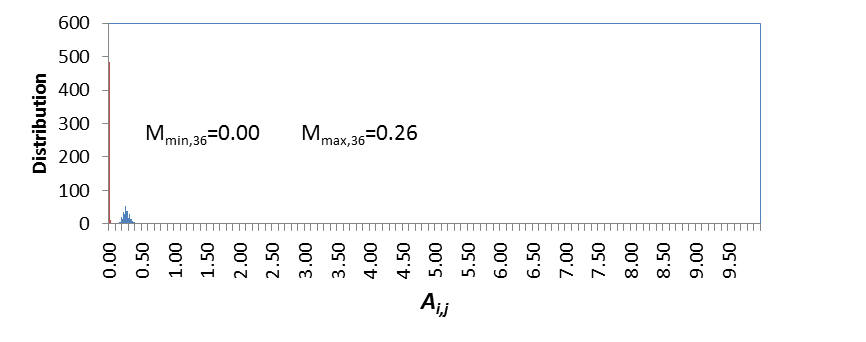
**

**Attribute** 37


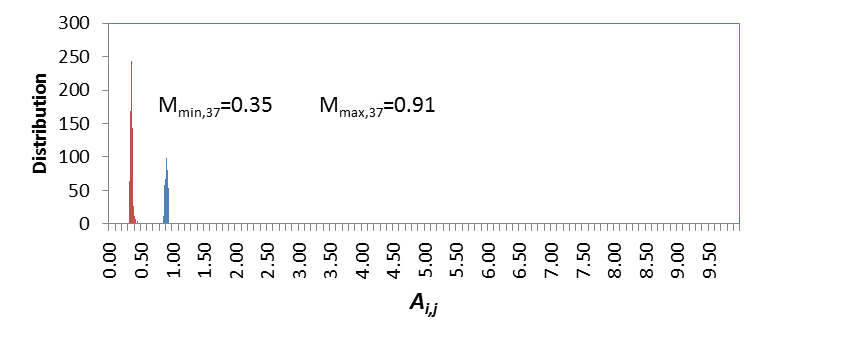


**Figure S2:** Distributions of maximal and minial *A_i,j_* (Equation (2) in the main text) calculated from the proteins in S497. *M_max,j_* shown in each of the panels is the median of the distribution of the maximal *A_i,j_* (distributions colored in blue) and *M_min,j_* is the median of the distribution of the minimal *A_i,j_* (distributions colored in red).
