# Supplementary material for: Prediction of Carbohydrate Binding Sites on Protein Surfaces with 3-Dimensional Probability Density Distributions of Interacting Atoms
Source: PLoS One. 2012 Jul 25;7(7):e40846. doi: 10.1371/journal.pone.0040846 (PMC3405063; doi:10.1371/journal.pone.0040846)
Supplement: Table S1 — A filter system used to eliminate non-interacting atomic pairs based on the work by McConkey et al with modifications. The carbohydrate-protein interactions were added to the Table by following the principle that aliphatic carbons do not interact with polar oxygen and nitrogen atoms. During the construction of the PDMs, only the atom pairs with the matrix value less than −0.1 were included in the probability density maps. The detail descriptions of the protein/carbohydrate atom types are shown in Table 1 in the main text. (DOC) [file pone.0040846.s004.doc]

**Table S1**

**Table S1:** A filter system used to eliminate non-interacting atomic pairs based on the work by McConkey et al with modifications. The carbohydrate-protein interactions were added to the Table by following the principle that aliphatic carbons do not interact with polar oxygen and nitrogen atoms. During the construction of the PDMs, only the atom pairs with the matrix value less than -0.1 were included in the probability density maps. The detail descriptions of the protein/carbohydrate atom types are shown in Table 1 in the main text.
