# Supplementary material for: Prediction of Carbohydrate Binding Sites on Protein Surfaces with 3-Dimensional Probability Density Distributions of Interacting Atoms
Source: PLoS One. 2012 Jul 25;7(7):e40846. doi: 10.1371/journal.pone.0040846 (PMC3405063; doi:10.1371/journal.pone.0040846)
Supplement: Table S2 — Ten-fold cross validation ANN prediction accuracy benchmarks on the S497 dataset. The dataset, the ten-fold cross validation, and the benchmark measurements have been described in the main text. Matthews correlation coefficient (MCC), F-score(Fsc), Accuracy(Acc), Precision(Pre), Sensitivity(Sen) and Specificity(Spe) are shown in Equations (4)∼(9). TP, FP, TN, and FN are true positive, false positive, true negative, and false negative respectively. C1∼C7 represent carbohydrate binding sites in each of the test proteins; different protein has different number of binding sites. In these columns, the number of the predicted true positive atoms is shown over the actual number of atoms involving in the binding site. Interactive examination of the prediction results for each of the proteins in the S497 dataset can be accessed from the web server: http://ismblab.genomics.sinica.edu.tw/> benchmark > protein-carbohydrate. (DOC) [file pone.0040846.s005.doc]

**Table S2**

| **PDBID** | **Residue-based ANN prediction benchmarks of ten-fold cross validation on S497 dataset** | | | | | | | | | | | | | | | | | |
| --- | --- | --- | --- | --- | --- | --- | --- | --- | --- | --- | --- | --- | --- | --- | --- | --- | --- | --- |
|  | **Residues Level** | | | | | | | | | | **Predict positive atoms / Actual binding atoms** | | | | | | | |
|  | **Acc** | **Pre** | **Sen** | **Spe** | **MCC** | **Fsc** | **TP** | **TN** | **FP** | **FN** | **All** | **C1** | **C2** | **C3** | **C4** | **C5** | **C6** | **C7** |
| 1MVQ | 1.00 | 1.00 | 1.00 | 1.00 | 1.00 | 1.00 | 10 | 197 | 0 | 0 | 30/33 | 30/33 | - | - | - | - | - | - |
| 1RPJ | 1.00 | 1.00 | 1.00 | 1.00 | 1.00 | 1.00 | 12 | 229 | 0 | 0 | 32/33 | 32/33 | - | - | - | - | - | - |
| 2JE7 | 1.00 | 1.00 | 0.90 | 1.00 | 0.95 | 0.95 | 9 | 202 | 0 | 1 | 30/36 | 30/36 | - | - | - | - | - | - |
| 2D6O | 0.99 | 1.00 | 0.88 | 1.00 | 0.93 | 0.93 | 7 | 136 | 0 | 1 | 27/31 | 27/31 | - | - | - | - | - | - |
| 1J8V | 0.99 | 0.85 | 1.00 | 0.99 | 0.92 | 0.92 | 17 | 501 | 3 | 0 | 59/60 | 59/60 | - | - | - | - | - | - |
| 2V4V | 0.99 | 1.00 | 0.86 | 1.00 | 0.92 | 0.92 | 6 | 110 | 0 | 1 | 33/35 | 33/35 | - | - | - | - | - | - |
| 1YOE | 0.99 | 0.81 | 1.00 | 0.99 | 0.90 | 0.90 | 13 | 248 | 3 | 0 | 31/31 | 31/31 | - | - | - | - | - | - |
| 2OVU | 0.99 | 1.00 | 0.82 | 1.00 | 0.90 | 0.90 | 9 | 197 | 0 | 2 | 33/40 | 33/40 | - | - | - | - | - | - |
| 2VGD | 0.98 | 0.83 | 1.00 | 0.98 | 0.90 | 0.91 | 20 | 153 | 4 | 0 | 72/82 | 72/82 | - | - | - | - | - | - |
| 1MG1 | 0.99 | 0.80 | 1.00 | 0.99 | 0.89 | 0.89 | 16 | 415 | 4 | 0 | 55/56 | 55/56 | - | - | - | - | - | - |
| 1K12 | 0.99 | 1.00 | 0.78 | 1.00 | 0.88 | 0.88 | 7 | 131 | 0 | 2 | 16/19 | 16/19 | - | - | - | - | - | - |
| 1KWF | 0.98 | 0.95 | 0.83 | 1.00 | 0.88 | 0.89 | 20 | 288 | 1 | 4 | 77/97 | 38/51 | 34/35 | 5/11 | - | - | - | - |
| 2J7M | 0.99 | 1.00 | 0.78 | 1.00 | 0.88 | 0.88 | 7 | 126 | 0 | 2 | 31/43 | 31/43 | - | - | - | - | - | - |
| 1FNZ | 0.99 | 1.00 | 0.77 | 1.00 | 0.87 | 0.87 | 10 | 196 | 0 | 3 | 29/41 | 29/41 | - | - | - | - | - | - |
| 1INV | 0.99 | 0.93 | 0.82 | 1.00 | 0.87 | 0.88 | 14 | 341 | 1 | 3 | 40/50 | 40/50 | - | - | - | - | - | - |
| 1US2 | 0.99 | 0.88 | 0.88 | 0.99 | 0.87 | 0.88 | 21 | 444 | 3 | 3 | 68/88 | 24/42 | 44/46 | - | - | - | - | - |
| 2VX6 | 0.98 | 0.81 | 0.96 | 0.98 | 0.87 | 0.88 | 21 | 285 | 5 | 1 | 74/80 | 74/80 | - | - | - | - | - | - |
| 1G1T | 0.98 | 1.00 | 0.75 | 1.00 | 0.86 | 0.86 | 9 | 131 | 0 | 3 | 16/25 | 16/25 | - | - | - | - | - | - |
| 1I82 | 0.98 | 0.91 | 0.83 | 0.99 | 0.86 | 0.87 | 10 | 159 | 1 | 2 | 39/45 | 39/45 | - | - | - | - | - | - |
| 2F0Z | 0.99 | 0.87 | 0.87 | 0.99 | 0.86 | 0.87 | 13 | 303 | 2 | 2 | 32/45 | 11/15 | 21/30 | - | - | - | - | - |
| 2FMD | 0.99 | 0.83 | 0.91 | 0.99 | 0.86 | 0.87 | 10 | 187 | 2 | 1 | 34/39 | 34/39 | - | - | - | - | - | - |
| 1KTC | 0.99 | 0.78 | 0.93 | 0.99 | 0.85 | 0.85 | 14 | 336 | 4 | 1 | 42/48 | 42/48 | - | - | - | - | - | - |
| 1S0I | 0.99 | 0.90 | 0.81 | 1.00 | 0.85 | 0.85 | 17 | 536 | 2 | 4 | 54/64 | 54/64 | - | - | - | - | - | - |
| 2VI0 | 0.98 | 0.86 | 0.86 | 0.99 | 0.85 | 0.86 | 19 | 214 | 3 | 3 | 79/88 | 79/88 | - | - | - | - | - | - |
| 1EU8 | 0.98 | 0.91 | 0.80 | 0.99 | 0.84 | 0.85 | 20 | 329 | 2 | 5 | 53/67 | 43/53 | 10/14 | - | - | - | - | - |
| 2B46 | 0.97 | 0.79 | 0.94 | 0.97 | 0.84 | 0.86 | 15 | 143 | 4 | 1 | 42/48 | 42/48 | - | - | - | - | - | - |
| 2ZID | 0.99 | 0.75 | 0.95 | 0.99 | 0.84 | 0.84 | 18 | 464 | 6 | 1 | 56/60 | 56/60 | - | - | - | - | - | - |
| 3G7W | 0.98 | 0.85 | 0.85 | 0.99 | 0.84 | 0.85 | 17 | 342 | 3 | 3 | 61/82 | 25/39 | 36/43 | - | - | - | - | - |
| 1DIL | 0.98 | 0.79 | 0.88 | 0.99 | 0.83 | 0.83 | 15 | 309 | 4 | 2 | 32/40 | 32/40 | - | - | - | - | - | - |
| 1ELJ | 0.98 | 0.78 | 0.90 | 0.98 | 0.83 | 0.84 | 18 | 301 | 5 | 2 | 65/78 | 65/78 | - | - | - | - | - | - |
| 1Y65 | 0.98 | 0.81 | 0.87 | 0.99 | 0.83 | 0.84 | 13 | 269 | 3 | 2 | 26/33 | 26/33 | - | - | - | - | - | - |
| 2GH9 | 0.98 | 0.74 | 0.94 | 0.98 | 0.83 | 0.83 | 17 | 313 | 6 | 1 | 70/74 | 70/74 | - | - | - | - | - | - |
| 3BYN | 0.99 | 0.83 | 0.83 | 0.99 | 0.83 | 0.83 | 15 | 369 | 3 | 3 | 49/59 | 49/59 | - | - | - | - | - | - |
| 1M03 | 0.99 | 0.74 | 0.93 | 0.99 | 0.82 | 0.82 | 14 | 427 | 5 | 1 | 51/53 | 51/53 | - | - | - | - | - | - |
| 2VVS | 0.99 | 0.76 | 0.89 | 0.99 | 0.82 | 0.82 | 16 | 493 | 5 | 2 | 48/52 | 48/52 | - | - | - | - | - | - |
| 2ZXT | 0.98 | 0.73 | 0.94 | 0.99 | 0.82 | 0.82 | 16 | 403 | 6 | 1 | 62/66 | 40/43 | 22/23 | - | - | - | - | - |
| 1CZA | 0.98 | 0.79 | 0.87 | 0.98 | 0.81 | 0.83 | 45 | 725 | 12 | 7 | 112/133 | 48/55 | 9/12 | 55/66 | - | - | - | - |
| 1FCV | 0.98 | 0.93 | 0.72 | 1.00 | 0.81 | 0.81 | 13 | 276 | 1 | 5 | 42/59 | 35/41 | 7/18 | - | - | - | - | - |
| 1Y4C | 0.98 | 0.67 | 1.00 | 0.98 | 0.81 | 0.80 | 16 | 422 | 8 | 0 | 55/59 | 55/59 | - | - | - | - | - | - |
| 2E2O | 0.97 | 0.68 | 1.00 | 0.97 | 0.81 | 0.81 | 15 | 235 | 7 | 0 | 41/41 | 41/41 | - | - | - | - | - | - |
| 2E9M | 0.98 | 0.67 | 1.00 | 0.98 | 0.81 | 0.80 | 14 | 387 | 7 | 0 | 34/34 | 34/34 | - | - | - | - | - | - |
| 2FVY | 0.97 | 0.67 | 1.00 | 0.97 | 0.81 | 0.80 | 14 | 246 | 7 | 0 | 49/49 | 49/49 | - | - | - | - | - | - |
| 2QQW | 0.99 | 0.71 | 0.94 | 0.99 | 0.81 | 0.81 | 15 | 455 | 6 | 1 | 50/53 | 50/53 | - | - | - | - | - | - |
| 1IS3 | 0.97 | 0.82 | 0.82 | 0.98 | 0.80 | 0.82 | 9 | 116 | 2 | 2 | 32/37 | 32/37 | - | - | - | - | - | - |
| 1QPK | 0.98 | 0.73 | 0.91 | 0.98 | 0.80 | 0.81 | 19 | 356 | 7 | 2 | 67/85 | 67/85 | - | - | - | - | - | - |
| 1R87 | 0.97 | 0.69 | 0.95 | 0.97 | 0.80 | 0.80 | 18 | 298 | 8 | 1 | 70/75 | 70/75 | - | - | - | - | - | - |
| 1RQ5 | 0.98 | 0.78 | 0.84 | 0.99 | 0.80 | 0.81 | 21 | 492 | 6 | 4 | 83/95 | 83/95 | - | - | - | - | - | - |
| 2OWZ | 0.98 | 0.71 | 0.92 | 0.98 | 0.80 | 0.80 | 12 | 277 | 5 | 1 | 23/32 | 23/32 | - | - | - | - | - | - |
| 7TAA | 0.98 | 0.90 | 0.72 | 1.00 | 0.80 | 0.80 | 18 | 407 | 2 | 7 | 62/93 | 48/59 | 14/34 | - | - | - | - | - |
| 1EHN | 0.97 | 0.96 | 0.67 | 1.00 | 0.79 | 0.79 | 26 | 440 | 1 | 13 | 97/157 | 73/107 | 24/30 | 0/20 | - | - | - | - |
| 1GZ9 | 0.98 | 1.00 | 0.64 | 1.00 | 0.79 | 0.78 | 9 | 206 | 0 | 5 | 25/43 | 25/43 | - | - | - | - | - | - |
| 1RWG | 0.98 | 0.67 | 0.95 | 0.99 | 0.79 | 0.78 | 20 | 654 | 10 | 1 | 72/77 | 72/77 | - | - | - | - | - | - |
| 1UAS | 0.98 | 0.64 | 1.00 | 0.97 | 0.79 | 0.78 | 14 | 300 | 8 | 0 | 40/41 | 40/41 | - | - | - | - | - | - |
| 2RJO | 0.97 | 0.64 | 1.00 | 0.97 | 0.79 | 0.78 | 14 | 278 | 8 | 0 | 45/47 | 45/47 | - | - | - | - | - | - |
| 2UVJ | 0.98 | 0.78 | 0.82 | 0.99 | 0.79 | 0.80 | 18 | 341 | 5 | 4 | 53/73 | 53/73 | - | - | - | - | - | - |
| 2YQS | 0.98 | 1.00 | 0.64 | 1.00 | 0.79 | 0.78 | 14 | 378 | 0 | 8 | 34/64 | 34/64 | - | - | - | - | - | - |
| 3H3K | 0.98 | 0.82 | 0.78 | 0.99 | 0.79 | 0.80 | 18 | 431 | 4 | 5 | 65/79 | 22/34 | 43/45 | - | - | - | - | - |
| 1LU1 | 0.98 | 1.00 | 0.62 | 1.00 | 0.78 | 0.76 | 8 | 216 | 0 | 5 | 18/39 | 18/39 | - | - | - | - | - | - |
| 1SLI | 0.98 | 0.61 | 1.00 | 0.98 | 0.78 | 0.76 | 19 | 592 | 12 | 0 | 39/40 | 39/40 | - | - | - | - | - | - |
| 1UQY | 0.97 | 0.76 | 0.83 | 0.98 | 0.78 | 0.79 | 19 | 288 | 6 | 4 | 72/90 | 40/58 | 32/32 | - | - | - | - | - |
| 2BVM | 0.98 | 0.62 | 1.00 | 0.98 | 0.78 | 0.77 | 13 | 473 | 8 | 0 | 32/33 | 32/33 | - | - | - | - | - | - |
| 2CIR | 0.98 | 0.63 | 1.00 | 0.98 | 0.78 | 0.77 | 10 | 239 | 6 | 0 | 31/33 | 31/33 | - | - | - | - | - | - |
| 2RJ7 | 0.96 | 0.70 | 0.91 | 0.97 | 0.78 | 0.79 | 19 | 240 | 8 | 2 | 57/73 | 36/45 | 21/28 | - | - | - | - | - |
| 2VMG | 0.98 | 1.00 | 0.63 | 1.00 | 0.78 | 0.77 | 5 | 125 | 0 | 3 | 15/23 | 15/23 | - | - | - | - | - | - |
| 2ZYN | 0.97 | 0.90 | 0.71 | 0.99 | 0.78 | 0.79 | 17 | 320 | 2 | 7 | 61/97 | 39/59 | 22/38 | - | - | - | - | - |
| 4A3H | 0.98 | 0.85 | 0.73 | 0.99 | 0.78 | 0.79 | 11 | 240 | 2 | 4 | 30/38 | 30/38 | - | - | - | - | - | - |
| 1G97 | 0.98 | 1.00 | 0.61 | 1.00 | 0.77 | 0.76 | 14 | 385 | 0 | 9 | 29/56 | 29/56 | - | - | - | - | - | - |
| 1LZC | 0.95 | 1.00 | 0.63 | 1.00 | 0.77 | 0.77 | 10 | 103 | 0 | 6 | 31/54 | 31/54 | - | - | - | - | - | - |
| 1MQE | 0.98 | 0.78 | 0.78 | 0.99 | 0.77 | 0.78 | 7 | 162 | 2 | 2 | 15/24 | 15/24 | - | - | - | - | - | - |
| 2A2D | 0.97 | 0.65 | 0.94 | 0.98 | 0.77 | 0.77 | 17 | 363 | 9 | 1 | 49/53 | 49/53 | - | - | - | - | - | - |
| 2FNC | 0.97 | 0.62 | 1.00 | 0.97 | 0.77 | 0.77 | 18 | 308 | 11 | 0 | 73/76 | 73/76 | - | - | - | - | - | - |
| 2WZG | 0.99 | 0.71 | 0.86 | 0.99 | 0.77 | 0.77 | 12 | 440 | 5 | 2 | 30/36 | 30/36 | - | - | - | - | - | - |
| 3D4C | 0.98 | 0.60 | 1.00 | 0.98 | 0.77 | 0.75 | 15 | 425 | 10 | 0 | 59/61 | 59/61 | - | - | - | - | - | - |
| 3HL3 | 0.97 | 0.77 | 0.81 | 0.98 | 0.77 | 0.79 | 13 | 200 | 4 | 3 | 26/36 | 26/36 | - | - | - | - | - | - |
| 3IM0 | 0.98 | 0.75 | 0.82 | 0.99 | 0.77 | 0.78 | 9 | 209 | 3 | 2 | 26/36 | 26/36 | - | - | - | - | - | - |
| 1B3Z | 0.97 | 0.88 | 0.68 | 0.99 | 0.76 | 0.77 | 15 | 241 | 2 | 7 | 50/88 | 0/24 | 50/64 | - | - | - | - | - |
| 1KJR | 0.97 | 0.78 | 0.78 | 0.98 | 0.76 | 0.78 | 7 | 120 | 2 | 2 | 22/35 | 14/25 | 8/10 | - | - | - | - | - |
| 1OUR | 0.96 | 1.00 | 0.60 | 1.00 | 0.76 | 0.75 | 6 | 92 | 0 | 4 | 14/29 | 14/29 | - | - | - | - | - | - |
| 1UA4 | 0.98 | 0.68 | 0.87 | 0.98 | 0.76 | 0.77 | 13 | 371 | 6 | 2 | 32/35 | 32/35 | - | - | - | - | - | - |
| 2IT6 | 0.97 | 1.00 | 0.60 | 1.00 | 0.76 | 0.75 | 6 | 113 | 0 | 4 | 14/29 | 14/29 | - | - | - | - | - | - |
| 3B9A | 0.97 | 0.79 | 0.77 | 0.99 | 0.76 | 0.78 | 26 | 449 | 7 | 8 | 97/130 | 29/39 | 46/62 | 22/29 | - | - | - | - |
| 3CUJ | 0.97 | 0.74 | 0.82 | 0.98 | 0.76 | 0.78 | 14 | 261 | 5 | 3 | 49/63 | 31/39 | 18/24 | - | - | - | - | - |
| 1C1L | 0.96 | 0.67 | 0.89 | 0.97 | 0.75 | 0.76 | 8 | 116 | 4 | 1 | 28/33 | 28/33 | - | - | - | - | - | - |
| 1KNM | 0.94 | 0.77 | 0.81 | 0.96 | 0.75 | 0.79 | 13 | 97 | 4 | 3 | 42/58 | 20/26 | 22/32 | - | - | - | - | - |
| 1URG | 0.97 | 0.62 | 0.93 | 0.98 | 0.75 | 0.74 | 13 | 329 | 8 | 1 | 52/53 | 52/53 | - | - | - | - | - | - |
| 2HTQ | 0.98 | 0.91 | 0.63 | 1.00 | 0.75 | 0.74 | 10 | 340 | 1 | 6 | 30/52 | 30/52 | - | - | - | - | - | - |
| 3F5F | 0.98 | 0.57 | 1.00 | 0.98 | 0.75 | 0.73 | 16 | 573 | 12 | 0 | 62/66 | 62/66 | - | - | - | - | - | - |
| 3F9M | 0.98 | 0.65 | 0.88 | 0.98 | 0.75 | 0.75 | 15 | 368 | 8 | 2 | 38/40 | 38/40 | - | - | - | - | - | - |
| 5CGT | 0.98 | 0.78 | 0.75 | 0.99 | 0.75 | 0.76 | 21 | 586 | 6 | 7 | 82/105 | 29/35 | 30/35 | 23/35 | - | - | - | - |
| 1KQY | 0.96 | 0.80 | 0.73 | 0.98 | 0.74 | 0.76 | 16 | 205 | 4 | 6 | 58/86 | 34/53 | 19/19 | 5/14 | - | - | - | - |
| 1N1T | 0.98 | 0.68 | 0.81 | 0.99 | 0.74 | 0.74 | 13 | 534 | 6 | 3 | 39/46 | 39/46 | - | - | - | - | - | - |
| 1S5M | 0.97 | 0.57 | 1.00 | 0.97 | 0.74 | 0.72 | 13 | 331 | 10 | 0 | 38/39 | 38/39 | - | - | - | - | - | - |
| 2FHF | 0.98 | 0.71 | 0.78 | 0.99 | 0.74 | 0.75 | 25 | 922 | 10 | 7 | 84/131 | 24/29 | 46/61 | 9/28 | 5/13 | - | - | - |
| 2OSX | 0.98 | 0.64 | 0.88 | 0.98 | 0.74 | 0.74 | 14 | 393 | 8 | 2 | 57/65 | 31/33 | 26/32 | - | - | - | - | - |
| 2P2V | 0.96 | 0.73 | 0.80 | 0.97 | 0.74 | 0.76 | 16 | 229 | 6 | 4 | 49/68 | 49/68 | - | - | - | - | - | - |
| 3EHS | 0.97 | 0.56 | 1.00 | 0.97 | 0.74 | 0.71 | 15 | 406 | 12 | 0 | 61/62 | 47/48 | 14/14 | - | - | - | - | - |
| 1Q33 | 0.97 | 0.67 | 0.83 | 0.98 | 0.73 | 0.74 | 10 | 252 | 5 | 2 | 28/33 | 28/33 | - | - | - | - | - | - |
| 2DT3 | 0.96 | 0.78 | 0.72 | 0.98 | 0.73 | 0.75 | 18 | 299 | 5 | 7 | 78/119 | 54/62 | 19/28 | 5/29 | - | - | - | - |
| 2VGQ | 0.97 | 0.71 | 0.77 | 0.98 | 0.73 | 0.74 | 17 | 394 | 7 | 5 | 62/96 | 30/54 | 32/42 | - | - | - | - | - |
| 2ZHN | 0.95 | 0.85 | 0.69 | 0.98 | 0.73 | 0.76 | 11 | 115 | 2 | 5 | 30/45 | 30/45 | - | - | - | - | - | - |
| 3FIZ | 0.97 | 0.56 | 1.00 | 0.97 | 0.73 | 0.71 | 15 | 370 | 12 | 0 | 31/34 | 31/34 | - | - | - | - | - | - |
| 1EOM | 0.96 | 0.82 | 0.67 | 0.99 | 0.72 | 0.74 | 14 | 213 | 3 | 7 | 41/72 | 39/50 | 2/22 | - | - | - | - | - |
| 1GNY | 0.97 | 0.78 | 0.70 | 0.99 | 0.72 | 0.74 | 7 | 129 | 2 | 3 | 32/43 | 32/43 | - | - | - | - | - | - |
| 1INW | 0.98 | 0.91 | 0.59 | 1.00 | 0.72 | 0.71 | 10 | 334 | 1 | 7 | 22/48 | 22/48 | - | - | - | - | - | - |
| 1T0O | 0.97 | 0.54 | 1.00 | 0.96 | 0.72 | 0.70 | 15 | 346 | 13 | 0 | 42/45 | 42/45 | - | - | - | - | - | - |
| 1W8N | 0.97 | 0.68 | 0.79 | 0.98 | 0.72 | 0.73 | 19 | 508 | 9 | 5 | 55/67 | 19/29 | 23/24 | 13/14 | - | - | - | - |
| 2C1Z | 0.97 | 0.57 | 0.94 | 0.97 | 0.72 | 0.71 | 16 | 374 | 12 | 1 | 49/59 | 29/39 | 20/20 | - | - | - | - | - |
| 2CHH | 0.95 | 1.00 | 0.55 | 1.00 | 0.72 | 0.71 | 6 | 89 | 0 | 5 | 16/33 | 16/33 | - | - | - | - | - | - |
| 2GH4 | 0.96 | 0.65 | 0.83 | 0.97 | 0.72 | 0.73 | 15 | 283 | 8 | 3 | 31/42 | 31/42 | - | - | - | - | - | - |
| 2W47 | 0.96 | 1.00 | 0.55 | 1.00 | 0.72 | 0.71 | 6 | 111 | 0 | 5 | 21/32 | 21/32 | - | - | - | - | - | - |
| 2WMG | 0.98 | 0.67 | 0.80 | 0.98 | 0.72 | 0.73 | 16 | 459 | 8 | 4 | 50/63 | 29/33 | 21/30 | - | - | - | - | - |
| 1BDG | 0.97 | 0.57 | 0.93 | 0.97 | 0.71 | 0.70 | 13 | 335 | 10 | 1 | 33/36 | 33/36 | - | - | - | - | - | - |
| 1CEN | 0.96 | 0.52 | 1.00 | 0.96 | 0.71 | 0.69 | 12 | 264 | 11 | 0 | 51/54 | 51/54 | - | - | - | - | - | - |
| 1KCD | 0.98 | 0.82 | 0.64 | 0.99 | 0.71 | 0.72 | 9 | 284 | 2 | 5 | 20/42 | 16/31 | 4/11 | - | - | - | - | - |
| 1PNF | 0.96 | 0.52 | 1.00 | 0.96 | 0.71 | 0.68 | 13 | 260 | 12 | 0 | 38/46 | 38/46 | - | - | - | - | - | - |
| 1QBB | 0.98 | 0.55 | 0.94 | 0.98 | 0.71 | 0.70 | 16 | 734 | 13 | 1 | 61/70 | 61/70 | - | - | - | - | - | - |
| 2VCB | 0.98 | 0.51 | 1.00 | 0.98 | 0.71 | 0.68 | 18 | 764 | 17 | 0 | 48/49 | 48/49 | - | - | - | - | - | - |
| 2WHM | 0.97 | 0.57 | 0.93 | 0.97 | 0.71 | 0.70 | 13 | 302 | 10 | 1 | 40/41 | 40/41 | - | - | - | - | - | - |
| 2Z1S | 0.96 | 0.66 | 0.81 | 0.97 | 0.71 | 0.72 | 21 | 363 | 11 | 5 | 51/69 | 11/20 | 35/37 | 5/12 | - | - | - | - |
| 1F9D | 0.95 | 0.66 | 0.80 | 0.96 | 0.70 | 0.73 | 37 | 483 | 19 | 9 | 133/169 | 97/122 | 36/47 | - | - | - | - | - |
| 1J84 | 0.97 | 0.86 | 0.60 | 0.99 | 0.70 | 0.71 | 6 | 147 | 1 | 4 | 20/38 | 15/27 | 5/11 | - | - | - | - | - |
| 1LSZ | 0.93 | 1.00 | 0.53 | 1.00 | 0.70 | 0.69 | 9 | 101 | 0 | 8 | 26/53 | 26/53 | - | - | - | - | - | - |
| 1UP2 | 0.95 | 0.62 | 0.84 | 0.96 | 0.70 | 0.71 | 16 | 232 | 10 | 3 | 52/65 | 52/65 | - | - | - | - | - | - |
| 1UU6 | 0.93 | 0.66 | 0.83 | 0.94 | 0.70 | 0.73 | 19 | 167 | 10 | 4 | 59/73 | 53/56 | 6/17 | - | - | - | - | - |
| 1W0O | 0.97 | 0.56 | 0.90 | 0.97 | 0.70 | 0.69 | 27 | 637 | 21 | 3 | 62/84 | 25/27 | 17/23 | 20/34 | - | - | - | - |
| 1ZDG | 0.97 | 0.56 | 0.90 | 0.97 | 0.70 | 0.69 | 9 | 218 | 7 | 1 | 23/27 | 23/27 | - | - | - | - | - | - |
| 2AEZ | 0.97 | 0.54 | 0.93 | 0.97 | 0.70 | 0.68 | 14 | 454 | 12 | 1 | 44/46 | 44/46 | - | - | - | - | - | - |
| 2C27 | 0.97 | 0.65 | 0.79 | 0.98 | 0.70 | 0.71 | 11 | 268 | 6 | 3 | 22/35 | 23/35 | - | - | - | - | - | - |
| 2VCE | 0.96 | 0.58 | 0.91 | 0.96 | 0.70 | 0.70 | 19 | 376 | 14 | 2 | 50/57 | 41/43 | 9/14 | - | - | - | - | - |
| 2W5O | 0.98 | 0.57 | 0.89 | 0.98 | 0.70 | 0.70 | 8 | 310 | 6 | 1 | 28/31 | 28/31 | - | - | - | - | - | - |
| 2YXS | 0.97 | 1.00 | 0.50 | 1.00 | 0.70 | 0.67 | 4 | 130 | 0 | 4 | 11/27 | 11/27 | - | - | - | - | - | - |
| 3JUL | 0.98 | 0.88 | 0.58 | 1.00 | 0.70 | 0.70 | 7 | 257 | 1 | 5 | 20/31 | 20/31 | - | - | - | - | - | - |
| 1EN2 | 0.96 | 1.00 | 0.50 | 1.00 | 0.69 | 0.67 | 3 | 76 | 0 | 3 | 14/36 | 14/36 | - | - | - | - | - | - |
| 1LZR | 0.94 | 0.83 | 0.63 | 0.98 | 0.69 | 0.71 | 10 | 105 | 2 | 6 | 33/59 | 3/23 | 30/36 | - | - | - | - | - |
| 1V03 | 0.97 | 0.57 | 0.87 | 0.98 | 0.69 | 0.68 | 13 | 403 | 10 | 2 | 39/41 | 39/41 | - | - | - | - | - | - |
| 2JEN | 0.93 | 0.83 | 0.65 | 0.98 | 0.69 | 0.73 | 20 | 164 | 4 | 11 | 71/118 | 71/107 | 0/11 | - | - | - | - | - |
| 1A9T | 0.97 | 0.59 | 0.83 | 0.97 | 0.68 | 0.69 | 10 | 239 | 7 | 2 | 27/38 | 27/38 | - | - | - | - | - | - |
| 1JIL | 0.98 | 0.73 | 0.67 | 0.99 | 0.68 | 0.70 | 8 | 287 | 3 | 4 | 18/38 | 18/38 | - | - | - | - | - | - |
| 2DEJ | 0.96 | 0.57 | 0.86 | 0.97 | 0.68 | 0.69 | 12 | 268 | 9 | 2 | 31/40 | 31/40 | - | - | - | - | - | - |
| 2HW1 | 0.96 | 0.58 | 0.85 | 0.97 | 0.68 | 0.69 | 11 | 245 | 8 | 2 | 30/36 | 30/36 | - | - | - | - | - | - |
| 2V0I | 0.98 | 1.00 | 0.47 | 1.00 | 0.68 | 0.64 | 8 | 397 | 0 | 9 | 17/51 | 17/51 | - | - | - | - | - | - |
| 2YVW | 0.97 | 0.65 | 0.73 | 0.98 | 0.68 | 0.69 | 11 | 359 | 6 | 4 | 29/39 | 29/39 | - | - | - | - | - | - |
| 3DM0 | 0.97 | 0.47 | 1.00 | 0.97 | 0.68 | 0.64 | 15 | 569 | 17 | 0 | 56/59 | 37/37 | 19/22 | - | - | - | - | - |
| 1BYH | 0.94 | 0.52 | 0.93 | 0.94 | 0.67 | 0.67 | 13 | 174 | 12 | 1 | 39/46 | 39/46 | - | - | - | - | - | - |
| 1E6X | 0.97 | 0.50 | 0.92 | 0.97 | 0.67 | 0.65 | 11 | 407 | 11 | 1 | 34/37 | 34/37 | - | - | - | - | - | - |
| 1EUS | 0.97 | 0.65 | 0.73 | 0.98 | 0.67 | 0.69 | 11 | 296 | 6 | 4 | 32/42 | 32/42 | - | - | - | - | - | - |
| 1G9R | 0.95 | 0.52 | 0.93 | 0.95 | 0.67 | 0.67 | 13 | 233 | 12 | 1 | 34/39 | 34/39 | - | - | - | - | - | - |
| 2FUE | 0.96 | 0.53 | 0.89 | 0.97 | 0.67 | 0.67 | 8 | 207 | 7 | 1 | 15/32 | 15/32 | - | - | - | - | - | - |
| 3GH5 | 0.97 | 0.50 | 0.93 | 0.97 | 0.67 | 0.65 | 13 | 416 | 13 | 1 | 46/48 | 46/48 | - | - | - | - | - | - |
| 1G9F | 0.97 | 1.00 | 0.46 | 1.00 | 0.66 | 0.63 | 5 | 196 | 0 | 6 | 11/31 | 11/31 | - | - | - | - | - | - |
| 1GAI | 0.98 | 0.59 | 0.77 | 0.98 | 0.66 | 0.67 | 10 | 410 | 7 | 3 | 37/55 | 37/55 | - | - | - | - | - | - |
| 1IUC | 0.94 | 0.76 | 0.63 | 0.98 | 0.66 | 0.69 | 19 | 255 | 6 | 11 | 51/84 | 16/30 | 35/54 | - | - | - | - | - |
| 1KWK | 0.98 | 0.44 | 1.00 | 0.97 | 0.66 | 0.62 | 12 | 566 | 15 | 0 | 37/37 | 37/37 | - | - | - | - | - | - |
| 1L8N | 0.97 | 0.57 | 0.81 | 0.98 | 0.66 | 0.67 | 17 | 559 | 13 | 4 | 67/76 | 67/76 | - | - | - | - | - | - |
| 1Q6D | 0.96 | 0.67 | 0.71 | 0.97 | 0.66 | 0.69 | 22 | 410 | 11 | 9 | 56/90 | 44/77 | 12/13 | - | - | - | - | - |
| 1RKD | 0.96 | 0.50 | 0.92 | 0.96 | 0.66 | 0.65 | 11 | 247 | 11 | 1 | 28/33 | 28/33 | - | - | - | - | - | - |
| 1TJ4 | 0.94 | 0.60 | 0.80 | 0.96 | 0.66 | 0.69 | 12 | 174 | 8 | 3 | 27/48 | 27/48 | - | - | - | - | - | - |
| 2BF6 | 0.96 | 0.52 | 0.88 | 0.96 | 0.66 | 0.65 | 15 | 365 | 14 | 2 | 42/48 | 42/48 | - | - | - | - | - | - |
| 2BOD | 0.95 | 0.65 | 0.72 | 0.97 | 0.66 | 0.68 | 13 | 215 | 7 | 5 | 52/69 | 52/69 | - | - | - | - | - | - |
| 2IW1 | 0.96 | 0.63 | 0.75 | 0.97 | 0.66 | 0.68 | 15 | 296 | 9 | 5 | 35/53 | 15/22 | 15/19 | 5/12 | - | - | - | - |
| 3CKQ | 0.97 | 0.58 | 0.78 | 0.98 | 0.66 | 0.67 | 7 | 256 | 5 | 2 | 23/33 | 13/18 | 10/15 | - | - | - | - | - |
| 3GNP | 0.96 | 0.45 | 1.00 | 0.96 | 0.66 | 0.62 | 14 | 395 | 17 | 0 | 39/39 | 39/39 | - | - | - | - | - | - |
| 1C3N | 0.96 | 1.00 | 0.44 | 1.00 | 0.65 | 0.62 | 4 | 126 | 0 | 5 | 7/26 | 7/26 | - | - | - | - | - | - |
| 1GOQ | 0.95 | 0.87 | 0.52 | 0.99 | 0.65 | 0.65 | 13 | 238 | 2 | 12 | 46/96 | 46/74 | 0/22 | - | - | - | - | - |
| 1GUI | 0.92 | 0.60 | 0.80 | 0.94 | 0.65 | 0.69 | 12 | 122 | 8 | 3 | 48/61 | 38/40 | 10/21 | - | - | - | - | - |
| 1HKK | 0.94 | 0.52 | 0.90 | 0.95 | 0.65 | 0.65 | 17 | 285 | 16 | 2 | 75/89 | 50/54 | 25/35 | - | - | - | - | - |
| 1HM2 | 0.97 | 0.47 | 0.94 | 0.97 | 0.65 | 0.63 | 15 | 568 | 17 | 1 | 65/73 | 65/73 | - | - | - | - | - | - |
| 1O03 | 0.95 | 0.75 | 0.60 | 0.98 | 0.65 | 0.67 | 9 | 178 | 3 | 6 | 21/40 | 21/40 | - | - | - | - | - | - |
| 1Y3P | 0.95 | 0.54 | 0.84 | 0.96 | 0.65 | 0.66 | 21 | 409 | 18 | 4 | 70/87 | 50/51 | 20/36 | - | - | - | - | - |
| 2OEG | 0.98 | 1.00 | 0.43 | 1.00 | 0.65 | 0.60 | 6 | 441 | 0 | 8 | 16/39 | 16/39 | - | - | - | - | - | - |
| 2VW1 | 0.98 | 0.56 | 0.78 | 0.98 | 0.65 | 0.65 | 14 | 567 | 11 | 4 | 29/42 | 29/42 | - | - | - | - | - | - |
| 2WZF | 0.98 | 0.55 | 0.79 | 0.98 | 0.65 | 0.65 | 11 | 453 | 9 | 3 | 26/34 | 26/34 | - | - | - | - | - | - |
| 2YVP | 0.96 | 0.78 | 0.58 | 0.99 | 0.65 | 0.67 | 7 | 157 | 2 | 5 | 15/42 | 15/42 | - | - | - | - | - | - |
| 154L | 0.94 | 1.00 | 0.44 | 1.00 | 0.64 | 0.61 | 7 | 140 | 0 | 9 | 17/48 | 6/19 | 11/29 | - | - | - | - | - |
| 1GZ1 | 0.94 | 0.59 | 0.77 | 0.95 | 0.64 | 0.67 | 20 | 284 | 14 | 6 | 70/97 | 62/87 | 8/10 | - | - | - | - | - |
| 1PMH | 0.96 | 0.86 | 0.50 | 0.99 | 0.64 | 0.63 | 6 | 159 | 1 | 6 | 35/54 | 35/54 | - | - | - | - | - | - |
| 1QHO | 0.96 | 0.85 | 0.51 | 0.99 | 0.64 | 0.64 | 22 | 574 | 4 | 21 | 84/156 | 59/81 | 0/31 | 8/12 | 17/32 | - | - | - |
| 2IT5 | 0.94 | 0.64 | 0.70 | 0.96 | 0.64 | 0.67 | 7 | 108 | 4 | 3 | 17/24 | 17/24 | - | - | - | - | - | - |
| 2J44 | 0.95 | 0.61 | 0.73 | 0.96 | 0.64 | 0.67 | 11 | 189 | 7 | 4 | 55/68 | 23/34 | 32/34 | - | - | - | - | - |
| 2V72 | 0.97 | 1.00 | 0.43 | 1.00 | 0.64 | 0.60 | 3 | 123 | 0 | 4 | 8/30 | 8/30 | - | - | - | - | - | - |
| 2ZAA | 0.93 | 0.93 | 0.48 | 1.00 | 0.64 | 0.63 | 13 | 181 | 1 | 14 | 29/73 | 18/47 | 11/26 | - | - | - | - | - |
| 3CA3 | 0.95 | 0.83 | 0.53 | 0.99 | 0.64 | 0.65 | 10 | 214 | 2 | 9 | 32/72 | 19/38 | 13/34 | - | - | - | - | - |
| 3E6J | 0.96 | 0.64 | 0.69 | 0.97 | 0.64 | 0.67 | 9 | 188 | 5 | 4 | 39/53 | 39/53 | - | - | - | - | - | - |
| 1B1Y | 0.95 | 0.55 | 0.78 | 0.96 | 0.63 | 0.65 | 21 | 403 | 17 | 6 | 51/76 | 51/76 | - | - | - | - | - | - |
| 1P5G | 0.96 | 0.41 | 1.00 | 0.95 | 0.63 | 0.59 | 12 | 356 | 17 | 0 | 30/32 | 30/32 | - | - | - | - | - | - |
| 1PIE | 0.96 | 0.58 | 0.73 | 0.98 | 0.63 | 0.65 | 11 | 315 | 8 | 4 | 27/40 | 27/40 | - | - | - | - | - | - |
| 1UA7 | 0.96 | 0.59 | 0.72 | 0.98 | 0.63 | 0.65 | 13 | 355 | 9 | 5 | 35/59 | 35/59 | - | - | - | - | - | - |
| 2B4F | 0.94 | 0.73 | 0.59 | 0.98 | 0.63 | 0.66 | 19 | 301 | 7 | 13 | 74/125 | 42/54 | 18/29 | 0/23 | 14/19 | - | - | - |
| 2BS7 | 0.95 | 0.53 | 0.80 | 0.96 | 0.63 | 0.64 | 8 | 147 | 7 | 2 | 25/39 | 25/39 | - | - | - | - | - | - |
| 3CKZ | 0.96 | 0.61 | 0.70 | 0.97 | 0.63 | 0.65 | 14 | 321 | 9 | 6 | 36/51 | 36/51 | - | - | - | - | - | - |
| 1G93 | 0.95 | 0.45 | 0.90 | 0.96 | 0.62 | 0.60 | 9 | 237 | 11 | 1 | 28/38 | 28/38 | - | - | - | - | - | - |
| 1KC3 | 0.98 | 0.71 | 0.56 | 0.99 | 0.62 | 0.63 | 5 | 261 | 2 | 4 | 18/32 | 18/32 | - | - | - | - | - | - |
| 1UAE | 0.96 | 0.50 | 0.81 | 0.96 | 0.62 | 0.62 | 13 | 340 | 13 | 3 | 32/51 | 32/51 | - | - | - | - | - | - |
| 1WD4 | 0.95 | 0.68 | 0.61 | 0.98 | 0.62 | 0.64 | 19 | 387 | 9 | 12 | 52/100 | 8/31 | 14/34 | 30/35 | - | - | - | - |
| 1Y9G | 0.96 | 0.39 | 1.00 | 0.96 | 0.62 | 0.56 | 11 | 437 | 17 | 0 | 39/39 | 39/39 | - | - | - | - | - | - |
| 2ACQ | 0.95 | 0.41 | 1.00 | 0.95 | 0.62 | 0.58 | 9 | 260 | 13 | 0 | 27/28 | 27/28 | - | - | - | - | - | - |
| 2BS5 | 0.86 | 0.82 | 0.61 | 0.95 | 0.62 | 0.70 | 14 | 60 | 3 | 9 | 38/70 | 29/38 | 9/32 | - | - | - | - | - |
| 2CGL | 0.96 | 0.41 | 1.00 | 0.96 | 0.62 | 0.58 | 13 | 404 | 19 | 0 | 41/41 | 41/41 | - | - | - | - | - | - |
| 2HTW | 0.97 | 0.80 | 0.50 | 0.99 | 0.62 | 0.62 | 8 | 332 | 2 | 8 | 21/47 | 21/47 | - | - | - | - | - | - |
| 2RL2 | 0.96 | 0.54 | 0.77 | 0.97 | 0.62 | 0.63 | 13 | 337 | 11 | 4 | 38/58 | 26/28 | 12/30 | - | - | - | - | - |
| 3B8A | 0.97 | 0.55 | 0.75 | 0.98 | 0.62 | 0.63 | 12 | 391 | 10 | 4 | 30/42 | 30/42 | - | - | - | - | - | - |
| 3BC9 | 0.95 | 0.70 | 0.59 | 0.98 | 0.62 | 0.64 | 23 | 486 | 10 | 16 | 101/178 | 27/42 | 38/42 | 34/44 | 2/50 | - | - | - |
| 3CZK | 0.96 | 0.43 | 0.94 | 0.96 | 0.62 | 0.59 | 17 | 527 | 23 | 1 | 50/56 | 50/56 | - | - | - | - | - | - |
| 1Z45 | 0.97 | 0.62 | 0.62 | 0.99 | 0.61 | 0.62 | 13 | 574 | 8 | 8 | 33/63 | 0/26 | 33/37 | - | - | - | - | - |
| 2D0G | 0.94 | 0.77 | 0.54 | 0.98 | 0.61 | 0.63 | 30 | 507 | 9 | 26 | 102/230 | 11/50 | 17/44 | 10/24 | 36/49 | 16/22 | 12/27 | 0/14 |
| 2IXB | 0.96 | 0.43 | 0.92 | 0.96 | 0.61 | 0.59 | 12 | 354 | 16 | 1 | 25/30 | 10/15 | 15/15 | - | - | - | - | - |
| 2WAO | 0.96 | 0.71 | 0.56 | 0.99 | 0.61 | 0.63 | 10 | 267 | 4 | 8 | 49/79 | 49/79 | - | - | - | - | - | - |
| 3BMW | 0.96 | 0.57 | 0.70 | 0.97 | 0.61 | 0.63 | 21 | 556 | 16 | 9 | 74/112 | 11/30 | 54/65 | 9/17 | - | - | - | - |
| 1I24 | 0.97 | 0.67 | 0.57 | 0.99 | 0.60 | 0.62 | 8 | 315 | 4 | 6 | 20/37 | 20/37 | - | - | - | - | - | - |
| 1LMQ | 0.91 | 0.71 | 0.59 | 0.96 | 0.60 | 0.65 | 10 | 97 | 4 | 7 | 37/56 | 37/56 | - | - | - | - | - | - |
| 1URX | 0.91 | 0.67 | 0.63 | 0.95 | 0.60 | 0.65 | 20 | 208 | 10 | 12 | 70/107 | 18/48 | 52/59 | - | - | - | - | - |
| 2G3J | 0.96 | 0.59 | 0.67 | 0.97 | 0.60 | 0.63 | 10 | 252 | 7 | 5 | 35/63 | 35/63 | - | - | - | - | - | - |
| 3B50 | 0.95 | 0.61 | 0.65 | 0.97 | 0.60 | 0.63 | 11 | 257 | 7 | 6 | 26/53 | 26/53 | - | - | - | - | - | - |
| 3HKN | 0.94 | 0.63 | 0.63 | 0.97 | 0.60 | 0.63 | 12 | 208 | 7 | 7 | 28/49 | 28/49 | - | - | - | - | - | - |
| 1CNQ | 0.95 | 0.75 | 0.50 | 0.99 | 0.59 | 0.60 | 12 | 273 | 4 | 12 | 26/62 | 26/37 | 0/25 | - | - | - | - | - |
| 1W9W | 0.92 | 0.78 | 0.50 | 0.98 | 0.59 | 0.61 | 7 | 103 | 2 | 7 | 31/59 | 14/41 | 17/18 | - | - | - | - | - |
| 2EQD | 0.95 | 0.55 | 0.68 | 0.97 | 0.59 | 0.61 | 17 | 419 | 14 | 8 | 61/101 | 10/30 | 31/41 | 20/30 | - | - | - | - |
| 1GU3 | 0.94 | 0.75 | 0.50 | 0.98 | 0.58 | 0.60 | 6 | 124 | 2 | 6 | 28/53 | 28/53 | - | - | - | - | - | - |
| 1Z4X | 0.96 | 0.52 | 0.69 | 0.98 | 0.58 | 0.60 | 11 | 383 | 10 | 5 | 34/47 | 25/36 | 9/11 | - | - | - | - | - |
| 2PHH | 0.96 | 0.35 | 1.00 | 0.96 | 0.58 | 0.52 | 8 | 344 | 15 | 0 | 23/27 | 23/27 | - | - | - | - | - | - |
| 2YHX | 0.98 | 0.63 | 0.56 | 0.99 | 0.58 | 0.59 | 5 | 345 | 3 | 4 | 12/20 | 12/20 | - | - | - | - | - | - |
| 4RHN | 0.93 | 1.00 | 0.36 | 1.00 | 0.58 | 0.53 | 4 | 91 | 0 | 7 | 10/29 | 10/29 | - | - | - | - | - | - |
| 1K1W | 0.96 | 0.36 | 0.92 | 0.96 | 0.57 | 0.52 | 12 | 527 | 21 | 1 | 37/40 | 37/40 | - | - | - | - | - | - |
| 1LED | 0.95 | 0.67 | 0.53 | 0.98 | 0.57 | 0.59 | 8 | 204 | 4 | 7 | 28/64 | 28/64 | - | - | - | - | - | - |
| 1R6D | 0.95 | 0.46 | 0.77 | 0.96 | 0.57 | 0.57 | 10 | 267 | 12 | 3 | 15/24 | 15/24 | - | - | - | - | - | - |
| 1XC6 | 0.97 | 0.33 | 1.00 | 0.97 | 0.57 | 0.50 | 13 | 865 | 26 | 0 | 35/36 | 35/36 | - | - | - | - | - | - |
| 2W52 | 0.93 | 0.50 | 0.74 | 0.94 | 0.57 | 0.60 | 14 | 229 | 14 | 5 | 48/73 | 48/73 | - | - | - | - | - | - |
| 3H4I | 0.96 | 0.59 | 0.59 | 0.98 | 0.57 | 0.59 | 10 | 334 | 7 | 7 | 29/56 | 29/56 | - | - | - | - | - | - |
| 1FOA | 0.94 | 0.46 | 0.75 | 0.95 | 0.56 | 0.57 | 12 | 276 | 14 | 4 | 38/56 | 28/37 | 10/19 | - | - | - | - | - |
| 2EAE | 0.97 | 0.35 | 0.93 | 0.97 | 0.56 | 0.51 | 14 | 762 | 26 | 1 | 53/60 | 53/60 | - | - | - | - | - | - |
| 2V8K | 0.96 | 0.39 | 0.86 | 0.96 | 0.56 | 0.53 | 12 | 462 | 19 | 2 | 30/45 | 30/45 | - | - | - | - | - | - |
| 2WHL | 0.94 | 0.38 | 0.90 | 0.94 | 0.56 | 0.53 | 9 | 221 | 15 | 1 | 39/49 | 39/49 | - | - | - | - | - | - |
| 3C7G | 0.96 | 0.44 | 0.77 | 0.97 | 0.56 | 0.56 | 10 | 397 | 13 | 3 | 33/50 | 21/23 | 12/27 | - | - | - | - | - |
| 4ENG | 0.91 | 0.77 | 0.48 | 0.98 | 0.56 | 0.59 | 13 | 162 | 4 | 14 | 33/88 | 27/66 | 6/22 | - | - | - | - | - |
| 1F0P | 0.93 | 0.75 | 0.46 | 0.98 | 0.55 | 0.57 | 12 | 220 | 4 | 14 | 24/82 | 22/47 | 2/35 | - | - | - | - | - |
| 1G0C | 0.96 | 0.40 | 0.80 | 0.96 | 0.55 | 0.53 | 8 | 291 | 12 | 2 | 26/36 | 26/36 | - | - | - | - | - | - |
| 1LOH | 0.96 | 0.57 | 0.57 | 0.98 | 0.55 | 0.57 | 17 | 616 | 13 | 13 | 62/106 | 43/53 | 8/10 | 10/28 | 1/15 | - | - | - |
| 1Z3W | 0.94 | 0.32 | 1.00 | 0.94 | 0.55 | 0.49 | 11 | 349 | 23 | 0 | 40/40 | 40/40 | - | - | - | - | - | - |
| 3DJ4 | 0.96 | 0.69 | 0.48 | 0.99 | 0.55 | 0.56 | 11 | 400 | 5 | 12 | 22/60 | 22/60 | - | - | - | - | - | - |
| 3MAN | 0.94 | 0.41 | 0.82 | 0.95 | 0.55 | 0.55 | 9 | 224 | 13 | 2 | 29/45 | 29/45 | - | - | - | - | - | - |
| 6CEL | 0.92 | 0.71 | 0.50 | 0.97 | 0.55 | 0.59 | 24 | 340 | 10 | 24 | 94/183 | 38/59 | 48/64 | 8/60 | - | - | - | - |
| 1IA7 | 0.95 | 0.31 | 1.00 | 0.95 | 0.54 | 0.47 | 8 | 347 | 18 | 0 | 37/37 | 37/37 | - | - | - | - | - | - |
| 1ITC | 0.92 | 0.85 | 0.39 | 0.99 | 0.54 | 0.54 | 23 | 415 | 4 | 36 | 67/198 | 43/90 | 11/34 | 0/45 | 13/29 | - | - | - |
| 1ULV | 0.98 | 0.38 | 0.79 | 0.98 | 0.54 | 0.51 | 11 | 893 | 18 | 3 | 35/43 | 35/43 | - | - | - | - | - | - |
| 1UXY | 0.96 | 0.70 | 0.44 | 0.99 | 0.54 | 0.54 | 7 | 290 | 3 | 9 | 18/42 | 18/42 | - | - | - | - | - | - |
| 2VJJ | 0.95 | 0.62 | 0.51 | 0.98 | 0.54 | 0.56 | 18 | 489 | 11 | 17 | 48/108 | 31/40 | 13/54 | 4/14 | - | - | - | - |
| 1D0M | 0.94 | 0.71 | 0.44 | 0.98 | 0.53 | 0.54 | 10 | 248 | 4 | 13 | 34/81 | 3/32 | 31/49 | - | - | - | - | - |
| 1UY4 | 0.92 | 1.00 | 0.31 | 1.00 | 0.53 | 0.47 | 4 | 105 | 0 | 9 | 23/52 | 23/52 | - | - | - | - | - | - |
| 2H44 | 0.95 | 0.41 | 0.75 | 0.96 | 0.53 | 0.53 | 9 | 276 | 13 | 3 | 22/34 | 22/34 | - | - | - | - | - | - |
| 2JEQ | 0.94 | 0.50 | 0.63 | 0.96 | 0.53 | 0.56 | 12 | 292 | 12 | 7 | 43/71 | 37/52 | 6/19 | - | - | - | - | - |
| 2WRA | 0.94 | 1.00 | 0.30 | 1.00 | 0.53 | 0.46 | 3 | 102 | 0 | 7 | 10/39 | 10/39 | - | - | - | - | - | - |
| 2AXR | 0.96 | 0.28 | 1.00 | 0.96 | 0.52 | 0.44 | 7 | 409 | 18 | 0 | 32/32 | 32/32 | - | - | - | - | - | - |
| 3FAX | 0.97 | 0.31 | 0.90 | 0.97 | 0.52 | 0.46 | 9 | 668 | 20 | 1 | 32/41 | 32/41 | - | - | - | - | - | - |
| 1ESW | 0.94 | 0.52 | 0.57 | 0.96 | 0.51 | 0.54 | 17 | 403 | 16 | 13 | 53/115 | 20/51 | 11/33 | 22/31 | - | - | - | - |
| 2O7I | 0.93 | 0.30 | 0.93 | 0.93 | 0.51 | 0.45 | 14 | 449 | 33 | 1 | 52/64 | 52/64 | - | - | - | - | - | - |
| 1WU5 | 0.93 | 0.27 | 1.00 | 0.92 | 0.50 | 0.43 | 9 | 293 | 24 | 0 | 31/33 | 31/33 | - | - | - | - | - | - |
| 1XEZ | 0.96 | 0.32 | 0.82 | 0.97 | 0.50 | 0.46 | 9 | 553 | 19 | 2 | 22/32 | 22/32 | - | - | - | - | - | - |
| 2HS3 | 0.96 | 0.39 | 0.69 | 0.97 | 0.50 | 0.50 | 11 | 494 | 17 | 5 | 16/33 | 16/33 | - | - | - | - | - | - |
| 1E3Z | 0.94 | 0.41 | 0.67 | 0.95 | 0.49 | 0.51 | 14 | 391 | 20 | 7 | 55/75 | 17/28 | 21/26 | 17/21 | - | - | - | - |
| 1M2J | 0.98 | 0.50 | 0.50 | 0.99 | 0.49 | 0.50 | 3 | 228 | 3 | 3 | 15/33 | 15/33 | - | - | - | - | - | - |
| 1QKQ | 0.91 | 0.33 | 0.83 | 0.92 | 0.49 | 0.48 | 5 | 109 | 10 | 1 | 19/29 | 19/29 | - | - | - | - | - | - |
| 1X9D | 0.94 | 0.41 | 0.67 | 0.95 | 0.49 | 0.51 | 12 | 326 | 17 | 6 | 30/52 | 30/52 | - | - | - | - | - | - |
| 1ZU0 | 0.94 | 0.36 | 0.74 | 0.94 | 0.49 | 0.48 | 14 | 423 | 25 | 5 | 54/82 | 54/82 | - | - | - | - | - | - |
| 4PFK | 0.97 | 0.57 | 0.44 | 0.99 | 0.49 | 0.50 | 4 | 255 | 3 | 5 | 13/32 | 13/32 | - | - | - | - | - | - |
| 1X1J | 0.95 | 0.24 | 1.00 | 0.95 | 0.48 | 0.39 | 11 | 624 | 35 | 0 | 37/38 | 37/38 | - | - | - | - | - | - |
| 2IHJ | 0.94 | 0.44 | 0.60 | 0.96 | 0.48 | 0.51 | 12 | 319 | 15 | 8 | 29/58 | 29/58 | - | - | - | - | - | - |
| 2WNB | 0.95 | 0.43 | 0.60 | 0.97 | 0.48 | 0.50 | 6 | 234 | 8 | 4 | 17/38 | 17/38 | - | - | - | - | - | - |
| 3C2V | 0.95 | 0.46 | 0.55 | 0.97 | 0.48 | 0.50 | 6 | 236 | 7 | 5 | 10/24 | 10/24 | - | - | - | - | - | - |
| 3CZN | 0.97 | 0.44 | 0.55 | 0.98 | 0.48 | 0.49 | 16 | 884 | 20 | 13 | 56/101 | 54/69 | 2/32 | - | - | - | - | - |
| 3D1R | 0.96 | 0.55 | 0.46 | 0.98 | 0.48 | 0.50 | 6 | 268 | 5 | 7 | 12/37 | 12/37 | - | - | - | - | - | - |
| 1MXD | 0.89 | 0.60 | 0.48 | 0.95 | 0.47 | 0.53 | 24 | 300 | 16 | 26 | 103/212 | 23/45 | 6/57 | 37/48 | 12/26 | 25/36 | - | - |
| 1UXX | 0.90 | 1.00 | 0.25 | 1.00 | 0.47 | 0.40 | 4 | 100 | 0 | 12 | 20/70 | 4/24 | 16/46 | - | - | - | - | - |
| 2D7R | 0.94 | 0.39 | 0.64 | 0.95 | 0.47 | 0.48 | 14 | 454 | 22 | 8 | 40/71 | 0/25 | 40/46 | - | - | - | - | - |
| 3II1 | 0.95 | 0.24 | 1.00 | 0.95 | 0.47 | 0.38 | 8 | 446 | 26 | 0 | 29/31 | 29/31 | - | - | - | - | - | - |
| 1JC9 | 0.93 | 0.40 | 0.60 | 0.95 | 0.46 | 0.48 | 6 | 179 | 9 | 4 | 22/31 | 22/31 | - | - | - | - | - | - |
| 1JZS | 0.98 | 0.50 | 0.44 | 0.99 | 0.46 | 0.47 | 7 | 751 | 7 | 9 | 15/42 | 15/42 | - | - | - | - | - | - |
| 1OH4 | 0.92 | 0.55 | 0.46 | 0.97 | 0.46 | 0.50 | 6 | 138 | 5 | 7 | 31/57 | 25/37 | 6/20 | - | - | - | - | - |
| 1QNR | 0.96 | 0.31 | 0.71 | 0.96 | 0.46 | 0.44 | 5 | 286 | 11 | 2 | 25/33 | 25/33 | - | - | - | - | - | - |
| 2D3N | 0.89 | 0.70 | 0.38 | 0.97 | 0.46 | 0.49 | 23 | 360 | 10 | 38 | 78/248 | 20/71 | 35/53 | 0/19 | 13/22 | 0/31 | 10/42 | 0/10 |
| 2JH7 | 0.98 | 0.67 | 0.33 | 1.00 | 0.46 | 0.44 | 2 | 191 | 1 | 4 | 4/27 | 4/27 | - | - | - | - | - | - |
| 3INA | 0.94 | 0.78 | 0.29 | 0.99 | 0.46 | 0.42 | 7 | 311 | 2 | 17 | 21/87 | 11/49 | 10/38 | - | - | - | - | - |
| 1G94 | 0.95 | 0.38 | 0.60 | 0.96 | 0.45 | 0.46 | 9 | 384 | 15 | 6 | 32/57 | 32/57 | - | - | - | - | - | - |
| 1OT2 | 0.92 | 0.78 | 0.29 | 0.99 | 0.45 | 0.42 | 18 | 532 | 5 | 44 | 65/231 | 1/51 | 46/60 | 0/29 | 18/32 | 0/36 | 0/23 | - |
| 1V7X | 0.96 | 0.28 | 0.77 | 0.96 | 0.45 | 0.41 | 10 | 647 | 26 | 3 | 35/50 | 35/50 | - | - | - | - | - | - |
| 2GQU | 0.95 | 0.80 | 0.27 | 1.00 | 0.45 | 0.40 | 4 | 231 | 1 | 11 | 10/52 | 6/39 | 4/13 | - | - | - | - | - |
| 2VUZ | 0.88 | 0.50 | 0.54 | 0.93 | 0.45 | 0.52 | 7 | 91 | 7 | 6 | 18/49 | 12/39 | 6/10 | - | - | - | - | - |
| 1E7Y | 0.98 | 0.50 | 0.40 | 0.99 | 0.44 | 0.44 | 4 | 438 | 4 | 6 | 8/25 | 8/25 | - | - | - | - | - | - |
| 1FFY | 0.97 | 0.31 | 0.67 | 0.97 | 0.44 | 0.43 | 10 | 838 | 22 | 5 | 36/50 | 36/50 | - | - | - | - | - | - |
| 1HV6 | 0.95 | 0.37 | 0.58 | 0.96 | 0.44 | 0.45 | 7 | 288 | 12 | 5 | 21/43 | 19/27 | 2/16 | - | - | - | - | - |
| 1OGO | 0.97 | 0.35 | 0.58 | 0.98 | 0.44 | 0.44 | 7 | 499 | 13 | 5 | 23/40 | 23/40 | - | - | - | - | - | - |
| 1V0C | 0.92 | 0.55 | 0.43 | 0.97 | 0.44 | 0.48 | 6 | 145 | 5 | 8 | 33/73 | 33/73 | - | - | - | - | - | - |
| 1SB8 | 0.95 | 0.54 | 0.39 | 0.98 | 0.43 | 0.45 | 7 | 283 | 6 | 11 | 21/66 | 21/66 | - | - | - | - | - | - |
| 1U7G | 0.97 | 0.50 | 0.40 | 0.99 | 0.43 | 0.44 | 4 | 311 | 4 | 6 | 12/44 | 12/44 | - | - | - | - | - | - |
| 2IHO | 0.91 | 0.44 | 0.52 | 0.94 | 0.43 | 0.48 | 11 | 236 | 14 | 10 | 34/96 | 18/54 | 16/42 | - | - | - | - | - |
| 2QZ2 | 0.87 | 0.55 | 0.46 | 0.94 | 0.43 | 0.50 | 11 | 133 | 9 | 13 | 29/87 | 29/44 | 0/43 | - | - | - | - | - |
| 2Z4T | 0.96 | 0.26 | 0.75 | 0.96 | 0.43 | 0.39 | 6 | 441 | 17 | 2 | 24/33 | 24/33 | - | - | - | - | - | - |
| 1FA2 | 0.93 | 0.28 | 0.71 | 0.94 | 0.42 | 0.40 | 10 | 418 | 26 | 4 | 21/38 | 21/38 | - | - | - | - | - | - |
| 1MWE | 0.93 | 0.59 | 0.35 | 0.98 | 0.42 | 0.44 | 10 | 325 | 7 | 19 | 27/85 | 27/47 | 0/38 | - | - | - | - | - |
| 1PYY | 0.98 | 0.50 | 0.36 | 0.99 | 0.42 | 0.42 | 4 | 540 | 4 | 7 | 12/45 | 1/23 | 11/22 | - | - | - | - | - |
| 2F6D | 0.95 | 0.53 | 0.38 | 0.98 | 0.42 | 0.44 | 9 | 420 | 8 | 15 | 34/97 | 34/54 | 0/43 | - | - | - | - | - |
| 2GJP | 0.91 | 0.47 | 0.47 | 0.95 | 0.42 | 0.47 | 17 | 361 | 19 | 19 | 60/150 | 7/28 | 0/30 | 23/40 | 4/20 | 10/11 | 16/21 | - |
| 2POQ | 0.95 | 0.31 | 0.63 | 0.96 | 0.42 | 0.42 | 5 | 272 | 11 | 3 | 17/31 | 17/31 | - | - | - | - | - | - |
| 2QMJ | 0.96 | 0.27 | 0.71 | 0.97 | 0.42 | 0.39 | 10 | 746 | 27 | 4 | 28/37 | 28/37 | - | - | - | - | - | - |
| 2WQQ | 0.93 | 0.67 | 0.30 | 0.99 | 0.42 | 0.41 | 6 | 203 | 3 | 14 | 16/61 | 0/19 | 16/42 | - | - | - | - | - |
| 1U8X | 0.96 | 0.29 | 0.63 | 0.97 | 0.41 | 0.40 | 5 | 380 | 12 | 3 | 15/27 | 15/27 | - | - | - | - | - | - |
| 2BFQ | 0.94 | 0.75 | 0.25 | 0.99 | 0.41 | 0.38 | 3 | 157 | 1 | 9 | 7/42 | 7/42 | - | - | - | - | - | - |
| 3ABX | 0.92 | 0.42 | 0.48 | 0.95 | 0.41 | 0.45 | 11 | 300 | 15 | 12 | 52/89 | 34/51 | 18/38 | - | - | - | - | - |
| 1RP8 | 0.90 | 0.61 | 0.33 | 0.97 | 0.40 | 0.42 | 14 | 322 | 9 | 29 | 44/178 | 0/54 | 0/36 | 43/66 | 1/22 | - | - | - |
| 1UMI | 0.97 | 1.00 | 0.17 | 1.00 | 0.40 | 0.29 | 1 | 159 | 0 | 5 | 3/35 | 3/35 | - | - | - | - | - | - |
| 2QKX | 0.95 | 0.43 | 0.43 | 0.98 | 0.40 | 0.43 | 6 | 323 | 8 | 8 | 11/37 | 11/37 | - | - | - | - | - | - |
| 1TYW | 0.95 | 0.41 | 0.43 | 0.97 | 0.39 | 0.42 | 9 | 478 | 13 | 12 | 29/72 | 29/61 | 0/11 | - | - | - | - | - |
| 2FST | 0.95 | 0.80 | 0.21 | 1.00 | 0.39 | 0.33 | 4 | 286 | 1 | 15 | 20/80 | 18/45 | 2/13 | 0/10 | 0/12 | - | - | - |
| 3EWR | 0.93 | 0.75 | 0.23 | 0.99 | 0.39 | 0.35 | 3 | 132 | 1 | 10 | 8/42 | 8/42 | - | - | - | - | - | - |
| 1LXM | 0.95 | 0.30 | 0.55 | 0.97 | 0.38 | 0.39 | 11 | 708 | 26 | 9 | 38/76 | 12/13 | 26/47 | 0/16 | - | - | - | - |
| 1YW1 | 0.98 | 1.00 | 0.14 | 1.00 | 0.38 | 0.25 | 1 | 351 | 0 | 6 | 2/26 | 2/26 | - | - | - | - | - | - |
| 2DWP | 0.92 | 0.38 | 0.48 | 0.95 | 0.38 | 0.42 | 12 | 354 | 20 | 13 | 26/68 | 12/33 | 14/35 | - | - | - | - | - |
| 2JHL | 0.95 | 0.43 | 0.38 | 0.98 | 0.38 | 0.40 | 3 | 180 | 4 | 5 | 8/27 | 8/27 | - | - | - | - | - | - |
| 3GD9 | 0.95 | 0.56 | 0.29 | 0.99 | 0.38 | 0.39 | 5 | 321 | 4 | 12 | 13/55 | 13/55 | - | - | - | - | - | - |
| 1FA9 | 0.95 | 0.19 | 0.80 | 0.96 | 0.37 | 0.30 | 8 | 737 | 35 | 2 | 25/29 | 25/29 | - | - | - | - | - | - |
| 1GJW | 0.94 | 0.38 | 0.42 | 0.97 | 0.37 | 0.40 | 11 | 546 | 18 | 15 | 37/87 | 20/67 | 17/20 | - | - | - | - | - |
| 2JAF | 0.97 | 1.00 | 0.14 | 1.00 | 0.37 | 0.25 | 1 | 221 | 0 | 6 | 9/28 | 9/28 | - | - | - | - | - | - |
| 2UUI | 0.96 | 1.00 | 0.14 | 1.00 | 0.37 | 0.25 | 1 | 136 | 0 | 6 | 0/23 | 0/23 | - | - | - | - | - | - |
| 1DLJ | 0.95 | 0.26 | 0.56 | 0.96 | 0.36 | 0.36 | 5 | 342 | 14 | 4 | 11/30 | 11/30 | - | - | - | - | - | - |
| 1J39 | 0.96 | 0.36 | 0.40 | 0.98 | 0.36 | 0.38 | 4 | 301 | 7 | 6 | 5/31 | 5/31 | - | - | - | - | - | - |
| 1L8T | 0.95 | 0.44 | 0.33 | 0.98 | 0.36 | 0.38 | 4 | 234 | 5 | 8 | 12/60 | 12/60 | - | - | - | - | - | - |
| 1MOR | 0.95 | 0.39 | 0.39 | 0.97 | 0.36 | 0.39 | 5 | 298 | 8 | 8 | 13/39 | 13/39 | - | - | - | - | - | - |
| 1GYM | 0.95 | 0.23 | 0.60 | 0.96 | 0.35 | 0.33 | 3 | 246 | 10 | 2 | 16/23 | 16/23 | - | - | - | - | - | - |
| 1SLY | 0.96 | 0.30 | 0.46 | 0.97 | 0.35 | 0.36 | 6 | 530 | 14 | 7 | 21/41 | 21/41 | - | - | - | - | - | - |
| 2PC8 | 0.94 | 0.33 | 0.44 | 0.96 | 0.35 | 0.38 | 7 | 326 | 14 | 9 | 25/57 | 25/25 | 0/32 | - | - | - | - | - |
| 1MFU | 0.91 | 0.46 | 0.32 | 0.97 | 0.34 | 0.38 | 12 | 382 | 14 | 25 | 49/155 | 22/24 | 0/21 | 0/30 | 11/22 | 0/30 | 16/28 | - |
| 1W3G | 0.95 | 0.75 | 0.17 | 1.00 | 0.34 | 0.27 | 3 | 282 | 1 | 15 | 8/73 | 8/42 | 0/31 | - | - | - | - | - |
| 2BHZ | 0.89 | 0.53 | 0.30 | 0.97 | 0.34 | 0.38 | 19 | 471 | 17 | 44 | 75/265 | 0/55 | 31/42 | 44/75 | 0/32 | 0/15 | 0/15 | 0/31 |
| 2C4D | 0.87 | 0.77 | 0.19 | 0.99 | 0.34 | 0.30 | 10 | 306 | 3 | 43 | 31/187 | 5/37 | 12/61 | 9/31 | 5/23 | 0/35 | - | - |
| 2HRL | 0.94 | 1.00 | 0.13 | 1.00 | 0.34 | 0.22 | 1 | 101 | 0 | 7 | 6/40 | 6/40 | - | - | - | - | - | - |
| 1L1R | 0.95 | 0.50 | 0.25 | 0.99 | 0.33 | 0.33 | 2 | 153 | 2 | 6 | 7/32 | 7/32 | - | - | - | - | - | - |
| 1V8R | 0.93 | 0.67 | 0.18 | 0.99 | 0.32 | 0.29 | 2 | 127 | 1 | 9 | 6/27 | 6/27 | - | - | - | - | - | - |
| 3FWL | 0.98 | 0.50 | 0.21 | 1.00 | 0.32 | 0.30 | 3 | 643 | 3 | 11 | 12/60 | 12/60 | - | - | - | - | - | - |
| 1YON | 0.96 | 0.67 | 0.15 | 1.00 | 0.31 | 0.25 | 2 | 253 | 1 | 11 | 3/43 | 0/14 | 3/29 | - | - | - | - | - |
| 2F5V | 0.92 | 0.17 | 0.67 | 0.93 | 0.31 | 0.28 | 8 | 474 | 38 | 4 | 20/31 | 20/31 | - | - | - | - | - | - |
| 2VXK | 0.94 | 1.00 | 0.10 | 1.00 | 0.31 | 0.18 | 1 | 146 | 0 | 9 | 2/37 | 2/37 | - | - | - | - | - | - |
| 3H2K | 0.95 | 0.50 | 0.22 | 0.99 | 0.31 | 0.31 | 4 | 329 | 4 | 14 | 5/51 | 5/40 | 0/11 | - | - | - | - | - |
| 1MW0 | 0.88 | 0.42 | 0.31 | 0.95 | 0.30 | 0.36 | 19 | 488 | 26 | 43 | 66/230 | 0/60 | 65/104 | 1/25 | 0/12 | 0/29 | - | - |
| 2W62 | 0.92 | 0.58 | 0.19 | 0.99 | 0.30 | 0.29 | 7 | 362 | 5 | 29 | 16/124 | 10/58 | 6/38 | 0/12 | 0/16 | - | - | - |
| 3CX4 | 0.89 | 0.48 | 0.26 | 0.97 | 0.30 | 0.34 | 12 | 359 | 13 | 34 | 46/191 | 0/38 | 0/50 | 15/63 | 31/40 | - | - | - |
| 2EWE | 0.91 | 0.33 | 0.35 | 0.95 | 0.29 | 0.34 | 7 | 273 | 14 | 13 | 20/50 | 20/50 | - | - | - | - | - | - |
| 3KH6 | 0.94 | 1.00 | 0.09 | 1.00 | 0.29 | 0.17 | 1 | 156 | 0 | 10 | 3/34 | 3/34 | - | - | - | - | - | - |
| 1UWF | 0.93 | 1.00 | 0.08 | 1.00 | 0.28 | 0.15 | 1 | 135 | 0 | 11 | 3/35 | 3/35 | - | - | - | - | - | - |
| 2VWG | 0.96 | 0.38 | 0.25 | 0.98 | 0.28 | 0.30 | 3 | 301 | 5 | 9 | 4/33 | 4/33 | - | - | - | - | - | - |
| 1GXO | 0.96 | 0.40 | 0.20 | 0.99 | 0.27 | 0.27 | 2 | 272 | 3 | 8 | 5/38 | 5/24 | 0/14 | - | - | - | - | - |
| 2VU9 | 0.92 | 0.21 | 0.46 | 0.94 | 0.27 | 0.29 | 6 | 357 | 23 | 7 | 22/57 | 22/57 | - | - | - | - | - | - |
| 1PIG | 0.89 | 0.37 | 0.27 | 0.95 | 0.26 | 0.31 | 11 | 390 | 19 | 30 | 36/148 | 0/32 | 17/36 | 19/28 | 0/33 | 0/19 | - | - |
| 1TZF | 0.96 | 0.29 | 0.29 | 0.98 | 0.26 | 0.29 | 2 | 215 | 5 | 5 | 8/25 | 8/25 | - | - | - | - | - | - |
| 2GR2 | 0.95 | 0.16 | 0.50 | 0.96 | 0.26 | 0.24 | 3 | 350 | 16 | 3 | 13/27 | 13/27 | - | - | - | - | - | - |
| 2UAG | 0.95 | 0.25 | 0.31 | 0.97 | 0.25 | 0.28 | 4 | 355 | 12 | 9 | 11/46 | 11/46 | - | - | - | - | - | - |
| 3CT5 | 0.90 | 1.00 | 0.07 | 1.00 | 0.25 | 0.13 | 1 | 111 | 0 | 13 | 3/52 | 3/52 | - | - | - | - | - | - |
| 1FWV | 0.92 | 0.29 | 0.29 | 0.96 | 0.24 | 0.29 | 2 | 116 | 5 | 5 | 12/31 | 12/31 | - | - | - | - | - | - |
| 1OA7 | 0.89 | 0.21 | 0.40 | 0.92 | 0.24 | 0.28 | 4 | 163 | 15 | 6 | 8/28 | 8/28 | - | - | - | - | - | - |
| 1SX6 | 0.96 | 0.50 | 0.13 | 1.00 | 0.24 | 0.20 | 1 | 187 | 1 | 7 | 12/34 | 12/34 | - | - | - | - | - | - |
| 3BIF | 0.96 | 0.30 | 0.23 | 0.98 | 0.24 | 0.26 | 3 | 371 | 7 | 10 | 5/45 | 5/45 | - | - | - | - | - | - |
| 3F3E | 0.93 | 0.32 | 0.24 | 0.97 | 0.24 | 0.27 | 6 | 399 | 13 | 19 | 18/81 | 8/36 | 10/45 | - | - | - | - | - |
| 1Q0Z | 0.92 | 0.22 | 0.33 | 0.95 | 0.23 | 0.27 | 4 | 256 | 14 | 8 | 8/36 | 8/36 | - | - | - | - | - | - |
| 3H1Y | 0.95 | 0.23 | 0.27 | 0.97 | 0.23 | 0.25 | 3 | 332 | 10 | 8 | 9/44 | 9/44 | - | - | - | - | - | - |
| 3HN1 | 0.94 | 0.25 | 0.25 | 0.97 | 0.22 | 0.25 | 4 | 388 | 12 | 12 | 21/63 | 21/52 | 0/11 | - | - | - | - | - |
| 5ADH | 0.94 | 0.23 | 0.27 | 0.97 | 0.22 | 0.25 | 3 | 300 | 10 | 8 | 6/33 | 6/33 | - | - | - | - | - | - |
| 2C9E | 0.97 | 0.50 | 0.10 | 1.00 | 0.21 | 0.17 | 1 | 305 | 1 | 9 | 7/38 | 7/15 | 0/23 | - | - | - | - | - |
| 1AUA | 0.88 | 0.16 | 0.42 | 0.90 | 0.20 | 0.23 | 5 | 247 | 27 | 7 | 7/55 | 7/31 | 0/24 | - | - | - | - | - |
| 1ILD | 0.85 | 0.21 | 0.37 | 0.89 | 0.20 | 0.27 | 7 | 206 | 26 | 12 | 20/91 | 0/25 | 13/21 | 4/21 | 0/12 | 3/12 | - | - |
| 1N0U | 0.98 | 0.15 | 0.29 | 0.99 | 0.20 | 0.20 | 2 | 740 | 11 | 5 | 6/23 | 6/23 | - | - | - | - | - | - |
| 2J8F | 0.89 | 0.24 | 0.29 | 0.93 | 0.20 | 0.26 | 6 | 267 | 19 | 15 | 19/81 | 19/81 | - | - | - | - | - | - |
| 1OGQ | 0.94 | 0.40 | 0.12 | 0.99 | 0.19 | 0.18 | 2 | 272 | 3 | 15 | 6/60 | 6/22 | 0/24 | 0/14 | - | - | - | - |
| 1OD3 | 0.88 | 0.33 | 0.17 | 0.96 | 0.18 | 0.22 | 2 | 100 | 4 | 10 | 10/51 | 10/51 | - | - | - | - | - | - |
| 2C3E | 0.95 | 0.22 | 0.20 | 0.97 | 0.18 | 0.21 | 2 | 255 | 7 | 8 | 4/30 | 4/30 | - | - | - | - | - | - |
| 2P3K | 0.94 | 0.33 | 0.13 | 0.99 | 0.18 | 0.18 | 1 | 135 | 2 | 7 | 1/33 | 1/33 | - | - | - | - | - | - |
| 1GYE | 0.91 | 0.14 | 0.30 | 0.93 | 0.17 | 0.19 | 3 | 255 | 18 | 7 | 5/26 | 5/26 | - | - | - | - | - | - |
| 1OFL | 0.89 | 0.30 | 0.16 | 0.96 | 0.16 | 0.21 | 6 | 371 | 14 | 32 | 10/118 | 8/83 | 2/35 | - | - | - | - | - |
| 1BG9 | 0.92 | 0.12 | 0.30 | 0.94 | 0.15 | 0.17 | 3 | 339 | 23 | 7 | 19/56 | 7/33 | 12/23 | - | - | - | - | - |
| 2GUP | 0.92 | 0.33 | 0.10 | 0.98 | 0.15 | 0.15 | 2 | 238 | 4 | 18 | 2/73 | 0/31 | 2/42 | - | - | - | - | - |
| 2GEJ | 0.94 | 0.17 | 0.18 | 0.97 | 0.14 | 0.17 | 2 | 294 | 10 | 9 | 6/37 | 6/37 | - | - | - | - | - | - |
| 1QZ6 | 0.93 | 0.10 | 0.29 | 0.94 | 0.13 | 0.14 | 2 | 298 | 19 | 5 | 6/24 | 6/24 | - | - | - | - | - | - |
| 4GPB | 0.97 | 0.20 | 0.11 | 0.99 | 0.13 | 0.14 | 2 | 745 | 8 | 17 | 3/59 | 3/20 | 0/39 | - | - | - | - | - |
| 1LRJ | 0.92 | 0.22 | 0.11 | 0.98 | 0.12 | 0.15 | 2 | 277 | 7 | 16 | 3/57 | 0/36 | 3/21 | - | - | - | - | - |
| 1ZX5 | 0.91 | 0.09 | 0.29 | 0.92 | 0.12 | 0.13 | 2 | 246 | 21 | 5 | 6/32 | 6/32 | - | - | - | - | - | - |
| 1T10 | 0.96 | 0.09 | 0.10 | 0.98 | 0.08 | 0.10 | 1 | 488 | 10 | 9 | 1/25 | 1/25 | - | - | - | - | - | - |
| 1UZ0 | 0.82 | 0.17 | 0.15 | 0.91 | 0.06 | 0.16 | 2 | 96 | 10 | 11 | 6/41 | 6/41 | - | - | - | - | - | - |
| 1F31 | 0.97 | 0.03 | 0.11 | 0.97 | 0.05 | 0.05 | 1 | 1111 | 31 | 8 | 2/42 | 2/42 | - | - | - | - | - | - |
| 2WWU | 0.93 | 0.11 | 0.06 | 0.97 | 0.05 | 0.08 | 1 | 300 | 8 | 16 | 0/56 | 0/35 | 0/21 | - | - | - | - | - |
| 1TL2 | 0.71 | 0.40 | 0.03 | 0.98 | 0.04 | 0.06 | 2 | 153 | 3 | 62 | 6/213 | 0/43 | 0/31 | 0/44 | 0/40 | 0/10 | 6/45 | - |
| 2RH1 | 0.93 | 0.06 | 0.07 | 0.96 | 0.03 | 0.07 | 1 | 392 | 16 | 13 | 3/51 | 3/51 | - | - | - | - | - | - |
| 148L | 0.00 | 0.00 | 0.00 | 0.00 | 0.00 | 0.00 | 0 | 136 | 0 | 18 | 0 | 0 | - | - | - | - | - | - |
| 1DRK | 0.00 | 0.00 | 0.00 | 0.00 | 0.00 | 0.00 | 0 | 214 | 0 | 11 | 0 | 0 | - | - | - | - | - | - |
| 1ECY | 0.00 | 0.00 | 0.00 | 0.00 | 0.00 | 0.00 | 0 | 110 | 0 | 28 | 0 | 0 | - | - | - | - | - | - |
| 1IZ2 | 0.00 | 0.00 | 0.00 | 0.00 | 0.00 | 0.00 | 0 | 336 | 0 | 7 | 0 | 0 | - | - | - | - | - | - |
| 1J1M | 0.00 | 0.00 | 0.00 | 0.00 | 0.00 | 0.00 | 0 | 228 | 0 | 16 | 0 | 0 | - | - | - | - | - | - |
| 1J2Z | 0.00 | 0.00 | 0.00 | 0.00 | 0.00 | 0.00 | 0 | 227 | 0 | 11 | 0 | 0 | - | - | - | - | - | - |
| 1JPC | 0.00 | 0.00 | 0.00 | 0.00 | 0.00 | 0.00 | 0 | 74 | 0 | 26 | 0 | 0 | - | - | - | - | - | - |
| 1NF9 | 0.00 | 0.00 | 0.00 | 0.00 | 0.00 | 0.00 | 0 | 175 | 0 | 5 | 0 | 0 | - | - | - | - | - | - |
| 1NPL | 0.00 | 0.00 | 0.00 | 0.00 | 0.00 | 0.00 | 0 | 78 | 0 | 25 | 0 | 0 | - | - | - | - | - | - |
| 1QGI | 0.00 | 0.00 | 0.00 | 0.00 | 0.00 | 0.00 | 0 | 223 | 0 | 10 | 0 | 0 | - | - | - | - | - | - |
| 1TJY | 0.00 | 0.00 | 0.00 | 0.00 | 0.00 | 0.00 | 0 | 244 | 0 | 13 | 0 | 0 | - | - | - | - | - | - |
| 1VST | 0.00 | 0.00 | 0.00 | 0.00 | 0.00 | 0.00 | 0 | 170 | 0 | 9 | 0 | 0 | - | - | - | - | - | - |
| 2C56 | 0.00 | 0.00 | 0.00 | 0.00 | 0.00 | 0.00 | 0 | 196 | 0 | 9 | 0 | 0 | - | - | - | - | - | - |
| 2CCV | 0.00 | 0.00 | 0.00 | 0.00 | 0.00 | 0.00 | 0 | 87 | 0 | 7 | 0 | 0 | - | - | - | - | - | - |
| 2JCR | 0.00 | 0.00 | 0.00 | 0.00 | 0.00 | 0.00 | 0 | 121 | 0 | 15 | 0 | 0 | - | - | - | - | - | - |
| 2JG0 | 0.00 | 0.00 | 0.00 | 0.00 | 0.00 | 0.00 | 0 | 432 | 0 | 16 | 0 | 0 | - | - | - | - | - | - |
| 2QIA | 0.00 | 0.00 | 0.00 | 0.00 | 0.00 | 0.00 | 0 | 224 | 0 | 11 | 0 | 0 | - | - | - | - | - | - |
| 2RDG | 0.00 | 0.00 | 0.00 | 0.00 | 0.00 | 0.00 | 0 | 173 | 0 | 11 | 0 | 0 | - | - | - | - | - | - |
| 2V5T | 0.00 | 0.00 | 0.00 | 0.00 | 0.00 | 0.00 | 0 | 166 | 0 | 7 | 0 | 0 | - | - | - | - | - | - |
| 2Z8L | 0.00 | 0.00 | 0.00 | 0.00 | 0.00 | 0.00 | 0 | 173 | 0 | 11 | 0 | 0 | - | - | - | - | - | - |
| 2ZG3 | 0.00 | 0.00 | 0.00 | 0.00 | 0.00 | 0.00 | 0 | 193 | 0 | 8 | 0 | 0 | - | - | - | - | - | - |
| 3IID | 0.00 | 0.00 | 0.00 | 0.00 | 0.00 | 0.00 | 0 | 145 | 0 | 13 | 0 | 0 | - | - | - | - | - | - |
| 966C | 0.00 | 0.00 | 0.00 | 0.00 | 0.00 | 0.00 | 0 | 137 | 0 | 12 | 0 | 0 | - | - | - | - | - | - |
| 9ABP | 0.00 | 0.00 | 0.00 | 0.00 | 0.00 | 0.00 | 0 | 246 | 0 | 16 | 0 | 0 | - | - | - | - | - | - |
| 1EXA | 0.97 | 0.00 | 0.00 | 0.99 | -0.01 | 0.00 | 0 | 212 | 2 | 4 | 0/11 | 0/11 | - | - | - | - | - | - |
| 1OB2 | 0.93 | 0.00 | 0.00 | 1.00 | -0.01 | 0.00 | 0 | 343 | 1 | 24 | 0/84 | 0/42 | 0/42 | - | - | - | - | - |
| 1OFC | 0.96 | 0.00 | 0.00 | 1.00 | -0.01 | 0.00 | 0 | 222 | 1 | 8 | 0/31 | 0/20 | 0/11 | - | - | - | - | - |
| 1OPR | 0.96 | 0.00 | 0.00 | 1.00 | -0.01 | 0.00 | 0 | 185 | 1 | 7 | 0/21 | 0/21 | - | - | - | - | - | - |
| 1TAQ | 0.97 | 0.00 | 0.00 | 0.98 | -0.01 | 0.00 | 0 | 724 | 16 | 5 | 0/23 | 0/23 | - | - | - | - | - | - |
| 1YC9 | 0.98 | 0.00 | 0.00 | 1.00 | -0.01 | 0.00 | 0 | 391 | 1 | 9 | 0/31 | 0/31 | - | - | - | - | - | - |
| 2A0Z | 0.97 | 0.00 | 0.00 | 0.99 | -0.01 | 0.00 | 0 | 602 | 7 | 11 | 0/40 | 0/20 | 0/20 | - | - | - | - | - |
| 2FN8 | 0.95 | 0.00 | 0.00 | 1.00 | -0.01 | 0.00 | 0 | 230 | 1 | 11 | 0/34 | 0/34 | - | - | - | - | - | - |
| 2HHQ | 0.94 | 0.00 | 0.00 | 1.00 | -0.01 | 0.00 | 0 | 492 | 1 | 28 | 0/105 | 0/47 | 0/23 | 0/35 | - | - | - | - |
| 2VF5 | 0.96 | 0.00 | 0.00 | 1.00 | -0.01 | 0.00 | 0 | 313 | 1 | 12 | 0/37 | 0/37 | - | - | - | - | - | - |
| 2YVV | 0.96 | 0.00 | 0.00 | 1.00 | -0.01 | 0.00 | 0 | 318 | 1 | 11 | 0/27 | 0/14 | 0/13 | - | - | - | - | - |
| 2ZZL | 0.96 | 0.00 | 0.00 | 1.00 | -0.01 | 0.00 | 0 | 208 | 1 | 8 | 0/29 | 0/29 | - | - | - | - | - | - |
| 1HFU | 0.96 | 0.00 | 0.00 | 0.98 | -0.02 | 0.00 | 0 | 432 | 11 | 9 | 0/44 | 0/30 | 0/14 | - | - | - | - | - |
| 1J4N | 0.96 | 0.00 | 0.00 | 0.99 | -0.02 | 0.00 | 0 | 217 | 2 | 8 | 0/28 | 0/28 | - | - | - | - | - | - |
| 1PW5 | 0.96 | 0.00 | 0.00 | 0.99 | -0.02 | 0.00 | 0 | 225 | 3 | 6 | 0/30 | 0/30 | - | - | - | - | - | - |
| 1Q2P | 0.95 | 0.00 | 0.00 | 0.98 | -0.02 | 0.00 | 0 | 229 | 4 | 7 | 0/31 | 0/31 | - | - | - | - | - | - |
| 1TXZ | 0.93 | 0.00 | 0.00 | 0.99 | -0.02 | 0.00 | 0 | 211 | 2 | 14 | 0/46 | 0/46 | - | - | - | - | - | - |
| 1UX7 | 0.93 | 0.00 | 0.00 | 0.99 | -0.02 | 0.00 | 0 | 99 | 1 | 6 | 0/30 | 0/30 | - | - | - | - | - | - |
| 2BDM | 0.92 | 0.00 | 0.00 | 0.99 | -0.02 | 0.00 | 0 | 408 | 3 | 32 | 0/118 | 0/68 | 0/21 | 0/28 | - | - | - | - |
| 2EBH | 0.95 | 0.00 | 0.00 | 0.97 | -0.02 | 0.00 | 0 | 622 | 17 | 13 | 0/66 | 0/49 | 0/17 | - | - | - | - | - |
| 2EVU | 0.96 | 0.00 | 0.00 | 0.99 | -0.02 | 0.00 | 0 | 210 | 3 | 6 | 0/30 | 0/17 | 0/13 | - | - | - | - | - |
| 2F5T | 0.92 | 0.00 | 0.00 | 1.00 | -0.02 | 0.00 | 0 | 191 | 1 | 16 | 0/60 | 0/60 | - | - | - | - | - | - |
| 2GY5 | 0.95 | 0.00 | 0.00 | 0.98 | -0.02 | 0.00 | 0 | 390 | 7 | 12 | 0/41 | 0/13 | 0/11 | 0/17 | - | - | - | - |
| 2OBT | 0.97 | 0.00 | 0.00 | 0.99 | -0.02 | 0.00 | 0 | 278 | 4 | 6 | 0/21 | 0/21 | - | - | - | - | - | - |
| 2ZJ3 | 0.95 | 0.00 | 0.00 | 0.99 | -0.02 | 0.00 | 0 | 292 | 2 | 13 | 0/36 | 0/36 | - | - | - | - | - | - |
| 3CL5 | 0.95 | 0.00 | 0.00 | 0.98 | -0.02 | 0.00 | 0 | 312 | 8 | 7 | 0/29 | 0/29 | - | - | - | - | - | - |
| 3FHH | 0.95 | 0.00 | 0.00 | 0.96 | -0.02 | 0.00 | 0 | 554 | 25 | 6 | 0/25 | 0/25 | - | - | - | - | - | - |
| 3GZ9 | 0.95 | 0.00 | 0.00 | 0.98 | -0.02 | 0.00 | 0 | 230 | 6 | 5 | 0/23 | 0/23 | - | - | - | - | - | - |
| 3HD6 | 0.96 | 0.00 | 0.00 | 0.98 | -0.02 | 0.00 | 0 | 330 | 8 | 7 | 0/30 | 0/30 | - | - | - | - | - | - |
| 3ICV | 0.96 | 0.00 | 0.00 | 0.98 | -0.02 | 0.00 | 0 | 244 | 4 | 7 | 0/26 | 0/13 | 0/13 | - | - | - | - | - |
| 3IIU | 0.96 | 0.00 | 0.00 | 0.99 | -0.02 | 0.00 | 0 | 143 | 2 | 4 | 0/19 | 0/19 | - | - | - | - | - | - |
| 1A5Z | 0.94 | 0.00 | 0.00 | 0.98 | -0.03 | 0.00 | 0 | 262 | 6 | 10 | 0/42 | 0/22 | 0/20 | - | - | - | - | - |
| 1C4O | 0.93 | 0.00 | 0.00 | 0.95 | -0.03 | 0.00 | 0 | 420 | 23 | 8 | 0/16 | 0/16 | - | - | - | - | - | - |
| 1CPY | 0.95 | 0.00 | 0.00 | 0.97 | -0.03 | 0.00 | 0 | 336 | 10 | 9 | 0/29 | 0/13 | 0/16 | - | - | - | - | - |
| 1FI1 | 0.94 | 0.00 | 0.00 | 0.96 | -0.03 | 0.00 | 0 | 651 | 31 | 11 | 0/36 | 0/36 | - | - | - | - | - | - |
| 1J8R | 0.94 | 0.00 | 0.00 | 0.99 | -0.03 | 0.00 | 0 | 176 | 2 | 10 | 0/47 | 0/47 | - | - | - | - | - | - |
| 1KQR | 0.93 | 0.00 | 0.00 | 0.99 | -0.03 | 0.00 | 0 | 138 | 2 | 8 | 0/30 | 0/30 | - | - | - | - | - | - |
| 1KZN | 0.91 | 0.00 | 0.00 | 0.99 | -0.03 | 0.00 | 0 | 160 | 2 | 13 | 0/41 | 0/41 | - | - | - | - | - | - |
| 1SE3 | 0.93 | 0.00 | 0.00 | 0.97 | -0.03 | 0.00 | 0 | 188 | 5 | 9 | 0/34 | 0/34 | - | - | - | - | - | - |
| 1U65 | 0.93 | 0.00 | 0.00 | 0.95 | -0.03 | 0.00 | 0 | 441 | 25 | 8 | 0/36 | 0/36 | - | - | - | - | - | - |
| 1W6K | 0.93 | 0.00 | 0.00 | 0.94 | -0.03 | 0.00 | 0 | 587 | 35 | 10 | 0/49 | 0/25 | 0/24 | - | - | - | - | - |
| 1YLJ | 0.94 | 0.00 | 0.00 | 0.98 | -0.03 | 0.00 | 0 | 219 | 4 | 10 | 0/33 | 0/33 | - | - | - | - | - | - |
| 2BBH | 0.89 | 0.00 | 0.00 | 0.99 | -0.03 | 0.00 | 0 | 192 | 2 | 21 | 0/83 | 0/65 | 0/18 | - | - | - | - | - |
| 2C78 | 0.94 | 0.00 | 0.00 | 0.96 | -0.03 | 0.00 | 0 | 339 | 13 | 10 | 0/33 | 0/33 | - | - | - | - | - | - |
| 2CNE | 0.95 | 0.00 | 0.00 | 0.97 | -0.03 | 0.00 | 0 | 246 | 8 | 6 | 0/32 | 0/32 | - | - | - | - | - | - |
| 2HZH | 0.94 | 0.00 | 0.00 | 0.97 | -0.03 | 0.00 | 0 | 416 | 14 | 15 | 0/58 | 0/14 | 0/12 | 0/32 | - | - | - | - |
| 2O9G | 0.95 | 0.00 | 0.00 | 0.98 | -0.03 | 0.00 | 0 | 198 | 4 | 7 | 2/30 | 2/30 | - | - | - | - | - | - |
| 2R68 | 0.95 | 0.00 | 0.00 | 0.98 | -0.03 | 0.00 | 0 | 381 | 8 | 12 | 0/42 | 0/42 | - | - | - | - | - | - |
| 2VE0 | 0.95 | 0.00 | 0.00 | 0.98 | -0.03 | 0.00 | 0 | 425 | 11 | 12 | 0/46 | 0/36 | 0/10 | - | - | - | - | - |
| 2VK2 | 0.93 | 0.00 | 0.00 | 0.99 | -0.03 | 0.00 | 0 | 231 | 3 | 14 | 0/41 | 0/41 | - | - | - | - | - | - |
| 2W1P | 0.93 | 0.00 | 0.00 | 0.99 | -0.03 | 0.00 | 0 | 219 | 3 | 13 | 0/70 | 0/18 | 0/30 | 0/22 | - | - | - | - |
| 3A7B | 0.94 | 0.00 | 0.00 | 0.95 | -0.03 | 0.00 | 0 | 484 | 25 | 6 | 0/28 | 0/28 | - | - | - | - | - | - |
| 3B9Z | 0.94 | 0.00 | 0.00 | 0.96 | -0.03 | 0.00 | 0 | 314 | 13 | 7 | 0/23 | 0/12 | 0/11 | - | - | - | - | - |
| 3BKL | 0.92 | 0.00 | 0.00 | 0.93 | -0.03 | 0.00 | 0 | 482 | 39 | 6 | 0/23 | 0/23 | - | - | - | - | - | - |
| 3C9E | 0.93 | 0.00 | 0.00 | 0.99 | -0.03 | 0.00 | 0 | 175 | 2 | 12 | 0/45 | 0/45 | - | - | - | - | - | - |
| 3CR9 | 0.94 | 0.00 | 0.00 | 0.96 | -0.03 | 0.00 | 0 | 600 | 26 | 10 | 1/36 | 1/36 | - | - | - | - | - | - |
| 3D3H | 0.91 | 0.00 | 0.00 | 0.99 | -0.03 | 0.00 | 0 | 140 | 1 | 13 | 0/39 | 0/39 | - | - | - | - | - | - |
| 3DAS | 0.94 | 0.00 | 0.00 | 0.98 | -0.03 | 0.00 | 0 | 284 | 7 | 12 | 0/41 | 0/23 | 0/18 | - | - | - | - | - |
| 3FP0 | 0.94 | 0.00 | 0.00 | 0.96 | -0.03 | 0.00 | 0 | 415 | 17 | 8 | 0/26 | 0/26 | - | - | - | - | - | - |
| 3I2T | 0.95 | 0.00 | 0.00 | 0.97 | -0.03 | 0.00 | 0 | 460 | 14 | 12 | 0/38 | 0/27 | 0/11 | - | - | - | - | - |
| 3IFE | 0.94 | 0.00 | 0.00 | 0.97 | -0.03 | 0.00 | 0 | 360 | 10 | 12 | 0/40 | 0/19 | 0/21 | - | - | - | - | - |
| 1FE2 | 0.93 | 0.00 | 0.00 | 0.96 | -0.04 | 0.00 | 0 | 471 | 22 | 15 | 0/64 | 0/19 | 0/45 | - | - | - | - | - |
| 1GWM | 0.89 | 0.00 | 0.00 | 0.99 | -0.04 | 0.00 | 0 | 129 | 2 | 14 | 0/65 | 0/35 | 0/30 | - | - | - | - | - |
| 1LLC | 0.93 | 0.00 | 0.00 | 0.96 | -0.04 | 0.00 | 0 | 283 | 11 | 10 | 0/32 | 0/32 | - | - | - | - | - | - |
| 1OAF | 0.92 | 0.00 | 0.00 | 0.95 | -0.04 | 0.00 | 0 | 216 | 11 | 9 | 0/32 | 0/32 | - | - | - | - | - | - |
| 1P49 | 0.92 | 0.00 | 0.00 | 0.94 | -0.04 | 0.00 | 0 | 468 | 30 | 10 | 0/26 | 0/14 | 0/12 | - | - | - | - | - |
| 1UXT | 0.91 | 0.00 | 0.00 | 0.93 | -0.04 | 0.00 | 0 | 415 | 30 | 10 | 0/31 | 0/31 | - | - | - | - | - | - |
| 1UYQ | 0.91 | 0.00 | 0.00 | 0.93 | -0.04 | 0.00 | 0 | 362 | 27 | 8 | 0/29 | 0/29 | - | - | - | - | - | - |
| 1YMG | 0.93 | 0.00 | 0.00 | 0.97 | -0.04 | 0.00 | 0 | 195 | 7 | 7 | 3/34 | 3/19 | 0/15 | - | - | - | - | - |
| 2DWJ | 0.92 | 0.00 | 0.00 | 0.95 | -0.04 | 0.00 | 0 | 292 | 14 | 11 | 0/42 | 0/42 | - | - | - | - | - | - |
| 2E59 | 0.92 | 0.00 | 0.00 | 0.97 | -0.04 | 0.00 | 0 | 129 | 4 | 8 | 0/37 | 0/24 | 0/13 | - | - | - | - | - |
| 3C02 | 0.91 | 0.00 | 0.00 | 0.93 | -0.04 | 0.00 | 0 | 187 | 14 | 5 | 0/17 | 0/17 | - | - | - | - | - | - |
| 3DIV | 0.92 | 0.00 | 0.00 | 0.96 | -0.04 | 0.00 | 0 | 407 | 17 | 18 | 0/60 | 0/22 | 0/17 | 0/21 | - | - | - | - |
| 3HZS | 0.90 | 0.00 | 0.00 | 0.98 | -0.04 | 0.00 | 0 | 167 | 3 | 16 | 0/52 | 0/39 | 0/13 | - | - | - | - | - |
| 1EU1 | 0.90 | 0.00 | 0.00 | 0.92 | -0.05 | 0.00 | 0 | 625 | 52 | 17 | 0/47 | 0/24 | 0/23 | - | - | - | - | - |
| 1ODA | 0.90 | 0.00 | 0.00 | 0.96 | -0.05 | 0.00 | 0 | 100 | 4 | 7 | 0/35 | 0/35 | - | - | - | - | - | - |
| 2BGI | 0.88 | 0.00 | 0.00 | 0.98 | -0.05 | 0.00 | 0 | 199 | 4 | 24 | 0/83 | 0/27 | 0/56 | - | - | - | - | - |
| 2O0V | 0.90 | 0.00 | 0.00 | 0.96 | -0.05 | 0.00 | 0 | 318 | 15 | 20 | 0/58 | 0/37 | 0/21 | - | - | - | - | - |
| 3A6T | 0.91 | 0.00 | 0.00 | 0.95 | -0.05 | 0.00 | 0 | 103 | 5 | 5 | 0/29 | 0/29 | - | - | - | - | - | - |
| 3BKV | 0.89 | 0.00 | 0.00 | 0.97 | -0.05 | 0.00 | 0 | 224 | 7 | 22 | 0/68 | 0/68 | - | - | - | - | - | - |
| 3FUS | 0.90 | 0.00 | 0.00 | 0.93 | -0.05 | 0.00 | 0 | 272 | 22 | 10 | 0/48 | 0/33 | 0/15 | - | - | - | - | - |
| 1DL2 | 0.89 | 0.00 | 0.00 | 0.92 | -0.06 | 0.00 | 0 | 384 | 33 | 16 | 0/60 | 0/17 | 0/14 | 0/29 | - | - | - | - |
| 1FX8 | 0.89 | 0.00 | 0.00 | 0.96 | -0.06 | 0.00 | 0 | 202 | 8 | 18 | 0/59 | 0/39 | 0/20 | - | - | - | - | - |
| 2IC8 | 0.84 | 0.00 | 0.00 | 0.98 | -0.06 | 0.00 | 0 | 137 | 3 | 23 | 0/94 | 0/43 | 0/15 | 0/11 | 0/25 | - | - | - |
| 2V8L | 0.88 | 0.00 | 0.00 | 0.94 | -0.07 | 0.00 | 0 | 85 | 5 | 7 | 0/36 | 0/36 | - | - | - | - | - | - |
| Total | 0.95 | 0.50 | 0.52 | 0.97 | 0.49 | 0.51 | 4180 | 156882 | 4168 | 3819 |  |  |  |  |  |  |  |  |

**Table S2:** Ten-fold cross validation ANN prediction accuracy benchmarks on the S497 dataset. The dataset, the ten-fold cross validation, and the benchmark measurements have been described in the main text. Matthews correlation coefficient (MCC), F-score(Fsc), Accuracy(Acc), Precision(Pre), Sensitivity(Sen) and Specificity(Spe) are shown in Equations (4)~(9). TP, FP, TN, and FN are true positive, false positive, true negative, and false negative respectively. C1~C7 represent carbohydrate binding sites in each of the test proteins; different protein has different number of binding sites. In these columns, the number of the predicted true positive atoms is shown over the actual number of atoms involving in the binding site. Interactive examination of the prediction results for each of the proteins in the S497 dataset can be accessed from the web server: <http://ismblab.genomics.sinica.edu.tw/>> benchmark > protein-carbohydrate.
