# Supplementary material for: Prediction of Carbohydrate Binding Sites on Protein Surfaces with 3-Dimensional Probability Density Distributions of Interacting Atoms
Source: PLoS One. 2012 Jul 25;7(7):e40846. doi: 10.1371/journal.pone.0040846 (PMC3405063; doi:10.1371/journal.pone.0040846)
Supplement: Table S3 — Ten-fold cross validation SVM prediction accuracy benchmarks on the S497 dataset. The dataset, the ten-fold cross validation, and the benchmark measurements have been described in the main text. Matthews correlation coefficient (MCC), F-score(Fsc), Accuracy(Acc), Precision(Pre), Sensitivity(Sen) and Specificity(Spe) are shown in Equations (4)∼(9). TP, FP, TN, and FN are true positive, false positive, true negative, and false negative respectively. C1∼C7 represent carbohydrate binding sites in each of the test proteins; different protein has different number of binding sites. In these columns, the number of the predicted true positive atoms is shown over the actual number of atoms involving in the binding site. Interactive examination of the prediction results for each of the proteins in the S497 dataset can be accessed from the web server: http://ismblab.genomics.sinica.edu.tw/> benchmark > protein-carbohydrate. (DOC) [file pone.0040846.s006.doc]

**Table S3**

| **PDBID** | **Residue-based SVM prediction benchmarks of ten-fold cross validation on S497 dataset** | | | | | | | | | | | | | | | | | |
| --- | --- | --- | --- | --- | --- | --- | --- | --- | --- | --- | --- | --- | --- | --- | --- | --- | --- | --- |
|  | **Residues Level** | | | | | | | | | | **Predict positive atoms / Actual binding atoms** | | | | | | | |
|  | **Acc** | **Pre** | **Sen** | **Spe** | **MCC** | **Fsc** | **TP** | **TN** | **FP** | **FN** | **All** | **C1** | **C2** | **C3** | **C4** | **C5** | **C6** | **C7** |
| 2IT6 | 1 | 1 | 1 | 1 | 1 | 1 | 10 | 113 | 0 | 0 | 22/29 | 22/29 | - | - | - | - | - | - |
| 1MVQ | 1 | 1 | 0.9 | 1 | 0.95 | 0.95 | 9 | 197 | 0 | 1 | 28/33 | 28/33 | - | - | - | - | - | - |
| 2J7M | 0.99 | 1 | 0.89 | 1 | 0.94 | 0.94 | 8 | 126 | 0 | 1 | 31/43 | 31/43 | - | - | - | - | - | - |
| 2V72 | 0.99 | 0.88 | 1 | 0.99 | 0.93 | 0.93 | 7 | 122 | 1 | 0 | 24/30 | 24/30 | - | - | - | - | - | - |
| 2VX6 | 0.99 | 0.88 | 1 | 0.99 | 0.93 | 0.94 | 22 | 287 | 3 | 0 | 77/80 | 77/80 | - | - | - | - | - | - |
| 1RPJ | 0.99 | 0.86 | 1 | 0.99 | 0.92 | 0.92 | 12 | 227 | 2 | 0 | 31/33 | 31/33 | - | - | - | - | - | - |
| 2V4V | 0.99 | 1 | 0.86 | 1 | 0.92 | 0.92 | 6 | 110 | 0 | 1 | 32/35 | 32/35 | - | - | - | - | - | - |
| 1DIL | 0.99 | 0.89 | 0.94 | 0.99 | 0.91 | 0.91 | 16 | 311 | 2 | 1 | 35/40 | 35/40 | - | - | - | - | - | - |
| 2ZID | 0.99 | 0.83 | 1 | 0.99 | 0.91 | 0.91 | 19 | 466 | 4 | 0 | 56/60 | 56/60 | - | - | - | - | - | - |
| 2FMD | 0.99 | 0.91 | 0.91 | 1 | 0.9 | 0.91 | 10 | 188 | 1 | 1 | 30/39 | 30/39 | - | - | - | - | - | - |
| 2OVU | 0.99 | 1 | 0.82 | 1 | 0.9 | 0.9 | 9 | 197 | 0 | 2 | 31/40 | 31/40 | - | - | - | - | - | - |
| 2JE7 | 0.99 | 1 | 0.8 | 1 | 0.89 | 0.89 | 8 | 202 | 0 | 2 | 27/36 | 27/36 | - | - | - | - | - | - |
| 1C3N | 0.99 | 1 | 0.78 | 1 | 0.88 | 0.88 | 7 | 126 | 0 | 2 | 10/26 | 10/26 | - | - | - | - | - | - |
| 1ELJ | 0.99 | 0.83 | 0.95 | 0.99 | 0.88 | 0.88 | 19 | 302 | 4 | 1 | 68/78 | 68/78 | - | - | - | - | - | - |
| 1J8V | 0.99 | 0.77 | 1 | 0.99 | 0.88 | 0.87 | 17 | 499 | 5 | 0 | 59/60 | 59/60 | - | - | - | - | - | - |
| 1KWF | 0.98 | 0.95 | 0.83 | 1 | 0.88 | 0.89 | 20 | 288 | 1 | 4 | 78/97 | 39/51 | 29/35 | 10/11 | - | - | - | - |
| 1MG1 | 0.99 | 0.83 | 0.94 | 0.99 | 0.88 | 0.88 | 15 | 416 | 3 | 1 | 54/56 | 54/56 | - | - | - | - | - | - |
| 2GH9 | 0.99 | 0.89 | 0.89 | 0.99 | 0.88 | 0.89 | 16 | 317 | 2 | 2 | 65/74 | 65/74 | - | - | - | - | - | - |
| 1YOE | 0.99 | 0.77 | 1 | 0.98 | 0.87 | 0.87 | 13 | 247 | 4 | 0 | 30/31 | 30/31 | - | - | - | - | - | - |
| 2D6O | 0.99 | 0.88 | 0.88 | 0.99 | 0.87 | 0.88 | 7 | 135 | 1 | 1 | 26/31 | 26/31 | - | - | - | - | - | - |
| 2F0Z | 0.99 | 0.82 | 0.93 | 0.99 | 0.87 | 0.88 | 14 | 302 | 3 | 1 | 34/45 | 13/15 | 21/30 | - | - | - | - | - |
| 2VVS | 0.99 | 0.81 | 0.94 | 0.99 | 0.87 | 0.87 | 17 | 494 | 4 | 1 | 49/52 | 49/52 | - | - | - | - | - | - |
| 2YXS | 0.99 | 0.88 | 0.88 | 0.99 | 0.87 | 0.88 | 7 | 129 | 1 | 1 | 22/27 | 22/27 | - | - | - | - | - | - |
| 1UQY | 0.98 | 0.91 | 0.83 | 0.99 | 0.86 | 0.86 | 19 | 292 | 2 | 4 | 71/90 | 40/58 | 31/32 | - | - | - | - | - |
| 3GH5 | 0.99 | 0.81 | 0.93 | 0.99 | 0.86 | 0.87 | 13 | 426 | 3 | 1 | 47/48 | 47/48 | - | - | - | - | - | - |
| 1US2 | 0.99 | 0.84 | 0.88 | 0.99 | 0.85 | 0.86 | 21 | 443 | 4 | 3 | 71/88 | 28/42 | 43/46 | - | - | - | - | - |
| 2FVY | 0.98 | 0.74 | 1 | 0.98 | 0.85 | 0.85 | 14 | 248 | 5 | 0 | 48/49 | 48/49 | - | - | - | - | - | - |
| 2VGD | 0.97 | 0.79 | 0.95 | 0.97 | 0.85 | 0.86 | 19 | 152 | 5 | 1 | 71/82 | 71/82 | - | - | - | - | - | - |
| 2E2O | 0.98 | 0.78 | 0.93 | 0.98 | 0.84 | 0.85 | 14 | 238 | 4 | 1 | 39/41 | 39/41 | - | - | - | - | - | - |
| 2UVJ | 0.98 | 0.83 | 0.86 | 0.99 | 0.84 | 0.84 | 19 | 342 | 4 | 3 | 58/73 | 58/73 | - | - | - | - | - | - |
| 2W47 | 0.98 | 1 | 0.73 | 1 | 0.84 | 0.84 | 8 | 111 | 0 | 3 | 23/32 | 23/32 | - | - | - | - | - | - |
| 1QPK | 0.98 | 0.82 | 0.86 | 0.99 | 0.83 | 0.84 | 18 | 359 | 4 | 3 | 67/85 | 67/85 | - | - | - | - | - | - |
| 1UAS | 0.98 | 0.7 | 1 | 0.98 | 0.83 | 0.82 | 14 | 302 | 6 | 0 | 40/41 | 40/41 | - | - | - | - | - | - |
| 2DT3 | 0.98 | 0.82 | 0.88 | 0.98 | 0.83 | 0.85 | 22 | 299 | 5 | 3 | 85/119 | 61/62 | 17/28 | 7/29 | - | - | - | - |
| 2IT5 | 0.98 | 1 | 0.7 | 1 | 0.83 | 0.82 | 7 | 112 | 0 | 3 | 17/24 | 17/24 | - | - | - | - | - | - |
| 2VI0 | 0.97 | 0.75 | 0.96 | 0.97 | 0.83 | 0.84 | 21 | 210 | 7 | 1 | 79/88 | 79/88 | - | - | - | - | - | - |
| 1OUR | 0.97 | 1 | 0.7 | 1 | 0.82 | 0.82 | 7 | 92 | 0 | 3 | 18/29 | 18/29 | - | - | - | - | - | - |
| 1S0I | 0.99 | 0.85 | 0.81 | 0.99 | 0.82 | 0.83 | 17 | 535 | 3 | 4 | 49/64 | 49/64 | - | - | - | - | - | - |
| 2ZXT | 0.98 | 0.73 | 0.94 | 0.99 | 0.82 | 0.82 | 16 | 403 | 6 | 1 | 62/66 | 40/43 | 22/23 | - | - | - | - | - |
| 1K12 | 0.98 | 1 | 0.67 | 1 | 0.81 | 0.8 | 6 | 131 | 0 | 3 | 13/19 | 13/19 | - | - | - | - | - | - |
| 1KTC | 0.98 | 0.77 | 0.87 | 0.99 | 0.81 | 0.81 | 13 | 336 | 4 | 2 | 40/48 | 40/48 | - | - | - | - | - | - |
| 1Q33 | 0.98 | 0.73 | 0.92 | 0.98 | 0.81 | 0.82 | 11 | 253 | 4 | 1 | 28/33 | 28/33 | - | - | - | - | - | - |
| 1R87 | 0.97 | 0.68 | 1 | 0.97 | 0.81 | 0.81 | 19 | 297 | 9 | 0 | 74/75 | 74/75 | - | - | - | - | - | - |
| 2CIR | 0.98 | 0.67 | 1 | 0.98 | 0.81 | 0.8 | 10 | 240 | 5 | 0 | 31/33 | 31/33 | - | - | - | - | - | - |
| 2FNC | 0.97 | 0.67 | 1 | 0.97 | 0.81 | 0.8 | 18 | 310 | 9 | 0 | 72/76 | 72/76 | - | - | - | - | - | - |
| 2OSX | 0.98 | 0.71 | 0.94 | 0.99 | 0.81 | 0.81 | 15 | 395 | 6 | 1 | 59/65 | 32/33 | 27/32 | - | - | - | - | - |
| 2RJO | 0.98 | 0.72 | 0.93 | 0.98 | 0.81 | 0.81 | 13 | 281 | 5 | 1 | 42/47 | 42/47 | - | - | - | - | - | - |
| 2ZHN | 0.96 | 1 | 0.69 | 1 | 0.81 | 0.82 | 11 | 117 | 0 | 5 | 33/45 | 33/45 | - | - | - | - | - | - |
| 3H3K | 0.98 | 0.86 | 0.78 | 0.99 | 0.81 | 0.82 | 18 | 432 | 3 | 5 | 64/79 | 25/34 | 39/45 | - | - | - | - | - |
| 1RWG | 0.99 | 0.69 | 0.95 | 0.99 | 0.8 | 0.8 | 20 | 655 | 9 | 1 | 72/77 | 72/77 | - | - | - | - | - | - |
| 1T0O | 0.98 | 0.65 | 1 | 0.98 | 0.8 | 0.79 | 15 | 351 | 8 | 0 | 42/45 | 42/45 | - | - | - | - | - | - |
| 2IW1 | 0.98 | 0.77 | 0.85 | 0.98 | 0.8 | 0.81 | 17 | 300 | 5 | 3 | 35/53 | 13/22 | 17/19 | 5/12 | - | - | - | - |
| 3D4C | 0.98 | 0.65 | 1 | 0.98 | 0.8 | 0.79 | 15 | 427 | 8 | 0 | 60/61 | 60/61 | - | - | - | - | - | - |
| 3EHS | 0.98 | 0.65 | 1 | 0.98 | 0.8 | 0.79 | 15 | 410 | 8 | 0 | 60/62 | 46/48 | 14/14 | - | - | - | - | - |
| 3IM0 | 0.98 | 0.89 | 0.73 | 1 | 0.8 | 0.8 | 8 | 211 | 1 | 3 | 24/36 | 24/36 | - | - | - | - | - | - |
| 4A3H | 0.98 | 0.77 | 0.87 | 0.98 | 0.8 | 0.81 | 13 | 238 | 4 | 2 | 30/38 | 30/38 | - | - | - | - | - | - |
| 1B3Z | 0.97 | 0.94 | 0.68 | 1 | 0.79 | 0.79 | 15 | 242 | 1 | 7 | 51/88 | 0/24 | 51/64 | - | - | - | - | - |
| 1CEN | 0.98 | 0.63 | 1 | 0.98 | 0.79 | 0.77 | 12 | 268 | 7 | 0 | 51/54 | 51/54 | - | - | - | - | - | - |
| 1EHN | 0.97 | 0.96 | 0.67 | 1 | 0.79 | 0.79 | 26 | 440 | 1 | 13 | 92/157 | 69/107 | 23/30 | 0/20 | - | - | - | - |
| 1I82 | 0.97 | 0.77 | 0.83 | 0.98 | 0.79 | 0.8 | 10 | 157 | 3 | 2 | 39/45 | 39/45 | - | - | - | - | - | - |
| 1Y4C | 0.98 | 0.64 | 1 | 0.98 | 0.79 | 0.78 | 16 | 421 | 9 | 0 | 56/59 | 56/59 | - | - | - | - | - | - |
| 1Y65 | 0.98 | 0.8 | 0.8 | 0.99 | 0.79 | 0.8 | 12 | 269 | 3 | 3 | 25/33 | 25/33 | - | - | - | - | - | - |
| 2A2D | 0.98 | 0.68 | 0.94 | 0.98 | 0.79 | 0.79 | 17 | 364 | 8 | 1 | 47/53 | 47/53 | - | - | - | - | - | - |
| 2BOD | 0.97 | 0.82 | 0.78 | 0.99 | 0.79 | 0.8 | 14 | 219 | 3 | 4 | 50/69 | 50/69 | - | - | - | - | - | - |
| 3CUJ | 0.98 | 0.78 | 0.82 | 0.99 | 0.79 | 0.8 | 14 | 262 | 4 | 3 | 47/63 | 32/39 | 15/24 | - | - | - | - | - |
| 1FCV | 0.98 | 0.87 | 0.72 | 0.99 | 0.78 | 0.79 | 13 | 275 | 2 | 5 | 43/59 | 34/41 | 9/18 | - | - | - | - | - |
| 1PNF | 0.97 | 0.62 | 1 | 0.97 | 0.78 | 0.77 | 13 | 264 | 8 | 0 | 43/46 | 43/46 | - | - | - | - | - | - |
| 2B46 | 0.96 | 0.74 | 0.88 | 0.97 | 0.78 | 0.8 | 14 | 142 | 5 | 2 | 42/48 | 42/48 | - | - | - | - | - | - |
| 2CHH | 0.96 | 1 | 0.64 | 1 | 0.78 | 0.78 | 7 | 89 | 0 | 4 | 17/33 | 17/33 | - | - | - | - | - | - |
| 2IXB | 0.98 | 0.67 | 0.92 | 0.98 | 0.78 | 0.77 | 12 | 364 | 6 | 1 | 21/30 | 12/15 | 9/15 | - | - | - | - | - |
| 2VMG | 0.98 | 1 | 0.63 | 1 | 0.78 | 0.77 | 5 | 125 | 0 | 3 | 15/23 | 15/23 | - | - | - | - | - | - |
| 3B9A | 0.97 | 0.79 | 0.79 | 0.99 | 0.78 | 0.79 | 27 | 449 | 7 | 7 | 99/130 | 31/39 | 45/62 | 23/29 | - | - | - | - |
| 3G7W | 0.98 | 0.83 | 0.75 | 0.99 | 0.78 | 0.79 | 15 | 342 | 3 | 5 | 58/82 | 22/39 | 36/43 | - | - | - | - | - |
| 1EU8 | 0.97 | 0.86 | 0.72 | 0.99 | 0.77 | 0.78 | 18 | 328 | 3 | 7 | 49/67 | 41/53 | 8/14 | - | - | - | - | - |
| 1M03 | 0.98 | 0.6 | 1 | 0.98 | 0.77 | 0.75 | 15 | 422 | 10 | 0 | 52/53 | 52/53 | - | - | - | - | - | - |
| 1MQE | 0.98 | 0.78 | 0.78 | 0.99 | 0.77 | 0.78 | 7 | 162 | 2 | 2 | 14/24 | 14/24 | - | - | - | - | - | - |
| 2GH4 | 0.97 | 0.7 | 0.89 | 0.98 | 0.77 | 0.78 | 16 | 284 | 7 | 2 | 37/42 | 37/42 | - | - | - | - | - | - |
| 2P2V | 0.97 | 0.88 | 0.7 | 0.99 | 0.77 | 0.78 | 14 | 233 | 2 | 6 | 38/68 | 38/68 | - | - | - | - | - | - |
| 2WZF | 0.99 | 0.71 | 0.86 | 0.99 | 0.77 | 0.77 | 12 | 457 | 5 | 2 | 26/34 | 26/34 | - | - | - | - | - | - |
| 1IS3 | 0.96 | 0.75 | 0.82 | 0.98 | 0.76 | 0.78 | 9 | 115 | 3 | 2 | 31/37 | 31/37 | - | - | - | - | - | - |
| 1KJR | 0.97 | 0.78 | 0.78 | 0.98 | 0.76 | 0.78 | 7 | 120 | 2 | 2 | 24/35 | 14/25 | 10/10 | - | - | - | - | - |
| 1UA4 | 0.98 | 0.68 | 0.87 | 0.98 | 0.76 | 0.77 | 13 | 371 | 6 | 2 | 30/35 | 30/35 | - | - | - | - | - | - |
| 2J44 | 0.97 | 0.75 | 0.8 | 0.98 | 0.76 | 0.77 | 12 | 192 | 4 | 3 | 49/68 | 22/34 | 27/34 | - | - | - | - | - |
| 2VCE | 0.97 | 0.66 | 0.91 | 0.97 | 0.76 | 0.76 | 19 | 380 | 10 | 2 | 49/57 | 38/43 | 11/14 | - | - | - | - | - |
| 2Z1S | 0.97 | 0.75 | 0.81 | 0.98 | 0.76 | 0.78 | 21 | 367 | 7 | 5 | 51/69 | 12/20 | 35/37 | 4/12 | - | - | - | - |
| 3F5F | 0.98 | 0.63 | 0.94 | 0.99 | 0.76 | 0.75 | 15 | 576 | 9 | 1 | 63/66 | 63/66 | - | - | - | - | - | - |
| 3F9M | 0.98 | 0.68 | 0.88 | 0.98 | 0.76 | 0.77 | 15 | 369 | 7 | 2 | 35/40 | 35/40 | - | - | - | - | - | - |
| 1C1L | 0.96 | 0.67 | 0.89 | 0.97 | 0.75 | 0.76 | 8 | 116 | 4 | 1 | 28/33 | 28/33 | - | - | - | - | - | - |
| 1EUS | 0.98 | 0.79 | 0.73 | 0.99 | 0.75 | 0.76 | 11 | 299 | 3 | 4 | 30/42 | 30/42 | - | - | - | - | - | - |
| 1G1T | 0.97 | 1 | 0.58 | 1 | 0.75 | 0.74 | 7 | 131 | 0 | 5 | 13/25 | 13/25 | - | - | - | - | - | - |
| 1KNM | 0.94 | 0.77 | 0.81 | 0.96 | 0.75 | 0.79 | 13 | 97 | 4 | 3 | 42/58 | 20/26 | 22/32 | - | - | - | - | - |
| 1URG | 0.97 | 0.62 | 0.93 | 0.98 | 0.75 | 0.74 | 13 | 329 | 8 | 1 | 51/53 | 51/53 | - | - | - | - | - | - |
| 1W8N | 0.98 | 0.73 | 0.79 | 0.99 | 0.75 | 0.76 | 19 | 510 | 7 | 5 | 55/67 | 21/29 | 22/24 | 12/14 | - | - | - | - |
| 2FHF | 0.98 | 0.77 | 0.75 | 0.99 | 0.75 | 0.76 | 24 | 925 | 7 | 8 | 87/131 | 24/29 | 40/61 | 16/28 | 7/13 | - | - | - |
| 2HTQ | 0.98 | 0.85 | 0.69 | 0.99 | 0.75 | 0.76 | 11 | 339 | 2 | 5 | 28/52 | 28/52 | - | - | - | - | - | - |
| 2RJ7 | 0.96 | 0.69 | 0.86 | 0.97 | 0.75 | 0.77 | 18 | 240 | 8 | 3 | 57/73 | 33/45 | 24/28 | - | - | - | - | - |
| 2VGQ | 0.98 | 0.8 | 0.73 | 0.99 | 0.75 | 0.76 | 16 | 397 | 4 | 6 | 57/96 | 26/54 | 31/42 | - | - | - | - | - |
| 2VW1 | 0.98 | 0.64 | 0.89 | 0.98 | 0.75 | 0.74 | 16 | 569 | 9 | 2 | 35/42 | 35/42 | - | - | - | - | - | - |
| 2WMG | 0.98 | 0.73 | 0.8 | 0.99 | 0.75 | 0.76 | 16 | 461 | 6 | 4 | 50/63 | 30/33 | 20/30 | - | - | - | - | - |
| 3BYN | 0.98 | 0.74 | 0.78 | 0.99 | 0.75 | 0.76 | 14 | 367 | 5 | 4 | 46/59 | 46/59 | - | - | - | - | - | - |
| 3HKN | 0.97 | 0.87 | 0.68 | 0.99 | 0.75 | 0.77 | 13 | 213 | 2 | 6 | 33/49 | 33/49 | - | - | - | - | - | - |
| 1CZA | 0.97 | 0.8 | 0.71 | 0.99 | 0.74 | 0.76 | 37 | 728 | 9 | 15 | 90/133 | 34/55 | 9/12 | 47/66 | - | - | - | - |
| 1GZ9 | 0.97 | 0.73 | 0.79 | 0.98 | 0.74 | 0.76 | 11 | 202 | 4 | 3 | 28/43 | 28/43 | - | - | - | - | - | - |
| 1N1T | 0.98 | 0.68 | 0.81 | 0.99 | 0.74 | 0.74 | 13 | 534 | 6 | 3 | 37/46 | 37/46 | - | - | - | - | - | - |
| 1UU6 | 0.94 | 0.69 | 0.87 | 0.95 | 0.74 | 0.77 | 20 | 168 | 9 | 3 | 54/73 | 48/56 | 6/17 | - | - | - | - | - |
| 2QQW | 0.98 | 0.64 | 0.88 | 0.98 | 0.74 | 0.74 | 14 | 453 | 8 | 2 | 48/53 | 48/53 | - | - | - | - | - | - |
| 2YVW | 0.98 | 0.65 | 0.87 | 0.98 | 0.74 | 0.74 | 13 | 358 | 7 | 2 | 33/39 | 33/39 | - | - | - | - | - | - |
| 1E7Y | 0.99 | 0.78 | 0.7 | 1 | 0.73 | 0.74 | 7 | 440 | 2 | 3 | 15/25 | 15/25 | - | - | - | - | - | - |
| 1G9F | 0.98 | 1 | 0.55 | 1 | 0.73 | 0.71 | 6 | 196 | 0 | 5 | 11/31 | 11/31 | - | - | - | - | - | - |
| 1KWK | 0.98 | 0.55 | 1 | 0.98 | 0.73 | 0.71 | 12 | 571 | 10 | 0 | 37/37 | 37/37 | - | - | - | - | - | - |
| 1RQ5 | 0.97 | 0.69 | 0.8 | 0.98 | 0.73 | 0.74 | 20 | 489 | 9 | 5 | 81/95 | 81/95 | - | - | - | - | - | - |
| 1W0O | 0.97 | 0.61 | 0.9 | 0.97 | 0.73 | 0.73 | 27 | 641 | 17 | 3 | 63/84 | 24/27 | 18/23 | 21/34 | - | - | - | - |
| 2BF6 | 0.97 | 0.63 | 0.88 | 0.98 | 0.73 | 0.73 | 15 | 370 | 9 | 2 | 43/48 | 43/48 | - | - | - | - | - | - |
| 2E9M | 0.98 | 0.59 | 0.93 | 0.98 | 0.73 | 0.72 | 13 | 385 | 9 | 1 | 33/34 | 33/34 | - | - | - | - | - | - |
| 2HW1 | 0.97 | 0.65 | 0.85 | 0.98 | 0.73 | 0.73 | 11 | 247 | 6 | 2 | 28/36 | 28/36 | - | - | - | - | - | - |
| 2OWZ | 0.98 | 0.89 | 0.62 | 1 | 0.73 | 0.73 | 8 | 281 | 1 | 5 | 18/32 | 18/32 | - | - | - | - | - | - |
| 2QIA | 0.98 | 1 | 0.55 | 1 | 0.73 | 0.71 | 6 | 224 | 0 | 5 | 11/41 | 11/41 | - | - | - | - | - | - |
| 2WZG | 0.99 | 0.77 | 0.71 | 0.99 | 0.73 | 0.74 | 10 | 442 | 3 | 4 | 24/36 | 24/36 | - | - | - | - | - | - |
| 2YQS | 0.98 | 0.93 | 0.59 | 1 | 0.73 | 0.72 | 13 | 377 | 1 | 9 | 31/64 | 31/64 | - | - | - | - | - | - |
| 5CGT | 0.98 | 0.77 | 0.71 | 0.99 | 0.73 | 0.74 | 20 | 586 | 6 | 8 | 83/105 | 27/35 | 29/35 | 27/35 | - | - | - | - |
| 1FNZ | 0.97 | 1 | 0.54 | 1 | 0.72 | 0.7 | 7 | 196 | 0 | 6 | 21/41 | 21/41 | - | - | - | - | - | - |
| 1G97 | 0.97 | 0.88 | 0.61 | 1 | 0.72 | 0.72 | 14 | 383 | 2 | 9 | 24/56 | 24/56 | - | - | - | - | - | - |
| 2BVM | 0.98 | 0.57 | 0.92 | 0.98 | 0.72 | 0.71 | 12 | 472 | 9 | 1 | 26/33 | 26/33 | - | - | - | - | - | - |
| 2VCB | 0.98 | 0.53 | 1 | 0.98 | 0.72 | 0.69 | 18 | 765 | 16 | 0 | 47/49 | 47/49 | - | - | - | - | - | - |
| 2WHM | 0.96 | 0.54 | 1 | 0.96 | 0.72 | 0.7 | 14 | 300 | 12 | 0 | 41/41 | 41/41 | - | - | - | - | - | - |
| 3GNP | 0.97 | 0.54 | 1 | 0.97 | 0.72 | 0.7 | 14 | 400 | 12 | 0 | 39/39 | 39/39 | - | - | - | - | - | - |
| 4RHN | 0.95 | 1 | 0.55 | 1 | 0.72 | 0.71 | 6 | 91 | 0 | 5 | 12/29 | 12/29 | - | - | - | - | - | - |
| 7TAA | 0.97 | 0.84 | 0.64 | 0.99 | 0.72 | 0.73 | 16 | 406 | 3 | 9 | 53/93 | 39/59 | 14/34 | - | - | - | - | - |
| 1KCD | 0.98 | 0.82 | 0.64 | 0.99 | 0.71 | 0.72 | 9 | 284 | 2 | 5 | 20/42 | 18/31 | 2/11 | - | - | - | - | - |
| 1KQY | 0.95 | 0.79 | 0.68 | 0.98 | 0.71 | 0.73 | 15 | 205 | 4 | 7 | 46/86 | 24/53 | 18/19 | 4/14 | - | - | - | - |
| 1PMH | 0.97 | 0.8 | 0.67 | 0.99 | 0.71 | 0.73 | 8 | 158 | 2 | 4 | 41/54 | 41/54 | - | - | - | - | - | - |
| 1Q6D | 0.96 | 0.66 | 0.81 | 0.97 | 0.71 | 0.73 | 25 | 408 | 13 | 6 | 58/90 | 46/77 | 12/13 | - | - | - | - | - |
| 1RKD | 0.97 | 0.58 | 0.92 | 0.97 | 0.71 | 0.71 | 11 | 250 | 8 | 1 | 26/33 | 26/33 | - | - | - | - | - | - |
| 1S5M | 0.97 | 0.52 | 1 | 0.97 | 0.71 | 0.68 | 13 | 329 | 12 | 0 | 38/39 | 38/39 | - | - | - | - | - | - |
| 1SLI | 0.98 | 0.59 | 0.9 | 0.98 | 0.71 | 0.71 | 17 | 592 | 12 | 2 | 34/40 | 34/40 | - | - | - | - | - | - |
| 2AEZ | 0.98 | 0.56 | 0.93 | 0.98 | 0.71 | 0.7 | 14 | 455 | 11 | 1 | 44/46 | 44/46 | - | - | - | - | - | - |
| 2OEG | 0.99 | 0.89 | 0.57 | 1 | 0.71 | 0.7 | 8 | 440 | 1 | 6 | 19/39 | 19/39 | - | - | - | - | - | - |
| 1BDG | 0.97 | 0.6 | 0.86 | 0.98 | 0.7 | 0.71 | 12 | 337 | 8 | 2 | 32/36 | 32/36 | - | - | - | - | - | - |
| 1F9D | 0.95 | 0.69 | 0.76 | 0.97 | 0.7 | 0.72 | 35 | 486 | 16 | 11 | 131/169 | 96/122 | 35/47 | - | - | - | - | - |
| 1UP2 | 0.96 | 0.7 | 0.74 | 0.98 | 0.7 | 0.72 | 14 | 236 | 6 | 5 | 48/65 | 48/65 | - | - | - | - | - | - |
| 1Y9G | 0.98 | 0.5 | 1 | 0.98 | 0.7 | 0.67 | 11 | 443 | 11 | 0 | 38/39 | 38/39 | - | - | - | - | - | - |
| 2DEJ | 0.97 | 0.71 | 0.71 | 0.99 | 0.7 | 0.71 | 10 | 273 | 4 | 4 | 28/40 | 28/40 | - | - | - | - | - | - |
| 2WAO | 0.97 | 0.85 | 0.61 | 0.99 | 0.7 | 0.71 | 11 | 269 | 2 | 7 | 44/79 | 44/79 | - | - | - | - | - | - |
| 3JUL | 0.98 | 1 | 0.5 | 1 | 0.7 | 0.67 | 6 | 258 | 0 | 6 | 13/31 | 13/31 | - | - | - | - | - | - |
| 1EOM | 0.95 | 0.81 | 0.62 | 0.99 | 0.69 | 0.7 | 13 | 213 | 3 | 8 | 39/72 | 37/50 | 2/22 | - | - | - | - | - |
| 1V03 | 0.97 | 0.57 | 0.87 | 0.98 | 0.69 | 0.68 | 13 | 403 | 10 | 2 | 39/41 | 39/41 | - | - | - | - | - | - |
| 2WRA | 0.96 | 1 | 0.5 | 1 | 0.69 | 0.67 | 5 | 102 | 0 | 5 | 14/39 | 14/39 | - | - | - | - | - | - |
| 2ZYN | 0.97 | 0.93 | 0.54 | 1 | 0.69 | 0.68 | 13 | 321 | 1 | 11 | 55/97 | 35/59 | 20/38 | - | - | - | - | - |
| 3FIZ | 0.96 | 0.5 | 1 | 0.96 | 0.69 | 0.67 | 15 | 367 | 15 | 0 | 33/34 | 33/34 | - | - | - | - | - | - |
| 1B1Y | 0.96 | 0.67 | 0.74 | 0.98 | 0.68 | 0.7 | 20 | 410 | 10 | 7 | 48/76 | 48/76 | - | - | - | - | - | - |
| 1E6X | 0.97 | 0.52 | 0.92 | 0.98 | 0.68 | 0.67 | 11 | 408 | 10 | 1 | 34/37 | 34/37 | - | - | - | - | - | - |
| 1GNY | 0.96 | 0.7 | 0.7 | 0.98 | 0.68 | 0.7 | 7 | 128 | 3 | 3 | 31/43 | 31/43 | - | - | - | - | - | - |
| 1HKK | 0.95 | 0.55 | 0.9 | 0.95 | 0.68 | 0.68 | 17 | 287 | 14 | 2 | 74/89 | 49/54 | 25/35 | - | - | - | - | - |
| 1OH4 | 0.96 | 0.8 | 0.62 | 0.99 | 0.68 | 0.7 | 8 | 141 | 2 | 5 | 33/57 | 25/37 | 8/20 | - | - | - | - | - |
| 1WD4 | 0.96 | 0.79 | 0.61 | 0.99 | 0.68 | 0.69 | 19 | 391 | 5 | 12 | 53/100 | 7/31 | 16/34 | 30/35 | - | - | - | - |
| 2CGL | 0.97 | 0.48 | 1 | 0.97 | 0.68 | 0.65 | 13 | 409 | 14 | 0 | 40/41 | 40/41 | - | - | - | - | - | - |
| 1INV | 0.97 | 0.67 | 0.71 | 0.98 | 0.67 | 0.69 | 12 | 336 | 6 | 5 | 35/50 | 35/50 | - | - | - | - | - | - |
| 1LU1 | 0.97 | 1 | 0.46 | 1 | 0.67 | 0.63 | 6 | 216 | 0 | 7 | 10/39 | 10/39 | - | - | - | - | - | - |
| 1QBB | 0.98 | 0.52 | 0.88 | 0.98 | 0.67 | 0.65 | 15 | 733 | 14 | 2 | 61/70 | 61/70 | - | - | - | - | - | - |
| 1QHO | 0.96 | 0.89 | 0.54 | 1 | 0.67 | 0.67 | 23 | 575 | 3 | 20 | 90/156 | 55/81 | 0/31 | 8/12 | 27/32 | - | - | - |
| 2C1Z | 0.96 | 0.54 | 0.88 | 0.97 | 0.67 | 0.67 | 15 | 373 | 13 | 2 | 41/59 | 21/39 | 20/20 | - | - | - | - | - |
| 3CA3 | 0.96 | 1 | 0.47 | 1 | 0.67 | 0.64 | 9 | 216 | 0 | 10 | 33/72 | 19/38 | 14/34 | - | - | - | - | - |
| 2HTW | 0.97 | 0.89 | 0.5 | 1 | 0.66 | 0.64 | 8 | 333 | 1 | 8 | 20/47 | 20/47 | - | - | - | - | - | - |
| 2W5O | 0.97 | 0.5 | 0.89 | 0.98 | 0.66 | 0.64 | 8 | 308 | 8 | 1 | 28/31 | 28/31 | - | - | - | - | - | - |
| 1GOQ | 0.95 | 0.87 | 0.52 | 0.99 | 0.65 | 0.65 | 13 | 238 | 2 | 12 | 49/96 | 49/74 | 0/22 | - | - | - | - | - |
| 1IUC | 0.93 | 0.65 | 0.73 | 0.95 | 0.65 | 0.69 | 22 | 249 | 12 | 8 | 61/84 | 21/30 | 40/54 | - | - | - | - | - |
| 1L8N | 0.97 | 0.57 | 0.76 | 0.98 | 0.65 | 0.65 | 16 | 560 | 12 | 5 | 65/76 | 65/76 | - | - | - | - | - | - |
| 1XC6 | 0.98 | 0.43 | 1 | 0.98 | 0.65 | 0.61 | 13 | 874 | 17 | 0 | 35/36 | 35/36 | - | - | - | - | - | - |
| 1ZDG | 0.96 | 0.5 | 0.9 | 0.96 | 0.65 | 0.64 | 9 | 216 | 9 | 1 | 24/27 | 24/27 | - | - | - | - | - | - |
| 2B4F | 0.95 | 0.82 | 0.56 | 0.99 | 0.65 | 0.67 | 18 | 304 | 4 | 14 | 69/125 | 37/54 | 18/29 | 0/23 | 14/19 | - | - | - |
| 2O7I | 0.96 | 0.44 | 1 | 0.96 | 0.65 | 0.61 | 15 | 463 | 19 | 0 | 59/64 | 59/64 | - | - | - | - | - | - |
| 3DM0 | 0.97 | 0.44 | 1 | 0.97 | 0.65 | 0.61 | 15 | 567 | 19 | 0 | 55/59 | 37/37 | 18/22 | - | - | - | - | - |
| 1BYH | 0.93 | 0.48 | 0.93 | 0.93 | 0.64 | 0.63 | 13 | 172 | 14 | 1 | 38/46 | 38/46 | - | - | - | - | - | - |
| 1G0C | 0.97 | 0.47 | 0.9 | 0.97 | 0.64 | 0.62 | 9 | 293 | 10 | 1 | 28/36 | 28/36 | - | - | - | - | - | - |
| 1HM2 | 0.97 | 0.48 | 0.88 | 0.97 | 0.64 | 0.62 | 14 | 570 | 15 | 2 | 63/73 | 63/73 | - | - | - | - | - | - |
| 1K1W | 0.97 | 0.46 | 0.92 | 0.97 | 0.64 | 0.62 | 12 | 534 | 14 | 1 | 33/40 | 33/40 | - | - | - | - | - | - |
| 1LMQ | 0.92 | 0.73 | 0.65 | 0.96 | 0.64 | 0.69 | 11 | 97 | 4 | 6 | 37/56 | 37/56 | - | - | - | - | - | - |
| 1LOH | 0.97 | 0.65 | 0.67 | 0.98 | 0.64 | 0.66 | 20 | 618 | 11 | 10 | 64/106 | 47/53 | 7/10 | 8/28 | 2/15 | - | - | - |
| 2DWP | 0.96 | 0.65 | 0.68 | 0.98 | 0.64 | 0.67 | 17 | 365 | 9 | 8 | 36/68 | 16/33 | 20/35 | - | - | - | - | - |
| 2JEN | 0.92 | 0.82 | 0.58 | 0.98 | 0.64 | 0.68 | 18 | 164 | 4 | 13 | 64/118 | 64/107 | 0/11 | - | - | - | - | - |
| 1LED | 0.96 | 0.8 | 0.53 | 0.99 | 0.63 | 0.64 | 8 | 206 | 2 | 7 | 29/64 | 29/64 | - | - | - | - | - | - |
| 1PIE | 0.97 | 0.69 | 0.6 | 0.99 | 0.63 | 0.64 | 9 | 319 | 4 | 6 | 23/40 | 23/40 | - | - | - | - | - | - |
| 2G3J | 0.96 | 0.58 | 0.73 | 0.97 | 0.63 | 0.65 | 11 | 251 | 8 | 4 | 44/63 | 44/63 | - | - | - | - | - | - |
| 2H44 | 0.97 | 0.56 | 0.75 | 0.98 | 0.63 | 0.64 | 9 | 282 | 7 | 3 | 23/34 | 23/34 | - | - | - | - | - | - |
| 3H4I | 0.96 | 0.6 | 0.71 | 0.98 | 0.63 | 0.65 | 12 | 333 | 8 | 5 | 32/56 | 32/56 | - | - | - | - | - | - |
| 1GAI | 0.97 | 0.53 | 0.77 | 0.98 | 0.62 | 0.63 | 10 | 408 | 9 | 3 | 33/55 | 33/55 | - | - | - | - | - | - |
| 1Y3P | 0.95 | 0.51 | 0.8 | 0.96 | 0.62 | 0.63 | 20 | 408 | 19 | 5 | 65/87 | 51/51 | 14/36 | - | - | - | - | - |
| 2GQU | 0.96 | 0.88 | 0.47 | 1 | 0.62 | 0.61 | 7 | 231 | 1 | 8 | 18/52 | 13/39 | 5/13 | - | - | - | - | - |
| 2VJJ | 0.96 | 0.71 | 0.57 | 0.98 | 0.62 | 0.64 | 20 | 492 | 8 | 15 | 51/108 | 32/40 | 13/54 | 6/14 | - | - | - | - |
| 3CZK | 0.96 | 0.43 | 0.94 | 0.96 | 0.62 | 0.59 | 17 | 527 | 23 | 1 | 49/56 | 49/56 | - | - | - | - | - | - |
| 3E6J | 0.95 | 0.6 | 0.69 | 0.97 | 0.62 | 0.64 | 9 | 187 | 6 | 4 | 41/53 | 41/53 | - | - | - | - | - | - |
| 1G9R | 0.93 | 0.43 | 0.93 | 0.93 | 0.61 | 0.59 | 13 | 228 | 17 | 1 | 34/39 | 34/39 | - | - | - | - | - | - |
| 1GZ1 | 0.94 | 0.6 | 0.69 | 0.96 | 0.61 | 0.64 | 18 | 286 | 12 | 8 | 67/97 | 60/87 | 7/10 | - | - | - | - | - |
| 2ACQ | 0.95 | 0.39 | 1 | 0.95 | 0.61 | 0.56 | 9 | 259 | 14 | 0 | 26/28 | 26/28 | - | - | - | - | - | - |
| 2C27 | 0.96 | 0.56 | 0.71 | 0.97 | 0.61 | 0.63 | 10 | 266 | 8 | 4 | 23/35 | 23/35 | - | - | - | - | - | - |
| 2EAE | 0.98 | 0.45 | 0.87 | 0.98 | 0.61 | 0.59 | 13 | 772 | 16 | 2 | 51/60 | 51/60 | - | - | - | - | - | - |
| 3BMW | 0.96 | 0.63 | 0.63 | 0.98 | 0.61 | 0.63 | 19 | 561 | 11 | 11 | 60/112 | 5/30 | 47/65 | 8/17 | - | - | - | - |
| 3CKQ | 0.97 | 0.5 | 0.78 | 0.97 | 0.61 | 0.61 | 7 | 254 | 7 | 2 | 21/33 | 11/18 | 10/15 | - | - | - | - | - |
| 1FFY | 0.98 | 0.5 | 0.73 | 0.99 | 0.6 | 0.6 | 11 | 849 | 11 | 4 | 32/50 | 32/50 | - | - | - | - | - | - |
| 1GUI | 0.92 | 0.63 | 0.67 | 0.95 | 0.6 | 0.65 | 10 | 124 | 6 | 5 | 46/61 | 37/40 | 9/21 | - | - | - | - | - |
| 1ITC | 0.93 | 0.93 | 0.42 | 1 | 0.6 | 0.58 | 25 | 417 | 2 | 34 | 74/198 | 53/90 | 13/34 | 0/45 | 8/29 | - | - | - |
| 1R6D | 0.95 | 0.46 | 0.85 | 0.95 | 0.6 | 0.6 | 11 | 266 | 13 | 2 | 17/24 | 17/24 | - | - | - | - | - | - |
| 1UA7 | 0.96 | 0.54 | 0.72 | 0.97 | 0.6 | 0.62 | 13 | 353 | 11 | 5 | 36/59 | 36/59 | - | - | - | - | - | - |
| 1UAE | 0.96 | 0.55 | 0.69 | 0.98 | 0.6 | 0.61 | 11 | 344 | 9 | 5 | 26/51 | 26/51 | - | - | - | - | - | - |
| 1V0C | 0.95 | 0.78 | 0.5 | 0.99 | 0.6 | 0.61 | 7 | 148 | 2 | 7 | 33/73 | 33/73 | - | - | - | - | - | - |
| 1E3Z | 0.95 | 0.52 | 0.71 | 0.97 | 0.59 | 0.6 | 15 | 397 | 14 | 6 | 50/75 | 14/28 | 19/26 | 17/21 | - | - | - | - |
| 1G94 | 0.96 | 0.5 | 0.73 | 0.97 | 0.59 | 0.6 | 11 | 388 | 11 | 4 | 30/57 | 30/57 | - | - | - | - | - | - |
| 1INW | 0.97 | 0.88 | 0.41 | 1 | 0.59 | 0.56 | 7 | 334 | 1 | 10 | 18/48 | 18/48 | - | - | - | - | - | - |
| 1Z45 | 0.97 | 0.59 | 0.62 | 0.99 | 0.59 | 0.61 | 13 | 573 | 9 | 8 | 33/63 | 0/26 | 33/37 | - | - | - | - | - |
| 2EQD | 0.96 | 0.59 | 0.64 | 0.98 | 0.59 | 0.62 | 16 | 422 | 11 | 9 | 56/101 | 14/30 | 32/41 | 10/30 | - | - | - | - |
| 2POQ | 0.96 | 0.41 | 0.88 | 0.97 | 0.59 | 0.56 | 7 | 273 | 10 | 1 | 20/31 | 20/31 | - | - | - | - | - | - |
| 3DJ4 | 0.97 | 0.83 | 0.44 | 1 | 0.59 | 0.57 | 10 | 403 | 2 | 13 | 24/60 | 24/60 | - | - | - | - | - | - |
| 1JC9 | 0.96 | 0.6 | 0.6 | 0.98 | 0.58 | 0.6 | 6 | 184 | 4 | 4 | 19/31 | 19/31 | - | - | - | - | - | - |
| 1Z4X | 0.96 | 0.52 | 0.69 | 0.98 | 0.58 | 0.6 | 11 | 383 | 10 | 5 | 31/47 | 25/36 | 6/11 | - | - | - | - | - |
| 2RL2 | 0.96 | 0.55 | 0.65 | 0.97 | 0.58 | 0.6 | 11 | 339 | 9 | 6 | 34/58 | 24/28 | 10/30 | - | - | - | - | - |
| 2W52 | 0.93 | 0.52 | 0.74 | 0.95 | 0.58 | 0.61 | 14 | 230 | 13 | 5 | 49/73 | 49/73 | - | - | - | - | - | - |
| 3C2V | 0.96 | 0.5 | 0.73 | 0.97 | 0.58 | 0.59 | 8 | 235 | 8 | 3 | 14/24 | 14/24 | - | - | - | - | - | - |
| 1GU3 | 0.94 | 0.83 | 0.42 | 0.99 | 0.57 | 0.56 | 5 | 125 | 1 | 7 | 25/53 | 25/53 | - | - | - | - | - | - |
| 1M2J | 0.98 | 0.5 | 0.67 | 0.98 | 0.57 | 0.57 | 4 | 227 | 4 | 2 | 17/33 | 17/33 | - | - | - | - | - | - |
| 1TJ4 | 0.94 | 0.6 | 0.6 | 0.97 | 0.57 | 0.6 | 9 | 176 | 6 | 6 | 18/48 | 18/48 | - | - | - | - | - | - |
| 1ULV | 0.98 | 0.42 | 0.79 | 0.98 | 0.57 | 0.55 | 11 | 896 | 15 | 3 | 34/43 | 34/43 | - | - | - | - | - | - |
| 1UMI | 0.98 | 1 | 0.33 | 1 | 0.57 | 0.5 | 2 | 159 | 0 | 4 | 8/35 | 8/35 | - | - | - | - | - | - |
| 2D0G | 0.94 | 0.79 | 0.46 | 0.99 | 0.57 | 0.58 | 26 | 509 | 7 | 30 | 96/230 | 5/50 | 15/44 | 7/24 | 41/49 | 17/22 | 11/27 | 0/14 |
| 2WHL | 0.94 | 0.39 | 0.9 | 0.94 | 0.57 | 0.55 | 9 | 222 | 14 | 1 | 38/49 | 38/49 | - | - | - | - | - | - |
| 1D0M | 0.94 | 0.73 | 0.48 | 0.98 | 0.56 | 0.58 | 11 | 248 | 4 | 12 | 34/81 | 2/32 | 32/49 | - | - | - | - | - |
| 1QKQ | 0.94 | 0.42 | 0.83 | 0.94 | 0.56 | 0.56 | 5 | 112 | 7 | 1 | 20/29 | 20/29 | - | - | - | - | - | - |
| 1URX | 0.9 | 0.63 | 0.59 | 0.95 | 0.56 | 0.61 | 19 | 207 | 11 | 13 | 77/107 | 24/48 | 53/59 | - | - | - | - | - |
| 2ZAA | 0.91 | 0.8 | 0.44 | 0.98 | 0.56 | 0.57 | 12 | 179 | 3 | 15 | 23/73 | 13/47 | 10/26 | - | - | - | - | - |
| 3B50 | 0.95 | 0.59 | 0.59 | 0.97 | 0.56 | 0.59 | 10 | 257 | 7 | 7 | 24/53 | 24/53 | - | - | - | - | - | - |
| 3C7G | 0.96 | 0.44 | 0.77 | 0.97 | 0.56 | 0.56 | 10 | 397 | 13 | 3 | 33/50 | 21/23 | 12/27 | - | - | - | - | - |
| 6CEL | 0.92 | 0.73 | 0.5 | 0.97 | 0.56 | 0.59 | 24 | 341 | 9 | 24 | 92/183 | 35/59 | 48/64 | 9/60 | - | - | - | - |
| 1CNQ | 0.94 | 0.73 | 0.46 | 0.99 | 0.55 | 0.56 | 11 | 273 | 4 | 13 | 26/62 | 26/37 | 0/25 | - | - | - | - | - |
| 1J84 | 0.96 | 0.8 | 0.4 | 0.99 | 0.55 | 0.53 | 4 | 147 | 1 | 6 | 19/38 | 15/27 | 4/11 | - | - | - | - | - |
| 2BS5 | 0.84 | 0.8 | 0.52 | 0.95 | 0.55 | 0.63 | 12 | 60 | 3 | 11 | 37/70 | 30/38 | 7/32 | - | - | - | - | - |
| 3BC9 | 0.94 | 0.65 | 0.51 | 0.98 | 0.55 | 0.57 | 20 | 485 | 11 | 19 | 87/178 | 21/42 | 36/42 | 25/44 | 5/50 | - | - | - |
| 3HL3 | 0.95 | 0.86 | 0.38 | 1 | 0.55 | 0.52 | 6 | 203 | 1 | 10 | 11/36 | 11/36 | - | - | - | - | - | - |
| 1A9T | 0.96 | 0.54 | 0.58 | 0.98 | 0.54 | 0.56 | 7 | 240 | 6 | 5 | 21/38 | 21/38 | - | - | - | - | - | - |
| 1P5G | 0.95 | 0.37 | 0.83 | 0.95 | 0.54 | 0.51 | 10 | 356 | 17 | 2 | 24/32 | 24/32 | - | - | - | - | - | - |
| 1V8R | 0.94 | 0.71 | 0.46 | 0.98 | 0.54 | 0.56 | 5 | 126 | 2 | 6 | 12/27 | 12/27 | - | - | - | - | - | - |
| 1WU5 | 0.94 | 0.31 | 1 | 0.94 | 0.54 | 0.47 | 9 | 297 | 20 | 0 | 31/33 | 31/33 | - | - | - | - | - | - |
| 1X9D | 0.95 | 0.52 | 0.61 | 0.97 | 0.54 | 0.56 | 11 | 333 | 10 | 7 | 23/52 | 23/52 | - | - | - | - | - | - |
| 2BS7 | 0.93 | 0.47 | 0.7 | 0.95 | 0.54 | 0.56 | 7 | 146 | 8 | 3 | 25/39 | 25/39 | - | - | - | - | - | - |
| 2VUZ | 0.91 | 0.64 | 0.54 | 0.96 | 0.54 | 0.58 | 7 | 94 | 4 | 6 | 18/49 | 12/39 | 6/10 | - | - | - | - | - |
| 3FAX | 0.97 | 0.33 | 0.9 | 0.97 | 0.54 | 0.49 | 9 | 670 | 18 | 1 | 32/41 | 32/41 | - | - | - | - | - | - |
| 1LZC | 0.91 | 1 | 0.31 | 1 | 0.53 | 0.48 | 5 | 103 | 0 | 11 | 19/54 | 19/54 | - | - | - | - | - | - |
| 1W9W | 0.92 | 0.75 | 0.43 | 0.98 | 0.53 | 0.55 | 6 | 103 | 2 | 8 | 30/59 | 12/41 | 18/18 | - | - | - | - | - |
| 1X1J | 0.96 | 0.29 | 1 | 0.96 | 0.53 | 0.45 | 11 | 632 | 27 | 0 | 38/38 | 38/38 | - | - | - | - | - | - |
| 1Z3W | 0.94 | 0.31 | 1 | 0.93 | 0.53 | 0.47 | 11 | 347 | 25 | 0 | 40/40 | 40/40 | - | - | - | - | - | - |
| 2JEQ | 0.94 | 0.5 | 0.63 | 0.96 | 0.53 | 0.56 | 12 | 292 | 12 | 7 | 42/71 | 37/52 | 5/19 | - | - | - | - | - |
| 2PHH | 0.96 | 0.33 | 0.88 | 0.96 | 0.53 | 0.48 | 7 | 345 | 14 | 1 | 18/27 | 18/27 | - | - | - | - | - | - |
| 2V0I | 0.97 | 1 | 0.29 | 1 | 0.53 | 0.46 | 5 | 397 | 0 | 12 | 12/51 | 12/51 | - | - | - | - | - | - |
| 3INA | 0.95 | 0.82 | 0.38 | 0.99 | 0.53 | 0.51 | 9 | 311 | 2 | 15 | 21/87 | 9/49 | 12/38 | - | - | - | - | - |
| 1UXY | 0.95 | 0.53 | 0.56 | 0.97 | 0.52 | 0.55 | 9 | 285 | 8 | 7 | 22/42 | 22/42 | - | - | - | - | - | - |
| 2AXR | 0.96 | 0.28 | 1 | 0.96 | 0.52 | 0.44 | 7 | 409 | 18 | 0 | 32/32 | 32/32 | - | - | - | - | - | - |
| 3CKZ | 0.95 | 0.59 | 0.5 | 0.98 | 0.52 | 0.54 | 10 | 323 | 7 | 10 | 28/51 | 28/51 | - | - | - | - | - | - |
| 4ENG | 0.9 | 0.79 | 0.41 | 0.98 | 0.52 | 0.54 | 11 | 163 | 3 | 16 | 31/88 | 29/66 | 2/22 | - | - | - | - | - |
| 1LSZ | 0.9 | 1 | 0.29 | 1 | 0.51 | 0.46 | 5 | 101 | 0 | 12 | 18/53 | 18/53 | - | - | - | - | - | - |
| 2QMJ | 0.97 | 0.34 | 0.79 | 0.97 | 0.51 | 0.48 | 11 | 752 | 21 | 3 | 29/37 | 29/37 | - | - | - | - | - | - |
| 1HV6 | 0.96 | 0.47 | 0.58 | 0.97 | 0.5 | 0.52 | 7 | 292 | 8 | 5 | 22/43 | 19/27 | 3/16 | - | - | - | - | - |
| 1IA7 | 0.95 | 0.3 | 0.88 | 0.96 | 0.5 | 0.45 | 7 | 349 | 16 | 1 | 35/37 | 35/37 | - | - | - | - | - | - |
| 1MWE | 0.94 | 0.73 | 0.38 | 0.99 | 0.5 | 0.5 | 11 | 328 | 4 | 18 | 28/85 | 28/47 | 0/38 | - | - | - | - | - |
| 3CZN | 0.97 | 0.46 | 0.59 | 0.98 | 0.5 | 0.52 | 17 | 884 | 20 | 12 | 56/101 | 53/69 | 3/32 | - | - | - | - | - |
| 3II1 | 0.95 | 0.26 | 1 | 0.95 | 0.5 | 0.41 | 8 | 449 | 23 | 0 | 29/31 | 29/31 | - | - | - | - | - | - |
| 2HRL | 0.95 | 1 | 0.25 | 1 | 0.49 | 0.4 | 2 | 101 | 0 | 6 | 12/40 | 12/40 | - | - | - | - | - | - |
| 1F0P | 0.92 | 0.65 | 0.42 | 0.97 | 0.48 | 0.51 | 11 | 218 | 6 | 15 | 25/82 | 23/47 | 2/35 | - | - | - | - | - |
| 1U8X | 0.97 | 0.39 | 0.63 | 0.98 | 0.48 | 0.48 | 5 | 384 | 8 | 3 | 17/27 | 17/27 | - | - | - | - | - | - |
| 1ZU0 | 0.93 | 0.33 | 0.79 | 0.93 | 0.48 | 0.47 | 15 | 418 | 30 | 4 | 60/82 | 60/82 | - | - | - | - | - | - |
| 2C4D | 0.89 | 1 | 0.26 | 1 | 0.48 | 0.42 | 14 | 309 | 0 | 39 | 49/187 | 4/37 | 19/61 | 9/31 | 10/23 | 7/35 | - | - |
| 2D3N | 0.89 | 0.69 | 0.41 | 0.97 | 0.48 | 0.52 | 25 | 359 | 11 | 36 | 87/248 | 20/71 | 32/53 | 0/19 | 14/22 | 13/31 | 8/42 | 0/10 |
| 2QZ2 | 0.89 | 0.65 | 0.46 | 0.96 | 0.48 | 0.54 | 11 | 136 | 6 | 13 | 30/87 | 30/44 | 0/43 | - | - | - | - | - |
| 1UXX | 0.9 | 1 | 0.25 | 1 | 0.47 | 0.4 | 4 | 100 | 0 | 12 | 24/70 | 4/24 | 20/46 | - | - | - | - | - |
| 2HS3 | 0.96 | 0.43 | 0.56 | 0.98 | 0.47 | 0.49 | 9 | 499 | 12 | 7 | 14/33 | 14/33 | - | - | - | - | - | - |
| 2V8K | 0.97 | 0.42 | 0.57 | 0.98 | 0.47 | 0.49 | 8 | 470 | 11 | 6 | 23/45 | 23/45 | - | - | - | - | - | - |
| 1XEZ | 0.96 | 0.31 | 0.73 | 0.97 | 0.46 | 0.43 | 8 | 554 | 18 | 3 | 18/32 | 18/32 | - | - | - | - | - | - |
| 2D7R | 0.94 | 0.41 | 0.59 | 0.96 | 0.46 | 0.48 | 13 | 457 | 19 | 9 | 40/71 | 0/25 | 40/46 | - | - | - | - | - |
| 3B8A | 0.97 | 0.6 | 0.38 | 0.99 | 0.46 | 0.46 | 6 | 397 | 4 | 10 | 16/42 | 16/42 | - | - | - | - | - | - |
| 1LXM | 0.97 | 0.39 | 0.55 | 0.98 | 0.45 | 0.46 | 11 | 717 | 17 | 9 | 37/76 | 13/13 | 24/47 | 0/16 | - | - | - | - |
| 1OT2 | 0.92 | 0.78 | 0.29 | 0.99 | 0.45 | 0.42 | 18 | 532 | 5 | 44 | 68/231 | 1/51 | 43/60 | 0/29 | 24/32 | 0/36 | 0/23 | - |
| 2IHO | 0.91 | 0.43 | 0.57 | 0.94 | 0.45 | 0.49 | 12 | 234 | 16 | 9 | 39/96 | 15/54 | 24/42 | - | - | - | - | - |
| 3EWR | 0.93 | 0.63 | 0.39 | 0.98 | 0.45 | 0.48 | 5 | 130 | 3 | 8 | 9/42 | 9/42 | - | - | - | - | - | - |
| 4PFK | 0.97 | 0.5 | 0.44 | 0.98 | 0.45 | 0.47 | 4 | 254 | 4 | 5 | 10/32 | 10/32 | - | - | - | - | - | - |
| 1I24 | 0.94 | 0.38 | 0.57 | 0.96 | 0.44 | 0.46 | 8 | 306 | 13 | 6 | 20/37 | 20/37 | - | - | - | - | - | - |
| 1MFU | 0.93 | 0.61 | 0.38 | 0.98 | 0.44 | 0.47 | 14 | 387 | 9 | 23 | 55/155 | 21/24 | 0/21 | 0/30 | 15/22 | 19/28 | 0/30 | - |
| 1PYY | 0.97 | 0.38 | 0.55 | 0.98 | 0.44 | 0.44 | 6 | 534 | 10 | 5 | 18/45 | 3/23 | 15/22 | - | - | - | - | - |
| 1FOA | 0.91 | 0.32 | 0.69 | 0.92 | 0.43 | 0.44 | 11 | 267 | 23 | 5 | 35/56 | 24/37 | 11/19 | - | - | - | - | - |
| 1GJW | 0.95 | 0.48 | 0.42 | 0.98 | 0.43 | 0.45 | 11 | 552 | 12 | 15 | 32/87 | 17/67 | 15/20 | - | - | - | - | - |
| 1MXD | 0.88 | 0.58 | 0.42 | 0.95 | 0.43 | 0.49 | 21 | 301 | 15 | 29 | 95/212 | 13/45 | 0/57 | 36/48 | 18/26 | 28/36 | - | - |
| 1TYW | 0.96 | 0.53 | 0.38 | 0.99 | 0.43 | 0.44 | 8 | 484 | 7 | 13 | 25/72 | 25/61 | 0/11 | - | - | - | - | - |
| 2F6D | 0.95 | 0.5 | 0.42 | 0.98 | 0.43 | 0.46 | 10 | 418 | 10 | 14 | 32/97 | 32/54 | 0/43 | - | - | - | - | - |
| 2WNB | 0.95 | 0.42 | 0.5 | 0.97 | 0.43 | 0.46 | 5 | 235 | 7 | 5 | 16/38 | 16/38 | - | - | - | - | - | - |
| 1ESW | 0.94 | 0.55 | 0.37 | 0.98 | 0.42 | 0.44 | 11 | 410 | 9 | 19 | 32/115 | 11/51 | 5/33 | 16/31 | - | - | - | - |
| 1G93 | 0.92 | 0.29 | 0.7 | 0.93 | 0.42 | 0.41 | 7 | 231 | 17 | 3 | 25/38 | 25/38 | - | - | - | - | - | - |
| 1V7X | 0.97 | 0.31 | 0.62 | 0.97 | 0.42 | 0.41 | 8 | 655 | 18 | 5 | 31/50 | 31/50 | - | - | - | - | - | - |
| 3MAN | 0.92 | 0.32 | 0.64 | 0.94 | 0.42 | 0.42 | 7 | 222 | 15 | 4 | 26/45 | 26/45 | - | - | - | - | - | - |
| 1OGO | 0.96 | 0.32 | 0.58 | 0.97 | 0.41 | 0.41 | 7 | 497 | 15 | 5 | 22/40 | 22/40 | - | - | - | - | - | - |
| 2IHJ | 0.94 | 0.43 | 0.45 | 0.96 | 0.41 | 0.44 | 9 | 322 | 12 | 11 | 26/58 | 26/58 | - | - | - | - | - | - |
| 2YHX | 0.98 | 0.5 | 0.33 | 0.99 | 0.4 | 0.4 | 3 | 345 | 3 | 6 | 6/20 | 6/20 | - | - | - | - | - | - |
| 1GYM | 0.96 | 0.27 | 0.6 | 0.97 | 0.39 | 0.38 | 3 | 248 | 8 | 2 | 17/23 | 17/23 | - | - | - | - | - | - |
| 1KC3 | 0.97 | 0.5 | 0.33 | 0.99 | 0.39 | 0.4 | 3 | 260 | 3 | 6 | 12/32 | 12/32 | - | - | - | - | - | - |
| 1LZR | 0.89 | 0.63 | 0.31 | 0.97 | 0.39 | 0.42 | 5 | 104 | 3 | 11 | 21/59 | 5/23 | 16/36 | - | - | - | - | - |
| 1O03 | 0.93 | 0.67 | 0.27 | 0.99 | 0.39 | 0.38 | 4 | 179 | 2 | 11 | 11/40 | 11/40 | - | - | - | - | - | - |
| 1UWF | 0.93 | 1 | 0.17 | 1 | 0.39 | 0.29 | 2 | 135 | 0 | 10 | 5/35 | 5/35 | - | - | - | - | - | - |
| 2Z4T | 0.95 | 0.22 | 0.75 | 0.95 | 0.39 | 0.34 | 6 | 437 | 21 | 2 | 23/33 | 23/33 | - | - | - | - | - | - |
| 3ABX | 0.93 | 0.47 | 0.39 | 0.97 | 0.39 | 0.43 | 9 | 305 | 10 | 14 | 46/89 | 31/51 | 15/38 | - | - | - | - | - |
| 2GJP | 0.91 | 0.46 | 0.42 | 0.95 | 0.38 | 0.44 | 15 | 362 | 18 | 21 | 40/150 | 6/28 | 0/30 | 21/40 | 4/20 | 9/11 | 0/21 | - |
| 3CX4 | 0.9 | 0.58 | 0.33 | 0.97 | 0.38 | 0.42 | 15 | 361 | 11 | 31 | 51/191 | 0/38 | 0/50 | 15/63 | 36/40 | - | - | - |
| 1MW0 | 0.89 | 0.51 | 0.36 | 0.96 | 0.37 | 0.42 | 22 | 493 | 21 | 40 | 76/230 | 10/60 | 65/104 | 1/25 | 0/12 | 0/29 | - | - |
| 1RP8 | 0.89 | 0.56 | 0.33 | 0.97 | 0.37 | 0.41 | 14 | 320 | 11 | 29 | 39/178 | 0/54 | 0/36 | 39/66 | 0/22 | - | - | - |
| 1UY4 | 0.9 | 0.57 | 0.31 | 0.97 | 0.37 | 0.4 | 4 | 102 | 3 | 9 | 22/52 | 22/52 | - | - | - | - | - | - |
| 1W3G | 0.95 | 0.67 | 0.22 | 0.99 | 0.37 | 0.33 | 4 | 281 | 2 | 14 | 10/73 | 7/42 | 3/31 | - | - | - | - | - |
| 2BHZ | 0.9 | 0.59 | 0.3 | 0.97 | 0.37 | 0.4 | 19 | 475 | 13 | 44 | 73/265 | 0/55 | 32/42 | 41/75 | 0/32 | 0/15 | 0/15 | 0/31 |
| 2EWE | 0.93 | 0.42 | 0.4 | 0.96 | 0.37 | 0.41 | 8 | 276 | 11 | 12 | 17/50 | 17/50 | - | - | - | - | - | - |
| 2JAF | 0.97 | 1 | 0.14 | 1 | 0.37 | 0.25 | 1 | 221 | 0 | 6 | 5/28 | 5/28 | - | - | - | - | - | - |
| 2PC8 | 0.93 | 0.33 | 0.5 | 0.95 | 0.37 | 0.4 | 8 | 324 | 16 | 8 | 23/57 | 23/25 | 0/32 | - | - | - | - | - |
| 2W62 | 0.92 | 0.78 | 0.19 | 1 | 0.37 | 0.31 | 7 | 365 | 2 | 29 | 15/124 | 8/58 | 7/38 | 0/12 | 0/16 | - | - | - |
| 2YVP | 0.93 | 0.5 | 0.33 | 0.98 | 0.37 | 0.4 | 4 | 155 | 4 | 8 | 8/42 | 8/42 | - | - | - | - | - | - |
| 2FST | 0.94 | 0.56 | 0.26 | 0.99 | 0.36 | 0.36 | 5 | 283 | 4 | 14 | 23/80 | 18/45 | 5/13 | 0/10 | 0/12 | - | - | - |
| 1FWV | 0.95 | 0.5 | 0.29 | 0.98 | 0.35 | 0.36 | 2 | 119 | 2 | 5 | 12/31 | 12/31 | - | - | - | - | - | - |
| 2V8L | 0.91 | 0.38 | 0.43 | 0.94 | 0.35 | 0.4 | 3 | 85 | 5 | 4 | 8/36 | 8/36 | - | - | - | - | - | - |
| 154L | 0.91 | 1 | 0.13 | 1 | 0.34 | 0.22 | 2 | 140 | 0 | 14 | 6/48 | 3/19 | 3/29 | - | - | - | - | - |
| 1FA2 | 0.93 | 0.24 | 0.57 | 0.94 | 0.34 | 0.33 | 8 | 418 | 26 | 6 | 18/38 | 18/38 | - | - | - | - | - | - |
| 1QNR | 0.95 | 0.24 | 0.57 | 0.96 | 0.34 | 0.33 | 4 | 284 | 13 | 3 | 23/33 | 23/33 | - | - | - | - | - | - |
| 2WQQ | 0.92 | 0.56 | 0.25 | 0.98 | 0.34 | 0.35 | 5 | 202 | 4 | 15 | 17/61 | 0/19 | 17/42 | - | - | - | - | - |
| 2F5V | 0.94 | 0.2 | 0.58 | 0.95 | 0.32 | 0.3 | 7 | 484 | 28 | 5 | 18/31 | 18/31 | - | - | - | - | - | - |
| 1JIL | 0.96 | 0.43 | 0.25 | 0.99 | 0.31 | 0.32 | 3 | 286 | 4 | 9 | 6/38 | 6/38 | - | - | - | - | - | - |
| 1NF9 | 0.97 | 0.5 | 0.2 | 0.99 | 0.31 | 0.29 | 1 | 174 | 1 | 4 | 3/12 | 3/12 | - | - | - | - | - | - |
| 1Q0Z | 0.91 | 0.24 | 0.5 | 0.93 | 0.31 | 0.32 | 6 | 251 | 19 | 6 | 7/36 | 7/36 | - | - | - | - | - | - |
| 1DLJ | 0.96 | 0.3 | 0.33 | 0.98 | 0.3 | 0.32 | 3 | 349 | 7 | 6 | 7/30 | 7/30 | - | - | - | - | - | - |
| 1PIG | 0.89 | 0.39 | 0.32 | 0.95 | 0.3 | 0.35 | 13 | 389 | 20 | 28 | 50/148 | 0/32 | 18/36 | 24/28 | 8/33 | 0/19 | - | - |
| 1OA7 | 0.92 | 0.29 | 0.4 | 0.94 | 0.29 | 0.33 | 4 | 168 | 10 | 6 | 15/28 | 15/28 | - | - | - | - | - | - |
| 3HN1 | 0.95 | 0.31 | 0.31 | 0.97 | 0.29 | 0.31 | 5 | 389 | 11 | 11 | 19/63 | 19/52 | 0/11 | - | - | - | - | - |
| 2GR2 | 0.97 | 0.25 | 0.33 | 0.98 | 0.28 | 0.29 | 2 | 360 | 6 | 4 | 7/27 | 7/27 | - | - | - | - | - | - |
| 2VWG | 0.96 | 0.38 | 0.25 | 0.98 | 0.28 | 0.3 | 3 | 301 | 5 | 9 | 5/33 | 5/33 | - | - | - | - | - | - |
| 2QKX | 0.94 | 0.29 | 0.29 | 0.97 | 0.26 | 0.29 | 4 | 321 | 10 | 10 | 10/37 | 10/37 | - | - | - | - | - | - |
| 3BIF | 0.96 | 0.33 | 0.23 | 0.98 | 0.26 | 0.27 | 3 | 372 | 6 | 10 | 6/45 | 6/45 | - | - | - | - | - | - |
| 1SB8 | 0.93 | 0.36 | 0.22 | 0.98 | 0.25 | 0.28 | 4 | 282 | 7 | 14 | 17/66 | 17/66 | - | - | - | - | - | - |
| 1SLY | 0.96 | 0.24 | 0.31 | 0.98 | 0.25 | 0.27 | 4 | 531 | 13 | 9 | 6/41 | 6/41 | - | - | - | - | - | - |
| 2J8F | 0.9 | 0.28 | 0.33 | 0.94 | 0.25 | 0.3 | 7 | 268 | 18 | 14 | 19/81 | 19/81 | - | - | - | - | - | - |
| 2JCR | 0.9 | 1 | 0.07 | 1 | 0.24 | 0.13 | 1 | 121 | 0 | 14 | 4/47 | 4/47 | - | - | - | - | - | - |
| 3H1Y | 0.95 | 0.25 | 0.27 | 0.97 | 0.24 | 0.26 | 3 | 333 | 9 | 8 | 7/44 | 7/44 | - | - | - | - | - | - |
| 2P3K | 0.92 | 0.29 | 0.25 | 0.96 | 0.23 | 0.27 | 2 | 132 | 5 | 6 | 7/33 | 7/33 | - | - | - | - | - | - |
| 3F3E | 0.92 | 0.3 | 0.24 | 0.97 | 0.23 | 0.27 | 6 | 398 | 14 | 19 | 19/81 | 12/36 | 7/45 | - | - | - | - | - |
| 1VST | 0.95 | 0.5 | 0.11 | 0.99 | 0.22 | 0.18 | 1 | 169 | 1 | 8 | 2/25 | 2/25 | - | - | - | - | - | - |
| 1ZX5 | 0.92 | 0.15 | 0.43 | 0.94 | 0.22 | 0.22 | 3 | 250 | 17 | 4 | 7/32 | 7/32 | - | - | - | - | - | - |
| 2BFQ | 0.92 | 0.4 | 0.17 | 0.98 | 0.22 | 0.24 | 2 | 155 | 3 | 10 | 2/42 | 2/42 | - | - | - | - | - | - |
| 2C9E | 0.96 | 0.29 | 0.2 | 0.98 | 0.22 | 0.24 | 2 | 301 | 5 | 8 | 7/38 | 0/15 | 7/23 | - | - | - | - | - |
| 1AUA | 0.93 | 0.23 | 0.25 | 0.96 | 0.21 | 0.24 | 3 | 264 | 10 | 9 | 7/55 | 7/31 | 0/24 | - | - | - | - | - |
| 2UAG | 0.95 | 0.25 | 0.23 | 0.98 | 0.21 | 0.24 | 3 | 358 | 9 | 10 | 9/46 | 9/46 | - | - | - | - | - | - |
| 1BG9 | 0.91 | 0.13 | 0.4 | 0.93 | 0.2 | 0.2 | 4 | 336 | 26 | 6 | 20/56 | 7/33 | 13/23 | - | - | - | - | - |
| 1GXO | 0.95 | 0.25 | 0.2 | 0.98 | 0.2 | 0.22 | 2 | 269 | 6 | 8 | 5/38 | 5/24 | 0/14 | - | - | - | - | - |
| 1TZF | 0.97 | 0.33 | 0.14 | 0.99 | 0.2 | 0.2 | 1 | 218 | 2 | 6 | 7/25 | 7/25 | - | - | - | - | - | - |
| 2C3E | 0.95 | 0.25 | 0.2 | 0.98 | 0.2 | 0.22 | 2 | 256 | 6 | 8 | 5/30 | 5/30 | - | - | - | - | - | - |
| 1J39 | 0.95 | 0.22 | 0.2 | 0.98 | 0.19 | 0.21 | 2 | 301 | 7 | 8 | 6/31 | 6/31 | - | - | - | - | - | - |
| 2ZZL | 0.96 | 0.33 | 0.13 | 0.99 | 0.19 | 0.18 | 1 | 207 | 2 | 7 | 2/29 | 2/29 | - | - | - | - | - | - |
| 1ILD | 0.88 | 0.23 | 0.26 | 0.93 | 0.18 | 0.24 | 5 | 215 | 17 | 14 | 14/91 | 0/25 | 10/21 | 4/21 | 0/12 | 0/12 | - | - |
| 1SX6 | 0.95 | 0.33 | 0.13 | 0.99 | 0.18 | 0.18 | 1 | 186 | 2 | 7 | 11/34 | 11/34 | - | - | - | - | - | - |
| 3IIU | 0.95 | 0.17 | 0.25 | 0.97 | 0.18 | 0.2 | 1 | 140 | 5 | 3 | 1/19 | 1/19 | - | - | - | - | - | - |
| 1OGQ | 0.94 | 0.33 | 0.12 | 0.99 | 0.17 | 0.17 | 2 | 271 | 4 | 15 | 6/60 | 6/22 | 0/24 | 0/14 | - | - | - | - |
| 1L8T | 0.93 | 0.22 | 0.17 | 0.97 | 0.16 | 0.19 | 2 | 232 | 7 | 10 | 7/60 | 7/60 | - | - | - | - | - | - |
| 2VU9 | 0.93 | 0.16 | 0.23 | 0.96 | 0.16 | 0.19 | 3 | 364 | 16 | 10 | 18/57 | 18/57 | - | - | - | - | - | - |
| 3GD9 | 0.94 | 0.29 | 0.12 | 0.99 | 0.16 | 0.17 | 2 | 320 | 5 | 15 | 4/55 | 4/55 | - | - | - | - | - | - |
| 1GYE | 0.91 | 0.13 | 0.3 | 0.93 | 0.15 | 0.18 | 3 | 253 | 20 | 7 | 6/26 | 6/26 | - | - | - | - | - | - |
| 1L1R | 0.94 | 0.25 | 0.13 | 0.98 | 0.15 | 0.17 | 1 | 152 | 3 | 7 | 6/32 | 6/32 | - | - | - | - | - | - |
| 1N0U | 0.99 | 0.17 | 0.14 | 0.99 | 0.15 | 0.15 | 1 | 746 | 5 | 6 | 4/23 | 4/23 | - | - | - | - | - | - |
| 3H2K | 0.95 | 0.5 | 0.06 | 1 | 0.15 | 0.1 | 1 | 332 | 1 | 17 | 1/51 | 1/40 | 0/11 | - | - | - | - | - |
| 3KH6 | 0.93 | 0.33 | 0.09 | 0.99 | 0.15 | 0.14 | 1 | 154 | 2 | 10 | 2/34 | 2/34 | - | - | - | - | - | - |
| 1LRJ | 0.91 | 0.2 | 0.17 | 0.96 | 0.14 | 0.18 | 3 | 272 | 12 | 15 | 7/57 | 0/36 | 7/21 | - | - | - | - | - |
| 3C9E | 0.93 | 0.33 | 0.08 | 0.99 | 0.14 | 0.13 | 1 | 175 | 2 | 11 | 2/45 | 2/45 | - | - | - | - | - | - |
| 1T10 | 0.97 | 0.2 | 0.1 | 0.99 | 0.13 | 0.13 | 1 | 494 | 4 | 9 | 1/25 | 1/25 | - | - | - | - | - | - |
| 2F5T | 0.92 | 0.33 | 0.06 | 0.99 | 0.12 | 0.11 | 1 | 190 | 2 | 15 | 2/60 | 2/60 | - | - | - | - | - | - |
| 4GPB | 0.97 | 0.18 | 0.11 | 0.99 | 0.12 | 0.13 | 2 | 744 | 9 | 17 | 4/59 | 4/20 | 0/39 | - | - | - | - | - |
| 1TL2 | 0.71 | 1 | 0.02 | 1 | 0.11 | 0.03 | 1 | 156 | 0 | 63 | 3/213 | 0/43 | 0/31 | 0/44 | 0/40 | 0/10 | 3/45 | - |
| 1U7G | 0.96 | 0.17 | 0.1 | 0.98 | 0.11 | 0.13 | 1 | 310 | 5 | 9 | 6/44 | 6/44 | - | - | - | - | - | - |
| 1YW1 | 0.96 | 0.1 | 0.14 | 0.97 | 0.1 | 0.12 | 1 | 342 | 9 | 6 | 4/26 | 4/26 | - | - | - | - | - | - |
| 3A7B | 0.97 | 0.08 | 0.17 | 0.98 | 0.1 | 0.11 | 1 | 498 | 11 | 5 | 3/28 | 3/28 | - | - | - | - | - | - |
| 1FA9 | 0.95 | 0.07 | 0.2 | 0.96 | 0.09 | 0.1 | 2 | 743 | 29 | 8 | 7/29 | 7/29 | - | - | - | - | - | - |
| 2GEJ | 0.95 | 0.14 | 0.09 | 0.98 | 0.09 | 0.11 | 1 | 298 | 6 | 10 | 1/37 | 1/37 | - | - | - | - | - | - |
| 3CL5 | 0.95 | 0.09 | 0.14 | 0.97 | 0.09 | 0.11 | 1 | 310 | 10 | 6 | 3/29 | 3/29 | - | - | - | - | - | - |
| 2BDM | 0.92 | 0.22 | 0.06 | 0.98 | 0.08 | 0.1 | 2 | 404 | 7 | 30 | 11/117 | 11/68 | 0/21 | 0/28 | - | - | - | - |
| 2GUP | 0.92 | 0.25 | 0.05 | 0.99 | 0.08 | 0.08 | 1 | 239 | 3 | 19 | 1/73 | 0/31 | 1/42 | - | - | - | - | - |
| 2IC8 | 0.85 | 0.33 | 0.04 | 0.99 | 0.08 | 0.08 | 1 | 138 | 2 | 22 | 1/94 | 1/43 | 0/15 | 0/11 | 0/25 | - | - | - |
| 3HD6 | 0.95 | 0.07 | 0.14 | 0.96 | 0.08 | 0.1 | 1 | 325 | 13 | 6 | 4/30 | 4/30 | - | - | - | - | - | - |
| 1F31 | 0.98 | 0.05 | 0.11 | 0.98 | 0.07 | 0.07 | 1 | 1124 | 18 | 8 | 8/42 | 8/42 | - | - | - | - | - | - |
| 1OD3 | 0.87 | 0.2 | 0.08 | 0.96 | 0.07 | 0.12 | 1 | 100 | 4 | 11 | 5/51 | 5/51 | - | - | - | - | - | - |
| 5ADH | 0.94 | 0.09 | 0.09 | 0.97 | 0.06 | 0.09 | 1 | 300 | 10 | 10 | 4/33 | 4/33 | - | - | - | - | - | - |
| 1UZ0 | 0.82 | 0.15 | 0.15 | 0.9 | 0.05 | 0.15 | 2 | 95 | 11 | 11 | 6/41 | 6/41 | - | - | - | - | - | - |
| 2WWU | 0.93 | 0.13 | 0.06 | 0.98 | 0.05 | 0.08 | 1 | 301 | 7 | 16 | 0/56 | 0/35 | 0/21 | - | - | - | - | - |
| 1OFL | 0.87 | 0.1 | 0.05 | 0.95 | 0.01 | 0.07 | 2 | 367 | 18 | 36 | 4/118 | 4/83 | 0/35 | - | - | - | - | - |
| 148L | 0 | 0 | 0 | 0 | 0 | 0 | 0 | 136 | 0 | 18 | 0 | 0 | - | - | - | - | - | - |
| 1DRK | 0 | 0 | 0 | 0 | 0 | 0 | 0 | 214 | 0 | 11 | 0 | 0 | - | - | - | - | - | - |
| 1ECY | 0 | 0 | 0 | 0 | 0 | 0 | 0 | 110 | 0 | 28 | 0 | 0 | - | - | - | - | - | - |
| 1EXA | 0 | 0 | 0 | 0 | 0 | 0 | 0 | 214 | 0 | 4 | 0 | 0 | - | - | - | - | - | - |
| 1GWM | 0 | 0 | 0 | 0 | 0 | 0 | 0 | 131 | 0 | 14 | 0 | 0 | - | - | - | - | - | - |
| 1J8R | 0 | 0 | 0 | 0 | 0 | 0 | 0 | 178 | 0 | 10 | 0 | 0 | - | - | - | - | - | - |
| 1JPC | 0 | 0 | 0 | 0 | 0 | 0 | 0 | 74 | 0 | 26 | 0 | 0 | - | - | - | - | - | - |
| 1KZN | 0 | 0 | 0 | 0 | 0 | 0 | 0 | 162 | 0 | 13 | 0 | 0 | - | - | - | - | - | - |
| 1NPL | 0 | 0 | 0 | 0 | 0 | 0 | 0 | 78 | 0 | 25 | 0 | 0 | - | - | - | - | - | - |
| 1OFC | 0 | 0 | 0 | 0 | 0 | 0 | 0 | 223 | 0 | 8 | 0 | 0 | - | - | - | - | - | - |
| 1OPR | 0 | 0 | 0 | 0 | 0 | 0 | 0 | 186 | 0 | 7 | 0 | 0 | - | - | - | - | - | - |
| 1QGI | 0 | 0 | 0 | 0 | 0 | 0 | 0 | 223 | 0 | 10 | 0 | 0 | - | - | - | - | - | - |
| 1TJY | 0 | 0 | 0 | 0 | 0 | 0 | 0 | 244 | 0 | 13 | 0 | 0 | - | - | - | - | - | - |
| 1UX7 | 0 | 0 | 0 | 0 | 0 | 0 | 0 | 100 | 0 | 6 | 0 | 0 | - | - | - | - | - | - |
| 1YC9 | 0 | 0 | 0 | 0 | 0 | 0 | 0 | 392 | 0 | 9 | 0 | 0 | - | - | - | - | - | - |
| 2C56 | 0 | 0 | 0 | 0 | 0 | 0 | 0 | 196 | 0 | 9 | 0 | 0 | - | - | - | - | - | - |
| 2CCV | 0 | 0 | 0 | 0 | 0 | 0 | 0 | 87 | 0 | 7 | 0 | 0 | - | - | - | - | - | - |
| 2JG0 | 0 | 0 | 0 | 0 | 0 | 0 | 0 | 432 | 0 | 16 | 0 | 0 | - | - | - | - | - | - |
| 2JH7 | 0 | 0 | 0 | 0 | 0 | 0 | 0 | 192 | 0 | 6 | 0 | 0 | - | - | - | - | - | - |
| 2JHL | 0 | 0 | 0 | 0 | 0 | 0 | 0 | 184 | 0 | 8 | 0 | 0 | - | - | - | - | - | - |
| 2RDG | 0 | 0 | 0 | 0 | 0 | 0 | 0 | 173 | 0 | 11 | 0 | 0 | - | - | - | - | - | - |
| 2V5T | 0 | 0 | 0 | 0 | 0 | 0 | 0 | 166 | 0 | 7 | 0 | 0 | - | - | - | - | - | - |
| 2VK2 | 0 | 0 | 0 | 0 | 0 | 0 | 0 | 234 | 0 | 14 | 0 | 0 | - | - | - | - | - | - |
| 2VXK | 0 | 0 | 0 | 0 | 0 | 0 | 0 | 146 | 0 | 10 | 0 | 0 | - | - | - | - | - | - |
| 2YVV | 0 | 0 | 0 | 0 | 0 | 0 | 0 | 319 | 0 | 11 | 0 | 0 | - | - | - | - | - | - |
| 2Z8L | 0 | 0 | 0 | 0 | 0 | 0 | 0 | 173 | 0 | 11 | 0 | 0 | - | - | - | - | - | - |
| 2ZJ3 | 0 | 0 | 0 | 0 | 0 | 0 | 0 | 294 | 0 | 13 | 0 | 0 | - | - | - | - | - | - |
| 3CT5 | 0 | 0 | 0 | 0 | 0 | 0 | 0 | 111 | 0 | 14 | 0 | 0 | - | - | - | - | - | - |
| 3D1R | 0 | 0 | 0 | 0 | 0 | 0 | 0 | 273 | 0 | 13 | 0 | 0 | - | - | - | - | - | - |
| 3HZS | 0 | 0 | 0 | 0 | 0 | 0 | 0 | 170 | 0 | 16 | 0 | 0 | - | - | - | - | - | - |
| 3IID | 0 | 0 | 0 | 0 | 0 | 0 | 0 | 145 | 0 | 13 | 0 | 0 | - | - | - | - | - | - |
| 966C | 0 | 0 | 0 | 0 | 0 | 0 | 0 | 137 | 0 | 12 | 0 | 0 | - | - | - | - | - | - |
| 9ABP | 0 | 0 | 0 | 0 | 0 | 0 | 0 | 246 | 0 | 16 | 0 | 0 | - | - | - | - | - | - |
| 1IZ2 | 0.98 | 0 | 0 | 1 | -0.01 | 0 | 0 | 335 | 1 | 7 | 0/29 | 0/29 | - | - | - | - | - | - |
| 1J2Z | 0.95 | 0 | 0 | 1 | -0.01 | 0 | 0 | 226 | 1 | 11 | 0/35 | 0/35 | - | - | - | - | - | - |
| 1JZS | 0.97 | 0 | 0 | 1 | -0.01 | 0 | 0 | 754 | 4 | 16 | 0/42 | 0/42 | - | - | - | - | - | - |
| 1PW5 | 0.97 | 0 | 0 | 1 | -0.01 | 0 | 0 | 227 | 1 | 6 | 0/30 | 0/30 | - | - | - | - | - | - |
| 1TAQ | 0.96 | 0 | 0 | 0.97 | -0.01 | 0 | 0 | 718 | 22 | 5 | 0/23 | 0/23 | - | - | - | - | - | - |
| 1YON | 0.95 | 0 | 0 | 1 | -0.01 | 0 | 0 | 253 | 1 | 13 | 0/43 | 0/14 | 0/29 | - | - | - | - | - |
| 2FN8 | 0.95 | 0 | 0 | 1 | -0.01 | 0 | 0 | 230 | 1 | 11 | 0/34 | 0/34 | - | - | - | - | - | - |
| 2OBT | 0.97 | 0 | 0 | 0.99 | -0.01 | 0 | 0 | 280 | 2 | 6 | 0/21 | 0/21 | - | - | - | - | - | - |
| 2R68 | 0.97 | 0 | 0 | 1 | -0.01 | 0 | 0 | 388 | 1 | 12 | 0/42 | 0/42 | - | - | - | - | - | - |
| 2ZG3 | 0.96 | 0 | 0 | 1 | -0.01 | 0 | 0 | 192 | 1 | 8 | 0/34 | 0/34 | - | - | - | - | - | - |
| 3FWL | 0.97 | 0 | 0 | 0.99 | -0.01 | 0 | 0 | 641 | 5 | 14 | 0/60 | 0/60 | - | - | - | - | - | - |
| 1HFU | 0.96 | 0 | 0 | 0.98 | -0.02 | 0 | 0 | 432 | 11 | 9 | 0/44 | 0/30 | 0/14 | - | - | - | - | - |
| 1J4N | 0.96 | 0 | 0 | 0.99 | -0.02 | 0 | 0 | 217 | 2 | 8 | 0/28 | 0/28 | - | - | - | - | - | - |
| 1MOR | 0.95 | 0 | 0 | 0.99 | -0.02 | 0 | 0 | 302 | 4 | 13 | 0/39 | 0/39 | - | - | - | - | - | - |
| 1QZ6 | 0.96 | 0 | 0 | 0.98 | -0.02 | 0 | 0 | 310 | 7 | 7 | 0/24 | 0/24 | - | - | - | - | - | - |
| 1TXZ | 0.93 | 0 | 0 | 0.99 | -0.02 | 0 | 0 | 211 | 2 | 14 | 0/46 | 0/46 | - | - | - | - | - | - |
| 1W6K | 0.96 | 0 | 0 | 0.97 | -0.02 | 0 | 0 | 606 | 16 | 10 | 0/49 | 0/25 | 0/24 | - | - | - | - | - |
| 1YLJ | 0.94 | 0 | 0 | 0.99 | -0.02 | 0 | 0 | 220 | 3 | 10 | 0/33 | 0/33 | - | - | - | - | - | - |
| 1YMG | 0.95 | 0 | 0 | 0.99 | -0.02 | 0 | 0 | 199 | 3 | 7 | 2/34 | 2/19 | 0/15 | - | - | - | - | - |
| 2A0Z | 0.97 | 0 | 0 | 0.98 | -0.02 | 0 | 0 | 599 | 10 | 11 | 0/40 | 0/20 | 0/20 | - | - | - | - | - |
| 2CNE | 0.97 | 0 | 0 | 0.99 | -0.02 | 0 | 0 | 251 | 3 | 6 | 0/32 | 0/32 | - | - | - | - | - | - |
| 2EBH | 0.97 | 0 | 0 | 0.99 | -0.02 | 0 | 0 | 632 | 7 | 13 | 0/66 | 0/49 | 0/17 | - | - | - | - | - |
| 2EVU | 0.96 | 0 | 0 | 0.99 | -0.02 | 0 | 0 | 210 | 3 | 6 | 0/30 | 0/17 | 0/13 | - | - | - | - | - |
| 2HHQ | 0.94 | 0 | 0 | 0.99 | -0.02 | 0 | 0 | 488 | 5 | 28 | 0/105 | 0/47 | 0/23 | 0/35 | - | - | - | - |
| 2UUI | 0.94 | 0 | 0 | 0.99 | -0.02 | 0 | 0 | 135 | 1 | 7 | 0/23 | 0/23 | - | - | - | - | - | - |
| 2VF5 | 0.95 | 0 | 0 | 0.99 | -0.02 | 0 | 0 | 310 | 4 | 12 | 0/37 | 0/37 | - | - | - | - | - | - |
| 3B9Z | 0.95 | 0 | 0 | 0.97 | -0.02 | 0 | 0 | 318 | 9 | 7 | 0/23 | 0/12 | 0/11 | - | - | - | - | - |
| 3CR9 | 0.96 | 0 | 0 | 0.98 | -0.02 | 0 | 0 | 612 | 14 | 10 | 0/36 | 0/36 | - | - | - | - | - | - |
| 3FHH | 0.94 | 0 | 0 | 0.95 | -0.02 | 0 | 0 | 548 | 31 | 6 | 0/25 | 0/25 | - | - | - | - | - | - |
| 3FP0 | 0.96 | 0 | 0 | 0.97 | -0.02 | 0 | 0 | 420 | 12 | 8 | 0/26 | 0/26 | - | - | - | - | - | - |
| 3GZ9 | 0.97 | 0 | 0 | 0.99 | -0.02 | 0 | 0 | 233 | 3 | 5 | 0/23 | 0/23 | - | - | - | - | - | - |
| 3ICV | 0.95 | 0 | 0 | 0.98 | -0.02 | 0 | 0 | 243 | 5 | 7 | 0/26 | 0/13 | 0/13 | - | - | - | - | - |
| 3IFE | 0.96 | 0 | 0 | 0.99 | -0.02 | 0 | 0 | 365 | 5 | 12 | 0/40 | 0/19 | 0/21 | - | - | - | - | - |
| 1A5Z | 0.94 | 0 | 0 | 0.98 | -0.03 | 0 | 0 | 262 | 6 | 10 | 0/42 | 0/22 | 0/20 | - | - | - | - | - |
| 1C4O | 0.94 | 0 | 0 | 0.96 | -0.03 | 0 | 0 | 424 | 19 | 8 | 0/16 | 0/16 | - | - | - | - | - | - |
| 1CPY | 0.94 | 0 | 0 | 0.96 | -0.03 | 0 | 0 | 333 | 13 | 9 | 0/29 | 0/13 | 0/16 | - | - | - | - | - |
| 1EN2 | 0.92 | 0 | 0 | 0.99 | -0.03 | 0 | 0 | 75 | 1 | 6 | 0/36 | 0/36 | - | - | - | - | - | - |
| 1FI1 | 0.94 | 0 | 0 | 0.96 | -0.03 | 0 | 0 | 654 | 28 | 11 | 0/36 | 0/36 | - | - | - | - | - | - |
| 1LLC | 0.93 | 0 | 0 | 0.97 | -0.03 | 0 | 0 | 284 | 10 | 10 | 0/32 | 0/32 | - | - | - | - | - | - |
| 1OB2 | 0.92 | 0 | 0 | 0.99 | -0.03 | 0 | 0 | 340 | 4 | 24 | 0/84 | 0/42 | 0/42 | - | - | - | - | - |
| 1Q2P | 0.95 | 0 | 0 | 0.98 | -0.03 | 0 | 0 | 228 | 5 | 7 | 0/31 | 0/31 | - | - | - | - | - | - |
| 1U65 | 0.94 | 0 | 0 | 0.96 | -0.03 | 0 | 0 | 447 | 19 | 8 | 0/36 | 0/36 | - | - | - | - | - | - |
| 2BBH | 0.89 | 0 | 0 | 0.99 | -0.03 | 0 | 0 | 192 | 2 | 21 | 0/83 | 0/65 | 0/18 | - | - | - | - | - |
| 2C78 | 0.94 | 0 | 0 | 0.97 | -0.03 | 0 | 0 | 340 | 12 | 10 | 0/33 | 0/33 | - | - | - | - | - | - |
| 2E59 | 0.93 | 0 | 0 | 0.99 | -0.03 | 0 | 0 | 131 | 2 | 8 | 0/37 | 0/24 | 0/13 | - | - | - | - | - |
| 2FUE | 0.94 | 0 | 0 | 0.98 | -0.03 | 0 | 0 | 210 | 4 | 9 | 0/32 | 0/32 | - | - | - | - | - | - |
| 2GY5 | 0.94 | 0 | 0 | 0.97 | -0.03 | 0 | 0 | 386 | 11 | 12 | 0/41 | 0/13 | 0/11 | 0/17 | - | - | - | - |
| 2O9G | 0.94 | 0 | 0 | 0.98 | -0.03 | 0 | 0 | 197 | 5 | 7 | 2/30 | 2/30 | - | - | - | - | - | - |
| 2VE0 | 0.95 | 0 | 0 | 0.98 | -0.03 | 0 | 0 | 425 | 11 | 12 | 0/46 | 0/36 | 0/10 | - | - | - | - | - |
| 2W1P | 0.93 | 0 | 0 | 0.98 | -0.03 | 0 | 0 | 218 | 4 | 13 | 0/70 | 0/18 | 0/30 | 0/22 | - | - | - | - |
| 3BKL | 0.91 | 0 | 0 | 0.92 | -0.03 | 0 | 0 | 481 | 40 | 6 | 0/23 | 0/23 | - | - | - | - | - | - |
| 3BKV | 0.91 | 0 | 0 | 0.99 | -0.03 | 0 | 0 | 229 | 2 | 22 | 0/68 | 0/68 | - | - | - | - | - | - |
| 3DAS | 0.93 | 0 | 0 | 0.97 | -0.03 | 0 | 0 | 283 | 8 | 12 | 0/41 | 0/23 | 0/18 | - | - | - | - | - |
| 1EU1 | 0.92 | 0 | 0 | 0.94 | -0.04 | 0 | 0 | 638 | 39 | 17 | 0/47 | 0/24 | 0/23 | - | - | - | - | - |
| 1FE2 | 0.92 | 0 | 0 | 0.95 | -0.04 | 0 | 0 | 469 | 24 | 15 | 0/64 | 0/19 | 0/45 | - | - | - | - | - |
| 1J1M | 0.91 | 0 | 0 | 0.98 | -0.04 | 0 | 0 | 223 | 5 | 16 | 0/56 | 0/16 | 0/40 | - | - | - | - | - |
| 1KQR | 0.92 | 0 | 0 | 0.97 | -0.04 | 0 | 0 | 136 | 4 | 8 | 0/30 | 0/30 | - | - | - | - | - | - |
| 1OAF | 0.92 | 0 | 0 | 0.95 | -0.04 | 0 | 0 | 216 | 11 | 9 | 0/32 | 0/32 | - | - | - | - | - | - |
| 1P49 | 0.92 | 0 | 0 | 0.94 | -0.04 | 0 | 0 | 466 | 32 | 10 | 0/26 | 0/14 | 0/12 | - | - | - | - | - |
| 1SE3 | 0.93 | 0 | 0 | 0.97 | -0.04 | 0 | 0 | 187 | 6 | 9 | 0/34 | 0/34 | - | - | - | - | - | - |
| 1UXT | 0.92 | 0 | 0 | 0.94 | -0.04 | 0 | 0 | 420 | 25 | 10 | 0/31 | 0/31 | - | - | - | - | - | - |
| 1UYQ | 0.91 | 0 | 0 | 0.93 | -0.04 | 0 | 0 | 360 | 29 | 8 | 0/29 | 0/29 | - | - | - | - | - | - |
| 2DWJ | 0.93 | 0 | 0 | 0.96 | -0.04 | 0 | 0 | 294 | 12 | 11 | 0/42 | 0/42 | - | - | - | - | - | - |
| 2HZH | 0.93 | 0 | 0 | 0.97 | -0.04 | 0 | 0 | 415 | 15 | 15 | 0/58 | 0/14 | 0/12 | 0/32 | - | - | - | - |
| 2RH1 | 0.93 | 0 | 0 | 0.96 | -0.04 | 0 | 0 | 392 | 16 | 14 | 0/51 | 0/51 | - | - | - | - | - | - |
| 3C02 | 0.93 | 0 | 0 | 0.95 | -0.04 | 0 | 0 | 191 | 10 | 5 | 0/17 | 0/17 | - | - | - | - | - | - |
| 3D3H | 0.9 | 0 | 0 | 0.98 | -0.04 | 0 | 0 | 138 | 3 | 13 | 0/39 | 0/39 | - | - | - | - | - | - |
| 3DIV | 0.93 | 0 | 0 | 0.97 | -0.04 | 0 | 0 | 410 | 14 | 18 | 0/60 | 0/22 | 0/17 | 0/21 | - | - | - | - |
| 3I2T | 0.93 | 0 | 0 | 0.95 | -0.04 | 0 | 0 | 452 | 22 | 12 | 0/38 | 0/27 | 0/11 | - | - | - | - | - |
| 1ODA | 0.9 | 0 | 0 | 0.96 | -0.05 | 0 | 0 | 100 | 4 | 7 | 0/35 | 0/35 | - | - | - | - | - | - |
| 2BGI | 0.88 | 0 | 0 | 0.98 | -0.05 | 0 | 0 | 199 | 4 | 24 | 0/83 | 0/27 | 0/56 | - | - | - | - | - |
| 2O0V | 0.9 | 0 | 0 | 0.96 | -0.05 | 0 | 0 | 319 | 14 | 20 | 0/58 | 0/37 | 0/21 | - | - | - | - | - |
| 3A6T | 0.91 | 0 | 0 | 0.95 | -0.05 | 0 | 0 | 103 | 5 | 5 | 0/29 | 0/29 | - | - | - | - | - | - |
| 1DL2 | 0.89 | 0 | 0 | 0.92 | -0.06 | 0 | 0 | 385 | 32 | 16 | 0/60 | 0/17 | 0/14 | 0/29 | - | - | - | - |
| 1FX8 | 0.89 | 0 | 0 | 0.96 | -0.06 | 0 | 0 | 202 | 8 | 18 | 0/59 | 0/39 | 0/20 | - | - | - | - | - |
| 3FUS | 0.88 | 0 | 0 | 0.91 | -0.06 | 0 | 0 | 266 | 28 | 10 | 0/48 | 0/33 | 0/15 | - | - | - | - | - |
| Total | 0.96 | 0.53 | 0.51 | 0.98 | 0.49 | 0.52 | 4039 | 157394 | 3656 | 3960 |  |  |  |  |  |  |  |  |

**Table S3:** Ten-fold cross validation SVM prediction accuracy benchmarks on the S497 dataset. The dataset, the ten-fold cross validation, and the benchmark measurements have been described in the main text. Matthews correlation coefficient (MCC), F-score(Fsc), Accuracy(Acc), Precision(Pre), Sensitivity(Sen) and Specificity(Spe) are shown in Equations (4)~(9). TP, FP, TN, and FN are true positive, false positive, true negative, and false negative respectively. C1~C7 represent carbohydrate binding sites in each of the test proteins; different protein has different number of binding sites. In these columns, the number of the predicted true positive atoms is shown over the actual number of atoms involving in the binding site. Interactive examination of the prediction results for each of the proteins in the S497 dataset can be accessed from the web server: <http://ismblab.genomics.sinica.edu.tw/>> benchmark > protein-carbohydrate.
