# Supplementary material for: Prediction of Carbohydrate Binding Sites on Protein Surfaces with 3-Dimensional Probability Density Distributions of Interacting Atoms
Source: PLoS One. 2012 Jul 25;7(7):e40846. doi: 10.1371/journal.pone.0040846 (PMC3405063; doi:10.1371/journal.pone.0040846)
Supplement: Table S4 — Ten-fold cross validation ANN_BAGGING prediction accuracy benchmarks on the S497 dataset. The dataset, the ten-fold cross validation, and the benchmark measurements have been described in the main text. Matthews correlation coefficient (MCC), F-score(Fsc), Accuracy(Acc), Precision(Pre), Sensitivity(Sen) and Specificity(Spe) are shown in Equations (4)∼(9). TP, FP, TN, and FN are true positive, false positive, true negative, and false negative respectively. C1∼C7 represent carbohydrate binding sites in each of the test proteins; different protein has different number of binding sites. In these columns, the number of the predicted true positive atoms is shown over the actual number of atoms involving in the binding site. Interactive examination of the prediction results for each of the proteins in the S497 dataset can be accessed from the web server: http://ismblab.genomics.sinica.edu.tw/> benchmark > protein-carbohydrate. (DOC) [file pone.0040846.s007.doc]

**Table S4**

| **PDBID** | **Residue-based ANN_BAGGING prediction benchmarks of ten-fold cross validation on S497 dataset** | | | | | | | | | | | | | | | | | |
| --- | --- | --- | --- | --- | --- | --- | --- | --- | --- | --- | --- | --- | --- | --- | --- | --- | --- | --- |
|  | **Residues Level** | | | | | | | | | | **Predict positive atoms / Actual binding atoms** | | | | | | | |
|  | **Acc** | **Pre** | **Sen** | **Spe** | **MCC** | **Fsc** | **TP** | **TN** | **FP** | **FN** | **All** | **C1** | **C2** | **C3** | **C4** | **C5** | **C6** | **C7** |
| 1MVQ | 1.00 | 1.00 | 1.00 | 1.00 | 1.00 | 1.00 | 10 | 197 | 0 | 0 | 31/33 | 31/33 | - | - | - | - | - | - |
| 1RPJ | 1.00 | 1.00 | 1.00 | 1.00 | 1.00 | 1.00 | 12 | 229 | 0 | 0 | 33/33 | 33/33 | - | - | - | - | - | - |
| 2V72 | 1.00 | 1.00 | 1.00 | 1.00 | 1.00 | 1.00 | 7 | 123 | 0 | 0 | 24/30 | 24/30 | - | - | - | - | - | - |
| 2OWZ | 1.00 | 1.00 | 0.92 | 1.00 | 0.96 | 0.96 | 12 | 282 | 0 | 1 | 26/32 | 26/32 | - | - | - | - | - | - |
| 2ZID | 1.00 | 0.91 | 1.00 | 1.00 | 0.95 | 0.95 | 19 | 468 | 2 | 0 | 57/60 | 57/60 | - | - | - | - | - | - |
| 1DIL | 0.99 | 0.90 | 1.00 | 0.99 | 0.94 | 0.94 | 17 | 311 | 2 | 0 | 39/40 | 39/40 | - | - | - | - | - | - |
| 1K12 | 0.99 | 1.00 | 0.89 | 1.00 | 0.94 | 0.94 | 8 | 131 | 0 | 1 | 15/19 | 15/19 | - | - | - | - | - | - |
| 1KWF | 0.99 | 0.96 | 0.92 | 1.00 | 0.93 | 0.94 | 22 | 288 | 1 | 2 | 86/97 | 41/51 | 34/35 | 11/11 | - | - | - | - |
| 1S0I | 0.99 | 1.00 | 0.81 | 1.00 | 0.90 | 0.90 | 17 | 538 | 0 | 4 | 51/64 | 51/64 | - | - | - | - | - | - |
| 2JE7 | 0.99 | 0.90 | 0.90 | 1.00 | 0.90 | 0.90 | 9 | 201 | 1 | 1 | 32/36 | 32/36 | - | - | - | - | - | - |
| 2ZXT | 0.99 | 0.81 | 1.00 | 0.99 | 0.90 | 0.90 | 17 | 405 | 4 | 0 | 64/66 | 42/43 | 22/23 | - | - | - | - | - |
| 1EU8 | 0.99 | 0.96 | 0.84 | 1.00 | 0.89 | 0.89 | 21 | 330 | 1 | 4 | 58/67 | 48/53 | 10/14 | - | - | - | - | - |
| 1MG1 | 0.99 | 0.80 | 1.00 | 0.99 | 0.89 | 0.89 | 16 | 415 | 4 | 0 | 55/56 | 55/56 | - | - | - | - | - | - |
| 2VI0 | 0.98 | 0.82 | 1.00 | 0.98 | 0.89 | 0.90 | 22 | 212 | 5 | 0 | 86/88 | 86/88 | - | - | - | - | - | - |
| 2YXS | 0.99 | 0.80 | 1.00 | 0.99 | 0.89 | 0.89 | 8 | 128 | 2 | 0 | 24/27 | 24/27 | - | - | - | - | - | - |
| 1C3N | 0.99 | 1.00 | 0.78 | 1.00 | 0.88 | 0.88 | 7 | 126 | 0 | 2 | 14/26 | 14/26 | - | - | - | - | - | - |
| 1J8V | 0.99 | 0.77 | 1.00 | 0.99 | 0.88 | 0.87 | 17 | 499 | 5 | 0 | 60/60 | 60/60 | - | - | - | - | - | - |
| 1MQE | 0.99 | 1.00 | 0.78 | 1.00 | 0.88 | 0.88 | 7 | 164 | 0 | 2 | 15/24 | 15/24 | - | - | - | - | - | - |
| 2J7M | 0.99 | 1.00 | 0.78 | 1.00 | 0.88 | 0.88 | 7 | 126 | 0 | 2 | 30/43 | 30/43 | - | - | - | - | - | - |
| 2D6O | 0.99 | 0.88 | 0.88 | 0.99 | 0.87 | 0.88 | 7 | 135 | 1 | 1 | 24/31 | 24/31 | - | - | - | - | - | - |
| 1UA4 | 0.99 | 0.87 | 0.87 | 1.00 | 0.86 | 0.87 | 13 | 375 | 2 | 2 | 30/35 | 30/35 | - | - | - | - | - | - |
| 1UP2 | 0.98 | 0.76 | 1.00 | 0.98 | 0.86 | 0.86 | 19 | 236 | 6 | 0 | 57/65 | 57/65 | - | - | - | - | - | - |
| 1US2 | 0.99 | 0.82 | 0.92 | 0.99 | 0.86 | 0.86 | 22 | 442 | 5 | 2 | 79/88 | 35/42 | 44/46 | - | - | - | - | - |
| 2DT3 | 0.98 | 0.82 | 0.92 | 0.98 | 0.86 | 0.87 | 23 | 299 | 5 | 2 | 96/119 | 60/62 | 23/28 | 13/29 | - | - | - | - |
| 2FMD | 0.99 | 0.83 | 0.91 | 0.99 | 0.86 | 0.87 | 10 | 187 | 2 | 1 | 35/39 | 35/39 | - | - | - | - | - | - |
| 2VMG | 0.99 | 1.00 | 0.75 | 1.00 | 0.86 | 0.86 | 6 | 125 | 0 | 2 | 17/23 | 17/23 | - | - | - | - | - | - |
| 1UAS | 0.98 | 0.74 | 1.00 | 0.98 | 0.85 | 0.85 | 14 | 303 | 5 | 0 | 41/41 | 41/41 | - | - | - | - | - | - |
| 2F0Z | 0.99 | 0.92 | 0.80 | 1.00 | 0.85 | 0.86 | 12 | 304 | 1 | 3 | 33/45 | 12/15 | 21/30 | - | - | - | - | - |
| 2GH9 | 0.98 | 0.77 | 0.94 | 0.98 | 0.85 | 0.85 | 17 | 314 | 5 | 1 | 67/74 | 67/74 | - | - | - | - | - | - |
| 2OVU | 0.99 | 0.90 | 0.82 | 1.00 | 0.85 | 0.86 | 9 | 196 | 1 | 2 | 33/40 | 33/40 | - | - | - | - | - | - |
| 2VX6 | 0.98 | 0.80 | 0.91 | 0.98 | 0.84 | 0.85 | 20 | 285 | 5 | 2 | 74/80 | 74/80 | - | - | - | - | - | - |
| 3H3K | 0.99 | 0.86 | 0.83 | 0.99 | 0.84 | 0.84 | 19 | 432 | 3 | 4 | 65/79 | 24/34 | 41/45 | - | - | - | - | - |
| 1C1L | 0.98 | 0.80 | 0.89 | 0.98 | 0.83 | 0.84 | 8 | 118 | 2 | 1 | 28/33 | 28/33 | - | - | - | - | - | - |
| 1UQY | 0.98 | 0.86 | 0.83 | 0.99 | 0.83 | 0.84 | 19 | 291 | 3 | 4 | 71/90 | 39/58 | 32/32 | - | - | - | - | - |
| 2V4V | 0.97 | 0.70 | 1.00 | 0.97 | 0.83 | 0.82 | 7 | 107 | 3 | 0 | 34/35 | 34/35 | - | - | - | - | - | - |
| 2VGD | 0.96 | 0.76 | 0.95 | 0.96 | 0.83 | 0.84 | 19 | 151 | 6 | 1 | 78/82 | 78/82 | - | - | - | - | - | - |
| 2WZF | 0.99 | 0.70 | 1.00 | 0.99 | 0.83 | 0.82 | 14 | 456 | 6 | 0 | 31/34 | 31/34 | - | - | - | - | - | - |
| 3JUL | 0.99 | 0.83 | 0.83 | 0.99 | 0.83 | 0.83 | 10 | 256 | 2 | 2 | 27/31 | 27/31 | - | - | - | - | - | - |
| 1FNZ | 0.98 | 1.00 | 0.69 | 1.00 | 0.82 | 0.82 | 9 | 196 | 0 | 4 | 27/41 | 27/41 | - | - | - | - | - | - |
| 1I82 | 0.98 | 0.83 | 0.83 | 0.99 | 0.82 | 0.83 | 10 | 158 | 2 | 2 | 39/45 | 39/45 | - | - | - | - | - | - |
| 1KTC | 0.98 | 0.74 | 0.93 | 0.99 | 0.82 | 0.82 | 14 | 335 | 5 | 1 | 43/48 | 43/48 | - | - | - | - | - | - |
| 1YOE | 0.98 | 0.68 | 1.00 | 0.98 | 0.82 | 0.81 | 13 | 245 | 6 | 0 | 31/31 | 31/31 | - | - | - | - | - | - |
| 2B46 | 0.96 | 0.75 | 0.94 | 0.97 | 0.82 | 0.83 | 15 | 142 | 5 | 1 | 46/48 | 46/48 | - | - | - | - | - | - |
| 2WZG | 0.99 | 0.80 | 0.86 | 0.99 | 0.82 | 0.83 | 12 | 442 | 3 | 2 | 27/36 | 27/36 | - | - | - | - | - | - |
| 1KNM | 0.95 | 0.75 | 0.94 | 0.95 | 0.81 | 0.83 | 15 | 96 | 5 | 1 | 49/58 | 20/26 | 29/32 | - | - | - | - | - |
| 1QPK | 0.98 | 0.78 | 0.86 | 0.99 | 0.81 | 0.82 | 18 | 358 | 5 | 3 | 68/85 | 68/85 | - | - | - | - | - | - |
| 1Y4C | 0.98 | 0.67 | 1.00 | 0.98 | 0.81 | 0.80 | 16 | 422 | 8 | 0 | 57/59 | 57/59 | - | - | - | - | - | - |
| 2A2D | 0.98 | 0.71 | 0.94 | 0.98 | 0.81 | 0.81 | 17 | 365 | 7 | 1 | 48/53 | 48/53 | - | - | - | - | - | - |
| 2RJO | 0.98 | 0.67 | 1.00 | 0.98 | 0.81 | 0.80 | 14 | 279 | 7 | 0 | 45/47 | 45/47 | - | - | - | - | - | - |
| 1CZA | 0.98 | 0.80 | 0.83 | 0.99 | 0.80 | 0.81 | 43 | 726 | 11 | 9 | 105/133 | 45/55 | 9/12 | 51/66 | - | - | - | - |
| 1EHN | 0.97 | 0.96 | 0.69 | 1.00 | 0.80 | 0.81 | 27 | 440 | 1 | 12 | 102/157 | 80/107 | 22/30 | 0/20 | - | - | - | - |
| 1G1T | 0.97 | 1.00 | 0.67 | 1.00 | 0.80 | 0.80 | 8 | 131 | 0 | 4 | 14/25 | 14/25 | - | - | - | - | - | - |
| 1IS3 | 0.97 | 0.82 | 0.82 | 0.98 | 0.80 | 0.82 | 9 | 116 | 2 | 2 | 29/37 | 29/37 | - | - | - | - | - | - |
| 1KC3 | 0.99 | 0.73 | 0.89 | 0.99 | 0.80 | 0.80 | 8 | 260 | 3 | 1 | 21/32 | 21/32 | - | - | - | - | - | - |
| 1R87 | 0.97 | 0.69 | 0.95 | 0.97 | 0.80 | 0.80 | 18 | 298 | 8 | 1 | 74/75 | 74/75 | - | - | - | - | - | - |
| 2BVM | 0.99 | 0.65 | 1.00 | 0.99 | 0.80 | 0.79 | 13 | 474 | 7 | 0 | 30/33 | 30/33 | - | - | - | - | - | - |
| 2WMG | 0.98 | 0.72 | 0.90 | 0.99 | 0.80 | 0.80 | 18 | 460 | 7 | 2 | 55/63 | 31/33 | 24/30 | - | - | - | - | - |
| 3B9A | 0.97 | 0.80 | 0.82 | 0.99 | 0.80 | 0.81 | 28 | 449 | 7 | 6 | 107/130 | 29/39 | 52/62 | 26/29 | - | - | - | - |
| 3EHS | 0.98 | 0.65 | 1.00 | 0.98 | 0.80 | 0.79 | 15 | 410 | 8 | 0 | 60/62 | 47/48 | 13/14 | - | - | - | - | - |
| 3F9M | 0.98 | 0.70 | 0.94 | 0.98 | 0.80 | 0.80 | 16 | 369 | 7 | 1 | 37/40 | 37/40 | - | - | - | - | - | - |
| 3G7W | 0.98 | 0.77 | 0.85 | 0.99 | 0.80 | 0.81 | 17 | 340 | 5 | 3 | 61/82 | 25/39 | 36/43 | - | - | - | - | - |
| 3IM0 | 0.98 | 0.71 | 0.91 | 0.98 | 0.80 | 0.80 | 10 | 208 | 4 | 1 | 27/36 | 27/36 | - | - | - | - | - | - |
| 1Y65 | 0.98 | 0.80 | 0.80 | 0.99 | 0.79 | 0.80 | 12 | 269 | 3 | 3 | 22/33 | 22/33 | - | - | - | - | - | - |
| 2FNC | 0.97 | 0.64 | 1.00 | 0.97 | 0.79 | 0.78 | 18 | 309 | 10 | 0 | 72/76 | 72/76 | - | - | - | - | - | - |
| 2FVY | 0.97 | 0.64 | 1.00 | 0.97 | 0.79 | 0.78 | 14 | 245 | 8 | 0 | 49/49 | 49/49 | - | - | - | - | - | - |
| 2UVJ | 0.97 | 0.71 | 0.91 | 0.98 | 0.79 | 0.80 | 20 | 338 | 8 | 2 | 59/73 | 59/73 | - | - | - | - | - | - |
| 2YQS | 0.98 | 0.94 | 0.68 | 1.00 | 0.79 | 0.79 | 15 | 377 | 1 | 7 | 36/64 | 36/64 | - | - | - | - | - | - |
| 1E7Y | 0.99 | 0.69 | 0.90 | 0.99 | 0.78 | 0.78 | 9 | 438 | 4 | 1 | 19/25 | 19/25 | - | - | - | - | - | - |
| 1V03 | 0.98 | 0.63 | 1.00 | 0.98 | 0.78 | 0.77 | 15 | 404 | 9 | 0 | 41/41 | 41/41 | - | - | - | - | - | - |
| 2E2O | 0.97 | 0.63 | 1.00 | 0.96 | 0.78 | 0.77 | 15 | 233 | 9 | 0 | 41/41 | 41/41 | - | - | - | - | - | - |
| 2FHF | 0.99 | 0.77 | 0.81 | 0.99 | 0.78 | 0.79 | 26 | 924 | 8 | 6 | 93/131 | 23/29 | 45/61 | 19/28 | 6/13 | - | - | - |
| 2IW1 | 0.97 | 0.74 | 0.85 | 0.98 | 0.78 | 0.79 | 17 | 299 | 6 | 3 | 36/53 | 16/22 | 16/19 | 4/12 | - | - | - | - |
| 2VVS | 0.98 | 0.65 | 0.94 | 0.98 | 0.78 | 0.77 | 17 | 489 | 9 | 1 | 50/52 | 50/52 | - | - | - | - | - | - |
| 3CA3 | 0.97 | 0.93 | 0.68 | 1.00 | 0.78 | 0.79 | 13 | 215 | 1 | 6 | 47/72 | 28/38 | 19/34 | - | - | - | - | - |
| 3D4C | 0.98 | 0.63 | 1.00 | 0.98 | 0.78 | 0.77 | 15 | 426 | 9 | 0 | 59/61 | 59/61 | - | - | - | - | - | - |
| 3HL3 | 0.97 | 0.92 | 0.69 | 1.00 | 0.78 | 0.79 | 11 | 203 | 1 | 5 | 22/36 | 22/36 | - | - | - | - | - | - |
| 4A3H | 0.97 | 0.72 | 0.87 | 0.98 | 0.78 | 0.79 | 13 | 237 | 5 | 2 | 33/38 | 33/38 | - | - | - | - | - | - |
| 1URG | 0.98 | 0.65 | 0.93 | 0.98 | 0.77 | 0.77 | 13 | 330 | 7 | 1 | 52/53 | 52/53 | - | - | - | - | - | - |
| 2BOD | 0.97 | 0.75 | 0.83 | 0.98 | 0.77 | 0.79 | 15 | 217 | 5 | 3 | 57/69 | 57/69 | - | - | - | - | - | - |
| 2OSX | 0.98 | 0.65 | 0.94 | 0.98 | 0.77 | 0.77 | 15 | 393 | 8 | 1 | 60/65 | 32/33 | 28/32 | - | - | - | - | - |
| 2RJ7 | 0.97 | 0.77 | 0.81 | 0.98 | 0.77 | 0.79 | 17 | 243 | 5 | 4 | 56/73 | 35/45 | 21/28 | - | - | - | - | - |
| 2VCE | 0.98 | 0.72 | 0.86 | 0.98 | 0.77 | 0.78 | 18 | 383 | 7 | 3 | 47/57 | 40/43 | 7/14 | - | - | - | - | - |
| 2ZHN | 0.96 | 0.92 | 0.69 | 0.99 | 0.77 | 0.79 | 11 | 116 | 1 | 5 | 32/45 | 32/45 | - | - | - | - | - | - |
| 1B3Z | 0.97 | 0.88 | 0.68 | 0.99 | 0.76 | 0.77 | 15 | 241 | 2 | 7 | 50/88 | 0/24 | 50/64 | - | - | - | - | - |
| 1ELJ | 0.97 | 0.71 | 0.85 | 0.98 | 0.76 | 0.77 | 17 | 299 | 7 | 3 | 68/78 | 68/78 | - | - | - | - | - | - |
| 1GZ9 | 0.97 | 0.83 | 0.71 | 0.99 | 0.76 | 0.77 | 10 | 204 | 2 | 4 | 26/43 | 26/43 | - | - | - | - | - | - |
| 1KJR | 0.97 | 0.78 | 0.78 | 0.98 | 0.76 | 0.78 | 7 | 120 | 2 | 2 | 23/35 | 14/25 | 9/10 | - | - | - | - | - |
| 1LED | 0.97 | 1.00 | 0.60 | 1.00 | 0.76 | 0.75 | 9 | 208 | 0 | 6 | 29/64 | 29/64 | - | - | - | - | - | - |
| 1OUR | 0.96 | 1.00 | 0.60 | 1.00 | 0.76 | 0.75 | 6 | 92 | 0 | 4 | 14/29 | 14/29 | - | - | - | - | - | - |
| 1Q33 | 0.97 | 0.65 | 0.92 | 0.98 | 0.76 | 0.76 | 11 | 251 | 6 | 1 | 31/33 | 31/33 | - | - | - | - | - | - |
| 1UU6 | 0.94 | 0.68 | 0.91 | 0.94 | 0.76 | 0.78 | 21 | 167 | 10 | 2 | 64/73 | 56/56 | 8/17 | - | - | - | - | - |
| 1W8N | 0.98 | 0.68 | 0.88 | 0.98 | 0.76 | 0.76 | 21 | 507 | 10 | 3 | 61/67 | 25/29 | 23/24 | 13/14 | - | - | - | - |
| 2IT6 | 0.97 | 1.00 | 0.60 | 1.00 | 0.76 | 0.75 | 6 | 113 | 0 | 4 | 13/29 | 13/29 | - | - | - | - | - | - |
| 2P2V | 0.97 | 0.76 | 0.80 | 0.98 | 0.76 | 0.78 | 16 | 230 | 5 | 4 | 51/68 | 51/68 | - | - | - | - | - | - |
| 2QQW | 0.98 | 0.67 | 0.88 | 0.99 | 0.76 | 0.76 | 14 | 454 | 7 | 2 | 49/53 | 49/53 | - | - | - | - | - | - |
| 2VGQ | 0.98 | 0.77 | 0.77 | 0.99 | 0.76 | 0.77 | 17 | 396 | 5 | 5 | 63/96 | 31/54 | 32/42 | - | - | - | - | - |
| 2ZYN | 0.97 | 0.89 | 0.67 | 0.99 | 0.76 | 0.76 | 16 | 320 | 2 | 8 | 64/97 | 39/59 | 25/38 | - | - | - | - | - |
| 1GZ1 | 0.95 | 0.65 | 0.92 | 0.96 | 0.75 | 0.76 | 24 | 285 | 13 | 2 | 82/97 | 74/87 | 8/10 | - | - | - | - | - |
| 1RWG | 0.98 | 0.61 | 0.95 | 0.98 | 0.75 | 0.74 | 20 | 651 | 13 | 1 | 72/77 | 72/77 | - | - | - | - | - | - |
| 1Z4X | 0.98 | 0.91 | 0.63 | 1.00 | 0.75 | 0.74 | 10 | 392 | 1 | 6 | 28/47 | 23/36 | 5/11 | - | - | - | - | - |
| 2HTQ | 0.98 | 0.91 | 0.63 | 1.00 | 0.75 | 0.74 | 10 | 340 | 1 | 6 | 27/52 | 27/52 | - | - | - | - | - | - |
| 2OEG | 0.99 | 1.00 | 0.57 | 1.00 | 0.75 | 0.73 | 8 | 441 | 0 | 6 | 25/39 | 25/39 | - | - | - | - | - | - |
| 2WHM | 0.97 | 0.58 | 1.00 | 0.97 | 0.75 | 0.74 | 14 | 302 | 10 | 0 | 41/41 | 41/41 | - | - | - | - | - | - |
| 3CUJ | 0.97 | 0.77 | 0.77 | 0.99 | 0.75 | 0.77 | 13 | 262 | 4 | 4 | 49/63 | 33/39 | 16/24 | - | - | - | - | - |
| 7TAA | 0.98 | 0.85 | 0.68 | 0.99 | 0.75 | 0.76 | 17 | 406 | 3 | 8 | 58/93 | 41/59 | 17/34 | - | - | - | - | - |
| 1EUS | 0.98 | 0.71 | 0.80 | 0.98 | 0.74 | 0.75 | 12 | 297 | 5 | 3 | 33/42 | 33/42 | - | - | - | - | - | - |
| 1GNY | 0.97 | 0.73 | 0.80 | 0.98 | 0.74 | 0.76 | 8 | 128 | 3 | 2 | 37/43 | 37/43 | - | - | - | - | - | - |
| 2GH4 | 0.96 | 0.64 | 0.89 | 0.97 | 0.74 | 0.74 | 16 | 282 | 9 | 2 | 39/42 | 39/42 | - | - | - | - | - | - |
| 2GQU | 0.97 | 0.79 | 0.73 | 0.99 | 0.74 | 0.76 | 11 | 229 | 3 | 4 | 25/52 | 19/39 | 6/13 | - | - | - | - | - |
| 1V8R | 0.96 | 1.00 | 0.55 | 1.00 | 0.73 | 0.71 | 6 | 128 | 0 | 5 | 12/27 | 12/27 | - | - | - | - | - | - |
| 2C1Z | 0.97 | 0.59 | 0.94 | 0.97 | 0.73 | 0.73 | 16 | 375 | 11 | 1 | 51/59 | 31/39 | 20/20 | - | - | - | - | - |
| 2JEN | 0.94 | 0.91 | 0.65 | 0.99 | 0.73 | 0.76 | 20 | 166 | 2 | 11 | 70/118 | 70/107 | 0/11 | - | - | - | - | - |
| 1BDG | 0.97 | 0.54 | 1.00 | 0.97 | 0.72 | 0.70 | 14 | 333 | 12 | 0 | 34/36 | 34/36 | - | - | - | - | - | - |
| 1EOM | 0.96 | 0.82 | 0.67 | 0.99 | 0.72 | 0.74 | 14 | 213 | 3 | 7 | 44/72 | 42/50 | 2/22 | - | - | - | - | - |
| 1F9D | 0.95 | 0.69 | 0.80 | 0.97 | 0.72 | 0.74 | 37 | 485 | 17 | 9 | 137/169 | 101/122 | 36/47 | - | - | - | - | - |
| 1FCV | 0.97 | 0.70 | 0.78 | 0.98 | 0.72 | 0.74 | 14 | 271 | 6 | 4 | 46/59 | 35/41 | 11/18 | - | - | - | - | - |
| 1KWK | 0.98 | 0.52 | 1.00 | 0.98 | 0.72 | 0.69 | 12 | 570 | 11 | 0 | 37/37 | 37/37 | - | - | - | - | - | - |
| 2C4D | 0.93 | 0.85 | 0.66 | 0.98 | 0.72 | 0.75 | 35 | 303 | 6 | 18 | 100/187 | 14/37 | 44/61 | 19/31 | 13/23 | 10/35 | - | - |
| 2V0I | 0.98 | 1.00 | 0.53 | 1.00 | 0.72 | 0.69 | 9 | 397 | 0 | 8 | 22/51 | 22/51 | - | - | - | - | - | - |
| 2W47 | 0.96 | 1.00 | 0.55 | 1.00 | 0.72 | 0.71 | 6 | 111 | 0 | 5 | 22/32 | 22/32 | - | - | - | - | - | - |
| 1CEN | 0.96 | 0.52 | 1.00 | 0.96 | 0.71 | 0.69 | 12 | 264 | 11 | 0 | 50/54 | 50/54 | - | - | - | - | - | - |
| 1G9R | 0.96 | 0.57 | 0.93 | 0.96 | 0.71 | 0.70 | 13 | 235 | 10 | 1 | 34/39 | 34/39 | - | - | - | - | - | - |
| 1GU3 | 0.96 | 0.80 | 0.67 | 0.98 | 0.71 | 0.73 | 8 | 124 | 2 | 4 | 35/53 | 35/53 | - | - | - | - | - | - |
| 1KCD | 0.98 | 0.82 | 0.64 | 0.99 | 0.71 | 0.72 | 9 | 284 | 2 | 5 | 19/42 | 18/31 | 1/11 | - | - | - | - | - |
| 1M03 | 0.97 | 0.52 | 1.00 | 0.97 | 0.71 | 0.68 | 15 | 418 | 14 | 0 | 52/53 | 52/53 | - | - | - | - | - | - |
| 1N1T | 0.98 | 0.58 | 0.88 | 0.98 | 0.71 | 0.70 | 14 | 530 | 10 | 2 | 40/46 | 40/46 | - | - | - | - | - | - |
| 1PNF | 0.96 | 0.52 | 1.00 | 0.96 | 0.71 | 0.68 | 13 | 260 | 12 | 0 | 43/46 | 43/46 | - | - | - | - | - | - |
| 1S5M | 0.97 | 0.52 | 1.00 | 0.97 | 0.71 | 0.68 | 13 | 329 | 12 | 0 | 38/39 | 38/39 | - | - | - | - | - | - |
| 1T0O | 0.96 | 0.52 | 1.00 | 0.96 | 0.71 | 0.68 | 15 | 345 | 14 | 0 | 44/45 | 44/45 | - | - | - | - | - | - |
| 1W0O | 0.97 | 0.59 | 0.90 | 0.97 | 0.71 | 0.71 | 27 | 639 | 19 | 3 | 66/84 | 24/27 | 20/23 | 22/34 | - | - | - | - |
| 2E9M | 0.97 | 0.57 | 0.93 | 0.98 | 0.71 | 0.70 | 13 | 384 | 10 | 1 | 33/34 | 33/34 | - | - | - | - | - | - |
| 2J44 | 0.96 | 0.67 | 0.80 | 0.97 | 0.71 | 0.73 | 12 | 190 | 6 | 3 | 52/68 | 20/34 | 32/34 | - | - | - | - | - |
| 2VW1 | 0.98 | 0.63 | 0.83 | 0.98 | 0.71 | 0.71 | 15 | 569 | 9 | 3 | 34/42 | 34/42 | - | - | - | - | - | - |
| 2WAO | 0.97 | 0.80 | 0.67 | 0.99 | 0.71 | 0.73 | 12 | 268 | 3 | 6 | 54/79 | 54/79 | - | - | - | - | - | - |
| 2Z1S | 0.97 | 0.73 | 0.73 | 0.98 | 0.71 | 0.73 | 19 | 367 | 7 | 7 | 47/69 | 11/20 | 35/37 | 1/12 | - | - | - | - |
| 3FIZ | 0.97 | 0.56 | 0.93 | 0.97 | 0.71 | 0.70 | 14 | 371 | 11 | 1 | 32/34 | 32/34 | - | - | - | - | - | - |
| 3GH5 | 0.98 | 0.57 | 0.93 | 0.98 | 0.71 | 0.70 | 13 | 419 | 10 | 1 | 47/48 | 47/48 | - | - | - | - | - | - |
| 1B1Y | 0.96 | 0.61 | 0.85 | 0.96 | 0.70 | 0.71 | 23 | 405 | 15 | 4 | 56/76 | 56/76 | - | - | - | - | - | - |
| 1E6X | 0.98 | 0.55 | 0.92 | 0.98 | 0.70 | 0.69 | 11 | 409 | 9 | 1 | 34/37 | 34/37 | - | - | - | - | - | - |
| 1QBB | 0.98 | 0.53 | 0.94 | 0.98 | 0.70 | 0.68 | 16 | 733 | 14 | 1 | 63/70 | 63/70 | - | - | - | - | - | - |
| 2IT5 | 0.96 | 0.86 | 0.60 | 0.99 | 0.70 | 0.71 | 6 | 111 | 1 | 4 | 14/24 | 14/24 | - | - | - | - | - | - |
| 3CKQ | 0.97 | 0.57 | 0.89 | 0.98 | 0.70 | 0.70 | 8 | 255 | 6 | 1 | 22/33 | 11/18 | 11/15 | - | - | - | - | - |
| 3DM0 | 0.98 | 0.50 | 1.00 | 0.97 | 0.70 | 0.67 | 15 | 571 | 15 | 0 | 57/59 | 37/37 | 20/22 | - | - | - | - | - |
| 1G97 | 0.97 | 0.82 | 0.61 | 0.99 | 0.69 | 0.70 | 14 | 382 | 3 | 9 | 29/56 | 29/56 | - | - | - | - | - | - |
| 1INV | 0.97 | 0.71 | 0.71 | 0.99 | 0.69 | 0.71 | 12 | 337 | 5 | 5 | 35/50 | 35/50 | - | - | - | - | - | - |
| 1IUC | 0.94 | 0.68 | 0.77 | 0.96 | 0.69 | 0.72 | 23 | 250 | 11 | 7 | 65/84 | 19/30 | 46/54 | - | - | - | - | - |
| 1Q6D | 0.96 | 0.71 | 0.71 | 0.98 | 0.69 | 0.71 | 22 | 412 | 9 | 9 | 58/90 | 46/77 | 12/13 | - | - | - | - | - |
| 3BYN | 0.97 | 0.60 | 0.83 | 0.97 | 0.69 | 0.70 | 15 | 362 | 10 | 3 | 48/59 | 48/59 | - | - | - | - | - | - |
| 5CGT | 0.97 | 0.63 | 0.79 | 0.98 | 0.69 | 0.70 | 22 | 579 | 13 | 6 | 86/105 | 29/35 | 31/35 | 26/35 | - | - | - | - |
| 1RQ5 | 0.97 | 0.63 | 0.76 | 0.98 | 0.68 | 0.69 | 19 | 487 | 11 | 6 | 79/95 | 79/95 | - | - | - | - | - | - |
| 1UXY | 0.97 | 0.77 | 0.63 | 0.99 | 0.68 | 0.69 | 10 | 290 | 3 | 6 | 28/42 | 28/42 | - | - | - | - | - | - |
| 2YVW | 0.97 | 0.60 | 0.80 | 0.98 | 0.68 | 0.69 | 12 | 357 | 8 | 3 | 30/39 | 30/39 | - | - | - | - | - | - |
| 1LU1 | 0.97 | 1.00 | 0.46 | 1.00 | 0.67 | 0.63 | 6 | 216 | 0 | 7 | 12/39 | 12/39 | - | - | - | - | - | - |
| 1PMH | 0.95 | 0.64 | 0.75 | 0.97 | 0.67 | 0.69 | 9 | 155 | 5 | 3 | 42/54 | 42/54 | - | - | - | - | - | - |
| 1QHO | 0.96 | 0.75 | 0.63 | 0.98 | 0.67 | 0.68 | 27 | 569 | 9 | 16 | 104/156 | 59/81 | 6/31 | 10/12 | 29/32 | - | - | - |
| 1VST | 0.97 | 0.83 | 0.56 | 0.99 | 0.67 | 0.67 | 5 | 169 | 1 | 4 | 10/25 | 10/25 | - | - | - | - | - | - |
| 2CGL | 0.97 | 0.46 | 1.00 | 0.97 | 0.67 | 0.63 | 13 | 408 | 15 | 0 | 41/41 | 41/41 | - | - | - | - | - | - |
| 2CIR | 0.96 | 0.48 | 1.00 | 0.96 | 0.67 | 0.65 | 10 | 234 | 11 | 0 | 31/33 | 31/33 | - | - | - | - | - | - |
| 3GNP | 0.97 | 0.50 | 0.93 | 0.97 | 0.67 | 0.65 | 13 | 399 | 13 | 1 | 38/39 | 38/39 | - | - | - | - | - | - |
| 1PIE | 0.96 | 0.57 | 0.80 | 0.97 | 0.66 | 0.67 | 12 | 314 | 9 | 3 | 28/40 | 28/40 | - | - | - | - | - | - |
| 1UY4 | 0.94 | 1.00 | 0.46 | 1.00 | 0.66 | 0.63 | 6 | 105 | 0 | 7 | 25/52 | 25/52 | - | - | - | - | - | - |
| 2AEZ | 0.97 | 0.48 | 0.93 | 0.97 | 0.66 | 0.64 | 14 | 451 | 15 | 1 | 45/46 | 45/46 | - | - | - | - | - | - |
| 2BS5 | 0.87 | 0.88 | 0.61 | 0.97 | 0.66 | 0.72 | 14 | 61 | 2 | 9 | 41/70 | 31/38 | 10/32 | - | - | - | - | - |
| 2EAE | 0.98 | 0.44 | 1.00 | 0.98 | 0.66 | 0.61 | 15 | 769 | 19 | 0 | 58/60 | 58/60 | - | - | - | - | - | - |
| 3F5F | 0.97 | 0.48 | 0.94 | 0.97 | 0.66 | 0.64 | 15 | 569 | 16 | 1 | 63/66 | 63/66 | - | - | - | - | - | - |
| 1GUI | 0.93 | 0.65 | 0.73 | 0.95 | 0.65 | 0.69 | 11 | 124 | 6 | 4 | 48/61 | 39/40 | 9/21 | - | - | - | - | - |
| 1HKK | 0.94 | 0.52 | 0.90 | 0.95 | 0.65 | 0.65 | 17 | 285 | 16 | 2 | 77/89 | 52/54 | 25/35 | - | - | - | - | - |
| 1J84 | 0.96 | 0.75 | 0.60 | 0.99 | 0.65 | 0.67 | 6 | 146 | 2 | 4 | 24/38 | 19/27 | 5/11 | - | - | - | - | - |
| 1KQY | 0.94 | 0.64 | 0.73 | 0.96 | 0.65 | 0.68 | 16 | 200 | 9 | 6 | 54/86 | 28/53 | 19/19 | 7/14 | - | - | - | - |
| 2CHH | 0.94 | 1.00 | 0.46 | 1.00 | 0.65 | 0.63 | 5 | 89 | 0 | 6 | 14/33 | 14/33 | - | - | - | - | - | - |
| 3E6J | 0.96 | 0.73 | 0.62 | 0.98 | 0.65 | 0.67 | 8 | 190 | 3 | 5 | 37/53 | 37/53 | - | - | - | - | - | - |
| 4RHN | 0.94 | 1.00 | 0.46 | 1.00 | 0.65 | 0.63 | 5 | 91 | 0 | 6 | 12/29 | 12/29 | - | - | - | - | - | - |
| 1GWM | 0.95 | 1.00 | 0.43 | 1.00 | 0.64 | 0.60 | 6 | 131 | 0 | 8 | 15/65 | 11/35 | 4/30 | - | - | - | - | - |
| 1HM2 | 0.97 | 0.46 | 0.94 | 0.97 | 0.64 | 0.61 | 15 | 567 | 18 | 1 | 66/73 | 66/73 | - | - | - | - | - | - |
| 1UA7 | 0.96 | 0.56 | 0.78 | 0.97 | 0.64 | 0.65 | 14 | 353 | 11 | 4 | 39/59 | 39/59 | - | - | - | - | - | - |
| 1UAE | 0.97 | 0.57 | 0.75 | 0.98 | 0.64 | 0.65 | 12 | 344 | 9 | 4 | 31/51 | 31/51 | - | - | - | - | - | - |
| 1Y9G | 0.97 | 0.42 | 1.00 | 0.97 | 0.64 | 0.60 | 11 | 439 | 15 | 0 | 38/39 | 38/39 | - | - | - | - | - | - |
| 3H4I | 0.96 | 0.57 | 0.77 | 0.97 | 0.64 | 0.65 | 13 | 331 | 10 | 4 | 37/56 | 37/56 | - | - | - | - | - | - |
| 4ENG | 0.92 | 0.88 | 0.52 | 0.99 | 0.64 | 0.65 | 14 | 164 | 2 | 13 | 33/88 | 27/66 | 6/22 | - | - | - | - | - |
| 1A9T | 0.96 | 0.56 | 0.75 | 0.97 | 0.63 | 0.64 | 9 | 239 | 7 | 3 | 25/38 | 25/38 | - | - | - | - | - | - |
| 1F0P | 0.94 | 0.87 | 0.50 | 0.99 | 0.63 | 0.63 | 13 | 222 | 2 | 13 | 27/82 | 25/47 | 2/35 | - | - | - | - | - |
| 1LZC | 0.92 | 1.00 | 0.44 | 1.00 | 0.63 | 0.61 | 7 | 103 | 0 | 9 | 19/54 | 19/54 | - | - | - | - | - | - |
| 1SLI | 0.97 | 0.49 | 0.84 | 0.97 | 0.63 | 0.62 | 16 | 587 | 17 | 3 | 36/40 | 36/40 | - | - | - | - | - | - |
| 1URX | 0.91 | 0.64 | 0.72 | 0.94 | 0.63 | 0.68 | 23 | 205 | 13 | 9 | 82/107 | 28/48 | 54/59 | - | - | - | - | - |
| 1UWF | 0.95 | 1.00 | 0.42 | 1.00 | 0.63 | 0.59 | 5 | 135 | 0 | 7 | 13/35 | 13/35 | - | - | - | - | - | - |
| 1WD4 | 0.95 | 0.64 | 0.68 | 0.97 | 0.63 | 0.66 | 21 | 384 | 12 | 10 | 58/100 | 20/31 | 9/34 | 29/35 | - | - | - | - |
| 1ZDG | 0.95 | 0.47 | 0.90 | 0.96 | 0.63 | 0.62 | 9 | 215 | 10 | 1 | 24/27 | 24/27 | - | - | - | - | - | - |
| 2BF6 | 0.96 | 0.48 | 0.88 | 0.96 | 0.63 | 0.63 | 15 | 363 | 16 | 2 | 45/48 | 45/48 | - | - | - | - | - | - |
| 2HW1 | 0.94 | 0.46 | 0.92 | 0.95 | 0.63 | 0.62 | 12 | 239 | 14 | 1 | 31/36 | 31/36 | - | - | - | - | - | - |
| 3B50 | 0.95 | 0.60 | 0.71 | 0.97 | 0.63 | 0.65 | 12 | 256 | 8 | 5 | 31/53 | 31/53 | - | - | - | - | - | - |
| 3C2V | 0.97 | 0.57 | 0.73 | 0.98 | 0.63 | 0.64 | 8 | 237 | 6 | 3 | 12/24 | 12/24 | - | - | - | - | - | - |
| 3CZK | 0.96 | 0.44 | 0.94 | 0.96 | 0.63 | 0.60 | 17 | 528 | 22 | 1 | 52/56 | 52/56 | - | - | - | - | - | - |
| 1BYH | 0.92 | 0.46 | 0.93 | 0.92 | 0.62 | 0.62 | 13 | 171 | 15 | 1 | 39/46 | 39/46 | - | - | - | - | - | - |
| 1G0C | 0.96 | 0.45 | 0.90 | 0.96 | 0.62 | 0.60 | 9 | 292 | 11 | 1 | 29/36 | 29/36 | - | - | - | - | - | - |
| 1GOQ | 0.94 | 0.81 | 0.52 | 0.99 | 0.62 | 0.63 | 13 | 237 | 3 | 12 | 49/96 | 49/74 | 0/22 | - | - | - | - | - |
| 1INW | 0.97 | 0.75 | 0.53 | 0.99 | 0.62 | 0.62 | 9 | 332 | 3 | 8 | 19/48 | 19/48 | - | - | - | - | - | - |
| 1K1W | 0.97 | 0.43 | 0.92 | 0.97 | 0.62 | 0.59 | 12 | 532 | 16 | 1 | 34/40 | 34/40 | - | - | - | - | - | - |
| 1LOH | 0.97 | 0.61 | 0.67 | 0.98 | 0.62 | 0.64 | 20 | 616 | 13 | 10 | 68/106 | 48/53 | 10/10 | 8/28 | 2/15 | - | - | - |
| 1O03 | 0.94 | 0.63 | 0.67 | 0.97 | 0.62 | 0.65 | 10 | 175 | 6 | 5 | 29/40 | 29/40 | - | - | - | - | - | - |
| 2C27 | 0.96 | 0.52 | 0.79 | 0.96 | 0.62 | 0.63 | 11 | 264 | 10 | 3 | 26/35 | 26/35 | - | - | - | - | - | - |
| 2EQD | 0.95 | 0.54 | 0.76 | 0.96 | 0.62 | 0.63 | 19 | 417 | 16 | 6 | 69/101 | 16/30 | 33/41 | 20/30 | - | - | - | - |
| 2HTW | 0.97 | 0.80 | 0.50 | 0.99 | 0.62 | 0.62 | 8 | 332 | 2 | 8 | 21/47 | 21/47 | - | - | - | - | - | - |
| 2WRA | 0.95 | 1.00 | 0.40 | 1.00 | 0.62 | 0.57 | 4 | 102 | 0 | 6 | 14/39 | 14/39 | - | - | - | - | - | - |
| 2ZAA | 0.92 | 0.79 | 0.56 | 0.98 | 0.62 | 0.65 | 15 | 178 | 4 | 12 | 35/73 | 21/47 | 14/26 | - | - | - | - | - |
| 3DJ4 | 0.97 | 0.85 | 0.48 | 1.00 | 0.62 | 0.61 | 11 | 403 | 2 | 12 | 26/60 | 26/60 | - | - | - | - | - | - |
| 4PFK | 0.97 | 0.60 | 0.67 | 0.98 | 0.62 | 0.63 | 6 | 254 | 4 | 3 | 21/32 | 21/32 | - | - | - | - | - | - |
| 1Y3P | 0.94 | 0.49 | 0.84 | 0.95 | 0.61 | 0.62 | 21 | 405 | 22 | 4 | 69/87 | 51/51 | 18/36 | - | - | - | - | - |
| 2G3J | 0.95 | 0.55 | 0.73 | 0.97 | 0.61 | 0.63 | 11 | 250 | 9 | 4 | 43/63 | 43/63 | - | - | - | - | - | - |
| 2QKX | 0.97 | 0.56 | 0.71 | 0.98 | 0.61 | 0.63 | 10 | 323 | 8 | 4 | 23/37 | 23/37 | - | - | - | - | - | - |
| 2V8K | 0.97 | 0.50 | 0.79 | 0.98 | 0.61 | 0.61 | 11 | 470 | 11 | 3 | 30/45 | 30/45 | - | - | - | - | - | - |
| 2VCB | 0.96 | 0.38 | 1.00 | 0.96 | 0.61 | 0.55 | 18 | 752 | 29 | 0 | 48/49 | 48/49 | - | - | - | - | - | - |
| 2VJJ | 0.96 | 0.73 | 0.54 | 0.99 | 0.61 | 0.62 | 19 | 493 | 7 | 16 | 49/108 | 30/40 | 13/54 | 6/14 | - | - | - | - |
| 3BC9 | 0.95 | 0.71 | 0.56 | 0.98 | 0.61 | 0.63 | 22 | 487 | 9 | 17 | 99/178 | 27/42 | 37/42 | 34/44 | 1/50 | - | - | - |
| 1OH4 | 0.92 | 0.53 | 0.77 | 0.94 | 0.60 | 0.63 | 10 | 134 | 9 | 3 | 42/57 | 31/37 | 11/20 | - | - | - | - | - |
| 1P5G | 0.95 | 0.38 | 1.00 | 0.95 | 0.60 | 0.55 | 12 | 353 | 20 | 0 | 32/32 | 32/32 | - | - | - | - | - | - |
| 2ACQ | 0.96 | 0.42 | 0.89 | 0.96 | 0.60 | 0.57 | 8 | 262 | 11 | 1 | 26/28 | 26/28 | - | - | - | - | - | - |
| 2DEJ | 0.95 | 0.50 | 0.79 | 0.96 | 0.60 | 0.61 | 11 | 266 | 11 | 3 | 30/40 | 30/40 | - | - | - | - | - | - |
| 2IHJ | 0.95 | 0.56 | 0.70 | 0.97 | 0.60 | 0.62 | 14 | 323 | 11 | 6 | 35/58 | 35/58 | - | - | - | - | - | - |
| 2RL2 | 0.95 | 0.50 | 0.77 | 0.96 | 0.60 | 0.61 | 13 | 335 | 13 | 4 | 39/58 | 28/28 | 11/30 | - | - | - | - | - |
| 2W5O | 0.95 | 0.38 | 1.00 | 0.95 | 0.60 | 0.55 | 9 | 301 | 15 | 0 | 31/31 | 31/31 | - | - | - | - | - | - |
| 1L8N | 0.96 | 0.46 | 0.81 | 0.97 | 0.59 | 0.59 | 17 | 552 | 20 | 4 | 66/76 | 66/76 | - | - | - | - | - | - |
| 1LMQ | 0.90 | 0.65 | 0.65 | 0.94 | 0.59 | 0.65 | 11 | 95 | 6 | 6 | 34/56 | 34/56 | - | - | - | - | - | - |
| 1ULV | 0.98 | 0.41 | 0.86 | 0.98 | 0.59 | 0.56 | 12 | 894 | 17 | 2 | 37/43 | 37/43 | - | - | - | - | - | - |
| 1W9W | 0.92 | 0.78 | 0.50 | 0.98 | 0.59 | 0.61 | 7 | 103 | 2 | 7 | 32/59 | 15/41 | 17/18 | - | - | - | - | - |
| 2H44 | 0.96 | 0.50 | 0.75 | 0.97 | 0.59 | 0.60 | 9 | 280 | 9 | 3 | 24/34 | 24/34 | - | - | - | - | - | - |
| 2QIA | 0.97 | 1.00 | 0.36 | 1.00 | 0.59 | 0.53 | 4 | 224 | 0 | 7 | 8/41 | 8/41 | - | - | - | - | - | - |
| 3C7G | 0.96 | 0.44 | 0.85 | 0.97 | 0.59 | 0.58 | 11 | 396 | 14 | 2 | 38/50 | 23/23 | 15/27 | - | - | - | - | - |
| 1V0C | 0.95 | 0.86 | 0.43 | 0.99 | 0.58 | 0.57 | 6 | 149 | 1 | 8 | 33/73 | 33/73 | - | - | - | - | - | - |
| 2D0G | 0.93 | 0.71 | 0.54 | 0.98 | 0.58 | 0.61 | 30 | 504 | 12 | 26 | 120/230 | 14/50 | 19/44 | 8/24 | 38/49 | 20/22 | 21/27 | 0/14 |
| 2PHH | 0.96 | 0.35 | 1.00 | 0.96 | 0.58 | 0.52 | 8 | 344 | 15 | 0 | 24/27 | 24/27 | - | - | - | - | - | - |
| 1ESW | 0.95 | 0.60 | 0.60 | 0.97 | 0.57 | 0.60 | 18 | 407 | 12 | 12 | 65/115 | 29/51 | 11/33 | 25/31 | - | - | - | - |
| 1JC9 | 0.95 | 0.50 | 0.70 | 0.96 | 0.57 | 0.58 | 7 | 181 | 7 | 3 | 23/31 | 23/31 | - | - | - | - | - | - |
| 1RKD | 0.95 | 0.47 | 0.75 | 0.96 | 0.57 | 0.58 | 9 | 248 | 10 | 3 | 26/33 | 26/33 | - | - | - | - | - | - |
| 1UX7 | 0.96 | 1.00 | 0.33 | 1.00 | 0.57 | 0.50 | 2 | 100 | 0 | 4 | 13/30 | 13/30 | - | - | - | - | - | - |
| 1WU5 | 0.95 | 0.35 | 1.00 | 0.95 | 0.57 | 0.51 | 9 | 300 | 17 | 0 | 32/33 | 32/33 | - | - | - | - | - | - |
| 2B4F | 0.93 | 0.68 | 0.53 | 0.97 | 0.57 | 0.60 | 17 | 300 | 8 | 15 | 64/125 | 43/54 | 8/29 | 0/23 | 13/19 | - | - | - |
| 2POQ | 0.97 | 0.46 | 0.75 | 0.98 | 0.57 | 0.57 | 6 | 276 | 7 | 2 | 20/31 | 20/31 | - | - | - | - | - | - |
| 1GAI | 0.96 | 0.44 | 0.77 | 0.97 | 0.56 | 0.56 | 10 | 404 | 13 | 3 | 37/55 | 37/55 | - | - | - | - | - | - |
| 1ITC | 0.92 | 0.79 | 0.46 | 0.98 | 0.56 | 0.58 | 27 | 412 | 7 | 32 | 80/198 | 49/90 | 14/34 | 0/45 | 17/29 | - | - | - |
| 1LSZ | 0.91 | 1.00 | 0.35 | 1.00 | 0.56 | 0.52 | 6 | 101 | 0 | 11 | 16/53 | 16/53 | - | - | - | - | - | - |
| 1LZR | 0.91 | 0.73 | 0.50 | 0.97 | 0.56 | 0.59 | 8 | 104 | 3 | 8 | 33/59 | 7/23 | 26/36 | - | - | - | - | - |
| 1Z45 | 0.97 | 0.54 | 0.62 | 0.98 | 0.56 | 0.58 | 13 | 571 | 11 | 8 | 36/63 | 1/26 | 35/37 | - | - | - | - | - |
| 2IXB | 0.95 | 0.39 | 0.85 | 0.95 | 0.56 | 0.54 | 11 | 353 | 17 | 2 | 20/30 | 10/15 | 10/15 | - | - | - | - | - |
| 2WHL | 0.94 | 0.38 | 0.90 | 0.94 | 0.56 | 0.53 | 9 | 221 | 15 | 1 | 38/49 | 38/49 | - | - | - | - | - | - |
| 3INA | 0.95 | 0.90 | 0.38 | 1.00 | 0.56 | 0.53 | 9 | 312 | 1 | 15 | 21/87 | 15/49 | 6/38 | - | - | - | - | - |
| 1CNQ | 0.94 | 0.67 | 0.50 | 0.98 | 0.55 | 0.57 | 12 | 271 | 6 | 12 | 29/62 | 29/37 | 0/25 | - | - | - | - | - |
| 1G94 | 0.96 | 0.44 | 0.73 | 0.97 | 0.55 | 0.55 | 11 | 385 | 14 | 4 | 39/57 | 39/57 | - | - | - | - | - | - |
| 1XC6 | 0.97 | 0.32 | 1.00 | 0.97 | 0.55 | 0.48 | 13 | 863 | 28 | 0 | 35/36 | 35/36 | - | - | - | - | - | - |
| 2JEQ | 0.94 | 0.52 | 0.63 | 0.96 | 0.55 | 0.57 | 12 | 293 | 11 | 7 | 42/71 | 36/52 | 6/19 | - | - | - | - | - |
| 1XEZ | 0.97 | 0.38 | 0.82 | 0.97 | 0.54 | 0.51 | 9 | 557 | 15 | 2 | 21/32 | 21/32 | - | - | - | - | - | - |
| 2BS7 | 0.93 | 0.47 | 0.70 | 0.95 | 0.54 | 0.56 | 7 | 146 | 8 | 3 | 25/39 | 25/39 | - | - | - | - | - | - |
| 1IA7 | 0.95 | 0.30 | 1.00 | 0.95 | 0.53 | 0.46 | 8 | 346 | 19 | 0 | 37/37 | 37/37 | - | - | - | - | - | - |
| 1TZF | 0.98 | 1.00 | 0.29 | 1.00 | 0.53 | 0.44 | 2 | 220 | 0 | 5 | 9/25 | 9/25 | - | - | - | - | - | - |
| 1X1J | 0.96 | 0.29 | 1.00 | 0.96 | 0.53 | 0.45 | 11 | 632 | 27 | 0 | 38/38 | 38/38 | - | - | - | - | - | - |
| 1Z3W | 0.94 | 0.31 | 1.00 | 0.93 | 0.53 | 0.47 | 11 | 347 | 25 | 0 | 40/40 | 40/40 | - | - | - | - | - | - |
| 2WNB | 0.96 | 0.50 | 0.60 | 0.98 | 0.53 | 0.55 | 6 | 236 | 6 | 4 | 17/38 | 17/38 | - | - | - | - | - | - |
| 3B8A | 0.96 | 0.53 | 0.56 | 0.98 | 0.53 | 0.55 | 9 | 393 | 8 | 7 | 23/42 | 23/42 | - | - | - | - | - | - |
| 3CZN | 0.97 | 0.53 | 0.55 | 0.99 | 0.53 | 0.54 | 16 | 890 | 14 | 13 | 56/101 | 53/69 | 3/32 | - | - | - | - | - |
| 1D0M | 0.94 | 0.65 | 0.48 | 0.98 | 0.52 | 0.55 | 11 | 246 | 6 | 12 | 37/81 | 2/32 | 35/49 | - | - | - | - | - |
| 1SX6 | 0.97 | 0.75 | 0.38 | 1.00 | 0.52 | 0.50 | 3 | 187 | 1 | 5 | 15/34 | 15/34 | - | - | - | - | - | - |
| 1U8X | 0.97 | 0.38 | 0.75 | 0.97 | 0.52 | 0.50 | 6 | 382 | 10 | 2 | 22/27 | 22/27 | - | - | - | - | - | - |
| 2BFQ | 0.95 | 0.71 | 0.42 | 0.99 | 0.52 | 0.53 | 5 | 156 | 2 | 7 | 9/42 | 9/42 | - | - | - | - | - | - |
| 2W52 | 0.92 | 0.46 | 0.68 | 0.94 | 0.52 | 0.55 | 13 | 228 | 15 | 6 | 50/73 | 50/73 | - | - | - | - | - | - |
| 3FAX | 0.97 | 0.31 | 0.90 | 0.97 | 0.52 | 0.46 | 9 | 668 | 20 | 1 | 34/41 | 34/41 | - | - | - | - | - | - |
| 6CEL | 0.91 | 0.65 | 0.50 | 0.96 | 0.52 | 0.57 | 24 | 337 | 13 | 24 | 89/183 | 39/59 | 50/64 | 0/60 | - | - | - | - |
| 1G9F | 0.96 | 1.00 | 0.27 | 1.00 | 0.51 | 0.43 | 3 | 196 | 0 | 8 | 9/31 | 9/31 | - | - | - | - | - | - |
| 1ZU0 | 0.93 | 0.34 | 0.84 | 0.93 | 0.51 | 0.49 | 16 | 417 | 31 | 3 | 60/82 | 60/82 | - | - | - | - | - | - |
| 2VUZ | 0.89 | 0.53 | 0.62 | 0.93 | 0.51 | 0.57 | 8 | 91 | 7 | 5 | 22/49 | 15/39 | 7/10 | - | - | - | - | - |
| 1MWE | 0.94 | 0.73 | 0.38 | 0.99 | 0.50 | 0.50 | 11 | 328 | 4 | 18 | 32/85 | 32/47 | 0/38 | - | - | - | - | - |
| 2DWP | 0.94 | 0.48 | 0.60 | 0.96 | 0.50 | 0.54 | 15 | 358 | 16 | 10 | 36/68 | 16/33 | 20/35 | - | - | - | - | - |
| 1E3Z | 0.95 | 0.46 | 0.57 | 0.97 | 0.49 | 0.51 | 12 | 397 | 14 | 9 | 46/75 | 14/28 | 16/26 | 16/21 | - | - | - | - |
| 1MFU | 0.92 | 0.56 | 0.51 | 0.96 | 0.49 | 0.54 | 19 | 381 | 15 | 18 | 86/155 | 22/24 | 0/21 | 19/30 | 15/22 | 21/28 | 9/30 | - |
| 1V7X | 0.97 | 0.33 | 0.77 | 0.97 | 0.49 | 0.47 | 10 | 653 | 20 | 3 | 34/50 | 34/50 | - | - | - | - | - | - |
| 2AXR | 0.95 | 0.25 | 1.00 | 0.95 | 0.49 | 0.40 | 7 | 406 | 21 | 0 | 32/32 | 32/32 | - | - | - | - | - | - |
| 2GJP | 0.91 | 0.50 | 0.58 | 0.95 | 0.49 | 0.54 | 21 | 359 | 21 | 15 | 74/150 | 9/28 | 11/30 | 23/40 | 9/20 | 11/11 | 11/21 | - |
| 2YHX | 0.98 | 0.75 | 0.33 | 1.00 | 0.49 | 0.46 | 3 | 347 | 1 | 6 | 3/20 | 3/20 | - | - | - | - | - | - |
| 3BMW | 0.95 | 0.47 | 0.57 | 0.97 | 0.49 | 0.52 | 17 | 553 | 19 | 13 | 62/112 | 0/30 | 52/65 | 10/17 | - | - | - | - |
| 1G93 | 0.93 | 0.32 | 0.80 | 0.93 | 0.48 | 0.46 | 8 | 231 | 17 | 2 | 28/38 | 28/38 | - | - | - | - | - | - |
| 1OT2 | 0.92 | 0.72 | 0.37 | 0.98 | 0.48 | 0.49 | 23 | 528 | 9 | 39 | 88/231 | 7/51 | 47/60 | 0/29 | 27/32 | 7/36 | 0/23 | - |
| 1TJ4 | 0.92 | 0.50 | 0.53 | 0.96 | 0.48 | 0.52 | 8 | 174 | 8 | 7 | 15/48 | 15/48 | - | - | - | - | - | - |
| 1U7G | 0.97 | 0.50 | 0.50 | 0.98 | 0.48 | 0.50 | 5 | 310 | 5 | 5 | 12/44 | 12/44 | - | - | - | - | - | - |
| 2D3N | 0.89 | 0.64 | 0.46 | 0.96 | 0.48 | 0.53 | 28 | 354 | 16 | 33 | 96/248 | 15/71 | 34/53 | 7/19 | 12/22 | 16/31 | 8/42 | 4/10 |
| 2O7I | 0.93 | 0.30 | 0.87 | 0.94 | 0.48 | 0.44 | 13 | 451 | 31 | 2 | 55/64 | 55/64 | - | - | - | - | - | - |
| 2QZ2 | 0.87 | 0.57 | 0.54 | 0.93 | 0.48 | 0.55 | 13 | 132 | 10 | 11 | 35/87 | 35/44 | 0/43 | - | - | - | - | - |
| 3CKZ | 0.93 | 0.44 | 0.60 | 0.96 | 0.48 | 0.51 | 12 | 315 | 15 | 8 | 32/51 | 32/51 | - | - | - | - | - | - |
| 3II1 | 0.95 | 0.24 | 1.00 | 0.95 | 0.48 | 0.39 | 8 | 447 | 25 | 0 | 30/31 | 30/31 | - | - | - | - | - | - |
| 1OGO | 0.96 | 0.35 | 0.67 | 0.97 | 0.47 | 0.46 | 8 | 497 | 15 | 4 | 27/40 | 27/40 | - | - | - | - | - | - |
| 1UXX | 0.90 | 1.00 | 0.25 | 1.00 | 0.47 | 0.40 | 4 | 100 | 0 | 12 | 24/70 | 4/24 | 20/46 | - | - | - | - | - |
| 2FST | 0.95 | 0.75 | 0.32 | 0.99 | 0.47 | 0.44 | 6 | 285 | 2 | 13 | 25/80 | 19/45 | 6/13 | 0/10 | 0/12 | - | - | - |
| 2IHO | 0.92 | 0.46 | 0.57 | 0.94 | 0.47 | 0.51 | 12 | 236 | 14 | 9 | 47/96 | 21/54 | 26/42 | - | - | - | - | - |
| 2QMJ | 0.96 | 0.30 | 0.79 | 0.97 | 0.47 | 0.43 | 11 | 747 | 26 | 3 | 28/37 | 28/37 | - | - | - | - | - | - |
| 3ABX | 0.91 | 0.41 | 0.65 | 0.93 | 0.47 | 0.50 | 15 | 293 | 22 | 8 | 54/89 | 37/51 | 17/38 | - | - | - | - | - |
| 1JIL | 0.97 | 0.67 | 0.33 | 0.99 | 0.46 | 0.44 | 4 | 288 | 2 | 8 | 8/38 | 8/38 | - | - | - | - | - | - |
| 1MXD | 0.88 | 0.59 | 0.48 | 0.95 | 0.46 | 0.53 | 24 | 299 | 17 | 26 | 105/212 | 13/45 | 5/57 | 38/48 | 19/26 | 30/36 | - | - |
| 1R6D | 0.93 | 0.35 | 0.69 | 0.94 | 0.46 | 0.46 | 9 | 262 | 17 | 4 | 15/24 | 15/24 | - | - | - | - | - | - |
| 1TYW | 0.95 | 0.44 | 0.52 | 0.97 | 0.46 | 0.48 | 11 | 477 | 14 | 10 | 32/72 | 32/61 | 0/11 | - | - | - | - | - |
| 1X9D | 0.94 | 0.44 | 0.56 | 0.96 | 0.46 | 0.49 | 10 | 330 | 13 | 8 | 24/52 | 24/52 | - | - | - | - | - | - |
| 1I24 | 0.95 | 0.40 | 0.57 | 0.96 | 0.45 | 0.47 | 8 | 307 | 12 | 6 | 21/37 | 21/37 | - | - | - | - | - | - |
| 2HS3 | 0.95 | 0.36 | 0.63 | 0.97 | 0.45 | 0.46 | 10 | 493 | 18 | 6 | 18/33 | 18/33 | - | - | - | - | - | - |
| 1FOA | 0.90 | 0.31 | 0.75 | 0.91 | 0.44 | 0.44 | 12 | 263 | 27 | 4 | 37/56 | 26/37 | 11/19 | - | - | - | - | - |
| 1SB8 | 0.94 | 0.50 | 0.44 | 0.97 | 0.44 | 0.47 | 8 | 281 | 8 | 10 | 26/66 | 26/66 | - | - | - | - | - | - |
| 2D7R | 0.92 | 0.33 | 0.68 | 0.94 | 0.44 | 0.44 | 15 | 445 | 31 | 7 | 48/71 | 8/25 | 40/46 | - | - | - | - | - |
| 2ZJ3 | 0.96 | 0.67 | 0.31 | 0.99 | 0.44 | 0.42 | 4 | 292 | 2 | 9 | 9/36 | 9/36 | - | - | - | - | - | - |
| 1HV6 | 0.94 | 0.35 | 0.58 | 0.96 | 0.42 | 0.44 | 7 | 287 | 13 | 5 | 25/43 | 22/27 | 3/16 | - | - | - | - | - |
| 2F6D | 0.95 | 0.48 | 0.42 | 0.97 | 0.42 | 0.44 | 10 | 417 | 11 | 14 | 36/97 | 36/54 | 0/43 | - | - | - | - | - |
| 3HKN | 0.92 | 0.50 | 0.42 | 0.96 | 0.42 | 0.46 | 8 | 207 | 8 | 11 | 17/49 | 17/49 | - | - | - | - | - | - |
| 3MAN | 0.92 | 0.32 | 0.64 | 0.94 | 0.42 | 0.42 | 7 | 222 | 15 | 4 | 27/45 | 27/45 | - | - | - | - | - | - |
| 1GYM | 0.97 | 0.30 | 0.60 | 0.97 | 0.41 | 0.40 | 3 | 249 | 7 | 2 | 17/23 | 17/23 | - | - | - | - | - | - |
| 1QKQ | 0.91 | 0.31 | 0.67 | 0.92 | 0.41 | 0.42 | 4 | 110 | 9 | 2 | 18/29 | 18/29 | - | - | - | - | - | - |
| 1QNR | 0.95 | 0.26 | 0.71 | 0.95 | 0.41 | 0.39 | 5 | 283 | 14 | 2 | 23/33 | 23/33 | - | - | - | - | - | - |
| 1LXM | 0.96 | 0.35 | 0.50 | 0.97 | 0.40 | 0.41 | 10 | 715 | 19 | 10 | 36/76 | 12/13 | 24/47 | 0/16 | - | - | - | - |
| 1GJW | 0.95 | 0.41 | 0.42 | 0.97 | 0.39 | 0.42 | 11 | 548 | 16 | 15 | 35/87 | 20/67 | 15/20 | - | - | - | - | - |
| 3EWR | 0.93 | 0.75 | 0.23 | 0.99 | 0.39 | 0.35 | 3 | 132 | 1 | 10 | 7/42 | 7/42 | - | - | - | - | - | - |
| 1DLJ | 0.96 | 0.29 | 0.56 | 0.97 | 0.38 | 0.39 | 5 | 344 | 12 | 4 | 9/30 | 9/30 | - | - | - | - | - | - |
| 2HRL | 0.94 | 0.67 | 0.25 | 0.99 | 0.38 | 0.36 | 2 | 100 | 1 | 6 | 9/40 | 9/40 | - | - | - | - | - | - |
| 2Z4T | 0.95 | 0.21 | 0.75 | 0.95 | 0.38 | 0.33 | 6 | 436 | 22 | 2 | 26/33 | 26/33 | - | - | - | - | - | - |
| 2EWE | 0.93 | 0.47 | 0.35 | 0.97 | 0.37 | 0.40 | 7 | 279 | 8 | 13 | 18/50 | 18/50 | - | - | - | - | - | - |
| 2UAG | 0.95 | 0.33 | 0.46 | 0.97 | 0.37 | 0.39 | 6 | 355 | 12 | 7 | 20/46 | 20/46 | - | - | - | - | - | - |
| 2W62 | 0.92 | 0.78 | 0.19 | 1.00 | 0.37 | 0.31 | 7 | 365 | 2 | 29 | 20/124 | 11/58 | 9/38 | 0/12 | 0/16 | - | - | - |
| 1FA9 | 0.96 | 0.20 | 0.70 | 0.96 | 0.36 | 0.31 | 7 | 744 | 28 | 3 | 24/29 | 24/29 | - | - | - | - | - | - |
| 1MW0 | 0.89 | 0.50 | 0.34 | 0.96 | 0.36 | 0.40 | 21 | 493 | 21 | 41 | 78/230 | 11/60 | 67/104 | 0/25 | 0/12 | 0/29 | - | - |
| 1FWV | 0.95 | 0.50 | 0.29 | 0.98 | 0.35 | 0.36 | 2 | 119 | 2 | 5 | 14/31 | 14/31 | - | - | - | - | - | - |
| 1PYY | 0.98 | 0.36 | 0.36 | 0.99 | 0.35 | 0.36 | 4 | 537 | 7 | 7 | 14/45 | 1/23 | 13/22 | - | - | - | - | - |
| 2BHZ | 0.89 | 0.53 | 0.32 | 0.96 | 0.35 | 0.40 | 20 | 470 | 18 | 43 | 76/265 | 0/55 | 33/42 | 43/75 | 0/32 | 0/15 | 0/15 | 0/31 |
| 1W3G | 0.95 | 0.75 | 0.17 | 1.00 | 0.34 | 0.27 | 3 | 282 | 1 | 15 | 12/73 | 12/42 | 0/31 | - | - | - | - | - |
| 2F5V | 0.92 | 0.18 | 0.75 | 0.92 | 0.34 | 0.29 | 9 | 471 | 41 | 3 | 19/31 | 19/31 | - | - | - | - | - | - |
| 2FUE | 0.94 | 0.31 | 0.44 | 0.96 | 0.34 | 0.36 | 4 | 205 | 9 | 5 | 7/32 | 7/32 | - | - | - | - | - | - |
| 2YVP | 0.93 | 0.50 | 0.25 | 0.98 | 0.32 | 0.33 | 3 | 156 | 3 | 9 | 6/42 | 6/42 | - | - | - | - | - | - |
| 3CX4 | 0.88 | 0.44 | 0.33 | 0.95 | 0.32 | 0.38 | 15 | 353 | 19 | 31 | 53/191 | 0/38 | 0/50 | 15/63 | 38/40 | - | - | - |
| 1FA2 | 0.93 | 0.23 | 0.50 | 0.95 | 0.31 | 0.31 | 7 | 420 | 24 | 7 | 18/38 | 18/38 | - | - | - | - | - | - |
| 1YON | 0.96 | 0.67 | 0.15 | 1.00 | 0.31 | 0.25 | 2 | 253 | 1 | 11 | 4/43 | 2/14 | 2/29 | - | - | - | - | - |
| 2PC8 | 0.92 | 0.28 | 0.44 | 0.95 | 0.31 | 0.34 | 7 | 322 | 18 | 9 | 24/57 | 24/25 | 0/32 | - | - | - | - | - |
| 1OA7 | 0.92 | 0.29 | 0.40 | 0.94 | 0.29 | 0.33 | 4 | 168 | 10 | 6 | 9/28 | 9/28 | - | - | - | - | - | - |
| 1TXZ | 0.94 | 0.67 | 0.14 | 1.00 | 0.29 | 0.24 | 2 | 212 | 1 | 12 | 6/46 | 6/46 | - | - | - | - | - | - |
| 2WQQ | 0.91 | 0.46 | 0.25 | 0.97 | 0.29 | 0.32 | 5 | 200 | 6 | 15 | 18/61 | 0/19 | 18/42 | - | - | - | - | - |
| 1GXO | 0.95 | 0.30 | 0.30 | 0.98 | 0.28 | 0.30 | 3 | 268 | 7 | 7 | 10/38 | 10/24 | 0/14 | - | - | - | - | - |
| 1JZS | 0.97 | 0.33 | 0.25 | 0.99 | 0.28 | 0.29 | 4 | 750 | 8 | 12 | 11/42 | 11/42 | - | - | - | - | - | - |
| 2GEJ | 0.94 | 0.25 | 0.36 | 0.96 | 0.27 | 0.30 | 4 | 292 | 12 | 7 | 7/37 | 7/37 | - | - | - | - | - | - |
| 3FWL | 0.98 | 0.50 | 0.14 | 1.00 | 0.26 | 0.22 | 2 | 644 | 2 | 12 | 11/60 | 11/60 | - | - | - | - | - | - |
| 3H1Y | 0.94 | 0.22 | 0.36 | 0.96 | 0.26 | 0.28 | 4 | 328 | 14 | 7 | 12/44 | 12/44 | - | - | - | - | - | - |
| 1FFY | 0.97 | 0.22 | 0.33 | 0.98 | 0.25 | 0.26 | 5 | 842 | 18 | 10 | 15/50 | 15/50 | - | - | - | - | - | - |
| 1PIG | 0.89 | 0.36 | 0.27 | 0.95 | 0.25 | 0.31 | 11 | 389 | 20 | 30 | 52/148 | 0/32 | 19/36 | 22/28 | 11/33 | 0/19 | - | - |
| 2UUI | 0.95 | 0.50 | 0.14 | 0.99 | 0.25 | 0.22 | 1 | 135 | 1 | 6 | 3/23 | 3/23 | - | - | - | - | - | - |
| 3F3E | 0.94 | 0.39 | 0.20 | 0.98 | 0.25 | 0.26 | 5 | 404 | 8 | 20 | 9/81 | 0/36 | 9/45 | - | - | - | - | - |
| 3IID | 0.92 | 0.50 | 0.15 | 0.99 | 0.25 | 0.24 | 2 | 143 | 2 | 11 | 7/41 | 7/41 | - | - | - | - | - | - |
| 1OD3 | 0.88 | 0.38 | 0.25 | 0.95 | 0.24 | 0.30 | 3 | 99 | 5 | 9 | 16/51 | 16/51 | - | - | - | - | - | - |
| 1RP8 | 0.87 | 0.40 | 0.23 | 0.96 | 0.24 | 0.29 | 10 | 316 | 15 | 33 | 34/178 | 0/54 | 0/36 | 34/66 | 0/22 | - | - | - |
| 3HN1 | 0.95 | 0.29 | 0.25 | 0.98 | 0.24 | 0.27 | 4 | 390 | 10 | 12 | 15/63 | 15/52 | 0/11 | - | - | - | - | - |
| 1L8T | 0.93 | 0.27 | 0.25 | 0.97 | 0.23 | 0.26 | 3 | 231 | 8 | 9 | 11/60 | 11/60 | - | - | - | - | - | - |
| 1SLY | 0.96 | 0.21 | 0.31 | 0.97 | 0.23 | 0.25 | 4 | 529 | 15 | 9 | 13/41 | 13/41 | - | - | - | - | - | - |
| 2GR2 | 0.94 | 0.13 | 0.50 | 0.94 | 0.23 | 0.20 | 3 | 345 | 21 | 3 | 12/27 | 12/27 | - | - | - | - | - | - |
| 2IC8 | 0.87 | 0.60 | 0.13 | 0.99 | 0.23 | 0.21 | 3 | 138 | 2 | 20 | 7/94 | 0/43 | 0/15 | 0/11 | 7/25 | - | - | - |
| 1AUA | 0.94 | 0.25 | 0.25 | 0.97 | 0.22 | 0.25 | 3 | 265 | 9 | 9 | 6/55 | 6/31 | 0/24 | - | - | - | - | - |
| 1ILD | 0.86 | 0.23 | 0.37 | 0.90 | 0.22 | 0.29 | 7 | 209 | 23 | 12 | 21/91 | 0/25 | 15/21 | 4/21 | 0/12 | 2/12 | - | - |
| 1J39 | 0.96 | 0.29 | 0.20 | 0.98 | 0.22 | 0.24 | 2 | 303 | 5 | 8 | 7/31 | 7/31 | - | - | - | - | - | - |
| 1M2J | 0.95 | 0.18 | 0.33 | 0.96 | 0.22 | 0.24 | 2 | 222 | 9 | 4 | 11/33 | 11/33 | - | - | - | - | - | - |
| 1QZ6 | 0.96 | 0.20 | 0.29 | 0.98 | 0.22 | 0.24 | 2 | 309 | 8 | 5 | 8/24 | 8/24 | - | - | - | - | - | - |
| 1TL2 | 0.73 | 0.73 | 0.13 | 0.98 | 0.22 | 0.21 | 8 | 153 | 3 | 56 | 24/213 | 5/43 | 0/31 | 0/44 | 9/40 | 0/10 | 10/45 | - |
| 1ZX5 | 0.92 | 0.15 | 0.43 | 0.94 | 0.22 | 0.22 | 3 | 250 | 17 | 4 | 7/32 | 7/32 | - | - | - | - | - | - |
| 3BIF | 0.95 | 0.25 | 0.23 | 0.98 | 0.22 | 0.24 | 3 | 369 | 9 | 10 | 6/45 | 6/45 | - | - | - | - | - | - |
| 3GD9 | 0.95 | 0.40 | 0.12 | 0.99 | 0.20 | 0.18 | 2 | 322 | 3 | 15 | 4/55 | 4/55 | - | - | - | - | - | - |
| 1OGQ | 0.94 | 0.33 | 0.12 | 0.99 | 0.17 | 0.17 | 2 | 271 | 4 | 15 | 8/60 | 8/22 | 0/24 | 0/14 | - | - | - | - |
| 3D1R | 0.94 | 0.25 | 0.15 | 0.98 | 0.17 | 0.19 | 2 | 267 | 6 | 11 | 7/37 | 7/37 | - | - | - | - | - | - |
| 1Q0Z | 0.92 | 0.17 | 0.25 | 0.94 | 0.16 | 0.20 | 3 | 255 | 15 | 9 | 6/36 | 6/36 | - | - | - | - | - | - |
| 2JH7 | 0.96 | 0.20 | 0.17 | 0.98 | 0.16 | 0.18 | 1 | 188 | 4 | 5 | 3/27 | 3/27 | - | - | - | - | - | - |
| 1L1R | 0.94 | 0.25 | 0.13 | 0.98 | 0.15 | 0.17 | 1 | 152 | 3 | 7 | 6/32 | 6/32 | - | - | - | - | - | - |
| 1OFL | 0.89 | 0.27 | 0.16 | 0.96 | 0.15 | 0.20 | 6 | 369 | 16 | 32 | 12/118 | 12/83 | 0/35 | - | - | - | - | - |
| 1T10 | 0.98 | 0.25 | 0.10 | 0.99 | 0.15 | 0.14 | 1 | 495 | 3 | 9 | 1/25 | 1/25 | - | - | - | - | - | - |
| 3KH6 | 0.93 | 0.33 | 0.09 | 0.99 | 0.15 | 0.14 | 1 | 154 | 2 | 10 | 3/34 | 3/34 | - | - | - | - | - | - |
| 5ADH | 0.94 | 0.18 | 0.18 | 0.97 | 0.15 | 0.18 | 2 | 301 | 9 | 9 | 6/33 | 6/33 | - | - | - | - | - | - |
| 1YW1 | 0.97 | 0.17 | 0.14 | 0.99 | 0.14 | 0.15 | 1 | 346 | 5 | 6 | 4/26 | 4/26 | - | - | - | - | - | - |
| 2J8F | 0.88 | 0.19 | 0.24 | 0.92 | 0.14 | 0.21 | 5 | 264 | 22 | 16 | 15/81 | 15/81 | - | - | - | - | - | - |
| 1BG9 | 0.91 | 0.10 | 0.30 | 0.92 | 0.13 | 0.15 | 3 | 334 | 28 | 7 | 13/56 | 0/33 | 13/23 | - | - | - | - | - |
| 4GPB | 0.97 | 0.20 | 0.11 | 0.99 | 0.13 | 0.14 | 2 | 745 | 8 | 17 | 4/59 | 4/20 | 0/39 | - | - | - | - | - |
| 1UZ0 | 0.82 | 0.21 | 0.23 | 0.90 | 0.12 | 0.22 | 3 | 95 | 11 | 10 | 8/41 | 8/41 | - | - | - | - | - | - |
| 2GUP | 0.91 | 0.25 | 0.10 | 0.98 | 0.12 | 0.14 | 2 | 236 | 6 | 18 | 2/73 | 0/31 | 2/42 | - | - | - | - | - |
| 2P3K | 0.92 | 0.20 | 0.13 | 0.97 | 0.12 | 0.15 | 1 | 133 | 4 | 7 | 1/33 | 1/33 | - | - | - | - | - | - |
| 1LRJ | 0.90 | 0.16 | 0.17 | 0.94 | 0.11 | 0.16 | 3 | 268 | 16 | 15 | 5/57 | 0/36 | 5/21 | - | - | - | - | - |
| 3BKV | 0.89 | 0.22 | 0.09 | 0.97 | 0.09 | 0.13 | 2 | 224 | 7 | 20 | 4/68 | 4/68 | - | - | - | - | - | - |
| 3CL5 | 0.95 | 0.09 | 0.14 | 0.97 | 0.09 | 0.11 | 1 | 310 | 10 | 6 | 2/29 | 2/29 | - | - | - | - | - | - |
| 1GYE | 0.90 | 0.09 | 0.20 | 0.92 | 0.08 | 0.12 | 2 | 252 | 21 | 8 | 5/26 | 5/26 | - | - | - | - | - | - |
| 2VWG | 0.94 | 0.11 | 0.08 | 0.97 | 0.07 | 0.10 | 1 | 298 | 8 | 11 | 2/33 | 2/33 | - | - | - | - | - | - |
| 2VU9 | 0.91 | 0.07 | 0.15 | 0.93 | 0.06 | 0.10 | 2 | 355 | 25 | 11 | 12/57 | 12/57 | - | - | - | - | - | - |
| 2WWU | 0.93 | 0.11 | 0.06 | 0.97 | 0.05 | 0.08 | 1 | 300 | 8 | 16 | 1/56 | 1/35 | 0/21 | - | - | - | - | - |
| 2O0V | 0.91 | 0.07 | 0.05 | 0.96 | 0.01 | 0.06 | 1 | 320 | 13 | 19 | 3/58 | 0/37 | 3/21 | - | - | - | - | - |
| 148L | 0.00 | 0.00 | 0.00 | 0.00 | 0.00 | 0.00 | 0 | 136 | 0 | 18 | 0 | 0 | - | - | - | - | - | - |
| 154L | 0.00 | 0.00 | 0.00 | 0.00 | 0.00 | 0.00 | 0 | 140 | 0 | 16 | 0 | 0 | - | - | - | - | - | - |
| 1DRK | 0.00 | 0.00 | 0.00 | 0.00 | 0.00 | 0.00 | 0 | 214 | 0 | 11 | 0 | 0 | - | - | - | - | - | - |
| 1ECY | 0.00 | 0.00 | 0.00 | 0.00 | 0.00 | 0.00 | 0 | 110 | 0 | 28 | 0 | 0 | - | - | - | - | - | - |
| 1EN2 | 0.00 | 0.00 | 0.00 | 0.00 | 0.00 | 0.00 | 0 | 76 | 0 | 6 | 0 | 0 | - | - | - | - | - | - |
| 1EXA | 0.00 | 0.00 | 0.00 | 0.00 | 0.00 | 0.00 | 0 | 214 | 0 | 4 | 0 | 0 | - | - | - | - | - | - |
| 1FX8 | 0.00 | 0.00 | 0.00 | 0.00 | 0.00 | 0.00 | 0 | 210 | 0 | 18 | 0 | 0 | - | - | - | - | - | - |
| 1IZ2 | 0.00 | 0.00 | 0.00 | 0.00 | 0.00 | 0.00 | 0 | 336 | 0 | 7 | 0 | 0 | - | - | - | - | - | - |
| 1J2Z | 0.00 | 0.00 | 0.00 | 0.00 | 0.00 | 0.00 | 0 | 227 | 0 | 11 | 0 | 0 | - | - | - | - | - | - |
| 1J4N | 0.00 | 0.00 | 0.00 | 0.00 | 0.00 | 0.00 | 0 | 219 | 0 | 8 | 0 | 0 | - | - | - | - | - | - |
| 1JPC | 0.00 | 0.00 | 0.00 | 0.00 | 0.00 | 0.00 | 0 | 74 | 0 | 26 | 0 | 0 | - | - | - | - | - | - |
| 1KZN | 0.00 | 0.00 | 0.00 | 0.00 | 0.00 | 0.00 | 0 | 162 | 0 | 13 | 0 | 0 | - | - | - | - | - | - |
| 1NF9 | 0.00 | 0.00 | 0.00 | 0.00 | 0.00 | 0.00 | 0 | 175 | 0 | 5 | 0 | 0 | - | - | - | - | - | - |
| 1NPL | 0.00 | 0.00 | 0.00 | 0.00 | 0.00 | 0.00 | 0 | 78 | 0 | 25 | 0 | 0 | - | - | - | - | - | - |
| 1OFC | 0.00 | 0.00 | 0.00 | 0.00 | 0.00 | 0.00 | 0 | 223 | 0 | 8 | 0 | 0 | - | - | - | - | - | - |
| 1OPR | 0.00 | 0.00 | 0.00 | 0.00 | 0.00 | 0.00 | 0 | 186 | 0 | 7 | 0 | 0 | - | - | - | - | - | - |
| 1QGI | 0.00 | 0.00 | 0.00 | 0.00 | 0.00 | 0.00 | 0 | 223 | 0 | 10 | 0 | 0 | - | - | - | - | - | - |
| 1TJY | 0.00 | 0.00 | 0.00 | 0.00 | 0.00 | 0.00 | 0 | 244 | 0 | 13 | 0 | 0 | - | - | - | - | - | - |
| 1YC9 | 0.00 | 0.00 | 0.00 | 0.00 | 0.00 | 0.00 | 0 | 392 | 0 | 9 | 0 | 0 | - | - | - | - | - | - |
| 1YLJ | 0.00 | 0.00 | 0.00 | 0.00 | 0.00 | 0.00 | 0 | 223 | 0 | 10 | 0 | 0 | - | - | - | - | - | - |
| 1YMG | 0.00 | 0.00 | 0.00 | 0.00 | 0.00 | 0.00 | 0 | 202 | 0 | 7 | 0 | 0 | - | - | - | - | - | - |
| 2BBH | 0.00 | 0.00 | 0.00 | 0.00 | 0.00 | 0.00 | 0 | 194 | 0 | 21 | 0 | 0 | - | - | - | - | - | - |
| 2C56 | 0.00 | 0.00 | 0.00 | 0.00 | 0.00 | 0.00 | 0 | 196 | 0 | 9 | 0 | 0 | - | - | - | - | - | - |
| 2CCV | 0.00 | 0.00 | 0.00 | 0.00 | 0.00 | 0.00 | 0 | 87 | 0 | 7 | 0 | 0 | - | - | - | - | - | - |
| 2JAF | 0.00 | 0.00 | 0.00 | 0.00 | 0.00 | 0.00 | 0 | 221 | 0 | 7 | 0 | 0 | - | - | - | - | - | - |
| 2JCR | 0.00 | 0.00 | 0.00 | 0.00 | 0.00 | 0.00 | 0 | 121 | 0 | 15 | 0 | 0 | - | - | - | - | - | - |
| 2JG0 | 0.00 | 0.00 | 0.00 | 0.00 | 0.00 | 0.00 | 0 | 432 | 0 | 16 | 0 | 0 | - | - | - | - | - | - |
| 2JHL | 0.00 | 0.00 | 0.00 | 0.00 | 0.00 | 0.00 | 0 | 184 | 0 | 8 | 0 | 0 | - | - | - | - | - | - |
| 2O9G | 0.00 | 0.00 | 0.00 | 0.00 | 0.00 | 0.00 | 0 | 202 | 0 | 7 | 0 | 0 | - | - | - | - | - | - |
| 2RDG | 0.00 | 0.00 | 0.00 | 0.00 | 0.00 | 0.00 | 0 | 173 | 0 | 11 | 0 | 0 | - | - | - | - | - | - |
| 2V5T | 0.00 | 0.00 | 0.00 | 0.00 | 0.00 | 0.00 | 0 | 166 | 0 | 7 | 0 | 0 | - | - | - | - | - | - |
| 2VXK | 0.00 | 0.00 | 0.00 | 0.00 | 0.00 | 0.00 | 0 | 146 | 0 | 10 | 0 | 0 | - | - | - | - | - | - |
| 2W1P | 0.00 | 0.00 | 0.00 | 0.00 | 0.00 | 0.00 | 0 | 222 | 0 | 13 | 0 | 0 | - | - | - | - | - | - |
| 2YVV | 0.00 | 0.00 | 0.00 | 0.00 | 0.00 | 0.00 | 0 | 319 | 0 | 11 | 0 | 0 | - | - | - | - | - | - |
| 2Z8L | 0.00 | 0.00 | 0.00 | 0.00 | 0.00 | 0.00 | 0 | 173 | 0 | 11 | 0 | 0 | - | - | - | - | - | - |
| 2ZG3 | 0.00 | 0.00 | 0.00 | 0.00 | 0.00 | 0.00 | 0 | 193 | 0 | 8 | 0 | 0 | - | - | - | - | - | - |
| 3C9E | 0.00 | 0.00 | 0.00 | 0.00 | 0.00 | 0.00 | 0 | 177 | 0 | 12 | 0 | 0 | - | - | - | - | - | - |
| 3CT5 | 0.00 | 0.00 | 0.00 | 0.00 | 0.00 | 0.00 | 0 | 111 | 0 | 14 | 0 | 0 | - | - | - | - | - | - |
| 3H2K | 0.00 | 0.00 | 0.00 | 0.00 | 0.00 | 0.00 | 0 | 333 | 0 | 18 | 0 | 0 | - | - | - | - | - | - |
| 966C | 0.00 | 0.00 | 0.00 | 0.00 | 0.00 | 0.00 | 0 | 137 | 0 | 12 | 0 | 0 | - | - | - | - | - | - |
| 9ABP | 0.00 | 0.00 | 0.00 | 0.00 | 0.00 | 0.00 | 0 | 246 | 0 | 16 | 0 | 0 | - | - | - | - | - | - |
| 1F31 | 0.97 | 0.00 | 0.00 | 0.98 | -0.01 | 0.00 | 0 | 1114 | 28 | 9 | 0/42 | 0/42 | - | - | - | - | - | - |
| 1N0U | 0.98 | 0.00 | 0.00 | 0.98 | -0.01 | 0.00 | 0 | 739 | 12 | 7 | 0/23 | 0/23 | - | - | - | - | - | - |
| 1OB2 | 0.93 | 0.00 | 0.00 | 1.00 | -0.01 | 0.00 | 0 | 343 | 1 | 24 | 0/84 | 0/42 | 0/42 | - | - | - | - | - |
| 1PW5 | 0.97 | 0.00 | 0.00 | 1.00 | -0.01 | 0.00 | 0 | 227 | 1 | 6 | 0/30 | 0/30 | - | - | - | - | - | - |
| 1TAQ | 0.97 | 0.00 | 0.00 | 0.98 | -0.01 | 0.00 | 0 | 724 | 16 | 5 | 0/23 | 0/23 | - | - | - | - | - | - |
| 2A0Z | 0.98 | 0.00 | 0.00 | 1.00 | -0.01 | 0.00 | 0 | 607 | 2 | 11 | 0/40 | 0/20 | 0/20 | - | - | - | - | - |
| 2BDM | 0.93 | 0.00 | 0.00 | 1.00 | -0.01 | 0.00 | 0 | 410 | 1 | 32 | 1/117 | 1/68 | 0/21 | 0/28 | - | - | - | - |
| 2C9E | 0.97 | 0.00 | 0.00 | 1.00 | -0.01 | 0.00 | 0 | 305 | 1 | 10 | 0/38 | 0/15 | 0/23 | - | - | - | - | - |
| 2EVU | 0.97 | 0.00 | 0.00 | 1.00 | -0.01 | 0.00 | 0 | 212 | 1 | 6 | 0/30 | 0/17 | 0/13 | - | - | - | - | - |
| 2FN8 | 0.95 | 0.00 | 0.00 | 1.00 | -0.01 | 0.00 | 0 | 230 | 1 | 11 | 0/34 | 0/34 | - | - | - | - | - | - |
| 2VF5 | 0.96 | 0.00 | 0.00 | 1.00 | -0.01 | 0.00 | 0 | 313 | 1 | 12 | 0/37 | 0/37 | - | - | - | - | - | - |
| 2ZZL | 0.96 | 0.00 | 0.00 | 1.00 | -0.01 | 0.00 | 0 | 208 | 1 | 8 | 0/29 | 0/29 | - | - | - | - | - | - |
| 1A5Z | 0.96 | 0.00 | 0.00 | 0.99 | -0.02 | 0.00 | 0 | 266 | 2 | 10 | 0/42 | 0/22 | 0/20 | - | - | - | - | - |
| 1HFU | 0.96 | 0.00 | 0.00 | 0.98 | -0.02 | 0.00 | 0 | 432 | 11 | 9 | 0/44 | 0/30 | 0/14 | - | - | - | - | - |
| 1UMI | 0.95 | 0.00 | 0.00 | 0.99 | -0.02 | 0.00 | 0 | 157 | 2 | 6 | 0/35 | 0/35 | - | - | - | - | - | - |
| 1W6K | 0.95 | 0.00 | 0.00 | 0.97 | -0.02 | 0.00 | 0 | 603 | 19 | 10 | 0/49 | 0/25 | 0/24 | - | - | - | - | - |
| 2C78 | 0.96 | 0.00 | 0.00 | 0.98 | -0.02 | 0.00 | 0 | 346 | 6 | 10 | 0/33 | 0/33 | - | - | - | - | - | - |
| 2CNE | 0.96 | 0.00 | 0.00 | 0.98 | -0.02 | 0.00 | 0 | 249 | 5 | 6 | 0/32 | 0/32 | - | - | - | - | - | - |
| 2EBH | 0.96 | 0.00 | 0.00 | 0.98 | -0.02 | 0.00 | 0 | 627 | 12 | 13 | 0/66 | 0/49 | 0/17 | - | - | - | - | - |
| 2F5T | 0.92 | 0.00 | 0.00 | 1.00 | -0.02 | 0.00 | 0 | 191 | 1 | 16 | 0/60 | 0/60 | - | - | - | - | - | - |
| 2HHQ | 0.94 | 0.00 | 0.00 | 1.00 | -0.02 | 0.00 | 0 | 491 | 2 | 28 | 0/105 | 0/47 | 0/23 | 0/35 | - | - | - | - |
| 2OBT | 0.97 | 0.00 | 0.00 | 0.99 | -0.02 | 0.00 | 0 | 278 | 4 | 6 | 0/21 | 0/21 | - | - | - | - | - | - |
| 2R68 | 0.95 | 0.00 | 0.00 | 0.98 | -0.02 | 0.00 | 0 | 382 | 7 | 12 | 0/42 | 0/42 | - | - | - | - | - | - |
| 2VK2 | 0.94 | 0.00 | 0.00 | 0.99 | -0.02 | 0.00 | 0 | 232 | 2 | 14 | 0/41 | 0/41 | - | - | - | - | - | - |
| 3B9Z | 0.96 | 0.00 | 0.00 | 0.98 | -0.02 | 0.00 | 0 | 320 | 7 | 7 | 0/23 | 0/12 | 0/11 | - | - | - | - | - |
| 3CR9 | 0.96 | 0.00 | 0.00 | 0.97 | -0.02 | 0.00 | 0 | 610 | 16 | 10 | 0/36 | 0/36 | - | - | - | - | - | - |
| 3FHH | 0.95 | 0.00 | 0.00 | 0.96 | -0.02 | 0.00 | 0 | 554 | 25 | 6 | 0/25 | 0/25 | - | - | - | - | - | - |
| 3GZ9 | 0.96 | 0.00 | 0.00 | 0.98 | -0.02 | 0.00 | 0 | 232 | 4 | 5 | 0/23 | 0/23 | - | - | - | - | - | - |
| 3HD6 | 0.97 | 0.00 | 0.00 | 0.99 | -0.02 | 0.00 | 0 | 333 | 5 | 7 | 0/30 | 0/30 | - | - | - | - | - | - |
| 3ICV | 0.96 | 0.00 | 0.00 | 0.99 | -0.02 | 0.00 | 0 | 245 | 3 | 7 | 0/26 | 0/13 | 0/13 | - | - | - | - | - |
| 3IFE | 0.96 | 0.00 | 0.00 | 0.99 | -0.02 | 0.00 | 0 | 366 | 4 | 12 | 0/40 | 0/19 | 0/21 | - | - | - | - | - |
| 3IIU | 0.95 | 0.00 | 0.00 | 0.98 | -0.02 | 0.00 | 0 | 142 | 3 | 4 | 0/19 | 0/19 | - | - | - | - | - | - |
| 1C4O | 0.94 | 0.00 | 0.00 | 0.95 | -0.03 | 0.00 | 0 | 422 | 21 | 8 | 0/16 | 0/16 | - | - | - | - | - | - |
| 1CPY | 0.94 | 0.00 | 0.00 | 0.96 | -0.03 | 0.00 | 0 | 332 | 14 | 9 | 0/29 | 0/13 | 0/16 | - | - | - | - | - |
| 1FE2 | 0.94 | 0.00 | 0.00 | 0.97 | -0.03 | 0.00 | 0 | 476 | 17 | 15 | 0/64 | 0/19 | 0/45 | - | - | - | - | - |
| 1FI1 | 0.95 | 0.00 | 0.00 | 0.96 | -0.03 | 0.00 | 0 | 657 | 25 | 11 | 0/36 | 0/36 | - | - | - | - | - | - |
| 1J8R | 0.93 | 0.00 | 0.00 | 0.98 | -0.03 | 0.00 | 0 | 175 | 3 | 10 | 0/47 | 0/47 | - | - | - | - | - | - |
| 1KQR | 0.93 | 0.00 | 0.00 | 0.99 | -0.03 | 0.00 | 0 | 138 | 2 | 8 | 1/30 | 1/30 | - | - | - | - | - | - |
| 1MOR | 0.94 | 0.00 | 0.00 | 0.98 | -0.03 | 0.00 | 0 | 301 | 5 | 13 | 0/39 | 0/39 | - | - | - | - | - | - |
| 1Q2P | 0.95 | 0.00 | 0.00 | 0.98 | -0.03 | 0.00 | 0 | 228 | 5 | 7 | 0/31 | 0/31 | - | - | - | - | - | - |
| 1U65 | 0.93 | 0.00 | 0.00 | 0.95 | -0.03 | 0.00 | 0 | 442 | 24 | 8 | 0/36 | 0/36 | - | - | - | - | - | - |
| 2C3E | 0.94 | 0.00 | 0.00 | 0.98 | -0.03 | 0.00 | 0 | 256 | 6 | 10 | 0/30 | 0/30 | - | - | - | - | - | - |
| 2DWJ | 0.94 | 0.00 | 0.00 | 0.97 | -0.03 | 0.00 | 0 | 298 | 8 | 11 | 0/42 | 0/42 | - | - | - | - | - | - |
| 2E59 | 0.93 | 0.00 | 0.00 | 0.99 | -0.03 | 0.00 | 0 | 131 | 2 | 8 | 0/37 | 0/24 | 0/13 | - | - | - | - | - |
| 2HZH | 0.94 | 0.00 | 0.00 | 0.97 | -0.03 | 0.00 | 0 | 418 | 12 | 15 | 0/58 | 0/14 | 0/12 | 0/32 | - | - | - | - |
| 2RH1 | 0.94 | 0.00 | 0.00 | 0.97 | -0.03 | 0.00 | 0 | 397 | 11 | 14 | 0/51 | 0/51 | - | - | - | - | - | - |
| 2VE0 | 0.95 | 0.00 | 0.00 | 0.97 | -0.03 | 0.00 | 0 | 424 | 12 | 12 | 0/46 | 0/36 | 0/10 | - | - | - | - | - |
| 3A7B | 0.94 | 0.00 | 0.00 | 0.95 | -0.03 | 0.00 | 0 | 484 | 25 | 6 | 0/28 | 0/28 | - | - | - | - | - | - |
| 3BKL | 0.91 | 0.00 | 0.00 | 0.92 | -0.03 | 0.00 | 0 | 479 | 42 | 6 | 0/23 | 0/23 | - | - | - | - | - | - |
| 3C02 | 0.93 | 0.00 | 0.00 | 0.96 | -0.03 | 0.00 | 0 | 192 | 9 | 5 | 0/17 | 0/17 | - | - | - | - | - | - |
| 3D3H | 0.91 | 0.00 | 0.00 | 0.99 | -0.03 | 0.00 | 0 | 140 | 1 | 13 | 0/39 | 0/39 | - | - | - | - | - | - |
| 3FP0 | 0.95 | 0.00 | 0.00 | 0.97 | -0.03 | 0.00 | 0 | 417 | 15 | 8 | 0/26 | 0/26 | - | - | - | - | - | - |
| 3HZS | 0.90 | 0.00 | 0.00 | 0.99 | -0.03 | 0.00 | 0 | 168 | 2 | 16 | 0/52 | 0/39 | 0/13 | - | - | - | - | - |
| 3I2T | 0.94 | 0.00 | 0.00 | 0.97 | -0.03 | 0.00 | 0 | 459 | 15 | 12 | 0/38 | 0/27 | 0/11 | - | - | - | - | - |
| 1EU1 | 0.91 | 0.00 | 0.00 | 0.94 | -0.04 | 0.00 | 0 | 634 | 43 | 17 | 0/47 | 0/24 | 0/23 | - | - | - | - | - |
| 1J1M | 0.91 | 0.00 | 0.00 | 0.98 | -0.04 | 0.00 | 0 | 223 | 5 | 16 | 0/56 | 0/16 | 0/40 | - | - | - | - | - |
| 1LLC | 0.93 | 0.00 | 0.00 | 0.96 | -0.04 | 0.00 | 0 | 283 | 11 | 10 | 0/32 | 0/32 | - | - | - | - | - | - |
| 1OAF | 0.92 | 0.00 | 0.00 | 0.96 | -0.04 | 0.00 | 0 | 218 | 9 | 9 | 0/32 | 0/32 | - | - | - | - | - | - |
| 1P49 | 0.92 | 0.00 | 0.00 | 0.94 | -0.04 | 0.00 | 0 | 469 | 29 | 10 | 0/26 | 0/14 | 0/12 | - | - | - | - | - |
| 1SE3 | 0.92 | 0.00 | 0.00 | 0.96 | -0.04 | 0.00 | 0 | 186 | 7 | 9 | 0/34 | 0/34 | - | - | - | - | - | - |
| 1UXT | 0.91 | 0.00 | 0.00 | 0.93 | -0.04 | 0.00 | 0 | 413 | 32 | 10 | 0/31 | 0/31 | - | - | - | - | - | - |
| 1UYQ | 0.92 | 0.00 | 0.00 | 0.94 | -0.04 | 0.00 | 0 | 364 | 25 | 8 | 0/29 | 0/29 | - | - | - | - | - | - |
| 2GY5 | 0.93 | 0.00 | 0.00 | 0.96 | -0.04 | 0.00 | 0 | 381 | 16 | 12 | 0/41 | 0/13 | 0/11 | 0/17 | - | - | - | - |
| 3A6T | 0.93 | 0.00 | 0.00 | 0.97 | -0.04 | 0.00 | 0 | 105 | 3 | 5 | 0/29 | 0/29 | - | - | - | - | - | - |
| 3DAS | 0.92 | 0.00 | 0.00 | 0.96 | -0.04 | 0.00 | 0 | 280 | 11 | 12 | 0/41 | 0/23 | 0/18 | - | - | - | - | - |
| 3DIV | 0.92 | 0.00 | 0.00 | 0.96 | -0.04 | 0.00 | 0 | 407 | 17 | 18 | 0/60 | 0/22 | 0/17 | 0/21 | - | - | - | - |
| 1ODA | 0.90 | 0.00 | 0.00 | 0.96 | -0.05 | 0.00 | 0 | 100 | 4 | 7 | 0/35 | 0/35 | - | - | - | - | - | - |
| 2BGI | 0.88 | 0.00 | 0.00 | 0.98 | -0.05 | 0.00 | 0 | 199 | 4 | 24 | 0/83 | 0/27 | 0/56 | - | - | - | - | - |
| 3FUS | 0.91 | 0.00 | 0.00 | 0.94 | -0.05 | 0.00 | 0 | 277 | 17 | 10 | 0/48 | 0/33 | 0/15 | - | - | - | - | - |
| 1DL2 | 0.89 | 0.00 | 0.00 | 0.92 | -0.06 | 0.00 | 0 | 383 | 34 | 16 | 0/60 | 0/17 | 0/14 | 0/29 | - | - | - | - |
| 2V8L | 0.88 | 0.00 | 0.00 | 0.94 | -0.07 | 0.00 | 0 | 85 | 5 | 7 | 0/36 | 0/36 | - | - | - | - | - | - |
| Total | 0.95 | 0.51 | 0.54 | 0.97 | 0.5 | 0.53 | 4323 | 156936 | 4114 | 3676 |  |  |  |  |  |  |  |  |

**Table S4:** Ten-fold cross validation ANN_BAGGING prediction accuracy benchmarks on the S497 dataset. The dataset, the ten-fold cross validation, and the benchmark measurements have been described in the main text. Matthews correlation coefficient (MCC), F-score(Fsc), Accuracy(Acc), Precision(Pre), Sensitivity(Sen) and Specificity(Spe) are shown in Equations (4)~(9). TP, FP, TN, and FN are true positive, false positive, true negative, and false negative respectively. C1~C7 represent carbohydrate binding sites in each of the test proteins; different protein has different number of binding sites. In these columns, the number of the predicted true positive atoms is shown over the actual number of atoms involving in the binding site. Interactive examination of the prediction results for each of the proteins in the S497 dataset can be accessed from the web server: <http://ismblab.genomics.sinica.edu.tw/>> benchmark > protein-carbohydrate.
