# Supplementary material for: Prediction of Carbohydrate Binding Sites on Protein Surfaces with 3-Dimensional Probability Density Distributions of Interacting Atoms
Source: PLoS One. 2012 Jul 25;7(7):e40846. doi: 10.1371/journal.pone.0040846 (PMC3405063; doi:10.1371/journal.pone.0040846)
Supplement: Table S5 — ANN_BAGGING prediction accuracy benchmarks on the independent test set S108. The dataset and the benchmark measurements have been described in the main text. Matthews correlation coefficient (MCC), F-score(Fsc), Accuracy(Acc), Precision(Pre), Sensitivity(Sen) and Specificity(Spe) are shown in Equations (4)∼(9). TP, FP, TN, and FN are true positive, false positive, true negative, and false negative respectively. C1∼C6 represent carbohydrate binding sites in each of the test proteins; different protein has different number of binding sites. In these columns, the number of the predicted true positive atoms is shown over the actual number of atoms involving in the binding site. The upper-limit of the pairwise sequence identity for each of the proteins in S108 to the homologues in S497 is shown in ID% column. Interactive examination of the prediction results for each of the proteins in the 108 independent test set can be accessed from the web server: http://ismblab.genomics.sinica.edu.tw/> benchmark > protein-carbohydrate. (DOC) [file pone.0040846.s008.doc]

**Table S**5

| **PDBID** | **Residue-based ANN_BAGGING prediction benchmarks on independent test set S108** | | | | | | | | | | | | | | | | | |
| --- | --- | --- | --- | --- | --- | --- | --- | --- | --- | --- | --- | --- | --- | --- | --- | --- | --- | --- |
|  | **Residues Level** | | | | | | | | | | **Predict positive atoms / Actual binding atoms** | | | | | | | **ID%** |
|  | **Acc** | **Pre** | **Sen** | **Spe** | **MCC** | **Fsc** | **TP** | **TN** | **FP** | **FN** | **All** | **C1** | **C2** | **C3** | **C4** | **C5** | **C6** |  |
| 3LLZ | 0.98 | 1.00 | 0.78 | 1.00 | 0.88 | 0.88 | 7 | 118 | 0 | 2 | 25/32 | 25/32 | - | - | - | - | - | 34.6 |
| 1UGX | 0.98 | 1.00 | 0.78 | 1.00 | 0.88 | 0.88 | 7 | 118 | 0 | 2 | 27/35 | 27/35 | - | - | - | - | - | 31.6 |
| 2CKR | 0.98 | 0.84 | 0.91 | 0.98 | 0.86 | 0.88 | 21 | 235 | 4 | 2 | 63/78 | 63/78 | - | - | - | - | - | 39.9 |
| 2C3X | 0.98 | 1.00 | 0.75 | 1.00 | 0.86 | 0.86 | 6 | 80 | 0 | 2 | 29/37 | 29/37 | - | - | - | - | - | 30.4 |
| 3N17 | 0.98 | 1.00 | 0.74 | 1.00 | 0.85 | 0.85 | 14 | 261 | 0 | 5 | 47/64 | 47/64 | - | - | - | - | - | 28.8 |
| 3LRL | 0.99 | 0.73 | 1.00 | 0.99 | 0.85 | 0.84 | 16 | 383 | 6 | 0 | 46/52 | 46/52 | - | - | - | - | - | 32.6 |
| 3K00 | 0.98 | 0.88 | 0.81 | 0.99 | 0.83 | 0.84 | 21 | 330 | 3 | 5 | 70/95 | 70/95 | - | - | - | - | - | 26.9 |
| 3I8T | 0.98 | 0.70 | 1.00 | 0.98 | 0.83 | 0.82 | 7 | 117 | 3 | 0 | 21/26 | 21/26 | - | - | - | - | - | 43.9 |
| 4GAL | 0.98 | 0.70 | 1.00 | 0.98 | 0.83 | 0.82 | 7 | 121 | 3 | 0 | 27/31 | 27/31 | - | - | - | - | - | 36.3 |
| 1S1A | 0.98 | 0.75 | 0.92 | 0.98 | 0.82 | 0.83 | 12 | 197 | 4 | 1 | 33/41 | 33/41 | - | - | - | - | - | 47.2 |
| 2GDV | 0.99 | 0.71 | 0.92 | 0.99 | 0.80 | 0.80 | 12 | 442 | 5 | 1 | 41/44 | 41/44 | - | - | - | - | - | 28.0 |
| 2H5Z | 0.97 | 0.80 | 0.80 | 0.98 | 0.78 | 0.80 | 8 | 101 | 2 | 2 | 28/47 | 28/47 | - | - | - | - | - | 42.6 |
| 3LL2 | 0.94 | 0.82 | 0.78 | 0.97 | 0.77 | 0.80 | 14 | 94 | 3 | 4 | 46/67 | 46/67 | - | - | - | - | - | 35.8 |
| 3PAK | 0.98 | 0.71 | 0.83 | 0.99 | 0.76 | 0.77 | 5 | 129 | 2 | 1 | 10/23 | 10/23 | - | - | - | - | - | 23.7 |
| 2MSB | 0.97 | 0.83 | 0.71 | 0.99 | 0.76 | 0.77 | 5 | 94 | 1 | 2 | 14/24 | 14/24 | - | - | - | - | - | 29.6 |
| 2XSB | 0.98 | 0.69 | 0.85 | 0.99 | 0.75 | 0.76 | 11 | 358 | 5 | 2 | 34/44 | 34/44 | - | - | - | - | - | 30.7 |
| 2AAI | 0.95 | 0.90 | 0.67 | 0.99 | 0.75 | 0.77 | 18 | 212 | 2 | 9 | 60/108 | 20/33 | 23/49 | 17/26 |  |  |  | 47.0 |
| 2XD3 | 0.97 | 0.90 | 0.65 | 0.99 | 0.75 | 0.76 | 17 | 306 | 2 | 9 | 66/102 | 66/102 | - | - | - | - | - | 30.5 |
| 2Y24 | 0.98 | 0.67 | 0.86 | 0.98 | 0.74 | 0.75 | 12 | 314 | 6 | 2 | 47/62 | 47/62 | - | - | - | - | - | 19.3 |
| 1NSR | 0.98 | 0.56 | 1.00 | 0.98 | 0.74 | 0.72 | 9 | 301 | 7 | 0 | 28/28 | 28/28 | - | - | - | - | - | 30.8 |
| 1SLA | 0.96 | 0.80 | 0.73 | 0.98 | 0.74 | 0.76 | 8 | 112 | 2 | 3 | 22/36 | 22/36 | - | - | - | - | - | 35.1 |
| 2X2T | 0.97 | 0.67 | 0.86 | 0.98 | 0.74 | 0.75 | 6 | 126 | 3 | 1 | 24/37 | 24/37 | - | - | - | - | - | 22.9 |
| 2V73 | 0.98 | 1.00 | 0.56 | 1.00 | 0.74 | 0.71 | 5 | 155 | 0 | 4 | 15/33 | 15/33 | - | - | - | - | - | 36.1 |
| 3ANK | 0.97 | 0.56 | 1.00 | 0.97 | 0.74 | 0.71 | 10 | 292 | 8 | 0 | 31/31 | 31/31 | - | - | - | - | - | 25.9 |
| 3NV3 | 0.96 | 0.64 | 0.88 | 0.97 | 0.73 | 0.74 | 7 | 120 | 4 | 1 | 24/31 | 24/31 | - | - | - | - | - | 42.8 |
| 1FGG | 0.97 | 0.73 | 0.73 | 0.99 | 0.71 | 0.73 | 8 | 210 | 3 | 3 | 31/39 | 31/39 | - | - | - | - | - | 29.5 |
| 3ILF | 0.95 | 0.59 | 0.87 | 0.96 | 0.69 | 0.70 | 13 | 209 | 9 | 2 | 47/51 | 47/51 | - | - | - | - | - | 29.9 |
| 3M3O | 0.97 | 0.75 | 0.67 | 0.99 | 0.69 | 0.71 | 6 | 137 | 2 | 3 | 18/32 | 18/32 | - | - | - | - | - | 23.4 |
| 1PUU | 0.96 | 0.71 | 0.71 | 0.98 | 0.68 | 0.71 | 12 | 225 | 5 | 5 | 39/62 | 16/32 | 23/30 | - | - | - | - | 42.2 |
| 3AFL | 0.98 | 0.55 | 0.84 | 0.98 | 0.67 | 0.67 | 16 | 662 | 13 | 3 | 62/72 | 62/72 | - | - | - | - | - | 12.9 |
| 2R6G | 0.99 | 0.78 | 0.58 | 1.00 | 0.67 | 0.67 | 7 | 449 | 2 | 5 | 24/46 | 24/46 | - | - | - | - | - | 18.5 |
| 3A4A | 0.97 | 0.46 | 1.00 | 0.96 | 0.66 | 0.63 | 15 | 489 | 18 | 0 | 36/36 | 36/36 | - | - | - | - | - | 35.3 |
| 3M9X | 0.96 | 0.45 | 1.00 | 0.96 | 0.66 | 0.62 | 9 | 236 | 11 | 0 | 28/29 | 28/29 | - | - | - | - | - | 22.7 |
| 3OEB | 0.95 | 0.75 | 0.60 | 0.98 | 0.65 | 0.67 | 6 | 115 | 2 | 4 | 29/47 | 29/47 | - | - | - | - | - | 25.0 |
| 1JSH | 0.98 | 0.86 | 0.50 | 1.00 | 0.64 | 0.63 | 6 | 282 | 1 | 6 | 15/38 | 15/38 | - | - | - | - | - | 20.1 |
| 2YG0 | 0.94 | 0.43 | 1.00 | 0.93 | 0.63 | 0.60 | 6 | 112 | 8 | 0 | 27/27 | 27/27 | - | - | - | - | - | 42.6 |
| 3AB6 | 0.92 | 0.80 | 0.53 | 0.98 | 0.61 | 0.64 | 8 | 91 | 2 | 7 | 14/47 | 14/47 | - | - | - | - | - | 31.2 |
| 3NJV | 0.96 | 0.70 | 0.56 | 0.99 | 0.61 | 0.62 | 14 | 433 | 6 | 11 | 30/71 | 30/71 | - | - | - | - | - | 25.8 |
| 3LEI | 0.95 | 0.56 | 0.71 | 0.97 | 0.61 | 0.63 | 5 | 114 | 4 | 2 | 20/24 | 20/24 | - | - | - | - | - | 34.0 |
| 2X42 | 0.97 | 0.38 | 1.00 | 0.97 | 0.60 | 0.55 | 12 | 597 | 20 | 0 | 35/35 | 35/35 | - | - | - | - | - | 23.6 |
| 2J1V | 0.96 | 0.67 | 0.57 | 0.98 | 0.60 | 0.62 | 4 | 115 | 2 | 3 | 15/29 | 15/29 | - | - | - | - | - | 33.1 |
| 1CLY | 0.97 | 1.00 | 0.36 | 1.00 | 0.59 | 0.53 | 4 | 190 | 0 | 7 | 30/59 | 30/59 | - | - | - | - | - | 25.1 |
| 1MFB | 0.96 | 0.60 | 0.60 | 0.98 | 0.58 | 0.60 | 6 | 182 | 4 | 4 | 32/49 | 32/49 | - | - | - | - | - | 21.9 |
| 1JSO | 0.96 | 0.50 | 0.69 | 0.97 | 0.57 | 0.58 | 9 | 267 | 9 | 4 | 19/38 | 19/38 | - | - | - | - | - | 15.1 |
| 1MFA | 0.94 | 0.50 | 0.71 | 0.95 | 0.57 | 0.59 | 5 | 96 | 5 | 2 | 22/36 | 22/36 | - | - | - | - | - | 29.2 |
| 3DV6 | 0.94 | 0.67 | 0.50 | 0.98 | 0.55 | 0.57 | 4 | 98 | 2 | 4 | 11/29 | 11/29 | - | - | - | - | - | 24.0 |
| 2WT0 | 0.95 | 0.36 | 0.89 | 0.95 | 0.55 | 0.52 | 8 | 265 | 14 | 1 | 29/35 | 29/35 | - | - | - | - | - | 17.2 |
| 1TVP | 0.93 | 0.36 | 0.90 | 0.93 | 0.54 | 0.51 | 9 | 216 | 16 | 1 | 29/37 | 29/37 | - | - | - | - | - | 42.0 |
| 1D9U | 0.88 | 1.00 | 0.32 | 1.00 | 0.53 | 0.49 | 8 | 121 | 0 | 17 | 32/121 | 32/121 | - | - | - | - | - | 23.4 |
| 3HTT | 0.96 | 0.54 | 0.54 | 0.98 | 0.52 | 0.54 | 7 | 286 | 6 | 6 | 21/43 | 21/43 | - | - | - | - | - | 32.4 |
| 2XOM | 0.95 | 0.57 | 0.50 | 0.98 | 0.51 | 0.53 | 4 | 117 | 3 | 4 | 28/40 | 28/40 | - | - | - | - | - | 36.2 |
| 1S3K | 0.96 | 0.67 | 0.40 | 0.99 | 0.50 | 0.50 | 4 | 181 | 2 | 6 | 30/56 | 30/56 | - | - | - | - | - | 27.9 |
| 3ALG | 0.94 | 0.50 | 0.56 | 0.97 | 0.50 | 0.53 | 10 | 284 | 10 | 8 | 31/65 | 31/65 | - | - | - | - | - | 32.0 |
| 3AIB | 0.96 | 0.25 | 1.00 | 0.96 | 0.49 | 0.40 | 10 | 753 | 30 | 0 | 32/35 | 32/35 | - | - | - | - | - | 16.8 |
| 2ZL7 | 0.97 | 0.75 | 0.33 | 1.00 | 0.49 | 0.46 | 3 | 253 | 1 | 6 | 8/25 | 8/25 | - | - | - | - | - | 41.7 |
| 2FV0 | 0.94 | 0.38 | 0.69 | 0.95 | 0.48 | 0.49 | 9 | 285 | 15 | 4 | 25/42 | 25/42 | - | - | - | - | - | 27.9 |
| 3ACH | 0.96 | 0.57 | 0.44 | 0.98 | 0.48 | 0.50 | 4 | 167 | 3 | 5 | 24/48 | 24/48 | - | - | - | - | - | 22.7 |
| 3QOM | 0.95 | 0.28 | 0.89 | 0.95 | 0.48 | 0.42 | 8 | 407 | 21 | 1 | 22/27 | 22/27 | - | - | - | - | - | 31.4 |
| 2WCO | 0.96 | 0.23 | 1.00 | 0.96 | 0.47 | 0.38 | 9 | 643 | 30 | 0 | 41/45 | 41/45 | - | - | - | - | - | 40.0 |
| 3EDD | 0.96 | 0.33 | 0.67 | 0.97 | 0.46 | 0.44 | 8 | 530 | 16 | 4 | 29/54 | 29/54 | - | - | - | - | - | 24.1 |
| 2UVF | 0.93 | 0.27 | 0.73 | 0.94 | 0.42 | 0.39 | 11 | 446 | 30 | 4 | 29/43 | 29/43 | - | - | - | - | - | 16.3 |
| 3NSN | 0.97 | 0.30 | 0.60 | 0.97 | 0.41 | 0.40 | 6 | 505 | 14 | 4 | 28/47 | 28/47 | - | - | - | - | - | 25.2 |
| 3ESW | 0.97 | 0.40 | 0.44 | 0.98 | 0.40 | 0.42 | 4 | 301 | 6 | 5 | 14/41 | 14/41 | - | - | - | - | - | 23.4 |
| 1K72 | 0.95 | 0.28 | 0.64 | 0.96 | 0.40 | 0.39 | 9 | 491 | 23 | 5 | 35/62 | 35/62 | - | - | - | - | - | 32.1 |
| 2Y5E | 0.97 | 0.31 | 0.53 | 0.98 | 0.39 | 0.39 | 8 | 753 | 18 | 7 | 28/56 | 28/56 | - | - | - | - | - | 39.4 |
| 2RFT | 0.96 | 0.75 | 0.19 | 1.00 | 0.36 | 0.30 | 3 | 317 | 1 | 13 | 5/45 | 5/45 | - | - | - | - | - | 34.0 |
| 3N1D | 0.96 | 0.33 | 0.43 | 0.97 | 0.36 | 0.38 | 3 | 212 | 6 | 4 | 11/29 | 11/29 | - | - | - | - | - | 35.8 |
| 3O0W | 0.93 | 1.00 | 0.11 | 1.00 | 0.31 | 0.19 | 2 | 210 | 0 | 17 | 11/59 | 11/59 | - | - | - | - | - | 28.6 |
| 3KLL | 0.95 | 0.30 | 0.31 | 0.97 | 0.28 | 0.31 | 10 | 878 | 23 | 22 | 33/125 | 33/125 | - | - | - | - | - | 14.8 |
| 3FRO | 0.95 | 0.29 | 0.31 | 0.97 | 0.27 | 0.30 | 4 | 364 | 10 | 9 | 12/33 | 12/33 | - | - | - | - | - | 32.6 |
| 3N98 | 0.95 | 0.22 | 0.29 | 0.97 | 0.23 | 0.25 | 4 | 464 | 14 | 10 | 21/72 | 0/10 | 21/62 | - | - | - | - | 26.2 |
| 3LE7 | 0.91 | 0.20 | 0.33 | 0.94 | 0.22 | 0.25 | 3 | 188 | 12 | 6 | 10/30 | 10/30 | - | - | - | - | - | 29.5 |
| 2XFD | 0.93 | 0.33 | 0.17 | 0.98 | 0.20 | 0.22 | 1 | 92 | 2 | 5 | 2/29 | 2/29 | - | - | - | - | - | 17.9 |
| 2QLX | 0.87 | 0.25 | 0.22 | 0.93 | 0.16 | 0.24 | 2 | 84 | 6 | 7 | 7/28 | 7/28 | - | - | - | - | - | 38.9 |
| 1Y75 | 0.84 | 0.25 | 0.06 | 0.97 | 0.06 | 0.10 | 1 | 94 | 3 | 15 | 2/49 | 2/49 | - | - | - | - | - | 36.4 |
| 1ND5 | 0.93 | 0.07 | 0.10 | 0.96 | 0.05 | 0.08 | 1 | 293 | 13 | 9 | 0/48 | 0/31 | 0/17 | - | - | - | - | 22.3 |
| 1ABR | 0.87 | 0.10 | 0.13 | 0.92 | 0.04 | 0.11 | 2 | 216 | 18 | 14 | 11/65 | 0/36 | 11/29 | - | - | - | - | 42.0 |
| 1O84 | 0.00 | 0.00 | 0.00 | 0.00 | 0.00 | 0.00 | 0 | 52 | 0 | 9 | 0/24 | 0/24 | - | - | - | - | - | 34.3 |
| 1LQ8 | 0.00 | 0.00 | 0.00 | 0.00 | 0.00 | 0.00 | 0 | 309 | 0 | 5 | 0/23 | 0/23 | - | - | - | - | - | 47.1 |
| 3A3Y | 0.00 | 0.00 | 0.00 | 0.00 | 0.00 | 0.00 | 0 | 249 | 0 | 7 | 0/27 | 0/11 | 0/16 | - | - | - | - | 28.2 |
| 3DGY | 0.00 | 0.00 | 0.00 | 0.00 | 0.00 | 0.00 | 0 | 81 | 0 | 5 | 0/24 | 0/24 | - | - | - | - | - | 33.0 |
| 3IC3 | 0.00 | 0.00 | 0.00 | 0.00 | 0.00 | 0.00 | 0 | 86 | 0 | 4 | 0/24 | 0/24 | - | - | - | - | - | 35.6 |
| 2F2E | 0.00 | 0.00 | 0.00 | 0.00 | 0.00 | 0.00 | 0 | 131 | 0 | 5 | 0/28 | 0/28 | - | - | - | - | - | 37.7 |
| 1U4J | 0.00 | 0.00 | 0.00 | 0.00 | 0.00 | 0.00 | 0 | 105 | 0 | 6 | 0/30 | 0/30 | - | - | - | - | - | 36.4 |
| 3EPZ | 0.00 | 0.00 | 0.00 | 0.00 | 0.00 | 0.00 | 0 | 194 | 0 | 9 | 0/33 | 0/33 | - | - | - | - | - | 19.0 |
| 1M7D | 0.00 | 0.00 | 0.00 | 0.00 | 0.00 | 0.00 | 0 | 197 | 0 | 7 | 0/27 | 0/27 | - | - | - | - | - | 25.6 |
| 1NI6 | 0.00 | 0.00 | 0.00 | 0.00 | 0.00 | 0.00 | 0 | 208 | 0 | 7 | 0/28 | 0/28 | - | - | - | - | - | 21.0 |
| 2C4F | 0.00 | 0.00 | 0.00 | 0.00 | 0.00 | 0.00 | 0 | 118 | 0 | 10 | 0/34 | 0/15 | 0/19 | - | - | - | - | 21.8 |
| 1H7T | 0.00 | 0.00 | 0.00 | 0.00 | 0.00 | 0.00 | 0 | 209 | 0 | 9 | 0/29 | 0/29 | - | - | - | - | - | 31.8 |
| 2ESR | 0.00 | 0.00 | 0.00 | 0.00 | 0.00 | 0.00 | 0 | 138 | 0 | 9 | 0/36 | 0/36 | - | - | - | - | - | 33.3 |
| 3FL3 | 0.00 | 0.00 | 0.00 | 0.00 | 0.00 | 0.00 | 0 | 104 | 0 | 11 | 0/37 | 0/37 | - | - | - | - | - | 33.9 |
| 3BL8 | 0.98 | 0.00 | 0.00 | 0.99 | -0.01 | 0.00 | 0 | 495 | 4 | 7 | 0/27 | 0/27 | - | - | - | - | - | 34.1 |
| 3OBT | 0.97 | 0.00 | 0.00 | 1.00 | -0.01 | 0.00 | 0 | 377 | 2 | 9 | 0/35 | 0/35 | - | - | - | - | - | 38.7 |
| 2WR3 | 0.96 | 0.00 | 0.00 | 0.99 | -0.02 | 0.00 | 0 | 439 | 5 | 12 | 0/36 | 0/36 | - | - | - | - | - | 16.2 |
| 1JX2 | 0.96 | 0.00 | 0.00 | 0.97 | -0.02 | 0.00 | 0 | 668 | 23 | 7 | 0/25 | 0/25 | - | - | - | - | - | 16.4 |
| 3JU4 | 0.96 | 0.00 | 0.00 | 0.97 | -0.02 | 0.00 | 0 | 595 | 19 | 7 | 0/25 | 0/25 | - | - | - | - | - | 22.1 |
| 2QFR | 0.96 | 0.00 | 0.00 | 0.97 | -0.02 | 0.00 | 0 | 363 | 10 | 6 | 0/26 | 0/11 | 0/15 | - | - | - | - | 19.8 |
| 2Z64 | 0.95 | 0.00 | 0.00 | 0.96 | -0.02 | 0.00 | 0 | 537 | 25 | 6 | 0/28 | 0/28 | - | - | - | - | - | 29.1 |
| 2GK1 | 0.95 | 0.00 | 0.00 | 0.96 | -0.02 | 0.00 | 0 | 402 | 17 | 6 | 0/26 | 0/26 | - | - | - | - | - | 28.2 |
| 2XJP | 0.92 | 0.00 | 0.00 | 0.99 | -0.03 | 0.00 | 0 | 203 | 2 | 15 | 0/50 | 0/31 | 0/19 | - | - | - | - | 27.5 |
| 3PIC | 0.95 | 0.00 | 0.00 | 0.97 | -0.03 | 0.00 | 0 | 297 | 9 | 8 | 0/26 | 0/26 | - | - | - | - | - | 19.5 |
| 2ZOE | 0.94 | 0.00 | 0.00 | 0.96 | -0.03 | 0.00 | 0 | 370 | 15 | 8 | 0/34 | 0/34 | - | - | - | - | - | 26.2 |
| 1RYD | 0.92 | 0.00 | 0.00 | 0.94 | -0.03 | 0.00 | 0 | 314 | 20 | 6 | 0/24 | 0/24 | - | - | - | - | - | 20.9 |
| 3A9G | 0.93 | 0.00 | 0.00 | 0.96 | -0.04 | 0.00 | 0 | 273 | 12 | 8 | 0/39 | 0/14 | 0/25 | - | - | - | - | 39.8 |
| 3AJ6 | 0.93 | 0.00 | 0.00 | 0.96 | -0.04 | 0.00 | 0 | 237 | 9 | 9 | 0/29 | 0/29 | - | - | - | - | - | 12.9 |
| 3FW3 | 0.91 | 0.00 | 0.00 | 0.94 | -0.04 | 0.00 | 0 | 192 | 12 | 6 | 0/30 | 0/30 | - | - | - | - | - | 35.7 |
| 2H6O | 0.87 | 0.00 | 0.00 | 0.98 | -0.05 | 0.00 | 0 | 359 | 7 | 46 | 0/136 | 0/41 | 0/27 | 0/22 | 0/12 | 0/19 | 0/15 | 17.2 |
| 2CL8 | 0.88 | 0.00 | 0.00 | 0.95 | -0.06 | 0.00 | 0 | 106 | 6 | 8 | 0/28 | 0/28 | - | - | - | - | - | 30.2 |
| Total | 0.96 | 0.45 | 0.49 | 0.97 | 0.45 | 0.47 | 615 | 29249 | 766 | 631 |  |  |  |  |  |  |  |  |

**Table S5:** ANN_BAGGING prediction accuracy benchmarks on the independent test set S108. The dataset and the benchmark measurements have been described in the main text. Matthews correlation coefficient (MCC), F-score(Fsc), Accuracy(Acc), Precision(Pre), Sensitivity(Sen) and Specificity(Spe) are shown in Equations (4)~(9). TP, FP, TN, and FN are true positive, false positive, true negative, and false negative respectively. C1~C6 represent carbohydrate binding sites in each of the test proteins; different protein has different number of binding sites. In these columns, the number of the predicted true positive atoms is shown over the actual number of atoms involving in the binding site. The upper-limit of the pairwise sequence identity for each of the proteins in S108 to the homologues in S497 is shown in ID% column. Interactive examination of the prediction results for each of the proteins in the 108 independent test set can be accessed from the web server: <http://ismblab.genomics.sinica.edu.tw/>> benchmark > protein-carbohydrate
