# Supplementary material for: Prediction of Carbohydrate Binding Sites on Protein Surfaces with 3-Dimensional Probability Density Distributions of Interacting Atoms
Source: PLoS One. 2012 Jul 25;7(7):e40846. doi: 10.1371/journal.pone.0040846 (PMC3405063; doi:10.1371/journal.pone.0040846)
Supplement: Table S6 — ANN_BAGGING prediction accuracy benchmarks on the unbound set S88. The dataset and the benchmark measurements have been described in the main text. Matthews correlation coefficient (MCC), F-score(Fsc), Accuracy(Acc), Precision(Pre), Sensitivity(Sen) and Specificity(Spe) are shown in Equations (4)∼(9). TP, FP, TN, and FN are true positive, false positive, true negative, and false negative respectively. Interactive examination of the prediction results for each of the proteins in the 88 unbound test set can be accessed from the web server: http://ismblab.genomics.sinica.edu.tw/> benchmark > protein-carbohydrate. (DOC) [file pone.0040846.s009.doc]

**Table S6**

| **Residue-based ANN_BAGGING prediction benchmarks on unbound set S88** | | | | | | | | | | |
| --- | --- | --- | --- | --- | --- | --- | --- | --- | --- | --- |
| **PDBID** | **Acc** | **Pre** | **Sen** | **Spe** | **MCC** | **Fsc** | **TP** | **TN** | **FP** | **FN** |
| 153L | 0.00 | 0.00 | 0.00 | 0.00 | 0.00 | 0.00 | 0 | 139 | 0 | 16 |
| 1AVA | 0.95 | 0.50 | 0.70 | 0.96 | 0.57 | 0.58 | 14 | 345 | 14 | 6 |
| 1B0I | 0.96 | 0.63 | 0.71 | 0.97 | 0.65 | 0.67 | 17 | 382 | 10 | 7 |
| 1B1X | 0.96 | 0.00 | 0.00 | 0.97 | -0.02 | 0.00 | 0 | 600 | 16 | 10 |
| 1BB6 | 0.90 | 0.69 | 0.53 | 0.96 | 0.55 | 0.60 | 9 | 97 | 4 | 8 |
| 1BJQ | 0.99 | 1.00 | 0.73 | 1.00 | 0.85 | 0.84 | 8 | 217 | 0 | 3 |
| 1C1F | 0.98 | 0.89 | 0.89 | 0.99 | 0.88 | 0.89 | 8 | 119 | 1 | 1 |
| 1C3K | 0.97 | 0.86 | 0.67 | 0.99 | 0.74 | 0.75 | 6 | 121 | 1 | 3 |
| 1C7P | 0.88 | 0.55 | 0.38 | 0.95 | 0.39 | 0.44 | 6 | 103 | 5 | 10 |
| 1CWY | 0.92 | 0.39 | 0.45 | 0.95 | 0.37 | 0.42 | 14 | 411 | 22 | 17 |
| 1DQG | 0.93 | 0.67 | 0.20 | 0.99 | 0.34 | 0.31 | 2 | 112 | 1 | 8 |
| 1E3X | 0.94 | 0.70 | 0.40 | 0.99 | 0.50 | 0.51 | 14 | 396 | 6 | 21 |
| 1E7M | 0.99 | 1.00 | 0.42 | 1.00 | 0.64 | 0.59 | 5 | 442 | 0 | 7 |
| 1EOK | 0.94 | 0.71 | 0.55 | 0.98 | 0.59 | 0.62 | 12 | 216 | 5 | 10 |
| 1ESL | 0.97 | 1.00 | 0.58 | 1.00 | 0.75 | 0.74 | 7 | 133 | 0 | 5 |
| 1EUR | 0.98 | 0.86 | 0.75 | 0.99 | 0.79 | 0.80 | 12 | 303 | 2 | 4 |
| 1FCQ | 0.95 | 0.59 | 0.72 | 0.97 | 0.63 | 0.65 | 13 | 255 | 9 | 5 |
| 1G01 | 0.95 | 0.35 | 0.78 | 0.96 | 0.50 | 0.48 | 7 | 292 | 13 | 2 |
| 1G6V | 0.92 | 0.50 | 0.39 | 0.97 | 0.40 | 0.44 | 7 | 196 | 7 | 11 |
| 1G95 | 0.00 | 0.00 | 0.00 | 0.00 | 0.00 | 0.00 | 0 | 386 | 0 | 24 |
| 1GJU | 0.96 | 0.50 | 0.40 | 0.98 | 0.43 | 0.44 | 10 | 561 | 10 | 15 |
| 1GMM | 0.88 | 0.71 | 0.31 | 0.98 | 0.42 | 0.44 | 5 | 93 | 2 | 11 |
| 1GOK | 0.94 | 0.87 | 0.50 | 0.99 | 0.63 | 0.63 | 13 | 230 | 2 | 13 |
| 1HDK | 0.91 | 0.31 | 0.83 | 0.91 | 0.47 | 0.46 | 5 | 109 | 11 | 1 |
| 1HJZ | 0.00 | 0.00 | 0.00 | 0.00 | 0.00 | 0.00 | 0 | 157 | 0 | 12 |
| 1IA6 | 0.96 | 0.33 | 0.89 | 0.96 | 0.53 | 0.49 | 8 | 350 | 16 | 1 |
| 1J7I | 0.94 | 0.25 | 0.17 | 0.97 | 0.17 | 0.20 | 2 | 229 | 6 | 10 |
| 1JXK | 0.91 | 0.66 | 0.43 | 0.97 | 0.49 | 0.52 | 21 | 384 | 11 | 28 |
| 1KBZ | 0.96 | 0.44 | 0.44 | 0.98 | 0.43 | 0.44 | 4 | 257 | 5 | 5 |
| 1KIT | 0.96 | 0.51 | 0.68 | 0.97 | 0.57 | 0.58 | 21 | 654 | 20 | 10 |
| 1KNL | 0.91 | 0.63 | 0.94 | 0.91 | 0.72 | 0.75 | 15 | 88 | 9 | 1 |
| 1KWG | 0.99 | 0.57 | 1.00 | 0.98 | 0.75 | 0.73 | 12 | 567 | 9 | 0 |
| 1L1Q | 0.95 | 0.50 | 0.33 | 0.98 | 0.38 | 0.40 | 3 | 154 | 3 | 6 |
| 1LSG | 0.91 | 1.00 | 0.29 | 1.00 | 0.52 | 0.46 | 5 | 118 | 0 | 12 |
| 1LSY | 0.93 | 1.00 | 0.53 | 1.00 | 0.70 | 0.69 | 9 | 101 | 0 | 8 |
| 1MOS | 0.91 | 0.00 | 0.00 | 0.97 | -0.04 | 0.00 | 0 | 292 | 10 | 18 |
| 1MWO | 0.88 | 0.63 | 0.46 | 0.96 | 0.48 | 0.53 | 24 | 296 | 14 | 28 |
| 1O8P | 0.87 | 0.33 | 0.25 | 0.94 | 0.22 | 0.29 | 3 | 100 | 6 | 9 |
| 1OGM | 0.96 | 0.19 | 0.36 | 0.97 | 0.24 | 0.25 | 4 | 504 | 17 | 7 |
| 1OLR | 0.93 | 0.65 | 0.77 | 0.95 | 0.67 | 0.71 | 17 | 164 | 9 | 5 |
| 1PBN | 0.96 | 0.60 | 0.60 | 0.98 | 0.58 | 0.60 | 9 | 245 | 6 | 6 |
| 1PIF | 0.89 | 0.41 | 0.26 | 0.96 | 0.27 | 0.32 | 12 | 388 | 17 | 35 |
| 1Q50 | 0.97 | 0.17 | 0.09 | 0.99 | 0.11 | 0.12 | 1 | 501 | 5 | 10 |
| 1QAZ | 0.96 | 0.54 | 0.58 | 0.98 | 0.54 | 0.56 | 7 | 291 | 6 | 5 |
| 1RWA | 0.97 | 0.57 | 0.87 | 0.98 | 0.69 | 0.69 | 20 | 652 | 15 | 3 |
| 1S2O | 0.93 | 0.00 | 0.00 | 1.00 | -0.02 | 0.00 | 0 | 186 | 1 | 13 |
| 1U9V | 0.00 | 0.00 | 0.00 | 0.00 | 0.00 | 0.00 | 0 | 180 | 0 | 12 |
| 1UG9 | 0.97 | 0.31 | 0.83 | 0.98 | 0.50 | 0.46 | 10 | 879 | 22 | 2 |
| 1UXZ | 0.83 | 0.23 | 0.23 | 0.91 | 0.14 | 0.23 | 3 | 97 | 10 | 10 |
| 1UY1 | 0.93 | 1.00 | 0.39 | 1.00 | 0.60 | 0.56 | 5 | 105 | 0 | 8 |
| 1W0N | 0.98 | 1.00 | 0.67 | 1.00 | 0.81 | 0.80 | 4 | 104 | 0 | 2 |
| 1W9S | 0.88 | 0.50 | 0.57 | 0.92 | 0.47 | 0.53 | 8 | 97 | 8 | 6 |
| 1WP6 | 0.89 | 0.69 | 0.44 | 0.97 | 0.49 | 0.54 | 27 | 347 | 12 | 35 |
| 1WU4 | 0.95 | 0.38 | 1.00 | 0.95 | 0.60 | 0.55 | 9 | 304 | 15 | 0 |
| 1X1H | 0.96 | 0.30 | 1.00 | 0.96 | 0.53 | 0.46 | 11 | 634 | 26 | 0 |
| 1ZR3 | 0.00 | 0.00 | 0.00 | 0.00 | 0.00 | 0.00 | 0 | 149 | 0 | 12 |
| 1ZTY | 0.93 | 0.08 | 0.05 | 0.97 | 0.03 | 0.06 | 1 | 441 | 12 | 19 |
| 2A8Z | 0.93 | 0.67 | 0.56 | 0.97 | 0.58 | 0.61 | 18 | 290 | 9 | 14 |
| 2E2N | 0.96 | 0.68 | 0.83 | 0.97 | 0.73 | 0.75 | 15 | 228 | 7 | 3 |
| 2G3I | 0.96 | 0.59 | 0.67 | 0.97 | 0.60 | 0.63 | 10 | 244 | 7 | 5 |
| 2HQQ | 0.95 | 0.50 | 0.85 | 0.96 | 0.63 | 0.63 | 11 | 242 | 11 | 2 |
| 2JEP | 0.94 | 0.52 | 0.63 | 0.96 | 0.55 | 0.57 | 12 | 294 | 11 | 7 |
| 2JF2 | 0.97 | 1.00 | 0.36 | 1.00 | 0.59 | 0.53 | 4 | 224 | 0 | 7 |
| 2LBD | 0.00 | 0.00 | 0.00 | 0.00 | 0.00 | 0.00 | 0 | 205 | 0 | 11 |
| 2O48 | 0.97 | 0.46 | 0.67 | 0.98 | 0.54 | 0.55 | 6 | 277 | 7 | 3 |
| 2O9F | 0.96 | 0.00 | 0.00 | 0.99 | -0.02 | 0.00 | 0 | 202 | 2 | 7 |
| 2O9P | 0.96 | 0.63 | 0.80 | 0.97 | 0.69 | 0.70 | 20 | 357 | 12 | 5 |
| 2R60 | 0.00 | 0.00 | 0.00 | 0.00 | 0.00 | 0.00 | 0 | 389 | 0 | 15 |
| 2V0H | 0.97 | 0.86 | 0.35 | 1.00 | 0.54 | 0.50 | 6 | 395 | 1 | 11 |
| 2V4M | 0.96 | 1.00 | 0.21 | 1.00 | 0.46 | 0.35 | 3 | 295 | 0 | 11 |
| 2V8I | 0.97 | 0.50 | 0.57 | 0.98 | 0.52 | 0.53 | 8 | 476 | 8 | 6 |
| 2VJI | 0.95 | 0.62 | 0.51 | 0.98 | 0.54 | 0.56 | 18 | 485 | 11 | 17 |
| 2VK5 | 0.96 | 0.55 | 0.94 | 0.96 | 0.70 | 0.69 | 17 | 365 | 14 | 1 |
| 2VUJ | 0.97 | 0.82 | 0.90 | 0.97 | 0.84 | 0.86 | 18 | 152 | 4 | 2 |
| 2VW0 | 0.99 | 0.77 | 0.72 | 0.99 | 0.74 | 0.74 | 13 | 583 | 4 | 5 |
| 2VX4 | 0.97 | 0.76 | 0.86 | 0.98 | 0.80 | 0.81 | 19 | 287 | 6 | 3 |
| 2W1W | 0.95 | 1.00 | 0.40 | 1.00 | 0.62 | 0.57 | 4 | 109 | 0 | 6 |
| 2YVM | 0.93 | 0.50 | 0.25 | 0.98 | 0.32 | 0.33 | 3 | 156 | 3 | 9 |
| 3A64 | 0.95 | 0.62 | 0.70 | 0.97 | 0.63 | 0.65 | 16 | 305 | 10 | 7 |
| 3BHS | 0.95 | 0.00 | 0.00 | 0.99 | -0.02 | 0.00 | 0 | 311 | 4 | 13 |
| 3BMV | 0.93 | 0.40 | 0.41 | 0.96 | 0.37 | 0.41 | 14 | 542 | 21 | 20 |
| 3BYL | 0.96 | 0.54 | 0.74 | 0.97 | 0.61 | 0.62 | 14 | 359 | 12 | 5 |
| 3CMJ | 0.96 | 0.48 | 1.00 | 0.96 | 0.68 | 0.65 | 13 | 365 | 14 | 0 |
| 3CSR | 0.00 | 0.00 | 0.00 | 0.00 | 0.00 | 0.00 | 0 | 114 | 0 | 14 |
| 3EWQ | 0.92 | 0.67 | 0.15 | 0.99 | 0.29 | 0.25 | 2 | 134 | 1 | 11 |
| 3EZ8 | 0.98 | 0.83 | 0.83 | 0.99 | 0.82 | 0.83 | 19 | 436 | 4 | 4 |
| 3GNO | 0.96 | 0.48 | 0.93 | 0.97 | 0.65 | 0.63 | 13 | 389 | 14 | 1 |
| 3PFK | 0.95 | 0.00 | 0.00 | 0.99 | -0.02 | 0.00 | 0 | 250 | 2 | 10 |
| Total | 0.95 | 0.55 | 0.49 | 0.98 | 0.49 | 0.52 | 765 | 25733 | 630 | 791 |

**Table S6:** ANN_BAGGING prediction accuracy benchmarks on the unbound set S88. The dataset and the benchmark measurements have been described in the main text. Matthews correlation coefficient (MCC), F-score(Fsc), Accuracy(Acc), Precision(Pre), Sensitivity(Sen) and Specificity(Spe) are shown in Equations (4)~(9). TP, FP, TN, and FN are true positive, false positive, true negative, and false negative respectively. Interactive examination of the prediction results for each of the proteins in the 88 unbound test set can be accessed from the web server: <http://ismblab.genomics.sinica.edu.tw/>> benchmark > protein-carbohydrate
