# Supplementary material for: Prediction of Carbohydrate Binding Sites on Protein Surfaces with 3-Dimensional Probability Density Distributions of Interacting Atoms
Source: PLoS One. 2012 Jul 25;7(7):e40846. doi: 10.1371/journal.pone.0040846 (PMC3405063; doi:10.1371/journal.pone.0040846)
Supplement: Table S7 — ANN_BAGGING prediction accuracy benchmarks on the unbound set S23. The dataset and the benchmark measurements have been described in the main text. Matthews correlation coefficient (MCC), F-score(Fsc), Accuracy(Acc), Precision(Pre), Sensitivity(Sen) and Specificity(Spe) are shown in Equations (4)∼(9). TP, FP, TN, and FN are true positive, false positive, true negative, and false negative respectively. Interactive examination of the prediction results for each of the proteins in the 23 unbound test set can be accessed from the web server: http://ismblab.genomics.sinica.edu.tw/> benchmark > protein-carbohydrate. (DOC) [file pone.0040846.s010.doc]

**Table S7**

| **Residue-based ANN_BAGGING prediction benchmarks on unbound set S23** | | | | | | | | | | |
| --- | --- | --- | --- | --- | --- | --- | --- | --- | --- | --- |
| PDBID | Acc | Pre | Sen | Spe | MCC | Fsc | TP | TN | FP | FN |
| 1G0Z | 0.00 | 0.00 | 0.00 | 0.00 | 0.00 | 0.00 | 0 | 105 | 0 | 6 |
| 1M71 | 0.95 | 0.00 | 0.00 | 0.99 | -0.02 | 0.00 | 0 | 196 | 2 | 8 |
| 1MSB | 0.95 | 1.00 | 0.38 | 1.00 | 0.60 | 0.55 | 3 | 98 | 0 | 5 |
| 1NOF | 0.97 | 0.63 | 0.71 | 0.98 | 0.65 | 0.67 | 10 | 317 | 6 | 4 |
| 1O82 | 0.00 | 0.00 | 0.00 | 0.00 | 0.00 | 0.00 | 0 | 52 | 0 | 8 |
| 1R13 | 0.96 | 0.63 | 0.71 | 0.98 | 0.65 | 0.67 | 5 | 126 | 3 | 2 |
| 1TVN | 0.95 | 0.42 | 0.80 | 0.95 | 0.56 | 0.55 | 8 | 219 | 11 | 2 |
| 2BPE | 0.91 | 0.00 | 0.00 | 0.97 | -0.04 | 0.00 | 0 | 109 | 3 | 8 |
| 2FBD | 0.97 | 0.89 | 0.80 | 0.99 | 0.83 | 0.84 | 8 | 100 | 1 | 2 |
| 2J1R | 0.94 | 0.50 | 0.43 | 0.97 | 0.43 | 0.46 | 3 | 114 | 3 | 4 |
| 2UVE | 0.94 | 0.34 | 0.93 | 0.94 | 0.55 | 0.50 | 14 | 452 | 27 | 1 |
| 2WSU | 0.94 | 0.33 | 0.78 | 0.95 | 0.49 | 0.47 | 7 | 261 | 14 | 2 |
| 2X2S | 0.93 | 0.40 | 0.86 | 0.93 | 0.56 | 0.55 | 6 | 122 | 9 | 1 |
| 2XD2 | 0.94 | 0.83 | 0.20 | 1.00 | 0.39 | 0.32 | 5 | 308 | 1 | 20 |
| 2XHH | 0.94 | 0.50 | 0.29 | 0.98 | 0.35 | 0.36 | 2 | 100 | 2 | 5 |
| 2XHN | 0.96 | 0.82 | 0.38 | 1.00 | 0.54 | 0.51 | 9 | 439 | 2 | 15 |
| 3A47 | 0.98 | 0.56 | 1.00 | 0.98 | 0.74 | 0.72 | 14 | 498 | 11 | 0 |
| 3ACF | 0.94 | 0.40 | 0.44 | 0.96 | 0.39 | 0.42 | 4 | 161 | 6 | 5 |
| 3K01 | 0.96 | 0.93 | 0.50 | 1.00 | 0.67 | 0.65 | 13 | 327 | 1 | 13 |
| 3LE0 | 0.96 | 0.56 | 0.83 | 0.97 | 0.66 | 0.67 | 5 | 114 | 4 | 1 |
| 3M9W | 0.94 | 0.33 | 0.88 | 0.94 | 0.52 | 0.48 | 7 | 231 | 14 | 1 |
| 3NSM | 0.97 | 0.29 | 0.50 | 0.98 | 0.37 | 0.37 | 5 | 491 | 12 | 5 |
| 3NV1 | 0.96 | 0.64 | 0.88 | 0.97 | 0.73 | 0.74 | 7 | 121 | 4 | 1 |
| Total | 0.95 | 0.50 | 0.53 | 0.97 | 0.49 | 0.51 | 135 | 5061 | 136 | 119 |

**Table S7:** ANN_BAGGING prediction accuracy benchmarks on the unbound set S23. The dataset and the benchmark measurements have been described in the main text. Matthews correlation coefficient (MCC), F-score(Fsc), Accuracy(Acc), Precision(Pre), Sensitivity(Sen) and Specificity(Spe) are shown in Equations (4)~(9). TP, FP, TN, and FN are true positive, false positive, true negative, and false negative respectively. Interactive examination of the prediction results for each of the proteins in the 23 unbound test set can be accessed from the web server: <http://ismblab.genomics.sinica.edu.tw/>> benchmark > protein-carbohydrate
