# Supplementary material for: Prediction of Carbohydrate Binding Sites on Protein Surfaces with 3-Dimensional Probability Density Distributions of Interacting Atoms
Source: PLoS One. 2012 Jul 25;7(7):e40846. doi: 10.1371/journal.pone.0040846 (PMC3405063; doi:10.1371/journal.pone.0040846)
Supplement: Table S8 — Root mean square deviation (RMSD) between carbohydrate-bound and unbound structure. The structural alignments were performed by PyMOL package. Interactive examination of the superimposed results for each pair of bound and unbound proteins can be accessed from the web server: http://ismblab.genomics.sinica.edu.tw/> benchmark > protein-carbohydrate. (DOC) [file pone.0040846.s011.doc]

**Table S8**

| **DataSet** | **Bound PDBID** | **Unbound PDBID** | **RMSD** |
| --- | --- | --- | --- |
| S88 | 154L | 153L | 0.16 |
| S88 | 1A9T | 1PBN | 0.47 |
| S88 | 1BG9 | 1AVA | 0.41 |
| S88 | 1C1L | 1C1F | 0.16 |
| S88 | 1C3N | 1C3K | 0.19 |
| S88 | 1E3Z | 1E3X | 0.19 |
| S88 | 1E7Y | 1E7M | 0.41 |
| S88 | 1EOM | 1EOK | 0.19 |
| S88 | 1ESW | 1CWY | 0.29 |
| S88 | 1EUS | 1EUR | 0.13 |
| S88 | 1EXA | 2LBD | 0.31 |
| S88 | 1FCV | 1FCQ | 0.59 |
| S88 | 1FWV | 1DQG | 0.22 |
| S88 | 1G0C | 1G01 | 0.14 |
| S88 | 1G1T | 1ESL | 0.23 |
| S88 | 1G94 | 1B0I | 0.34 |
| S88 | 1G97 | 1G95 | 0.66 |
| S88 | 1GJW | 1GJU | 0.3 |
| S88 | 1GOQ | 1GOK | 0.15 |
| S88 | 1HV6 | 1QAZ | 0.18 |
| S88 | 1IA7 | 1IA6 | 0.23 |
| S88 | 1KC3 | 1KBZ | 0.51 |
| S88 | 1KNM | 1KNL | 0.42 |
| S88 | 1KWK | 1KWG | 0.16 |
| S88 | 1L1R | 1L1Q | 0.3 |
| S88 | 1L8T | 1J7I | 1.08 |
| S88 | 1LMQ | 1BB6 | 0.17 |
| S88 | 1LSZ | 1LSY | 0.17 |
| S88 | 1LU1 | 1BJQ | 0.15 |
| S88 | 1LZC | 1LSG | 0.66 |
| S88 | 1LZR | 1C7P | 0.53 |
| S88 | 1MFU | 1JXK | 0.27 |
| S88 | 1MOR | 1MOS | 0.29 |
| S88 | 1MXD | 1MWO | 0.22 |
| S88 | 1OD3 | 1O8P | 0.33 |
| S88 | 1OGO | 1OGM | 0.23 |
| S88 | 1PIG | 1PIF | 0.18 |
| S88 | 1QKQ | 1HDK | 0.15 |
| S88 | 1RWG | 1RWA | 0.34 |
| S88 | 1T10 | 1Q50 | 0.28 |
| S88 | 1TJ4 | 1S2O | 2.76 |
| S88 | 1ULV | 1UG9 | 0.51 |
| S88 | 1UU6 | 1OLR | 0.16 |
| S88 | 1UX7 | 1W0N | 0.4 |
| S88 | 1UXX | 1GMM | 0.33 |
| S88 | 1UY4 | 1UY1 | 0.07 |
| S88 | 1UZ0 | 1UXZ | 0.22 |
| S88 | 1W0O | 1KIT | 0.8 |
| S88 | 1W9W | 1W9S | 0.29 |
| S88 | 1WU5 | 1WU4 | 0.17 |
| S88 | 1X1J | 1X1H | 0.22 |
| S88 | 1ZU0 | 1ZTY | 6.8 |
| S88 | 2B4F | 2A8Z | 0.36 |
| S88 | 2BF6 | 2VK5 | 0.1 |
| S88 | 2BFQ | 1HJZ | 0.27 |
| S88 | 2D3N | 1WP6 | 0.17 |
| S88 | 2E2O | 2E2N | 2.95 |
| S88 | 2G3J | 2G3I | 0.25 |
| S88 | 2HW1 | 2HQQ | 0.27 |
| S88 | 2JEQ | 2JEP | 0.27 |
| S88 | 2O9G | 2O9F | 0.27 |
| S88 | 2POQ | 2O48 | 0.12 |
| S88 | 2QIA | 2JF2 | 0.24 |
| S88 | 2R68 | 2R60 | 0.22 |
| S88 | 2V0I | 2V0H | 0.79 |
| S88 | 2V8K | 2V8I | 0.21 |
| S88 | 2VGD | 2VUJ | 0.35 |
| S88 | 2VJJ | 2VJI | 0.19 |
| S88 | 2VW1 | 2VW0 | 0.58 |
| S88 | 2VX6 | 2VX4 | 0.17 |
| S88 | 2W47 | 2W1W | 0.41 |
| S88 | 2YVP | 2YVM | 0.13 |
| S88 | 2Z1S | 2O9P | 0.26 |
| S88 | 2ZJ3 | 2V4M | 0.42 |
| S88 | 3ABX | 3A64 | 0.1 |
| S88 | 3B9Z | 3BHS | 0.17 |
| S88 | 3BMW | 3BMV | 0.19 |
| S88 | 3BYN | 3BYL | 0.16 |
| S88 | 3C9E | 1U9V | 0.35 |
| S88 | 3CR9 | 1B1X | 0.23 |
| S88 | 3CT5 | 3CSR | 0.15 |
| S88 | 3EWR | 3EWQ | 0.28 |
| S88 | 3FIZ | 3CMJ | 0.22 |
| S88 | 3GNP | 3GNO | 0.15 |
| S88 | 3H3K | 3EZ8 | 0.26 |
| S88 | 3HKN | 1G6V | 0.71 |
| S88 | 3IID | 1ZR3 | 1.05 |
| S88 | 4PFK | 3PFK | 0.23 |
| S23 | 1M7D | 1M71 | 0.4 |
| S23 | 1O84 | 1O82 | 0.35 |
| S23 | 1TVP | 1TVN | 0.27 |
| S23 | 1U4J | 1G0Z | 0.13 |
| S23 | 2CL8 | 2BPE | 0.32 |
| S23 | 2H5Z | 2FBD | 0.22 |
| S23 | 2J1V | 2J1R | 0.16 |
| S23 | 2MSB | 1MSB | 0.44 |
| S23 | 2UVF | 2UVE | 0.24 |
| S23 | 2WT0 | 2WSU | 0.59 |
| S23 | 2X2T | 2X2S | 0.27 |
| S23 | 2XD3 | 2XD2 | 2.6 |
| S23 | 2XFD | 2XHH | 0.27 |
| S23 | 2Y24 | 1NOF | 0.26 |
| S23 | 3A4A | 3A47 | 0.1 |
| S23 | 3ACH | 3ACF | 0.25 |
| S23 | 3K00 | 3K01 | 3.17 |
| S23 | 3LEI | 3LE0 | 0.1 |
| S23 | 3M9X | 3M9W | 0.24 |
| S23 | 3NJV | 2XHN | 0.16 |
| S23 | 3NSN | 3NSM | 0.2 |
| S23 | 3NV3 | 3NV1 | 0.09 |
| S23 | 3PAK | 1R13 | 0.33 |

**Table S8:** Root mean square deviation (RMSD) between carbohydrate-bound and unbound structure. The structural alignments were performed by PyMOL package. Interactive examination of the superimposed results for each pair of bound and unbound proteins can be accessed from the web server: <http://ismblab.genomics.sinica.edu.tw/>> benchmark > protein-carbohydrate
